# Connection of Isolated Stereoclusters by Combining ^13^C-RCSA, RDC, and *J*-Based Configurational Analyses and Structural Revision of a Tetraprenyltoluquinol Chromane Meroterpenoid from *Sargassum muticum*

**DOI:** 10.3390/md20070462

**Published:** 2022-07-18

**Authors:** Juan Carlos C. Fuentes-Monteverde, Nilamoni Nath, Abel M. Forero, Elena M. Balboa, Armando Navarro-Vázquez, Christian Griesinger, Carlos Jiménez, Jaime Rodríguez

**Affiliations:** 1 Departamento de Química e Centro de Investigacións Científicas Avanzadas (CICA), Universidade da Coruña, 15071 A Coruña, Spain; jufu@mpinat.mpg.de (J.C.C.F.-M.); mateo.forerot@udc.es (A.M.F.); 2 NMR Based Structural Biology, MPI for Multidisciplinary Sciences, Am Fassberg 11, 37077 Göttingen, Germany; 3 Department of Chemistry, Gauhati University, Gopinath Bardoloi Nagar, Guwahati 781014, India; nnath@gauhati.ac.in; 4 Department of Chemical Engineering, Faculty of Science, Campus Ourense, University of Vigo, As Lagoas s/n, 32004 Ourense, Spain; elenamba@uvigo.es; 5 Departamento de Química Fundamental, CCEN, Universidade Federal de Pernambuco, Cidade Universitária, Recife 50740-550, Brazil; armando.deus@gmail.com

**Keywords:** *Sargassum muticum*, meroditerpene, coupling constants, *J*-DP4, ^13^C-RCSA, RDC, ECD/OR

## Abstract

The seaweed *Sargassum muticum*, collected on the southern coast of Galicia, yielded a tetraprenyltoluquinol chromane meroditerpene compound known as **1b**, whose structure is revised. The relative configuration of **1b** was determined by *J*-based configurational methodology combined with an i*J*/DP4 statistical analysis and further confirmed by measuring two anisotropic properties: carbon residual chemical shift anisotropies (^13^C-RCSAs) and one-bond ^1^H-^13^C residual dipolar couplings (^1^*D*_CH_-RDCs). The absolute configuration of **1b** was deduced by ECD/OR/TD-DFT methods and established as *3R*,*7S*,*11R*.

## 1. Introduction

*Sargassum muticum* (*SM*), also known as Japanese wireweed, is a brown alga, first described and classified by Yendo in 1907 as *Sargassum kjellmanianum* form *muticum*, based on morphological and ecological differences [1]. In 1955, Fensholt reconsidered this form as sufficiently differentiated from *S. kjellmanianum* and separated it into two species of their own, until it was finally named *Sargassum muticum* (Yendo) Fensholt [2,3].

This species has been the object of a multitude of studies. In 2017, Balboa et al. reported the use of *SM* as a potential antioxidant agent and as a preservative in the preparation of cosmetics [4]. Another work, conducted by Park, investigated the feasibility of using *SM* as a source of bioactive compounds [5]. This study concluded that adding *SM* pills to the normal diet showed liver benefits in addition to decreasing fatigue and stress. These effects were related to bioactive compounds compatible with meroterpenes or fucoxanthines [6]. 

Meroditerpenoids of the chromene, where the polyprenyl moiety is bonded to a hydroquinone or similar aromatic ring [7], constitute a very important family of compounds isolated from *Sargassum*. Almost a hundred meroditerpenes have been reported from marine organisms such as fish, macroalgae, sponges, coelenterates, and tunicates [7,8,9,10]. These compounds have been used in various cancer therapies based on their ability to protect against oxidative damage [11,12].

**Figure 1:**
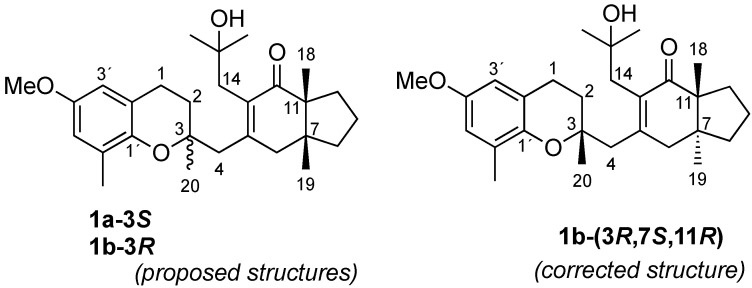
Structures of the meroditerpenoids **1a**–**b**.

In 1993, Praud et al. isolated C3-epimeric meroditerpenoids **1a** and **1b** from *Cystoseira baccata* [13]. They proposed a *cis*-fused chromene ring structure, although the configuration at C3 could not be established at that point. Rapid epimer interconversion was observed in CDCl_3_. Later, Varela et al. also reported antileishmanial activity in those compounds [14]. In 2015, we isolated tetraprenyltoluquinol chromane meroterpenoids **1a** and **1b** as an epimeric mixture at position C3 from the methanolic extract of the alga (See Figure 1). Moreover, we described their use as photoprotective agents used to mitigate skin damage caused by UV exposure [10].

Considering that obtaining the **1a**/**1b** mixture was probably due to the use of CDCl_3_ [10,13,15], we repeated the isolation procedure recording the NMR analysis in CD_2_Cl_2_. Additionally, the relative configuration of two well-separated stereoclusters (C3 and C7/C11) in **1a**–**1b** was determined by *J*-based configurational analysis along with ^13^C-residual chemical shift anisotropies (^13^C-RCSA) [16] and residual dipolar couplings (RDCs) [17].

## 2. Results and Discussion

From the methanolic extract of *SM*, the *n*-hexane soluble fraction was separated by flash column chromatography and then purified by HPLC to obtain one of the epimers at position 3. The 950 MHz NMR spectra collected in CD_2_Cl_2_ did not show any evidence of epimerization for compound **1b**. We observed the epimerization of the pure compound in CDCl_3_ after 25 min. The high resolution mass spectrum showed peaks at *m*/*z* 441.2969 ((M + H)^+^), 463.2811 ((M + Na)^+^), and 903.5741 ((2M + Na)^+^), thus confirming the molecular formula C_28_H_40_O_4_. The ^1^H NMR spectrum of **1b** (Table 1) was then assigned in CD_2_Cl_2_, showing a singlet signal at δ_H_ 3.701, corresponding to the methoxy group attached to the aromatic nucleus. Two doublets, at δ_H_ 6.563 and 6.450, were assigned to aromatic protons, a harmonious relationship then being revealed between the small coupling constant (*J* = 3.0 Hz) and the 1′,2′,4′,6′-substituents *meta*-arranged around the benzene ring. A spin system formed by two contiguous methylene groups was also assigned by observing a ^1^H-^1^H COSY correlation between the protons at δ_H_ 1.805 (*J* = 13.5 Hz and 6.9 Hz) and at δ_H_ 2.774 (*J* = 6.9 Hz) disclosing the remaining chromane moiety. The five singlet signals belonging to methyl groups on *sp*^3^ quaternary carbons were observed at δ_H_ 0.804, 1.041, 1.105, 1.225, and 1.235, along with the three isolated methylene systems at δ_H_ 2.705/2.514 (*J* = 13.7 Hz), 3.028/2.237 (*J* = 18.7 Hz), and 2.570/2.506 (*J* = 14.3 Hz). These three pairs were easily assigned to non-equivalent protons located at C4, C6, and C14. The remaining structure of **1b** was completed by ^1^H and ^13^C data, 2D NMR (^1^H–^1^H COSY, HSQC, HMBC), and mass spectral data (see Figures S3–S13 and Table 1) confirmed the structure of the epimers previously isolated from *Sargassum muticum* and from a macroalgae belonging to the *Cystoseira genus* [13,14].

**Figure 2:**
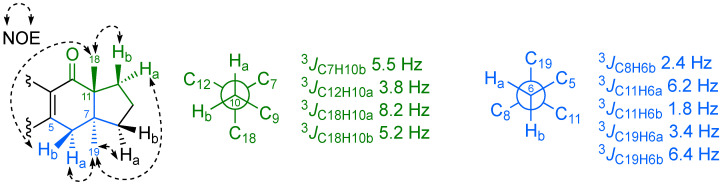
Experimental ^3^*J*_CH_ and NOE correlations observed in the hydrindane skeleton of **1b**.

**Figure 3:**
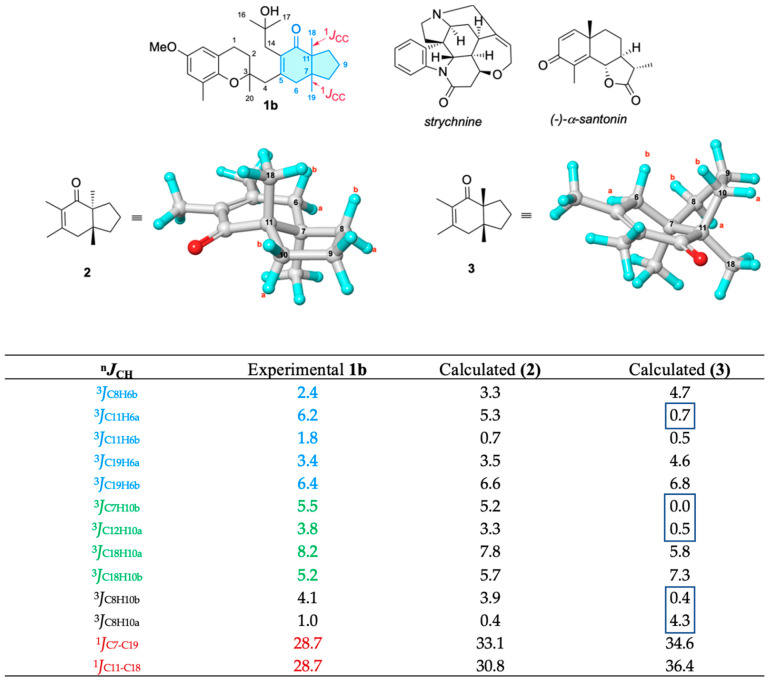
Experimental ^1^*J*_CC_ and ^3^*J*_CH_ values in **1b** extracted from an IPAP-HSQMBC used in the *J*-based configurational analysis vs. DFT-calculated ^1^*J*_CC_ and ^3^*J*_CH_ (GIAO/OLYP/Def2TZV//B3LYP/6-31G(d) gas phase) values in silico models **2** and **3**. Several ^1^*J*_CC_ were measured from a 2D *J*-modulated ADEQUATE experiment. Santonin and strychnine were used as tests. Strongly deviating couplings allowing the assignment of relative configuration are boxed. All vales are expressed in Hz.

Configurational analysis of **1b**. Surprisingly, when we tried to confirm the reported *cis* relationship of the two methyl groups at C7 and C11 in the hydrindane skeleton [10,13,14], we found an unexpected lack of NOE contact between Me18 and Me19 when both were selectively irradiated in a 1D-NOE experiment. A deeper NOE study (see Figure 2), along with a *J*-based configurational analysis [18,19,20,21] was performed through both ^3^*J*_CH_ and ^2^*J*_CH_ coupling constants obtained from IPAP-HSQMBC spectra and ^1^*J*_CC_ from a 2D *J*-modulated ADEQUATE spectrum (see Figure 3) to deduce the bicyclo[4.3.0]nonane fusion present in **1b** (see Figures S14–S22 and Table S4).

The relative configuration of the C6 prochiral protons could be established just by the observation of NOE contacts between H19 and H6a and between H10a and H6a. The NOE correlations observed between H6b/H18 and H10b/H18, and between H6a/H19 and H8a/H10a, suggested that the relative configuration at C7 and C11 must be *trans*-7S*,11R* rather than *cis*. The high ^3^*J*_CH_ values measured between H10a and C18 of 8.2 Hz, and between H6b and C19 of 6.4 Hz in an IPAP-HSQMBC experiment (Figures S14–S18) were in harmony with a *trans* disposition between both methyl groups (see Figure 2).

To discriminate between *cis* and *trans* dimethyl configurations, ^2^*J*_CH_ and ^3^*J*_CH_ couplings were computed on in silico models **2** (*trans*) and **3** (*cis*) (see Figure 3) by using DFT calculations and compared with the experimental values of **1b**. We tested different combinations of functional/basis set/gas phase, and solvent models to obtain heteronuclear couplings as accurate as possible, by using (-)-α-santonin and strychnine as test systems [22,23,24], (see Figure 3). To further improve accuracy, a scaling factor was used in the computations as described in the Supplementary Information (SI).

Overall, 46 DFT methods were examined using an empirical scaling factor to improve the accuracy of the computations (see SI) [25,26,27,28,29,30,31,32,33,34,35]. Heteronuclear ^2^*J*_CH_ and ^3^*J*_CH_ for (-)-α-santonin and strychnine were measured in DMSO-*d*_6_ from IPAP-HSQMBC and HECADE-HSQC experiments. Among all the methodologies tested, the combination GIAO/OLYP/Def2TZV gave the best performance/computation time ratio. Afterwards, the standard deviation (σ) and the coefficient of determination (R^2^) were computed (^2^*J*_CH_: σ = 1.5 Hz, R^2^ = 0.967, ^3^*J*_CH_: σ = 2.3 Hz, R^2^ = 0.978; see all details in SI). The good performance of OLYP is notable considering it is a pure GGA functional that does not include exact exchange. The DFT-calculated heteronuclear coupling constant values involving C7 and C11 in the *trans*-model **2** were in much better agreement with those of **1b** in relation to the theoretical data computed for the *cis*-model **3** (see Figure 3). The *trans* fusion was also confirmed for this hydrindane by measuring the one-bond carbon–carbon scalar coupling constants (^1^*J*_CC_) between C7–C19 and C11–C18 in **1b** from the 2D *J*-modulated ADEQUATE spectrum and comparing them to those theoretically obtained by DFT computation for models **2** and **3** at the same OLYP/Def2TZV level of theory. Both ^1^*J*_C7C19_ and ^1^*J*_C11C18_ of 28.7 Hz betokened the dimethyl *trans* configuration (DFT-calculated ^1^*J*_C7C19_ and ^1^*J*_C11C18_ are 33.1 and 30.8 Hz, respectively).

**Figure 4:**
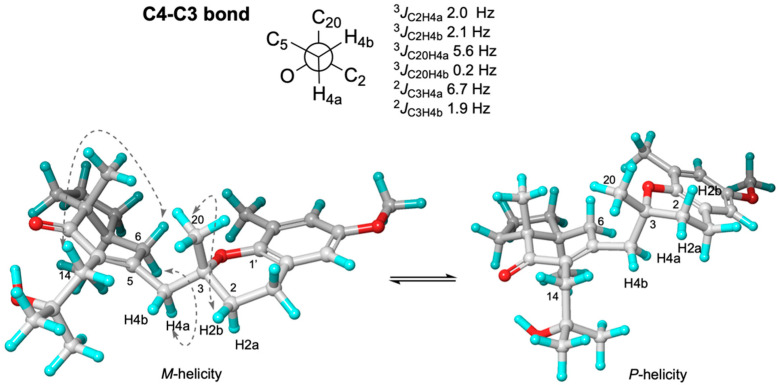
Carbon–proton coupling constants from IPAP-HSQMBC experiments and NOE contacts to relate C3, C7, and C11 stereogenic centers of (3R*,7S*,11R*)-1b (see SI for the diastereoisomer (3S*,7S*,11R*)-1b). ^2^*J*_CH_ couplings were measured as absolute value. See *P-* and *M*-helicities on Figure 8.

Once the relative configuration of 7S*,11R* for **1b** was unequivocally assigned, we allocated its relation to C3. Unfortunately, *J*-based configurational analyses and NOE measurements were not able to discriminate the possible configurations for the two stereoclusters, whether (3R*,7S*,11R*) or (3S*,7S*,11R*) of **1b** (Figure 4 only shows the analysis of 3R*,7S*,11R*; for 3S*,7S*,11R*, see Figure S31).

A conformational equilibrium around the C1′-O-C3-C2 dihedral, leading to two possible helicities, *M*- (C1′-O-C3-C2 < 0°) and *P*- (C1′-O-C3-C2 > 0°), was deduced from the observed NOE correlations observed between H2b/H20 (strong) and H2a/H20 (weak), and from the ^3^*J*_HH_, ^3^*J*_CH_, and ^2^*J*_CH_ coupling constants.

This conformational flip/flop is characteristic of this type of chromane conformer on the dihydropyran ring (see Figure 4, Figures S24 and S25), as it was deduced from the isochronic chemical shifts observed in both H1 and the averaged coupling constant measured for ^3^*J*_H1H2a_ = 6.7 Hz, ^2^*J*_C3H2a_ = 3.4 Hz, and ^2^*J*_C3H2b_ = 2.8 Hz (see Figure S21). From the observed coupling constants, we were able to deduce an equilibrium between *P*-helicity and *M*-helicity conformers in a 3:2 ratio.

Trying to establish the relative configuration at C3, we followed the i*J*/DP4 protocol of Daranas, Sarotti et al., involving a conformational search within a 5-kcal/mol interval, and then calculated the theoretical chemical shifts at the B3LYP/6-31G(d,p) level [36,37,38]. Contrasting calculated vs. experimental chemical shifts gave rise to a better DP4 score for the (3R*,7S*,11R*)-1b diastereoisomer (C/DP4: 94.25%, H/DP4: 100%). Next, ^3^*J*_C2H4a_ = 2.0 Hz, ^3^*J*_C2H4b_ = 2.1 Hz, ^3^*J*_C20H4a_ = 5.6 Hz, ^3^*J*_C20H4b_ = 0.2 Hz, ǀ^2^*J*_C3H4a_ǀ = 6.7 Hz, and ǀ^2^*J*_C3H4b_ǀ = 1.9 Hz from IPAP-HSQMBC experiments (see Newman projection on Figure 4) were read into the application of the i*J*/DP4, implying four dihedral angle restrictions around the C3–C4 bond, to wit: dihedral O-C3-C4-H4b (180° ± 15°), dihedral O-C3-C4-H4a (±60° ± 15°), dihedral C20-C3-C4-H4a (180° ± 15°), and dihedral C20-C3-C4-H4b (±60° ± 15°).

**Figure 5:**
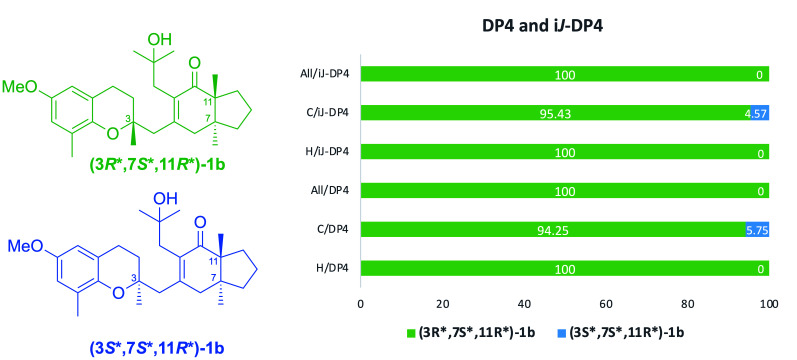
i*J*-DP4 statistical analysis of (3R*,7S*,11R*)-1b and (3S*,7S*,11R*)-1b.

Using iJ/DP4, we fully predicted the (3R*,7S*,11R*)-1b isomer, a slight 95.43% improvement being gained at ^13^C and a complete one at ^1^H (see Figure 5). Most populated conformers in both plausible configurations in the DP4 ensembles, generated by an unrestricted conformational search, satisfy the main constraints of chromane helicities, prochiral protons at C4 and C14, and weak hydrogen bonding between OH at C15 and C12 (C=O).

Similarly, the forementioned set of conformers of plausible configurations of **1b** was studied by applying the Computer-Assisted 3D Structure Elucidation (CASE-3D) strategy, as implemented in MNova StereoFitter [39,40], which makes no a priori assumptions about the conformational space. Only isotropic NMR data were utilized to elucidate the relative configuration of **1b**. Both constitutions were ranked according to chemical shift predictions at the DFT level (GIAO/B3LYP/6-31G(d,p)) and ^3^J_CH_ coupling computed by Karplus-like equations. The CASE-3D analysis scored the (3R*,7S*,11R*)-1b configuration slightly better (See Figure S42).

Anisotropic measurements of **1b**: ^13^C-RCSA and ^1^D_CH_-RDC**.** To revalidate the structure of **1b** deduced from chemical shift-based configurational analyses, anisotropic parameters such as carbon residual chemical shift anisotropy (^13^C-RCSA) were utilized to relate both stereoclusters, separated from each other by four bonds. The RCSAs are manifested by the change in the chemical shift when the analyte is placed in a weak alignment medium such as a gel or a liquid crystal. The carbon RCSAs provide the relative orientations of the structural moieties, including those of non-protonated carbons C3, C7, and C11 as in our case study [16,41,42].

After an unrestricted geometry search, we then subjected all conformers to gas phase geometry optimization at B3LYP/6-31G+(d,p) for plausible configurations (3R*,7S*,11R*)-**1b** and (3S*,7S*,11R*)-**1b**. Subsequently, from DFT atomic coordinates, we selected those optimized conformers fitting short-range NMR data (J-couplings, chemical shifts, nuclear Overhauser effects (NOEs)) [43].

**Figure 6:**
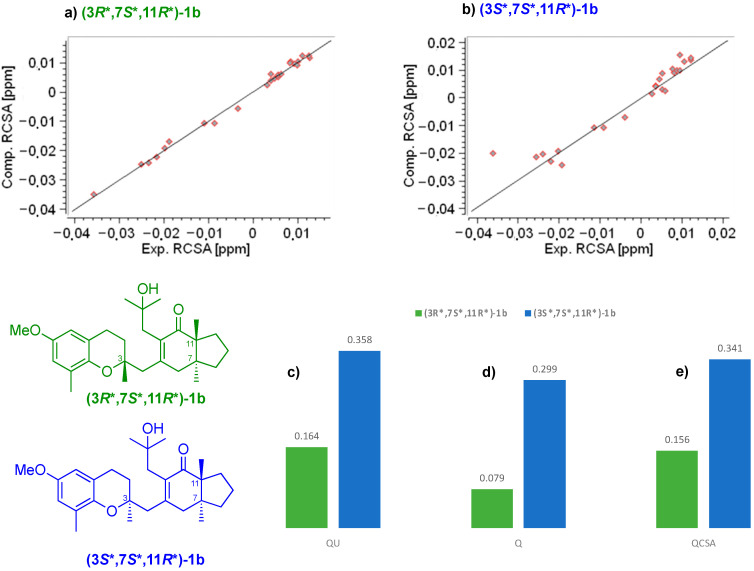
Anisotropic data (^13^C-RCSA) of meroditerpene **1b** swollen in 70/0.25 PMMA-*d*_8_ gel (200 MHz, CD_2_Cl_2_. (**a**,**b**) Fitting for carbon residual chemical shift anisotropies of diastereoisomers (3R*,7S*,11R*)-1b (green) and (3S*,7S*,11R*)-1b (blue), respectively. (**c**) *Q_U_*: quality factor of uncorrected RCSAs; (**d**) *Q* and (**e**) *Q*_CSA_ quality factors for the configurations.

The conformational ensemble used for this analysis is shown for the correct configuration in Figure 6. Twenty-eight ^13^C-RCSA values were measured using an in-house made 3 mm compression device and compressible PMMA-d_8_ gel. Before applying the former to the RCSA data collection of **1b**, its performance was tested using estrone as a standard sample (see Section S6 in SI). RCSAs were induced although small isotropic chemical shift changes also ensued because of the change in the solvent to gel ratio under compressed conditions (See Figure S32) [16]. These ΔRCSA values, not corrected for isotropic contributions, ranged between −7.4 and 5.5 Hz (see Table S7). Aromatic and carbonyl carbons exhibited larger ΔRCSAs than aliphatic carbons, reflecting the respective sizes of the CSA tensors [16,40]. A higher error in the fitting curve was noticed in the (3S*,7S*,11R*)-1b diastereoisomer relative to the other plausible configuration, even though automatic isotropic shift correction was not applied (Figure 6c and Figure S38).

When we corrected for isotropic shifts, a better agreement was achieved between the experimental and back-calculated ^13^C-RCSAs for (3R*,7S*,11R*)-1b (Q(Q_CSA_) 0.079 (0.156)) as compared to (3S*,7S*,11R*)-1b (Q(Q_CSA_) 0.299 (0.341)) (Figure 6a,d,e). Furthermore, a relative difference in Q between both diastereomers was increased (ΔQu = 0.19; ΔQ = 0.22) (Figure 6c,d).

Consequently, we were able to differentiate between both plausible diastereomers by ^13^C RCSA and to establish the relative configuration between the stereocenter at C3 and the stereocluster C7–C11 located four bonds away.

Data discrimination was tested by two resampling methods, with and without replacement, to wit: Monte Carlo bootstrapping [16,22] and leave-one-out cross-validation (Jackknife) [44]. Both bell curves derived from bootstrapping and resampled influence bar-plotted from Jackknife clearly indicate that ^13^C-RCSA data unambiguously confirmed (3R*,7S*,11R*)-**1b** as the correct relative configuration (Figures S39 and S40).

One-bond ^13^C-^1^H residual dipolar couplings (^1^D_CH_-RDC) measurements were also conducted to confirm the relative configuration of **1b**. Thus, a CLIP/CLAP-HSQC, spurred by non-uniform sampling [45,46] with a 1.6 mg sample, and using a deuterated PMMA-d_8_ (70/0.25) in a low viscosity solvent (CD_2_Cl_2_), was measured to obtain RDC values. The accuracy of the ^1^D_CH_ values was enhanced as there was almost no interference from deuterated polymer background signals.

**Figure 7:**
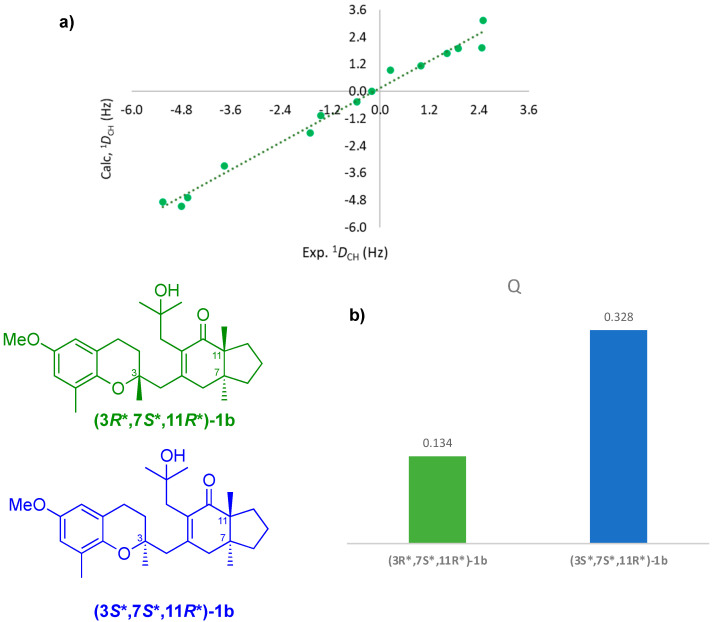
RDC fitting curve of (3R*,7S*,11R*)-1b (**a**) and *Q* factor found for (3R*,7S*,11R*)-1b (green) and (3S*,7S*,11R*)-1b (blue) (**b**).

Seventeen RDCs were meticulously measured thanks to the narrow spectral line, resulting in ^1^D_CH_ values within a range of between 3.0 and −6.7 Hz. RDC data were fitted on an experimentally constrained ensemble of conformers for both plausible configurations as in the RCSA analysis (See Figure 7).

Fitting the ^1^D_CH_ data, determining and aligning the tensor, and calculating Cornilescu’s quality factor Q were carried out through single-tensor approximation on the MSpin-RDC software. Configuration (3R*,7S*,11R*)-1b showed a Q of 0.134, while (3S*,7S*,11R*)-1b had a Q of 0.328, the discrimination presenting a degree of linearity between experimental and back-calculated values (see Figure 7b). By using ^1^D_CH_-RDC, configuration (3S*,7S*,11R*)-1b was removed from consideration with ease, showing the efficacy of the RDC methodology for establishing relative orientations of two distant stereoclusters of meroditerpene **1b**. 

Absolute configuration of **1b.** Finally, we resorted to chiroptical methods (ECD/OR) to assign the absolute configuration (AC) of **1b.** We used the empirical chromane helicity rule [47,48] and compared the experimental chiroptical data with predicted ECD/ORD from ab initio time-dependent DFT (TD-DFT). Currently, chiroptical methodologies associated with quantum chemical calculations are some of the most powerful tools for elucidating stereochemistry and examining even minute changes in the geometry of chiral molecules [49,50,51]. Determining the AC of this framework is also possible by following a chromane helicity rule [47,48]. This rule, established by Crabbé and related to ^1^L_b_ excitation of the chromophore, relates the sign of the ECD band with the helicity of the trisubstituted dihydropyran ring [48] at around 270–290 nm. An extra requirement for the application of the rule is that the largest substituent at the C3 carbon atom should favorably occupy an equatorial position. This rule was exhaustively studied in 2014 by Górecki and Frelek, who revised both the scope and limitations of comparing the experimental ECD spectra with those simulated by TD-DFT, and should, therefore, be used with great caution [52,53]. In our case, following Crabbé’s rule, the ECD curve strongly depends on the population ratio of **1b** conformers with the methyl group at C3 either in axial or equatorial positions.

**Figure 8:**
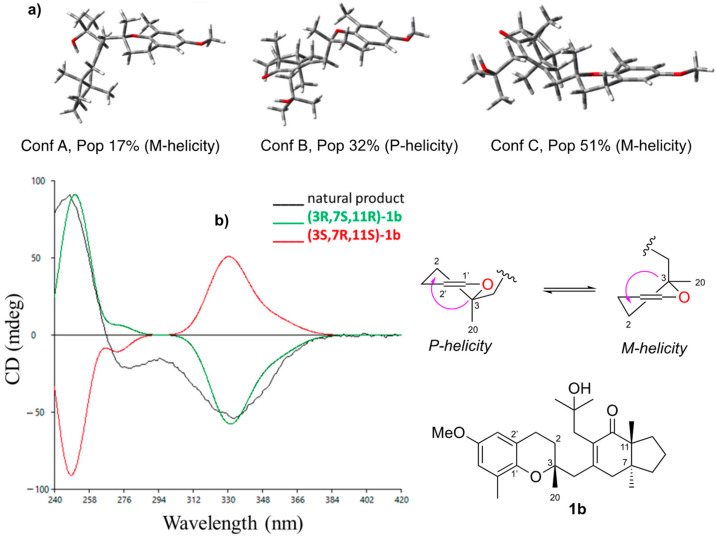
Assignment of the absolute configuration of compound **1b** in equilibrium with *P*-helicity and *M*-helicity conformers (**a**), by comparison of the experimental CD curve in CH_2_Cl_2_ (black) with the theoretically predicted CD spectra (green and red) computed at HSE06/6-311G+(2d,p)/DGA1 level of theory (**b**).

Both the experimental and calculated ECD spectra as well as the OR value were applied to establish the AC of the stereoisomeric pairs exhibiting opposite ECD and OR. Two different levels of theory were chosen to calculate ECD curves: PBE0/Def2TVZ/W06 and HSE06/6-311+G(2d,p)/DGA1 basis sets, with 50 and 38 transition states, respectively, and COSMO-IEFPCM as solvent models (CH_2_Cl_2_). Populations were calculated through Q factor minimization by NMR anisotropy constraints [54,55,56]. Calculations at two levels of theory clearly reproduced the 240–290 nm range of the ECD of **1b**, demonstrating an equilibrium between conformers with M- (68%) and P- helicities (32%) with an excellent agreement towards the (3R,7S,11R) AC for **1b** (see Figure 8). Figure 8a depicts the conformers found for the correct configuration of **1b** along with their populations, derived from the analysis of NMR anisotropic parameters.

The specific optical rotation (${\left[\alpha \right]}_{D}^{25}$) of **1b** was experimentally measured, −11.67 (c 0.12, CH_2_Cl_2_), and then computed at CAM-B3LYP/6-311++G(2d,2p)/DGA1 DFT level. The IEFPCM (CH_2_Cl_2_) solvation effect model and the conformer population were the same as with the ECD calculation (Figure 8). After comparing the experimental results with the DFT computed values, the sign was well reproduced, although the ${\left[\alpha \right]}_{D}^{25}$ was overestimated for **(3R,7S,11R)-1b** −49.4 (see Table S8). The absolute signs indicate that our approach is suitable for correlating the absolute configuration of molecules with rather restricted flexibility to the sign of the ${\left[\alpha \right]}_{D}^{25}$. Both the ECD and the optical rotation approaches agree with the same identifications of **1b** as 3R,7S,11R [57].

## 3. Materials and Methods

### 3.1. General

ECD and UV spectra were recorded on a JASCO J-815 CD spectrometer. The ECD spectrum of **1b** was recorded in the region from 225 to 470 nm at a concentration of 0.2 mg/mL (2.88 × 10^−4^ M) in CH_2_Cl_2_ in a 1 mm cell, totaling five accumulations at a scan rate of 20 nm/min and a temperature of 25 °C. Specific optical rotation (OR) of **1b** was recorded on a Jasco DIP-1000 polarimeter at 589 nm (Na lamp) at a concentration of 0.12 g/100 mL in CH_2_Cl_2_ in a 1 dm/SiO_2_ cuvette (25 °C). NMR spectra were recorded on either a 950, 900, 800, or a 700 MHz Bruker, all the signals being referenced to ^13^C (54.00 ppm) and ^1^H (5.320 ppm) signals of CD_2_Cl_2_. HR-MS were obtained on a Thermo Scientific LTQ Orbitrap XL mass spectrometer (Thermo Fisher Scientific, Waltham, MA, USA). Semipreparative HPLC was performed on an Agilent column (RP-C18 column 10 × 100 mm; 4.6 mL/min). TLC was performed on silica gel (Merck, Kieselgel 60 F_254_) plates; the spots were visualized by exposure to UV light (254 nm). Column chromatography was carried on silica gel (Merck, Kieselgel 60).

### 3.2. Data Processing

Experimental and calculated ECD and UV data were handled with SpecDis V 1.7.1 developed by T. Bruhn [58]. NMR data were processed and analyzed by Bruker TopSpin Software V 4.1.1. Graphics were carried out on Microsoft Excel 365. Geometrical optimization and TDSCF calculation were carried out with the Gaussian 16 Suite. Molecular structures were drawn in ChemDraw V 12.0 and Avogadro V 1.0 [59]. Orbitrap files were processed using Xcalibur™ V 3.0 Software—Thermo Fisher Scientific—and its chemical molecular generator module was employed to provide elemental formulas. Conformer weighing by isotropic constraints was handled by the StereoFitter module nested in MNova Suite [39,40]. Anisotropic NMR and *J*-coupling data processing were performed using MSpin-RDC V 2.6.1.

### 3.3. Raw Material

The seaweed *Sargassum muticum* (*SM*) was collected on a rocky shore on the southern coast of Galicia, Praia da Mourisca (Alcabre, Spain) during the summer of 2011. Specimens were washed carefully with water and then oven-dried at 50 °C for 72 h to preserve them until use. Dried algae were milled to facilitate sample handling and provide higher extraction yields by increasing the contact surface. First, it was milled in a cutting mill up to a particle size of 1–2 cm length and then further milled to obtain a coarse powder.

### 3.4. Extraction and Isolation

The raw methanolic extract of *SM* (4.9 g) was dissolved in methanol/water (1:10) and subsequently partitioned with: *n*-hexane (FH), dichloromethane (FD), *n*-butanol (WB), and water (WW) [60]. Fractions were concentrated under reduced pressure (temp: 32 °C); yielded FH 1.1 g, FD 0.2 g, WB 0.3 g, and WW 2.1 g. The FH (1.1 g) was subjected to NMR-guided fractioning through flash silica gel column chromatography (25 × 2 cm; 80 mL/min) using a stepwise gradient of hexane/Et_2_O and Et_2_O/AcOEt to produce 30 fractions, which were grouped by TLC. Fraction 19 was eluted with a 17:8 Hex/Et_2_O solution.

#### Purification of **1b**

Fraction 19 (Fr. 19) showed doublet (*d*) belonging to two meta-coupled aromatic protons (protons 3′ and 5′) in an aromatic ring part of the bicycling system (6.412–6.533 ppm; *J* = 3.0 Hz) characteristic of the meroditerpenes. F.19 (103 mg) was further purified by semipreparative HPLC (Atlantis RP-C18 column 10 × 100 mm; 4.6 mL/min) equipped with a variable wavelength detector (VWD) at 320 nm, an Agilent pump supplying the following solvent profile: 2.2 min isocratic step (80 *v*/*v*% ACN/H_2_O) and 12.3 min gradient step (from 80 to 100 *v*/*v*% ACN/H_2_O) to afford compound **1b** (27.4 mg; Rt = 5.17 min).

### 3.5. Computational Section 

Conformational searches for *J*-DP4 and CASE-3D analyses were performed by employing MAESTRO software, using an energy window of 5 kcal/mol**.** Energy cut-off (5 kcal/mol) for CASE-3D was based on calculations at B3LYP/6-31G(d,p), solvent model = IEFPCM (CH_2_Cl_2_). Conformational searches for NMR anisotropy analyses were carried out on relevant rotatable bounds ρ1, ρ2, ρ3, and ρ4 (See Figures S21–S27) using the grid search module in PCModel V10. Both *P*- and *M*- helicities were considered. Conformers used on NMR anisotropy analyses were optimized with DFT calculations at B3LYP/6-31+G(d) level, with vibrational frequency calculations confirming the presence of minima, using the Gaussian16 program. Chemical shielding tensors (CST) were computed at the GIAO/MPW1PW9/6-311+G(2d,p) (in gas phase). *J*-couplings were computed either at DFT level or by using Karplus-like equations [61,62]. All chiroptical properties were calculated as Boltzmann averages, weighted with conformer population factors obtained from NMR anisotropic *Q* factor minimization. Time-dependent DFT calculations were performed for each configuration using the combinations PBE0/Def2TZV/W06, solvent model: COSMO, 50 excited states and HSE06/6-311+G(2d,p)/DGA1; solvent model: IEFPCM, 38 excited states. In both, solvent CH_2_Cl_2_ parameters were used. ECD spectra were generated using the program SpecDis by applying a Gaussian band shape with a 0.18 eV width and 25 blue shifts to facilitate comparison to the experimental data. OR values were computed, for both configurations, at CAM-B3LYP/6-311++G(2d,2p)/DGA1 (IEFPCM = CH_2_Cl_2_) level of theory at 589 nm.

#### J-Coupling Calculation

(-)-(α)-santonin and strychnine were purchased at Sigma-Aldrich and both molecular models were optimized based on RCSA/RDC and NOE, respectively, as described before [22]. For strychnine, two conformers were taken into account as found by Butts et al. [24] for ^2,3^J_CH_ benchmarking at different levels of theory. J-coupling data were handled using the CST module nested in MSpin (See SI).

### 3.6. ^13^C-RCSA Measurements

**Figure 9:**
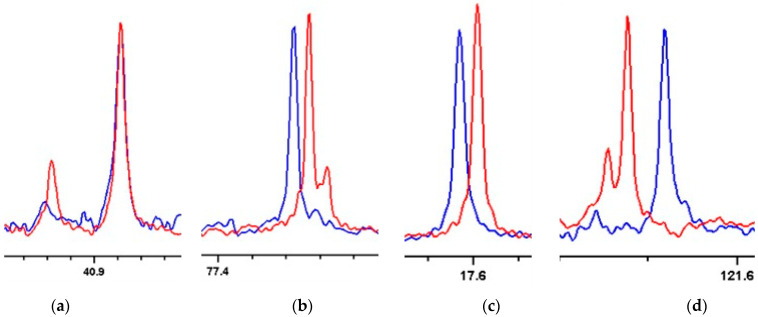
(**a**–**d**) shows resonances from the ^13^C-{^1^H} 200 MHz NMR spectra for meroditerpene **1b** under minimum (Δδ_i,min_; red) and maximum (Δδ_i,max_, blue) compression. The C14 resonance shown in panel (**a**) was used as the reference resonance. Note the presence of both isotropic (marked with an asterisk) and anisotropic signals for some carbons. Spectra recorded with minimum alignment were recorded under complete relaxation of the PMMA-*d*_8_ gel. (∆H_Q_ = 4.2 Hz).

A first attempt to establish the relative configuration of meroditerpene **1b** by ^13^C RCSA involved the use of a 4 mg sample in protonated PMMA swollen in CD_2_Cl_2_ and using a conventional 5 mm outer diameter compression device. A number of resonances were obscured by the polymer background signal (see Figures S35–S43 and Table S6). Therefore, 2.2 mg of **1b** was dissolved in CD_2_Cl_2_ and swollen into a deuterated chemically cross-linked poly(methylmethacrylate) (PMMA-d_8_) gel in an in-house made 3 mm compression device. RCSA were extracted as the difference in the referenced chemical shift between signals at maximum and minimum compression (^13^C-RCSA_i_ = Δδ_i,max_ − Δδ_i,min_) [63]. Due to the inherent properties of the compression device, the gel was surrounded by a layer of isotropic solution; carbon signals were phenomenologically observed from within (Δδ_i,min_) and without (Δδ_i,iso_) the gel (asterisk marked in Figure 9) [16]. After compressing the gel, the analyte concentration inside the gel increased, changing the isotropic contribution of the chemical shifts, and hindering the precise extraction of RCSAs without compensating for these isotropic shift contributions [16]. Therefore, an isotropic shift contribution of gel, expressed as free parameter c, was computed by applying an automatic linear correction [64] (Equation (1)). A quality factor computed without using this means is considered as uncorrected (Qu); it tends to have less discriminating power or even give ambiguous results. Δδ_i_ represents the referenced chemical shift with respect to a selected resonance at the corresponding conditions. Whenever possible, a nucleus with low associated anisotropy, a methylene group, for instance, was taken (Equation (2)). Data were fitted in units of ppm to obtain reasonable weight [63].

^13^C-RCSA_i_ = Δδ_i,max_ − Δδ_i,min_ – c × (Δδ_i,min_ − Δδ_i,iso_) (1)

Δδ_i,max_ = δ_i,max_ − δ_ref,max_ (2)

The quality of the fitting is scored in terms of quality factor Q. When carbon chemical shift variations due to analyte concentration changes are not considered, the uncorrected quality factor is named as Q_U_. To yield minimum Q factor [65], free parameter c was minimized simultaneously along with the alignment tensor during the fitting procedure, and RCSA was fitted by singular value decomposition [63,66]. Conformers were fitted using a multiconformer single tensor approach and distances among the heavy atoms were minimized. The unscaled quality factor (Q) and the chemical shift anisotropic weighted quality factor (Q_CSA_) were computed using Equations (3) and (4), respectively [16].

(3) Q=(∑i=1n(C13 RCSAexp,i−C13 RCSAcalc,i)2∑i=1nC13 RCSA2i)12

(4) QCSA=(∑i=1n([C13 RCSAexp,i−C13 RCSAcalc,i]/CSAi,ax)2∑i=1n(C13 RCSAi/CSAi,ax)2)12

Data robustness was addressed by either bootstrapping analysis or Jackknife resampling as implemented in MSpin software and Excel [67].

Some significant variations in carbon chemical shifts in the oriented media were observed (see Figure 9). A certain amount of isotropic analyte was always detected because the gel in the compression device did not completely fill the entire sample space, even under maximum compression [68]. As before, the isotropic signals were easily distinguishable from their anisotropic counterparts, as their intensities diminished upon compression.

## 4. Conclusions

We demonstrated that the use of an integrated approach combining isotropic and anisotropic NMR means and incorporating chiroptical methods is a perfect toolbox to deduce the three-dimensional structure containing stereoclusters that cannot be connected through local correlations.

We also detected, through ^3^J_HH_, ^2^J_CH_, ^3^J_CH_, coupling constants and RDC, flip-flop conformational changes around the C1′-O-C3-C2 dihedral. Using this approach, the structure of the tetraprenyltoluquinol chromane meroterpenoids **1b** isolated from Sargassum muticum was corrected to the trans fusion dimethylbicyclo [4.3.0]nonane system; and the distant stereocenter at C3 was related to C7 and C11 with the use of DP4, iJ-DP4, CASE-3D, ^1^D_CH_-RDC, and ^13^C-RCSA methodologies. Finally, a set of chiroptical methods (ECD and OR) and of DFT calculations allows the absolute configuration of **1b** to be assigned as (3R,7S,11R). This is the first time this kind of terpenoids has been well studied by anisotropic NMR methodology, which unambiguously confirms the relative configuration of two distant stereoclusters separated by four covalent bonds.

## Figures and Tables

**Table 1:** NMR spectroscopic data for compound **1b** (CD_2_Cl_2_).

	1b	
Position	δ_C_, *mult*. ^a,b^	δ_H_, *mult*., *J* (in Hz) ^c^
1	23.06 CH_2_	2.774 (t, 6.9)
2	34.04 CH_2_	H2a: 1.859 (dt, 13.5, 6.9)
		H2b: 1.805 (dt, 13.5, 6.9)
3	76.80 qC	-
4	45.15 CH_2_	H4b: 2.705 (d, 13.7)
		H4a: 2.514 (d, 13.7)
5	155.10 qC	-
6	44.77 CH_2_	H6b: 3.028 (d, 18.7)
		H6a: 2.237 (d, 18.7)
7	45.25 qC	-
8	35.38 CH_2_	H8b: 1.754 (m)
		H8a: 1.520 (m)
9	19.32 CH_2_	1.744 (m)
10	30.03 CH_2_	H10a: 1.944 (td, 12.0, 11.9, 6.8)
		H10b: 1.441 (ddd, 13.1, 8.4, 3.1)
11	55.42 qC	-
12	209.32 qC	-
13	133.45 qC	-
14	40.37 CH_2_	H14b: 2.570 (d, 14.3)
		H14a: 2.506 (d, 14.3)
15	71.17 qC	-
Me16-	31.88 CH_3_	1.235 (s)
Me17-	29.08 CH_3_	1.041 (s)
Me18-	21.51 CH_3_	1.105 (s)
Me19-	22.71 CH_3_	0.804 (s)
Me20-	24.28 CH_3_	1.225 (s)
1′	145.77 qC	-
2′	121.16 qC	-
3′	111.58 CH	6.450 (d, 3.0)
4′	153.13 qC	-
5′	115.64 CH	6.563 (d, 3.0)
6′	127.43 qC	-
MeO-4′	55.97 CH_3_	3.701 (s)
Me-6′	17.08 CH_3_	2.165 (s)
OH	-	3.983 (br s)

^a^ Multiplicities inferred from DEPT-135 and HSQC experiments. Solvent as internal standard s: singlet, d: doublet; dd: doublet of a doublet; t: triplet; m: multiplet. ^b^ Measured at 200 MHz. ^c^ Measured at 950 MHz.

## Data Availability

All data can be obtained from JR or CJ at CICA at Universidad da Coruña.

## Supplementary Materials

The following supporting information can be downloaded at: https://www.mdpi.com/article/10.3390/md20070462/s1.
