# Supplementary material for: Connection of Isolated Stereoclusters by Combining 13C-RCSA, RDC, and J-Based Configurational Analyses and Structural Revision of a Tetraprenyltoluquinol Chromane Meroterpenoid from Sargassum muticum
Source: Mar Drugs. 2022 Jul 18;20(7):462. doi: 10.3390/md20070462 (PMC9319238; doi:10.3390/md20070462)
Supplement: Supplementary file 1 [file marinedrugs-20-00462-s001.zip › marinedrugs-1805055-supplementary.pdf]

Supplementary Material

Connection of Isolated Stereoclusters by Combining ^{13}C-RCSA, RDC, and *J*-Based Configurational Analyses and Structural Revision of a Tetraprenyltoluquinol Chromane Meroterpenoid from *Sargassum muticum*

Juan Carlos C. Fuentes-Monteverde[1,2], Nilamoni Nath[3], Abel M. Forero[2], Elena Balboa[4], Armando Navarro-Vázquez[5], Christian Griesinger[2*], Carlos Jiménez[1*], and Jaime Rodríguez[1*]

[1]Departamento de Química e Centro de Investigacións Científicas Avanzadas (CICA), Universidade da Coruña, 15071 A Coruña, Spain
[2]NMR based Structural Biology, MPI for Multidisciplinary Sciences, Am Fassberg 11, 37077 Göttingen, Germany
[3]Department of Chemistry, Gauhati University, Gopinath Bardoloi Nagar, Guwahati-781014, India
[4]Department Chemical Engineering, Faculty of Science, Campus Ourense, University of Vigo, As Lagoas s/n, 32004 Ourense, Spain
[5]Departamento de Química Fundamental, CCEN, Universidade Federal de Pernambuco, Cidade Universitária, Recife, Brazil

Table of Contents

1. Isolation of merodijterpene 1b 7

Figure S1. *^{1}H Spectrum (300 MHz, CD_2Cl_2) of fraction 19 (Fr. 19) from flash chromatography column (a), inset shows the characteristic doublet belonging to two meta-coupled aromatic protons from the meroditerpene skeleton (b) (300 MHz, CD_2Cl_2).* 7

Figure S2. *HPLC isolation of compound **1b** (5.17 min) from Fr. 19. Red line indicates solvent gradient.* 7

2. Spectra of meroditerpene 1b 8

Figure S3. *HRMS spectrum of compound **1b**.* 8

Figure S4. *Experimental ECD (a) and UV (b) spectra of **1b** recorded in CH_2Cl_2 (c 0.2 mg/mL). Data collection parameters: data pitch of 1 nm, scanning speed of 20 nm/min, 5 accumulations, wavelength range was 224-470 nm and cell path length 1 mm. Curve smoothing algorithm used was a 3-wavelength center moving average with 4 iterations.* 8

Figure S6. *Pure shift ^{1}H NMR spectrum of compound **1b** (CD_2Cl_2, NS: 32, 800 MHz). Inset shows assignment of H8b, H9, H4b and H14a.* 9

Figure S7. *^{13}C {^{1}H} NMR spectrum of compound **1b** (zgdc30, CD_2Cl_2, NS: 6 K, 200 MHz).* 10

Figure S8. *DEPT 135 spectrum of compound **1b** (dept135, CD_2Cl_2, NS 800, 200 MHz).* 10

Figure S9. *Pure shift-HSQC spectrum of compound **1b** (CD_2Cl_2, NS: 32, 800 MHz). Parameters: $^1J_{CH}$ = 130 Hz. (NUS: 25%/512/128). The spectrum on top is a normal ^{1}H spectrum.* 11

Figure S10. *^{13}C-^{1}H HMBC spectrum of compound **1b** (hmbcetgpnd, CD_2Cl_2, NS: 16, 800 MHz). Parameters: $^nJ_{CH}$ = 8 Hz.* 11

Figure S11. *^{1}H-^{1}H-DQFCOSY spectrum of compound **1b** (cosyqf45, CD_2Cl_2, 800 MHz).* 12

Figure S12. *NOESY spectrum of compound **1b** (a). Relevant NOESY contacts are shown in insets b) and c). (Noesyetgp, CD_2Cl_2, 800 MHz, mixing time = 300 ms).* 13

3. Benchmarking DFT methods for the calculation of $^1J_{CC}$, $^2J_{CH}$, $^3J_{CH}$ 15

Table S1. *Experimental spin-spin carbon proton long range couplings ($^{2,3}J_{CH}$) of minus-alpha-santonin and strychnine in isotropic solution (DMSO-d_6) $^\gamma$* 16

Table S2. *Derived fitting parameters for the spin-spin carbon proton long range couplings ($^{2,3}J_{CH}$) in isotropic solution (DMSO-d_6). Fitting done over unsinged values* 17

Table S3. *Scaling factor correction for $^nJ_{XH}$ (X=H, C) computed at MPW1PW91/6-311+G(2d,p) (gas phase) level of theory used in the J-based analysis of **1b**.* 17

4. *J*-Based Configurational Analysis and NOE Measurements 18

Figure S14. *2D HSQMBC-IPAP spectrum (optimized to 6 Hz) after selective inversion of **H6b** (3.028 ppm) proton of meroditerpene **1b** (a). IPAP-HSQMBC α (blue) and β (red) multiplets of the cross-peaks involving C13 (6.0 Hz), C8 (2.4 Hz), C19 (6.4 Hz) and C15 (1.8 Hz) (b-e). Measurement of $^3J_{CH}$ and $^5J_{CH}$ values were performed by analysis of IPAP multiplet patterns. NS: 32. NUS: 30%/640/96.* 18

Figure S15. *2D HSQMBC-IPAP spectrum (optimized to 6 Hz) after selective inversion of **H6a** (2.237 ppm) proton of meroditerpene **1b** (a). IPAP-HSQMBC α (blue) and β (red) multiplets of the cross-peaks involving C13 (5.2 Hz), C19 (3.4 Hz), C11 (6.2 Hz) and C15 (1.4 Hz) (b-e). Measurement of $^3J_{CH}$ and $^5J_{CH}$ values were performed by analysis of IPAP multiplet patterns. NS:32. NUS: 33%/640/104.* 18

Figure S16. *2D HSQMBC-IPAP spectrum (optimized to 6 Hz) after selective inversion of **H10b** (1.441 ppm) proton of meroditerpene **1b** (a). IPAP-HSQMBC α (blue) and β (red) multiplets of the cross-peaks*

involving C18 (5.2 Hz), C8 (4.1 Hz) and C7 (5.5 Hz) (b-d). Measurement of $^{3}J_{CH}$ values were performed by analysis of IPAP multiplet patterns. NS: 32. NUS: 23%/640/73. 19

Figure S17. *2D HSQMBC-IPAP spectrum (optimized to 6 Hz) after selective inversion of* ***H10a*** *(1.944 ppm) proton of meroditerpene* ***1b*** *(a). IPAP-HSQMBC α (blue) and β (red) multiplets of the cross-peaks involving the C12 (3.8 Hz), C18 (8.2 Hz) and C8 (1.0 Hz) (b-c). Measurement of $^{n}J_{CH}$ values were performed by analysis of IPAP multiplet patterns. $^{3}J_{C8H10a}$ was extracted from an HSQMBC-IPAP spectrum optimized to 1.5 Hz (d). NS: 32. NUS: 23%/640/73.* 19

Figure S18. *2D HSQMBC-IPAP spectrum (optimized to 2 Hz) after selective inversion of* ***H6a*** *(2.237 ppm) proton of meroditerpene* ***1b*** *(a). IPAP-HSQMBC α (blue) and β (red) multiplets of the cross-peaks involving C3 (1.1 Hz) (b). 2D HSQMBC-IPAP spectrum (optimized to 2 Hz) after selective inversion of* ***H6b*** *(3.028 ppm) proton of meroditerpene* ***1b*** *(c). IPAP-HSQMBC α (blue) and β (red) multiplets of the cross-peaks involving C3 (1.7 Hz) and C11 (1.8 Hz) (d,e). Measurement of $^{n}J_{CH}$ values were performed by analysis of IPAP multiplet patterns (CD_2Cl_2, 700MHz). NS: 104. NUS parameters: 13%/512/17.* 20

Table S4. *^{13}C-^{1}H coupling constants for compound* ***1b****.* 21

Figure S19. *Detail of the 2D NOESY spectra of* ***1b*** *(a), selective NOE irradiation of H19 (b) and selective irradiation of H18 (c) of compound* ***1b****. (CD_2Cl_2, NS: 320, 800 MHz). Mixing time: 450 ms.* 22

Figure S20. *Detail of the 2D NOESY spectrum of* ***1b*** *(a). Observed NOESY contacts between H2 protons and H20, subjecting the existence of 2 conformations in the chromane fragment; strong NOE contact is indicated with a green dashed arrow, while weak NOE contact is indicating with a blue dashed arrow (b). In the conformation on the left H2a is far from H20 while in conformation on the right both protons have a similar distance to H20.* 22

Figure S21. *Equilibrium of the P-helicity (left conformation) and M-helicity (right conformation) conformers in the chromane fragment of* ***1b*** *and its impact on the J coupling value.* 23

Figure S22. *2D HSQMBC-IPAP spectrum of* ***1b*** *(optimized to 6 Hz) after selective inversion of* ***H2a*** *(1.859 ppm) proton of meroditerpene* ***1*** *(a). IPAP-HSQMBC α (blue) and β (red) multiplets of the cross-peaks involving C20 (3.6 Hz), C4 (1.7 Hz), C2' (4.1 Hz), C1(-3.7 Hz*) and C3 (3.4 Hz) to H2a (b-f). 2D HSQMBC-IPAP spectrum (optimized to 6 Hz) after selective inversion of* ***H2b*** *(1.805 ppm) proton of meroditerpene* ***1b*** *(g). IPAP-HSQMBC α (blue) and β (red) multiplets of the cross-peaks involving C20 (1.8 Hz), C4 (3.6 Hz), C2' (4.4 Hz), C1 (-3.8 Hz*) and C3 (2.8 Hz) to H2b (h-l). Measurement of $^{2,3}J_{CH}$ values were performed by analysis of IPAP multiplet patterns. NS: 48. NUS: 11%/640/35. *Sign determined from HSQC-HECADE.* 24

5. *J*-Based and NOE analysis around the rotatable bounds **25**

Figure S23. *Molecular structure of* ***1b****. Relevant rotatable bounds considered in the PC Model conformational search/J-based analysis/quantitative NOE are indicated in blue arrows.* 25

Figure S24. *Newman projection of rotamers of* ***1b*** *around ρ1 [H4b-C4-C3-C20]; computed $^{2}J_{C3H4}$ are indicated in dark red (a). 2D HSQMBC-IPAP spectrum (optimized to 6 Hz) after selective inversion of* ***H4b*** *(2.705 ppm) proton of meroditerpene* ***1b*** *(b). IPAP-HSQMBC α (blue) and β (red) multiplets of the cross-peaks involving H4b-C2 (2.1 Hz), H4b-C20 (0.2 Hz) and H4b-C3 (1.9 Hz) (c-e). 2D HSQMBC-IPAP spectrum (optimized to 6 Hz) after selective inversion of* ***H4a*** *(2.514 ppm) proton of meroditerpene* ***1b*** *(f). IPAP-HSQMBC α (blue) and β (red) multiplets of the cross-peaks involving H4a-C2 (2.0 Hz), H4a-C20 (5.6 Hz) and H4a-C3 (6.7 Hz) (g-i). Measurement of $^{2,3}J_{CH}$ values were performed by analysis of IPAP multiplet patterns. Detail of 2D NOESY spectrum showing the NOE contact between H4b and H20, and between H14a and H20 (j). NS: 72. NUS: 18%/640/57. $^{n}J_{CH}$ were computed at GIAO/OLYP/Def2TZV (gas phase) level.* 26

Figure S25. *Newman projection of rotamers around of* ***1b*** *ρ2 [H4b-C4-C5-C13] (a). Computed J-couplings of possible rotamers around ρ2 (level of theory GIAO/OLYP/Def2TZV) and experimental J-coupling of* ***1b*** *(b). IPAP-HSQMBC α (blue) and β (red) multiplets of the cross-peaks involving* ***H4b***

(2.705 ppm) to C13 (4.2 Hz), C6 (6.0 Hz) and C5 (5.0 Hz); and multiplets of the cross-peaks involving **H4a** (2.514 ppm) to C13 (5.6 Hz), C6 (3.6 Hz) and C5 (4.9 Hz). Measurement of $^{2,3}J_{CH}$ values were performed, as before, by analysis of IPAP multiplet patterns. (c-h). Selective 1D-NOESY spectrum of H4b showing the NOE contact between H14b (2.570 ppm), H2a, H2b, H20 and H17 (i). 3D model of 3S*,7R*,11S* showing NOE contact between H4b and H14b and between H4a and H6a (j). Detail of 2D NOESY spectrum of meroditerpene **1b** showing the interaction between H4a and H6a (k). 27

Figure S26. *Rotamer fragment around* ρ*3 [C12-C13-C14-C15] of merediterpene* **1b** *(a). Relevant experimental and computed (level of theory GIAO/OLYP/Def2TZV)* $^{2,3}J_{CH}$ *around* ρ*3 (b). Molecular model of compound* **1b***, showing NOE (indicated by a green dashed arrow) contacts between H14b and H4b, H20, H16 and H17, between H4a and H6a, and finally between H20 and H18 (c.1). Selective 1D NOESY spectrum of H14b showing the NOE contact between H4b and H16, H17, and H20 (c.2). Selective 1D NOESY spectrum of H18 showing the NOE contact with H20 (c.3). Selective 1D NOESY spectrum of H19 showing the NOE contact with H6a, H10a, H8a, and H17 (c.4). 2D HSQMBC-IPAP spectrum (optimized to 6 Hz) after selective inversion of* **H14b** *(2.570 ppm) proton of compound* **1b** *(d). IPAP-HSQMBC α (blue) and β (red) multiplets of the cross-peaks involving from left to right H14b to C12 (5.3 Hz), H14b to C5 (3.9 Hz), H14b to C13 (4.6 Hz),* **H14a** *to C12 (3.7 Hz) and finally H14a to C5 (5.2 Hz) (e-i). Measurement of* $^{2,3}J_{CH}$ *values were performed by analysis of IPAP multiplet patterns. NR: Not readable* 28

Figure S27. *Rotamer fragment around* ρ*4 [C13-C14-C15-C16] of meroditerpene* **1b** *(a-c). Experimental and computed (GIAO/OLYP/DEF2TZV)* $^{2,3}J_{CH}$ *around* ρ*4 (d). Selective 1D NOESY spectrum of H17 showing the NOE contact between H19, H14b, H14a, H4b and H16 (e.1). Correlations are indicated in a molecular model. H17 does not show NOE contact with the chromane system (e.2). Slice from a 2D NOESY spectrum showing NOE contacts from H16 to H1, H2a, H2b, H4b, H14a and H14b (e.3). Correlations are shown in a molecular model. H17 does not show NOE contact with the bicyclo[4.3.0]nonane moiety (e.4). IPAP-HSQMBC α (blue) and β (red) multiplets of the cross-peaks involving H14b (2.570 ppm) to C16 (1.3 Hz), H14b to C17 (2.4 Hz), H14a (2.506 ppm) to C16 (1.8 Hz), H14a to C17 (4.5 Hz), H14b to C15 (4.1 Hz) and H14a to C15 (5.9 Hz) (d-i). Measurement of* $^{2,3}J_{CH}$ *values were performed by analysis of IPAP multiplet patterns. NOE 1D experiment on selective inversion over OH-C15 (j).* 29

Figure S28. *NOE 1D experiment of* **1b** *on selective inversion over Me-C6' showing NOE contacts to H6b, H20, H8 and H19. (CD_2Cl_2, NS: 400, 800 MHz). Parameters: Mixing time: 450 ms.* 30

Figure S29. ^{1}*H 1D spectrum section of* **1b** *from 1 ppm until 1.3 ppm at 298.1 k (red line) and at 280 K (blue line) (a,b). Selective NOE experiment over H20 at 280 K (c), showing in the inset (green dashed box) NOE contacts between H20 and H4b, H14b and H4a (d). Both experiments were measured in a 950 MHz Bruker spectrometer (Pulprog: selnogp, NS: 40, mixing time: 500 ms).* 30

Figure S30*. J-modulated ADEQUATE spectrum of* **1b** *(adeq11etgpjcrdsp) (a). Parameters:* $^{1}J_{CH}$ *= 138 Hz,* $^{1}J_{CC}$ *= 40 Hz, Scaling factor = 8, relaxation delay = 1.5 s, NUS: 35%/512/89. (800 MHz/CD_2Cl_2)*........... 31

Figure S31. *Carbon-proton coupling constants from an IPAP-HSQMBC experiment and NOE contacts to relate C3, C7, and C11 stereogenic centers of (**3S*,7S*,11R*)-1b**).* $^{2}J_{CH}$ *sign was measured as absolute value.* 31

Table S5. *DP4 (a) and iJ-DP4 (b) Analysis of* **1b***.* **1***=***(3R7S11R)-1b***,* **2***=***(3S7S11R)-1b***.*............................ 32

6. Anisotropic measurements of 1b **41**

Figure S32. *Experimental arrangement needed for our in-house-made 3 mm compression device. Complete alignment system shown here includes: a common 3 mm NMR tube, swallowed PMMA (75/0.25) gel in $CDCl_3$, and the semi-micro compression apparatus (a). Fully relaxed swallowed gel stick ideally has a length of 4.5 cm length, while when it is fully compressed has a length of 3.5 cm. (b). Compression device consists of a clamping head/nut and fastening screws, which keep the plunger*

attached to the 3 mm glass NMR tube when the gel stick is under compression (c). Gel was orange colored using a pigment from Faber Castell. 41

Figure S33. $^{13}C\ \{^{1}H\}$ spectrum of estrone (800 µg) swollen in relaxed Poly-HEMA (80/0.27) in a 3 mm compression device. Number of scans 16K. (a). Under anisotropic conditions $\Delta^{2}H_{Q}$ was 4.6 Hz (b). (NS = 16K, 600 MHz, DMSO-d_6). 41

Figure S34. Chemical structure of estrone (a) and 13-epi-estrone (b). The quality of the data fitting is expressed as a quality factor (Q) where the correct structure is expected to have the lowest Q value. Results from the in-house 3 mm compression device: Q (blue) and Q_{CSA} (grey) factors calculated for lowest-energy structures of estrone and 13-epi-estrone from DFT calculation (GIAO/MPW1PW91/6-31+g(2d,p) iefpcm=DMSO) using only ^{13}C RCSAs. 42

Table S6. Estrone uncorrected residual chemical shift anisotropies data (Hz) analyzed in a 3 mm compression device. 42

Figure S35. $^{13}C\ \{^{1}H\}$ spectrum of **1b** swallow on PMMA-H_8 measured in a 950 MHz Bruker spectrometer (5 mm compression device) (a). Buried resonances of **1b** below polymer background signal: MeO-C4', C11, C7, C4 and C6 are shown. In blue, isotropic $^{13}C\ \{^{1}H\}$ spectrum of **1b** (2 mg sample in an 800 MHz Bruker spectrometer) and in red $^{13}C\ \{^{1}H\}$ spectrum of **1b** swallow in PMMA-H8 (4 mg) (b). NS: 2048. Relaxation delay: 2 s. 43

Figure 36. $^{13}C\ \{^{1}H\}$ spectrum of **1b** swallow on PMMA-d_8 measured in an 800 MHz Bruker spectrometer (in-house-made 3 mm compression device) (a). Former buried resonances of **1b** below polymer background are visible now: MeO-C4', C11, C7, C4 and C6. Isotropic contribution of some resonances is marked with an asterisk. Sample mass 2.2 mg. NS: 7200. Relaxation delay: 2 s. 43

Preparation of deuterated compression compatible PMMA gel (PMMA-d_8 gel) 44

Figure S37. 1D ^{2}H spectrum of CD_2Cl_2 in the sample of compound 1b, when PMMA-d_8 gel stick is fully relaxed (a). ^{2}H spectrum of CD_2Cl_2 on fully compressed PMMA-d_8 gel (b), and evolution of it through the time. Experimental time is expressed in hours (Exp. Time) (c-d). PMMA-d_8 yields a stable $\Delta^{2}H_{Q}$ (4.2 Hz) during at least 41 h. (Solvent CD_2Cl_2). 44

Figure S38 ^{13}C-RCSA analysis of meroditerpene **1b** swollen in 70/0.25 PMMA-d_8 gel (200 MHz, CD_2Cl_2) Fitting for carbon residual chemical shift anisotropies of diastereoisomers **(3R*,7S*,11R*)-1b** (a) and **(3S*,7S*,11R*)-1b** (b), when automatic isotropic shift correction is not applied. Q factors were 0.164 (a) and 0.358 (b). 44

Figure S39. Resample influence bar-plot for the Jackknife analysis of ^{13}C RCSA of compound **1b**. 45

Figure S40. Bootstrapping bell curves for ^{13}C RCSA analysis of **(3R*,7S*,11R*)-1b** and **(3S*,7S*,11R*)-1b** are indicated in green line and dashed blue line, respectively. Inspection of bell curves derived from Bootstrapping, made discrimination between diastereoisomers evident. (Sample size = 5000 points, distribution: Gaussian). 46

Figure S41. Bootstrapping bell curves for $^{1}D_{CH}$ analysis of **(3R*,7S*,11R*)-1b** and **(3S*,7S*,11R*)-1b** are indicated in green and dashed blue, respectively. (Sample size = 5000 points, distribution: Gaussian). 46

Figure S42. Bar plots for the CASE-3D analysis (AIC: black, χ^2: green) of both plausible configurations of compound **1b**. When conformer populations are fitted to its minimum by NMR isotropic data (a) and when populations populations were weighted by DFT energies at B3LYP/6-31G(d,p) level (b). Study was done on the same batch of conformers used in the J-DP4 analysis. The **(3R*,7S*,11R*)-1b** configuration scores better. 47

Figure S43. ^{1}H-1D experiment (a) and $^{13}C\ \{^{1}H\}$ spectrum (b) of **1b** recovered from the anisotropic sample (PMMA-d_8), NS was 16 and 800 respectively. No polymer resonances were observed in both $^{13}C\ \{^{1}H\}$ and ^{1}H 1D spectra of the recovered sample (b). Comparison between the $^{13}C\ \{^{1}H\}$ spectrum

PMMA-H8 (blue) and PMMA-d_8 (red) (d); it is evident the reduction in the polymer background signal when PMMA-d8 is used. Experiments were measured either at 800 or 950 MHz/CD_2Cl_2. .... *48*

***Table S7**. Uncorrected carbon residual chemical shift anisotropies data (Hz) for **1b**. C14 was taken as reference* *49*

7. Absolute configuration of 1b by TD-DFT ECD. **50**

***Figure 44.** Calculated (red line) and experimental (black line) ECD of the conformers of **(3R,7S,11R)-1b** (a) and **(3S,7R,11S)-1b** (c). Conformers and its population are displayed in panels b and d respectively. Conformers were weighted by NMR anisotropy. ECD was computed at level: HSE0/6-311G+(2d,p)/DGA1 (solvent model/parameters/n-states: IEFPCM/CH_2Cl_2/38)* *50*

***Figure 45.** Calculated (red line) and experimental (black line) ECD of the conformers of **(3R,7S,11R)-1b** (a) and **(3S,7R,11S)-1b** (c). Conformers and its population are displayed in panels b and d respectively. Conformers were weighted by NMR anisotropy. ECD was computed at level: PBE0/Def2TZV/W06 (solvent model/parameters/n-states: COSMO/CH_2Cl_2/50).* *51*

***Table S8.** Comparison of experimental and calculated $\alpha D25$ values for compound **1b** and both stereoisomers pairs. Optical rotation ($\alpha D25$) was computed at CAM-B3LYP/6-311++(2d,2p)/DGA1 IEPCM (CH_2Cl_2) level of theory.* *51*

8. DFT Calculations for (-)-α-santonin **52**

***Table S9.** Functional-basis set combinational used on (-)-α-santonin test systems.* *52*

***Table S10.** Computation time needed for various combinations of functional/basis set combinational. Test systems: (-)-α-santonin.* *53*

9. Molecular coordinates **54**

10. Z-matrices and CSA tensors **60**

11. Alignment tensors **95**

1. Isolation of merodіterpene 1b

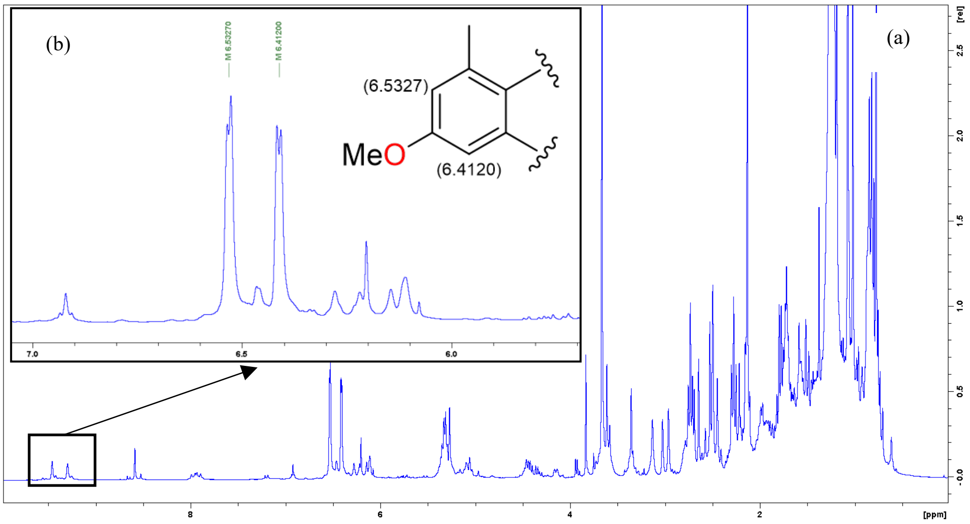

Figure S1. [1]H Spectrum (300 MHz, CD_2Cl_2) of fraction 19 (Fr. 19) from flash chromatography column (a), inset shows the characteristic doublet belonging to two meta-coupled aromatic protons from the meroditerpene skeleton (b) (300 MHz, CD_2Cl_2).

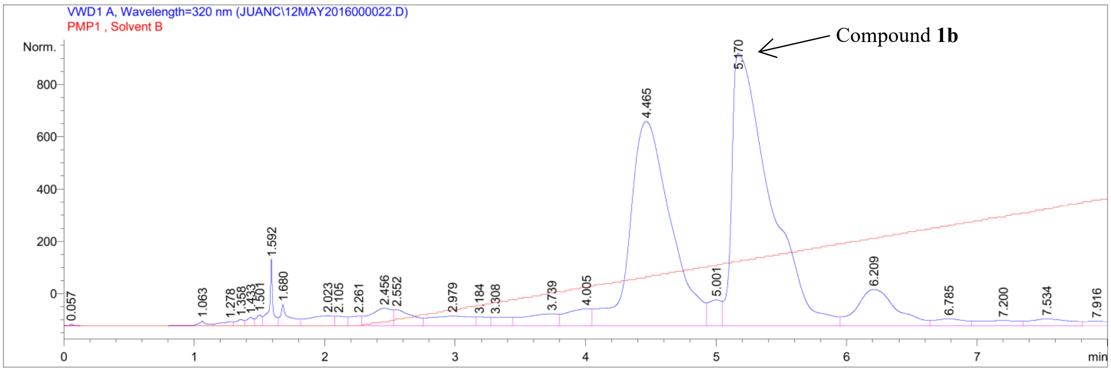

Figure S2. HPLC isolation of compound **1b** (5.17 min) from Fr. 19. Red line indicates solvent gradient.

2. Spectra of meroditerpene 1b

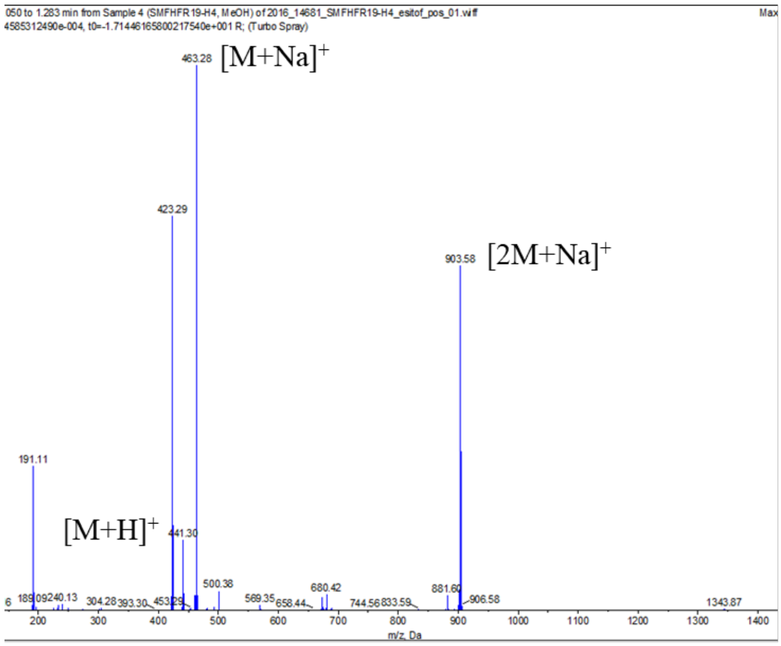

m/z (Da/e)	Centroid mass (Da)	Intensity (counts)	Centroid intensity	Intensity sum	Is Mono-isotopic
191.1069	191.1070	263.6667	333.4333	1197.0667	Yes
423.2874	423.2905	722.9333	1103.0889	3970.3333	Yes
441.2969	441.3012	129.3333	216.7778	742.6667	Yes
463.2811	463.2825	999.8667	1552.3333	5620.3333	Yes
903.5741	903.5758	632.4000	1116.6889	4100.1333	Yes

Figure S3. HRMS spectrum of compound **1b**.

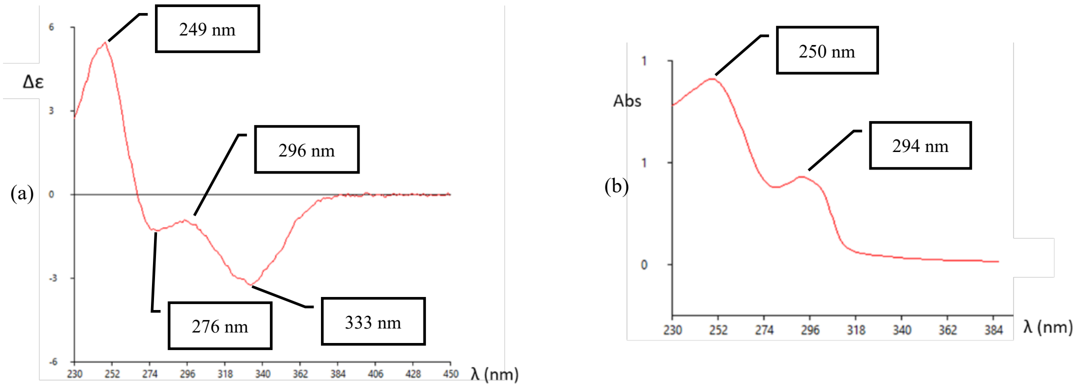

Figure S4. Experimental ECD (a) and UV (b) spectra of **1b** recorded in CH_2Cl_2 (*c* 0.2 mg/mL). Data collection parameters: data pitch of 1 nm, scanning speed of 20 nm/min, 5 accumulations, wavelength range was 224-470 nm and cell path length 1 mm. Curve smoothing algorithm used was a 3-wavelength center moving average with 4 iterations.

Note to the reader: Number of scans is indicated with the acronym NS. Experiments where non-uniform sampling uses the notation described in C. M. Thiele, W. Bermel, *Journal of Magnetic Resonance,* **2012**, 216:134-43.

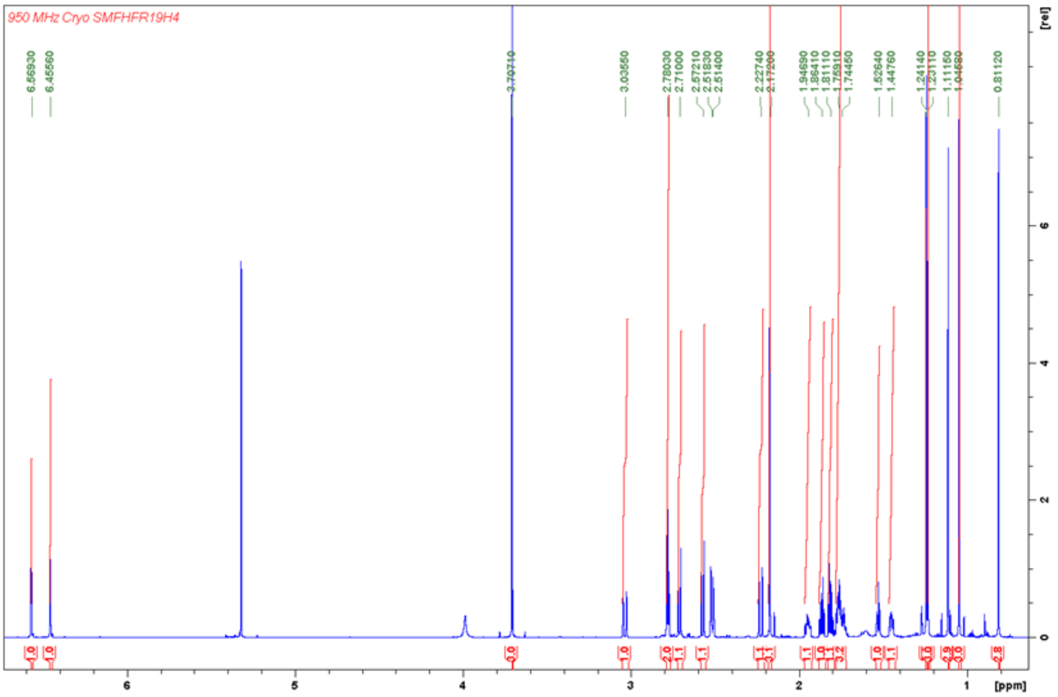

Figure S5. ^{1}H NMR spectrum of compound **1b** (CD_2Cl_2, NS: 16, 950 MHz).

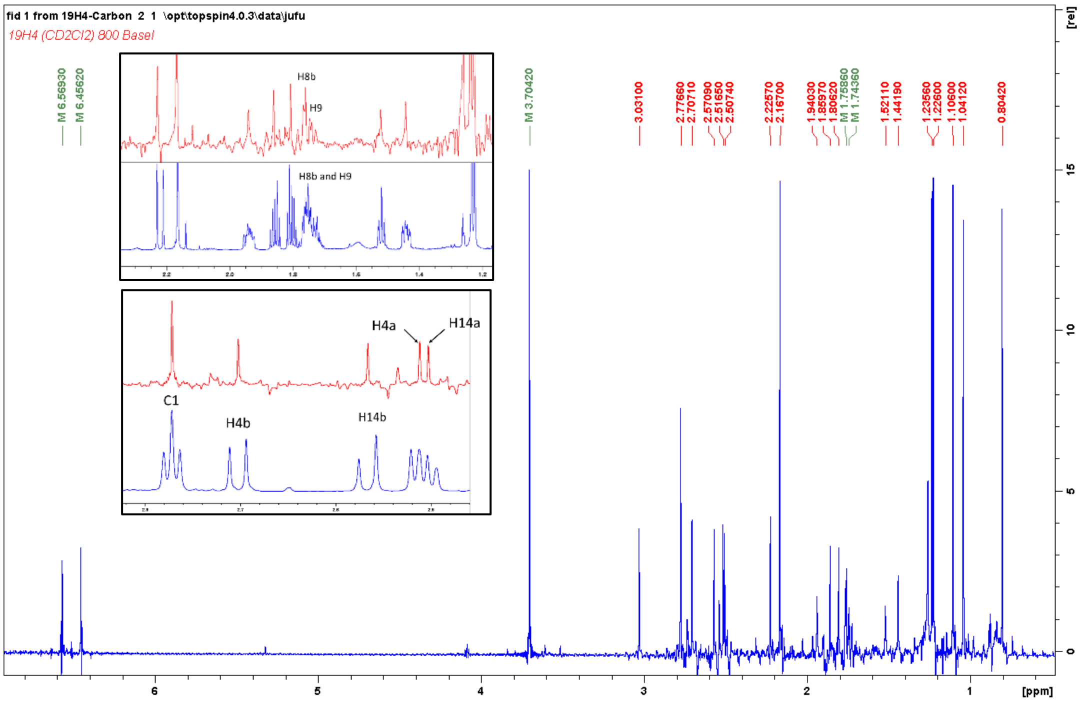

Figure S6. Pure shift ^{1}H NMR spectrum of compound **1b** (CD_2Cl_2, NS: 32, 800 MHz). Inset shows assignment of H8b, H9, H4b and H14a.

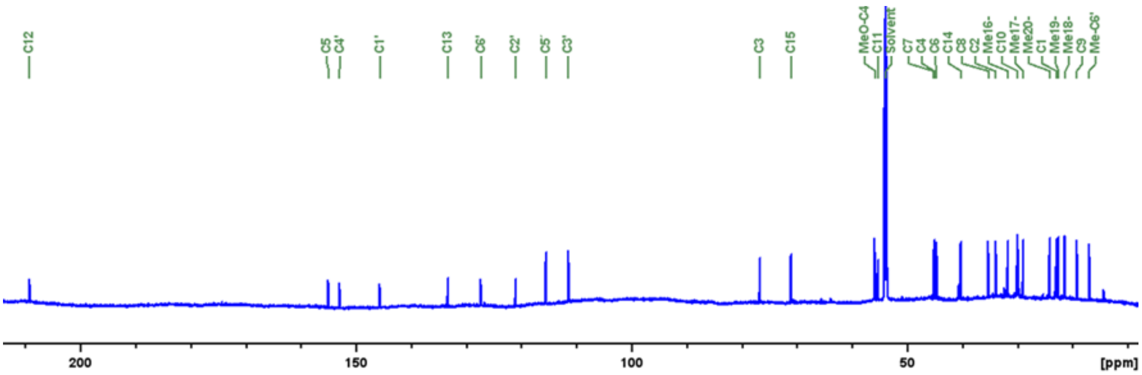

Figure S7. ^{13}C {^{1}H} NMR spectrum of compound **1b** (*zgdc30*, CD_2Cl_2, NS: 6 K, 200 MHz).

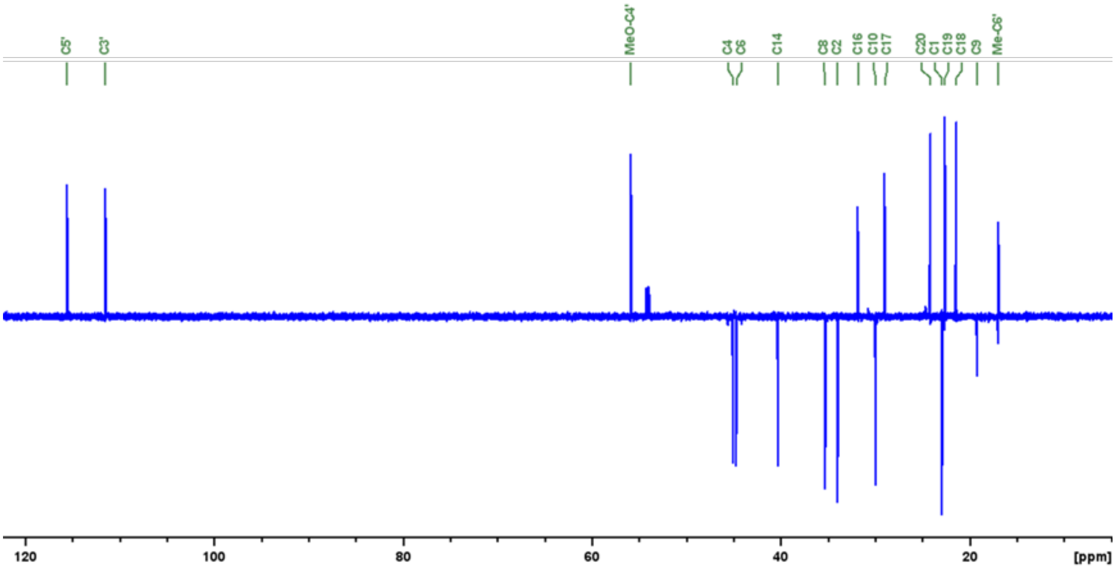

Figure S8. DEPT 135 spectrum of compound **1b** (*dept135*, CD_2Cl_2, NS 800, 200 MHz).

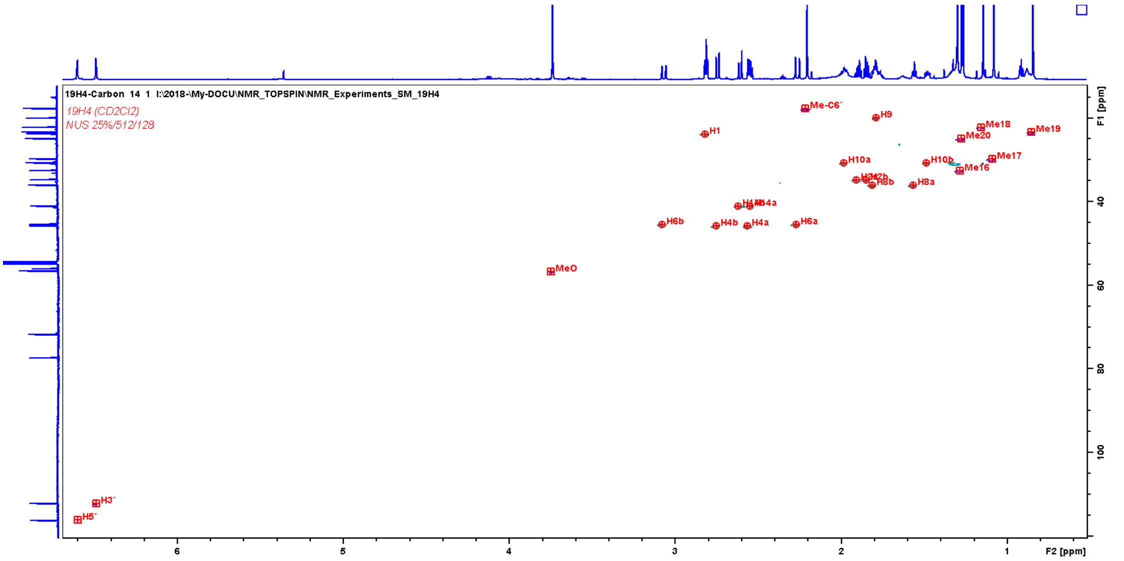

Figure S9. Pure shift-HSQC spectrum of compound **1b** (CD_2Cl_2, NS: 32, 800 MHz). Parameters: $^{1}J_{CH}$ = 130 Hz. (NUS: 25%/512/128). The spectrum on top is a normal ^{1}H spectrum.

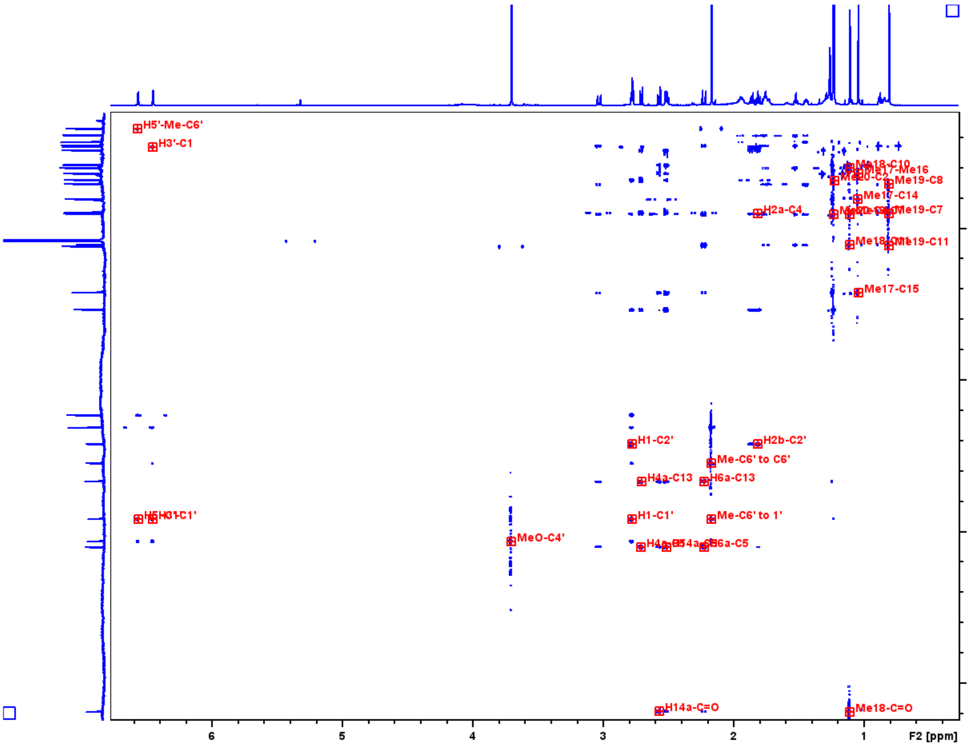

Figure S10. ^{13}C-^{1}H HMBC spectrum of compound **1b** (*hmbcetgpnd*, CD_2Cl_2, NS: 16, 800 MHz). Parameters: $^{n}J_{CH}$ = 8 Hz.

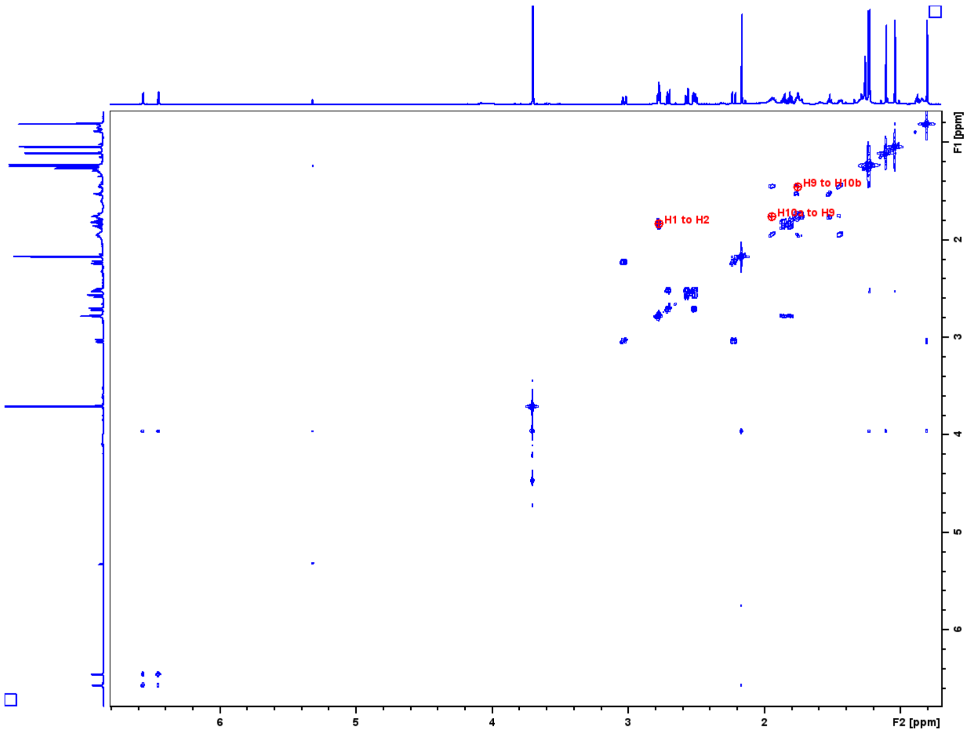

Figure S11. ^{1}H-^{1}H-DQFCOSY spectrum of compound **1b** (*cosyqf45*, CD_2Cl_2, 800 MHz).

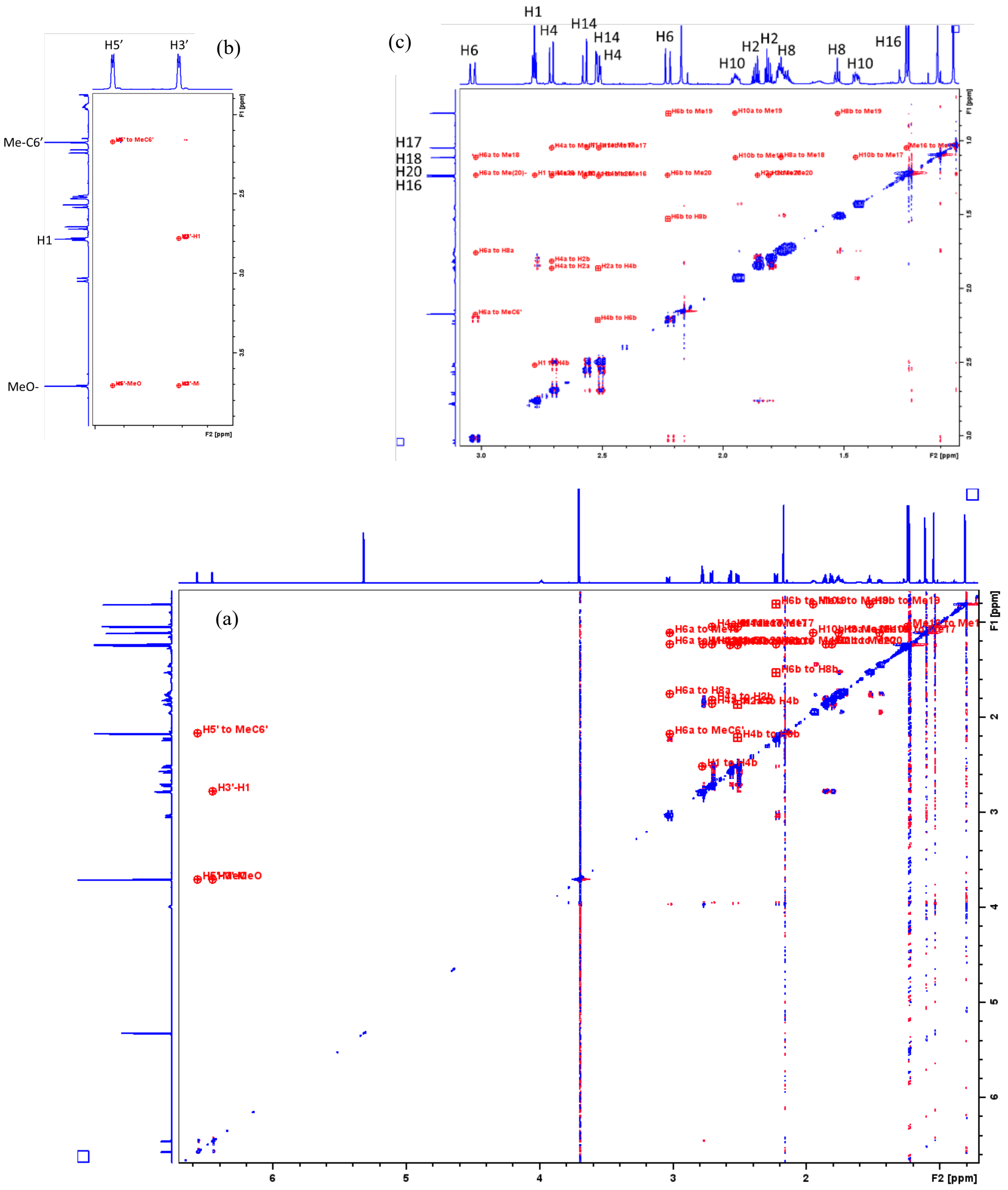

Figure S12. NOESY spectrum of compound **1b** (a). Relevant NOESY contacts are shown in insets b) and c). (*Noesyetgp*, CD_2Cl_2, 800 MHz, mixing time = 300 ms).

OH- attached to 15 was assigned by ^{13}C-^{1}H correlation taken from a low temperature HMBC experiment (265 K)

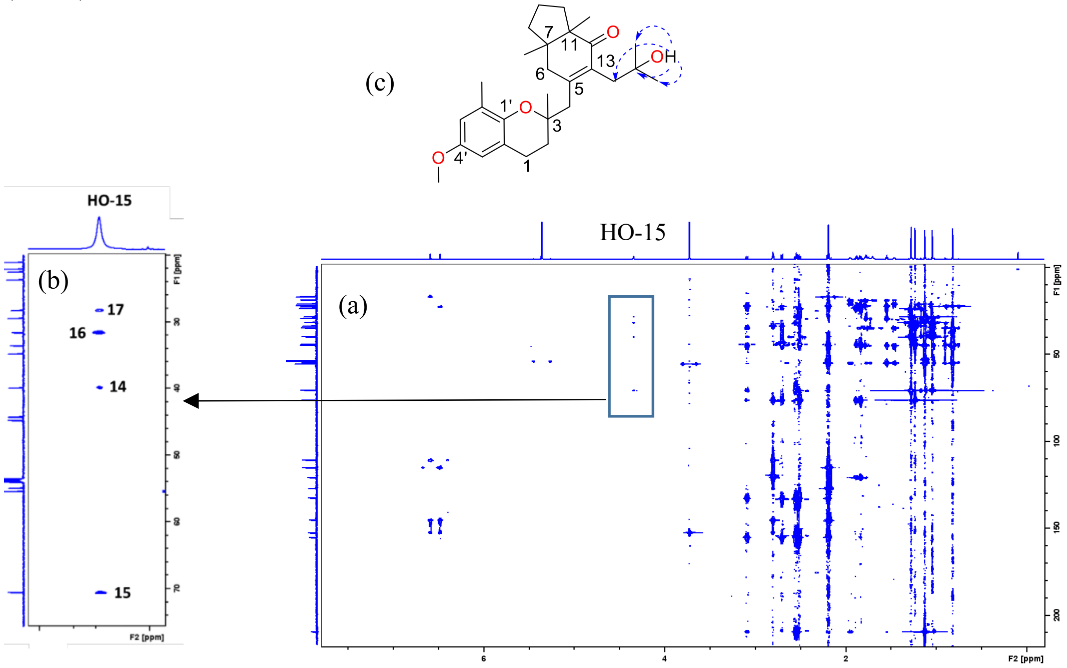

Figure S13. ^{13}C-^{1}H HMBC experiment of **1b** (a) recorded at 265 K (*hmbcetgpl3nd*, CD_2Cl_2, NS: 16, 950 MHz). (a) Inset shows several HMBC correlation from OH-15 (b). Correlations of **1b** are indicated by blue dashed lines (c) Parameters: $^{n}J_{CH}$ = 6 Hz. NUS: 20%/800/80

3. Benchmarking DFT methods for the calculation of $^{1}J_{CC}$, $^{2}J_{CH}$, $^{3}J_{CH}$

Molecular model of santonin and strychnine used were proposed before by NMR anisotropy and NOE analysis respectively. All the couplings were measured from either IPAP-HSQMBC or HSQC-HECADE experiments. This study focuses on comparing how well density functional theory (DFT) methods employing small basis sets can estimate absolute value of $^{2,3}J_{CH}$, and $^{3}J_{HH}$ in a rigid system.

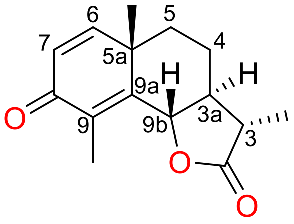

(-)-α-santonin

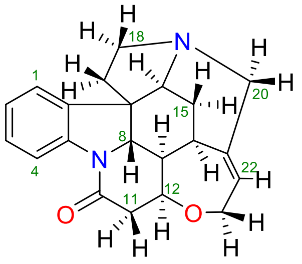

strychnine

Several methods were included for comparison. Altogether, 46 DFT methods were tested using one data set which the *J*-coupling have been determined by isotropic NMR methods in DMSO-d_6. The OLYP basis set performed far better than other functional basis set combinations. Due to its short computational demands, it is suitable for most of the common systems.

Table S1. Experimental spin-spin carbon proton long range couplings ($^{2,3}J_{CH}$) of minus-alpha-santonin and strychnine in isotropic solution (DMSO-d_6) ^{ɣ}

(-)-α-Santonin			(-)-α-Santonin		
Correlation		$^2J_{CH}$ Exp. (Hz)	Correlation		$^3J_{CH}$ Exp. (Hz)
C8	H7*	0.8	C9a	H3a	2.4*
C5a	H6*	3.0	C9	H7	3.5*
C9a	H9b	3.5	C5a	H7	7.9*
C5a	H5a*	3.5	C8	H6	9.9*
C5a	H5b*	3.5	C9a	H6	6.6*
C5a	Me*	3.7	C9	H9b	4.3*
C9	Me*	6.4	C5a	H4b	1.6*
C9b	H3a	-5.7	C5a	H4a	8.5*
C3a	H9b	-3.3	C9a	Me-	3.2*
C5	H4b	-4.3			
C5	H4a	-3.6	C8	Me-C9	3.7*
C4	H5b	-3.0	C5	H6	2.9*
C3a	H3	-4.7	C4	H9b	2.4
C3	H3a	-2.9	C9b	H4b	2.6
C2	H3	7.4	C9b	H4a	11.0
C3	Me	-4.9	C6	H5a	3.0*
Me	H3	-4.3	C3a	H5a	1.9
			C9b	H3	0.7
			C4	H3	3.7
			C6	Me-	4.7*
			C5	Me-	4.5*
			C3a	Me-C3	4.6*
			C3	H9b	2.2*
			C3	H4b	2.0
			MeC3	H3a	4.1
			MeC5a	H6	2.5*
			MeC5a	H5a	7.8*
			MeC5a	H5b	3.4*

Strychnine		
Atoms		$^2J_{CH}$ Exp. (Hz)
C11	H12*	2.1
C8	H13*	6.4
C14	H13*	4.9

^{ɣ} Spin-spin couplings were extracted either from HECADE-HSQC or IPAP-HSQMBC spectrum. (*) Unsigned coupling measured from IPAP-HSQMBC.

Table S2. Derived fitting parameters for the spin-spin carbon proton long range couplings ($^{2,3}J_{CH}$) in isotropic solution (DMSO-d_6). Fitting done over unsinged values

Level of theory: GIAO: OLYP/DefT2TZV				
Spin-spin coupling	*Slope*	*Coefficient correlation*	*Standard deviation*	*Number of Spin-spin coupling fitted*
$^{2}J_{CH}$	0.846	0.967	1.5	20
$^{3}J_{CH}$	0.951	0.978	2.3	32
Level of theory: GIAO: MPW1PW91/6-311+G(2d,p)				
Spin-spin coupling	*Slope*	*Coefficient correlation*	*Standard deviation*	*Number of Spin-spin coupling fitted*
$^{2}J_{CH}$	0.9834	0.992	1.4	20
$^{3}J_{CH}$	0.9139	0.993	2.4	32

Chemical shielding tensors and spin-spin coupling for ^{13}C RCSA/*J*-based analysis was computed at GIAO/MPW1PW91/6-311G+(2d,p) in gas phase. Spin-spin coupling for the *J*-based analysis in the bicyclo[4.3.0]nonane moiety were computed with the combination GIAO/OLYP/Def2TZV in gas phase. ^{n}J-Couplings correction factor is shown in Table S3.

Table S3. Scaling factor correction for $^{n}J_{XH}$ (X=H, C) computed at MPW1PW91/6-311+G(2d,p) (gas phase) level of theory used in the *J*-based analysis of **1b**.

$^{3}J_{CH}$	$^{2}J_{CH}$	$^{3}J_{HH}$
0.9139	0.9834	0.8824

Note: Spin-spin coupling was computed to such a low level of theory (OLYP/Def2TZV) due number of molecular models considered in the ensemble and the relatively cheap computational cost of it. Whenever possible, we recommend considering other options as GIAO/MPW1PW91/6-311G+(2d,p).

To the reader: Spin-spin coupling were computed as “Total nuclear spin-spin coupling” considering the four terms contributing to the Nuclear Spin-spin Coupling Constants: the paramagnetic and diamagnetic orbital terms, and the electron-spin dependent Fermi-contact and spin-dipole term.

4. *J*-Based Configurational Analysis and NOE Measurements

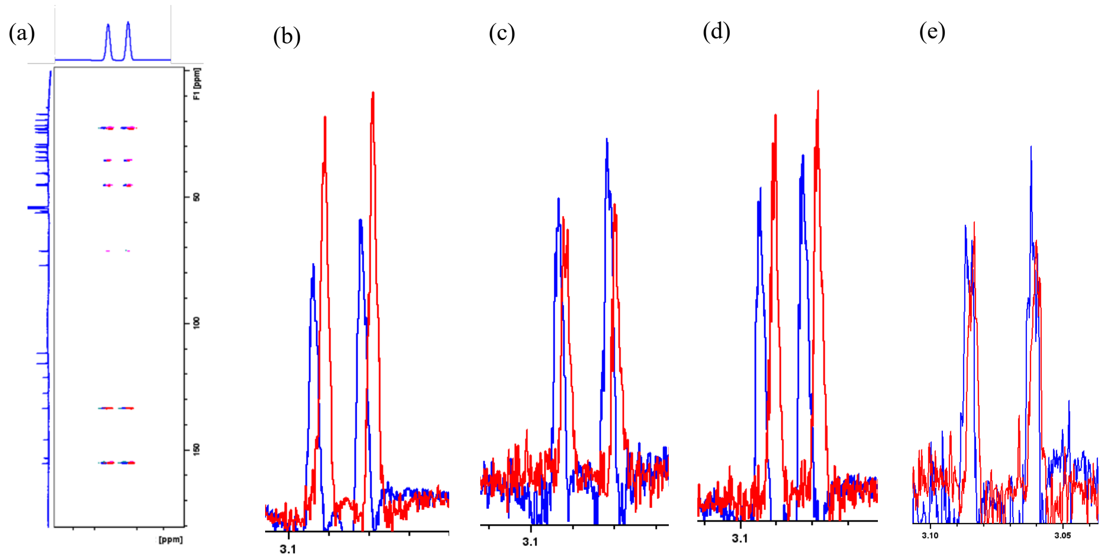

Figure S14. 2D HSQMBC-IPAP spectrum (optimized to 6 Hz) after selective inversion of **H6b** (3.028 ppm) proton of meroditerpene **1b** (a). IPAP-HSQMBC α (blue) and β (red) multiplets of the cross-peaks involving C13 (6.0 Hz), C8 (2.4 Hz), C19 (6.4 Hz) and C15 (1.8 Hz) (b-e). Measurement of $^{3}J_{CH}$ and $^{5}J_{CH}$ values were performed by analysis of IPAP multiplet patterns. NS: 32. NUS: 30%/640/96.

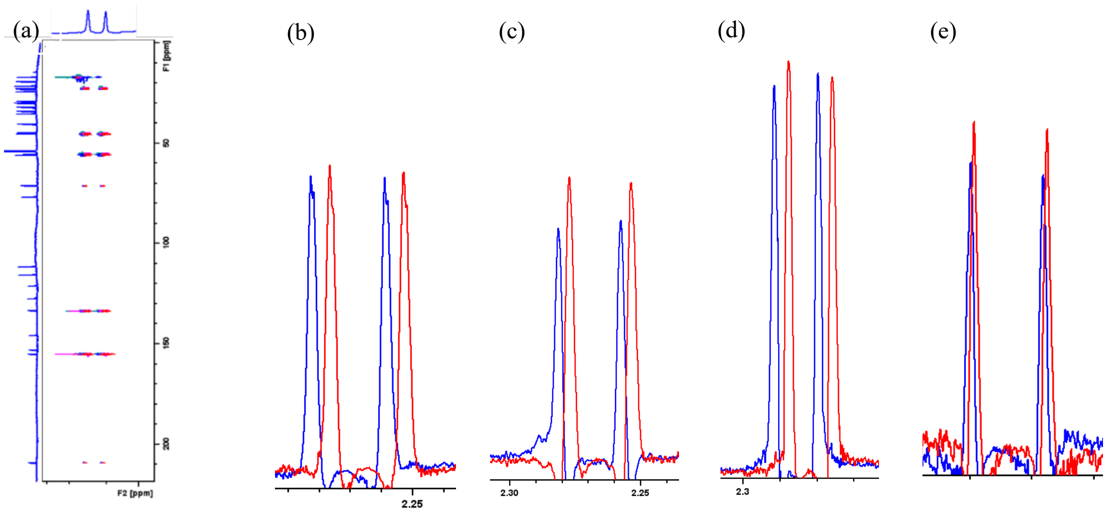

Figure S15. 2D HSQMBC-IPAP spectrum (optimized to 6 Hz) after selective inversion of **H6a** (2.237 ppm) proton of meroditerpene **1b** (a). IPAP-HSQMBC α (blue) and β (red) multiplets of the cross-peaks involving C13 (5.2 Hz), C19 (3.4 Hz), C11 (6.2 Hz) and C15 (1.4 Hz) (b-e). Measurement of $^{3}J_{CH}$ and $^{5}J_{CH}$ values were performed by analysis of IPAP multiplet patterns. NS:32. NUS: 33%/640/104.

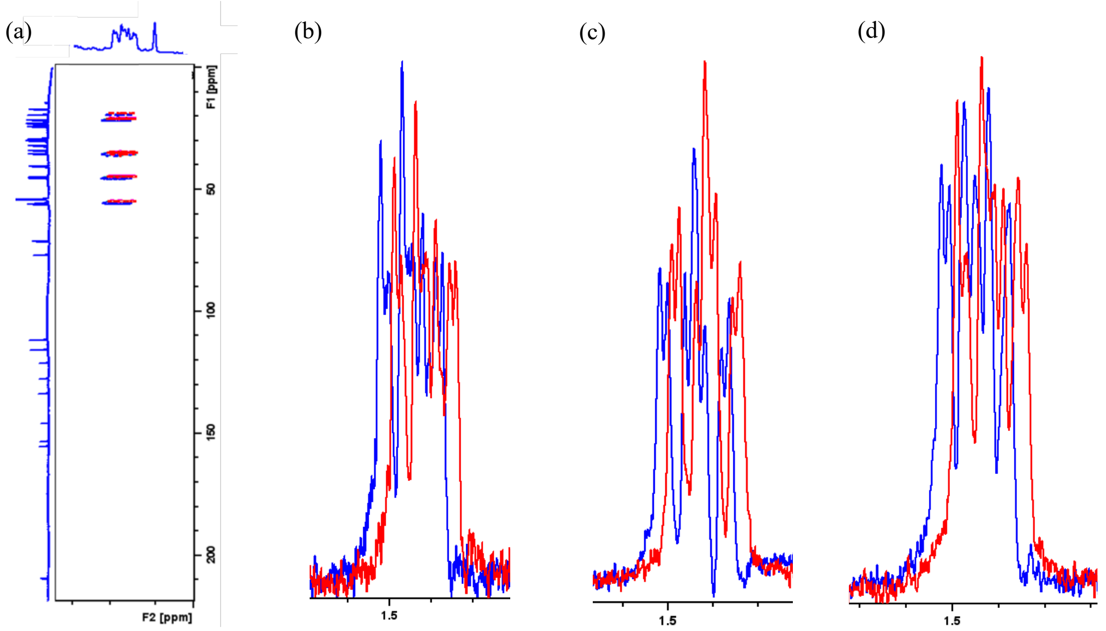

Figure S16. 2D HSQMBC-IPAP spectrum (optimized to 6 Hz) after selective inversion of **H10b** (1.441 ppm) proton of meroditerpene **1b** (a). IPAP-HSQMBC α (blue) and β (red) multiplets of the cross-peaks involving C18 (5.2 Hz), C8 (4.1 Hz) and C7 (5.5 Hz) (b-d). Measurement of $^{3}J_{CH}$ values were performed by analysis of IPAP multiplet patterns. NS: 32. NUS: 23%/640/73.

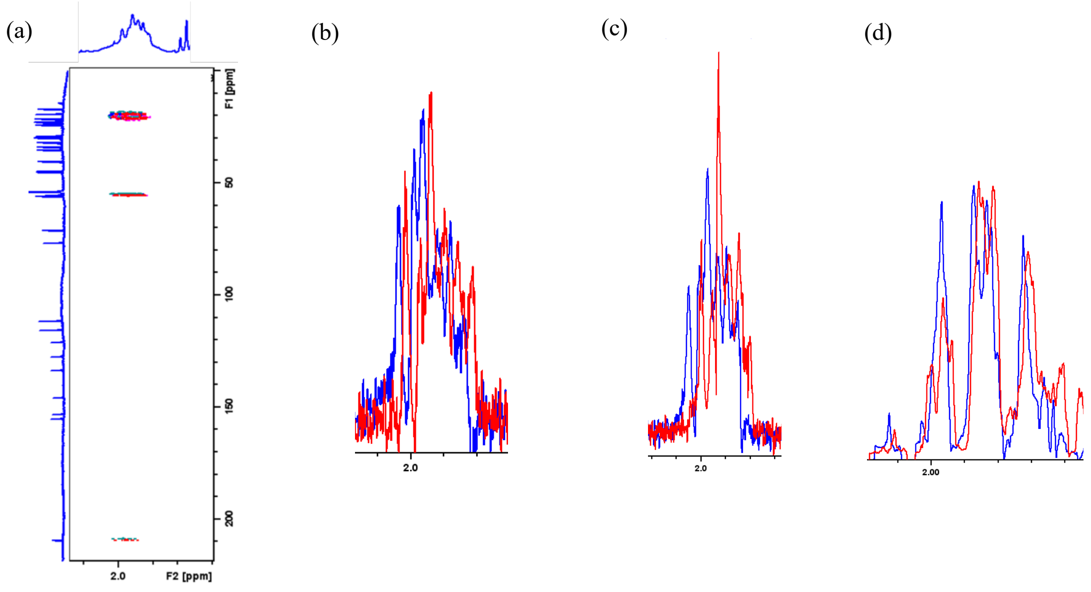

Figure S17. 2D HSQMBC-IPAP spectrum (optimized to 6 Hz) after selective inversion of **H10a** (1.944 ppm) proton of meroditerpene **1b** (a). IPAP-HSQMBC α (blue) and β (red) multiplets of the cross-peaks involving the C12 (3.8 Hz), C18 (8.2 Hz) and C8 (1.0 Hz) (b-c). Measurement of $^{n}J_{CH}$ values were performed by analysis of IPAP multiplet patterns. $^{3}J_{C8H10a}$ was extracted from an HSQMBC-IPAP spectrum optimized to 1.5 Hz (d). NS: 32. NUS: 23%/640/73.

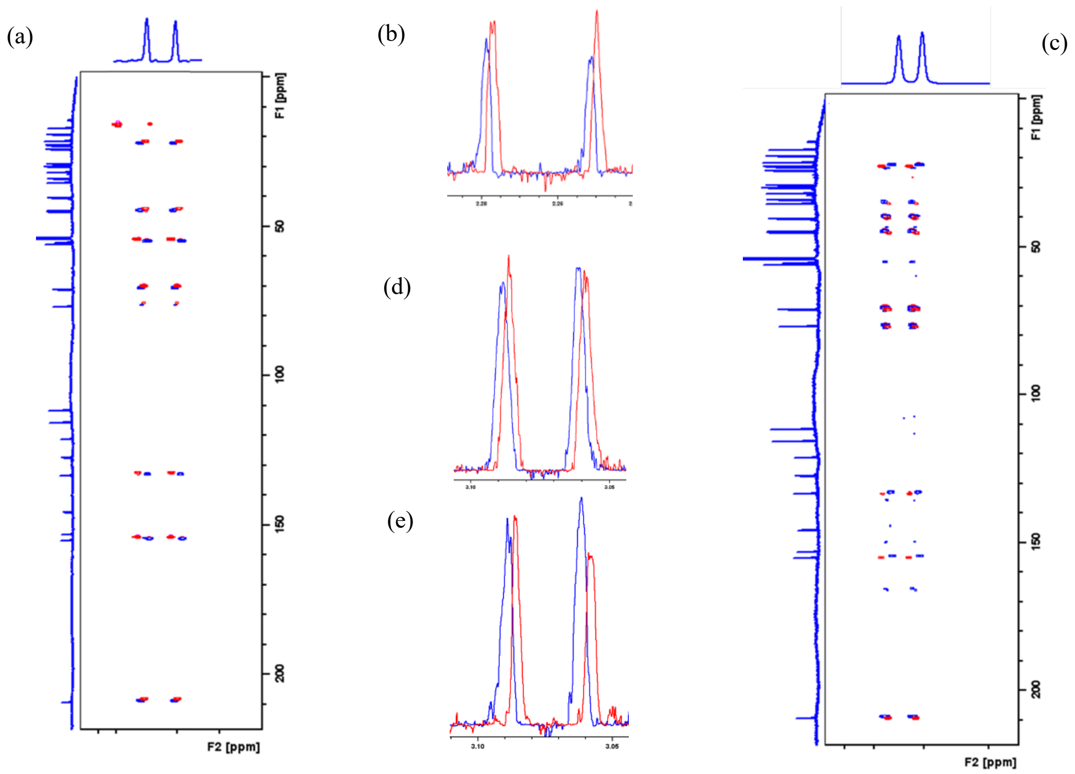

Figure S18. 2D HSQMBC-IPAP spectrum (optimized to 2 Hz) after selective inversion of **H6a** (2.237 ppm) proton of merodíterpene **1b** (a). IPAP-HSQMBC α (blue) and β (red) multiplets of the cross-peaks involving C3 (1.1 Hz) (b). 2D HSQMBC-IPAP spectrum (optimized to 2 Hz) after selective inversion of **H6b** (3.028 ppm) proton of meroditerpene **1b** (c). IPAP-HSQMBC α (blue) and β (red) multiplets of the cross-peaks involving C3 (1.7 Hz) and C11 (1.8 Hz) (d,e). Measurement of $^{n}J_{CH}$ values were performed by analysis of IPAP multiplet patterns (CD_2Cl_2, 700MHz). NS: 104. NUS parameters: 13%/512/17.

Table S4. ^{13}C-^{1}H coupling constants for compound **1b**.

IPAP-HSQMBC Correlation	Spin-Spin Coupling value (Hz)	IPAP-HSQMBC Correlation	Spin-Spin Coupling value (Hz)
$^{3}J_{C8H6b}$	2.4	$^{3}J_{C2H4b}$	2.1
$^{3}J_{C19H6a}$	3.4	$^{3}J_{C20H4b}$	0.2
$^{3}J_{C11H6a}$	6.2	$^{2}J_{C3H4b}$	1.9
$^{3}J_{C19H6b}$	6.4	$^{2}J_{C5H4b}$	5.0
$^{3}J_{C18H10b}$	5.2	$^{3}J_{C13H4b}$	4.2
$^{3}J_{C7H10b}$	5.5	$^{3}J_{C6H4b}$	6.0
$^{3}J_{C8H10b}$	4.1	$^{2}J_{C5H4a}$	4.9
$^{3}J_{C12H10a}$	3.8	$^{3}J_{C2H4a}$	2.0
$^{3}J_{C18H10a}$	8.2	$^{3}J_{C20H4a}$	5.6
		$^{3}J_{C13H4a}$	5.6
$^{3}J_{C4H2b}$	3.6	$^{3}J_{C6H4a}$	3.6
$^{3}J_{C2'H2b}$	4.4	$^{2}J_{C3H4a}$	6.7
$^{3}J_{C20H2b}$	1.8		
$^{2}J_{C1H2b}$	-3.8 [2]	$^{3}J_{C12H14b}$	5.3
$^{2}J_{C3H2b}$	2.8	$^{3}J_{C5H14b}$	3.9
		$^{2}J_{C13H14b}$	4.6
$^{3}J_{C4H2a}$	1.7	$^{3}J_{C16H14b}$	1.3
$^{3}J_{C2'H2a}$	4.1	$^{3}J_{C17H14b}$	2.4
$^{3}J_{C20H2a}$	3.6	$^{2}J_{C15H14b}$	4.1
$^{2}J_{C1H2a}$	-3.7 [2]		
$^{2}J_{C3H2a}$	3.4	$^{3}J_{C16H14a}$	1.8
		$^{3}J_{C12H14a}$	3.7
$^{4}J_{C3H6a}$	1.1	$^{3}J_{C5H14a}$	5.2
$^{4}J_{C3H6b}$	1.7		
$^{3}J_{C13H6b}$	6.0	$^{2}J_{C15H4a}$	5.9
$^{5}J_{C15H6b}$	1.8	$^{3}J_{C17H14a}$	4.5
$^{3}J_{C13H6a}$	5.2		
$^{5}J_{C15H6a}$	1.4		
$^{3}J_{C3H1}$	4.4 [1]		
$^{3}J_{C8H6a}$	NR		
$^{3}J_{C11H6b}$	1.8		
$^{3}J_{C7H10a}$	NR		
$^{3}J_{C8H10a}$	1.0		
$^{3}J_{C12H10b}$	NR		

NR not readble

(1) Measured from a *J*-HMBC experiment.

(2) Sign determined from a HSQC-HECADE experiment, which does not work for couplings to quaternary carbons.

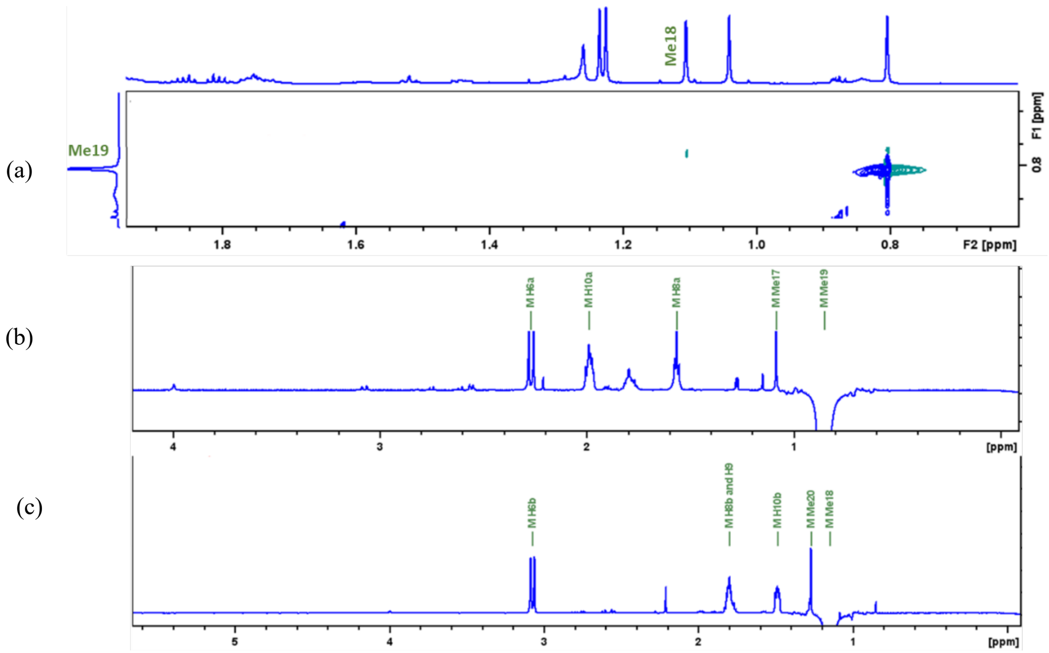

Figure S19. Detail of the 2D NOESY spectra of **1b** (a), selective NOE irradiation of H19 (b) and selective irradiation of H18 (c) of compound **1b**. (CD_2Cl_2, NS: 320, 800 MHz). Mixing time: 450 ms.

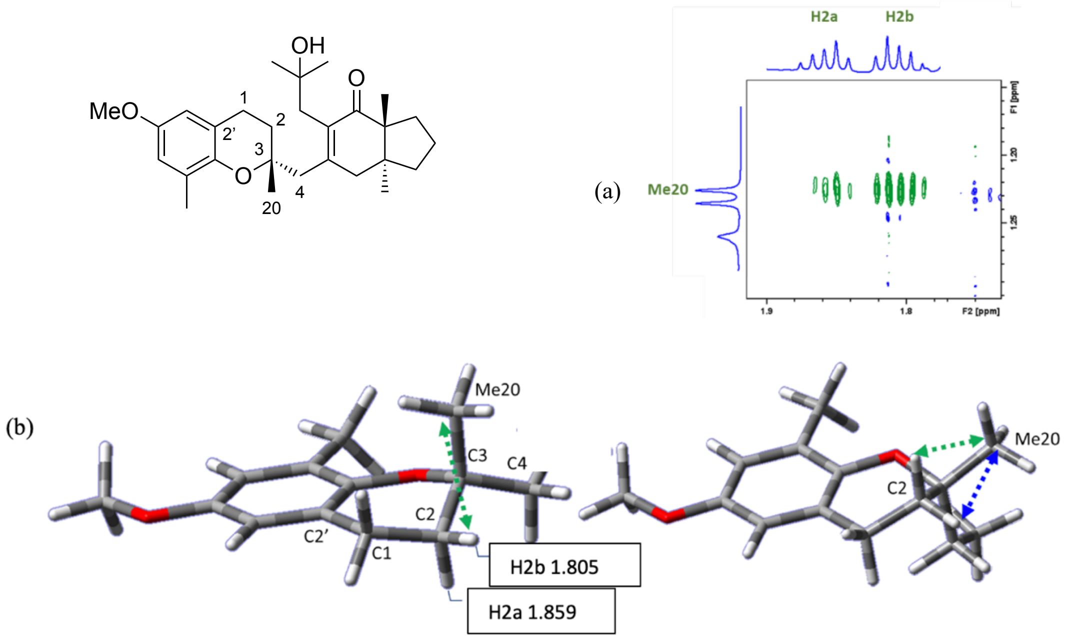

Figure S20. Detail of the 2D NOESY spectrum of **1b** (a). Observed NOESY contacts between H2 protons and H20, subjecting the existence of 2 conformations in the chromane fragment; strong NOE contact is indicated with a green dashed arrow, while weak NOE contact is indicating with a blue dashed arrow (b). In the conformation on the left H2a is far from H20 while in conformation on the right both protons have a similar distance to H20.

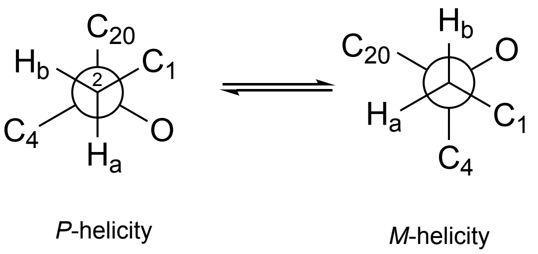

Atoms	Spin-Spin coupling Exp. (Hz)	Spin-Spin coupling Calc. *P*-helicity (Hz)	Spin-Spin coupling Calc. *M*-helicity (Hz)
$^3J_{C4H2b}$	3.6	1.2	5.8
$^3J_{C2'H2b}$	4.4	7.2	1.0
$^3J_{C20H2b}$	1.8	1.2	3.8
$^2J_{C1H2b}$	-3.8	-3.4	-3.3
$^2J_{C3H2b}$	2.8	-1.0	-5.5
$^3J_{C4H2a}$	1.7	3.8	1.4
$^3J_{C2'H2a}$	4.1	1.0	7.2
$^3J_{C20H2a}$	3.6	5.7	1.0
$^2J_{C1H2a}$	-3.7	-3.3	-3.3
$^2J_{C3H2a}$	3.4	-5.5	-1.0

Computed at OLYP/Def2TZV/in gas phase DFT level. Experimental couplings were determined as absolute values. $^2J_{C1H2}$ sign was determined from an HSQC-HECADE experiment.

Figure S21. Equilibrium of the *P*-helicity (left conformation) and *M*-helicity (right conformation) conformers in the chromane fragment of **1b** and its impact on the *J* coupling value.

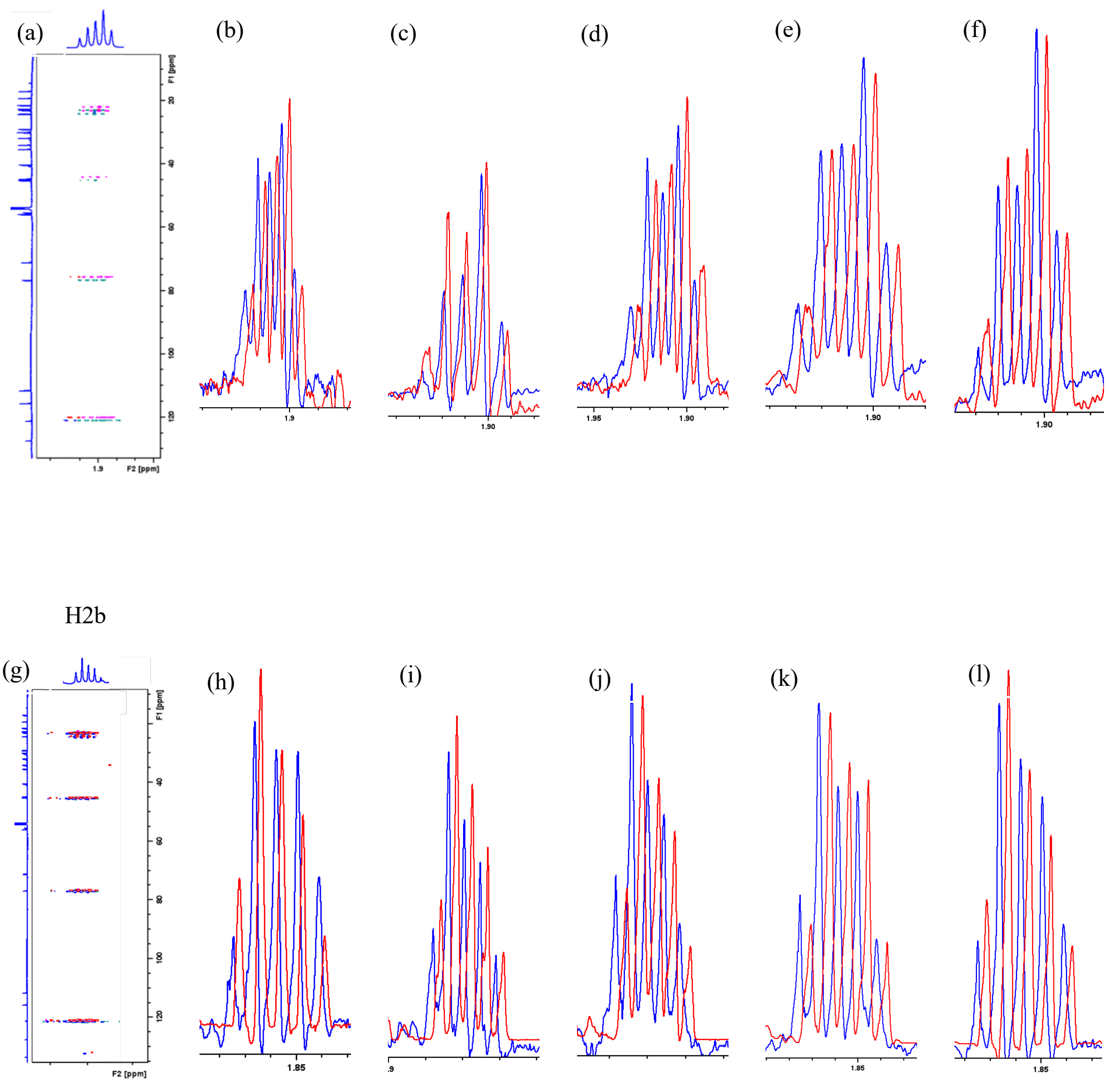

Figure S22. 2D HSQMBC-IPAP spectrum of **1b** (optimized to 6 Hz) after selective inversion of **H2a** (1.859 ppm) proton of meroditerpene **1** (a). IPAP-HSQMBC α (blue) and β (red) multiplets of the cross-peaks involving C20 (3.6 Hz), C4 (1.7 Hz), C2' (4.1 Hz), C1(-3.7 Hz*) and C3 (3.4 Hz) to H2a (b-f). 2D HSQMBC-IPAP spectrum (optimized to 6 Hz) after selective inversion of **H2b** (1.805 ppm) proton of meroditerpene **1b** (g). IPAP-HSQMBC α (blue) and β (red) multiplets of the cross-peaks involving C20 (1.8 Hz), C4 (3.6 Hz), C2' (4.4 Hz), C1 (-3.8 Hz*) and C3 (2.8 Hz) to H2b (h-l). Measurement of $^{2,3}J_{CH}$ values were performed by analysis of IPAP multiplet patterns. NS: 48. NUS: 11%/640/35. *Sign determined from HSQC-HECADE.

5. *J*-Based and NOE analysis around the rotatable bounds

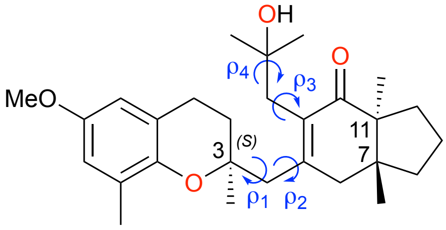

ρ1 [H4b-C4-C3-C20]
ρ2 [H4b-C4-C5-C13]
ρ3 [C12-C13-C14-C15]
ρ4 [C13-C14-C15-C16]

Figure S23. Molecular structure of **1b**. Relevant rotatable bounds considered in the PC Model conformational search/*J*-based analysis/quantitative NOE are indicated in blue arrows.

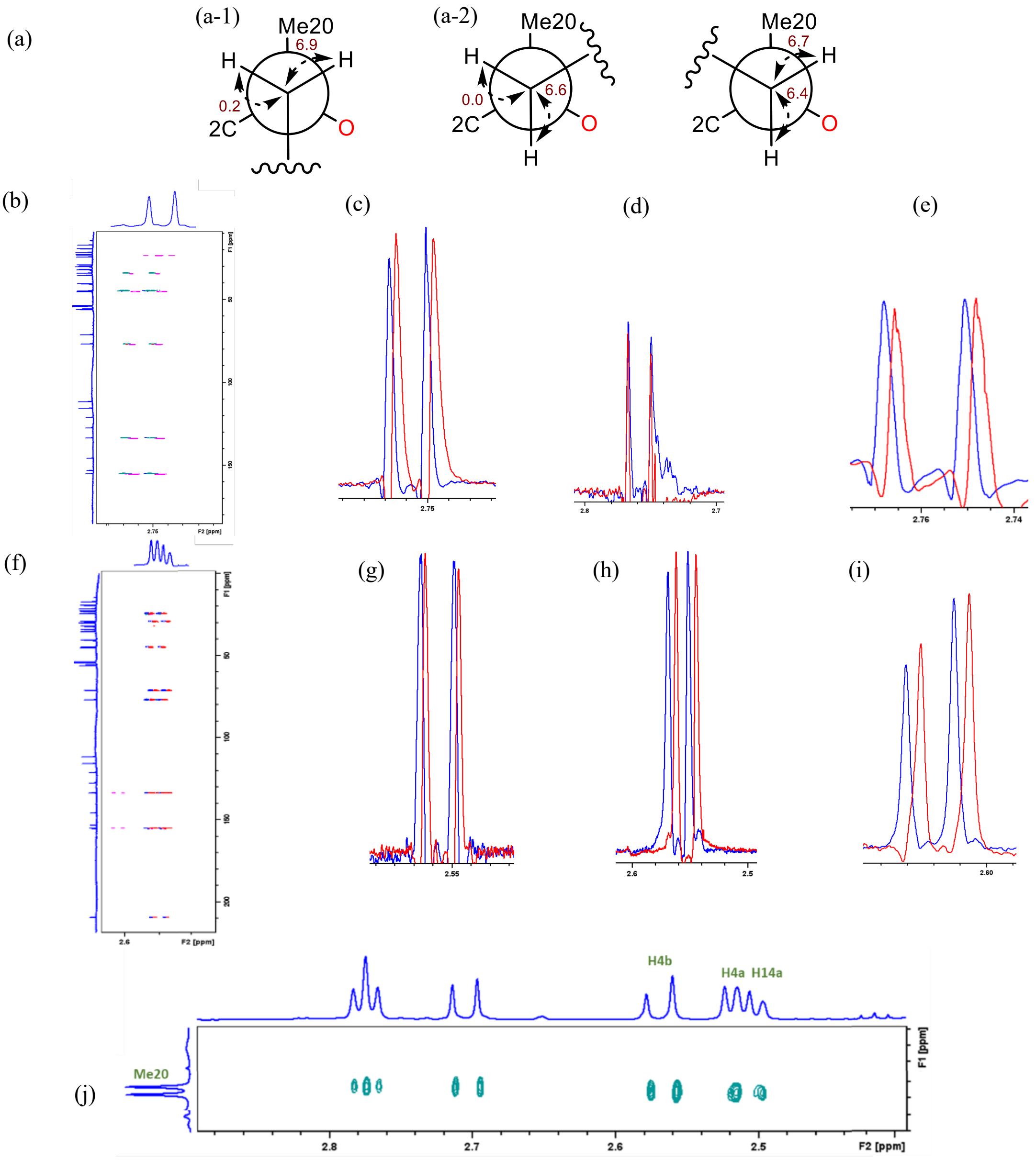

Figure S24. Newman projection of rotamers of **1b** around ρ1 [H4b-C4-C3-C20]; computed $^{2}J_{C3H4}$ are indicated in dark red (a). 2D HSQMBC-IPAP spectrum (optimized to 6 Hz) after selective inversion of **H4b** (2.705 ppm) proton of meroditerpene **1b** (b). IPAP-HSQMBC α (blue) and β (red) multiplets of the cross-peaks involving H4b-C2 (2.1 Hz), H4b-C20 (0.2 Hz) and H4b-C3 (1.9 Hz) (c-e). 2D HSQMBC-IPAP spectrum (optimized to 6 Hz) after selective inversion of **H4a** (2.514 ppm) proton of meroditerpene **1b** (f). IPAP-HSQMBC α (blue) and β (red) multiplets of the cross-peaks involving H4a-C2 (2.0 Hz), H4a-C20 (5.6 Hz) and H4a-C3 (6.7 Hz) (g-i). Measurement of $^{2,3}J_{CH}$ values were performed by analysis of IPAP multiplet patterns. Detail of 2D NOESY spectrum showing the NOE contact between H4b and H20, and between H14a and H20 (j). NS: 72. NUS: 18%/640/57. $^{n}J_{CH}$ were computed at GIAO/OLYP/Def2TZV (gas phase) level.

(a)

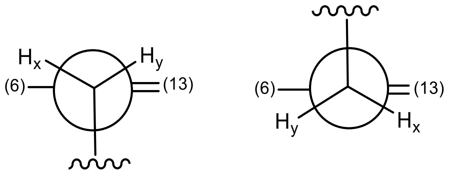

Experimental *J*-couplings

(b)

Carbon	Hx	Hy	Hx	Hy	H4a (2.514 ppm)	H4b (2.705 ppm)
C13	5.5	3.7	3.8	5.4	5.6	4.2
C6	4.9	5.4	5.6	4.9	3.6	6.0
C5	6.1	5.0	5.2	5.9	4.9	5.0

(c)

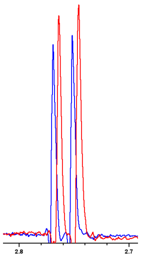

(d)

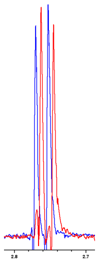

(e)

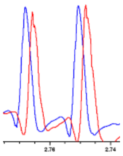

(f)

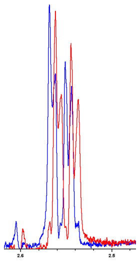

(g)

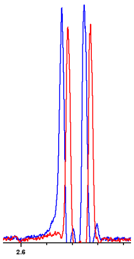

(h)

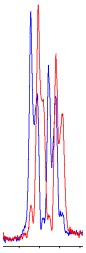

(i)

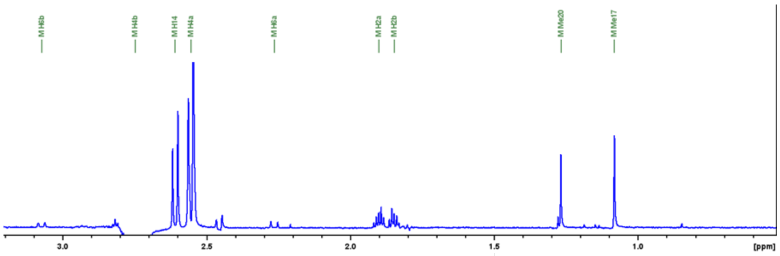

(j)

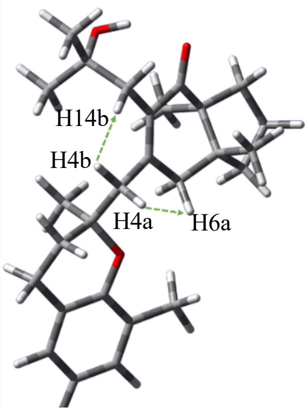

(k)

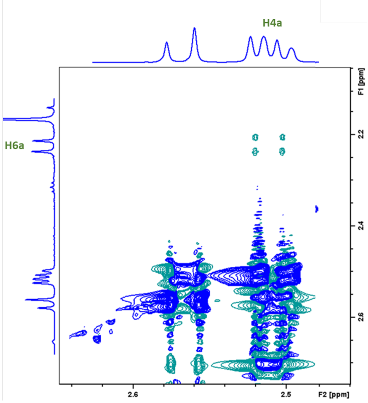

Figure S25. Newman projection of rotamers around of **1b** ρ2 [H4b-C4-C5-C13] (a). Computed *J*-couplings of possible rotamers around ρ2 (level of theory GIAO/OLYP/Def2TZV) and experimental *J*-coupling of **1b** (b). IPAP-HSQMBC α (blue) and β (red) multiplets of the cross-peaks involving **H4b** (2.705 ppm) to C13 (4.2 Hz), C6 (6.0 Hz) and C5 (5.0 Hz); and multiplets of the cross-peaks involving **H4a** (2.514 ppm) to C13 (5.6 Hz), C6 (3.6 Hz) and C5 (4.9 Hz). Measurement of $^{2,3}J_{CH}$ values were performed, as before, by analysis of IPAP multiplet patterns. (c-h). Selective 1D-NOESY spectrum of H4b showing the NOE contact between H14b (2.570 ppm), H2a, H2b, H20 and H17 (i). 3D model of 3S*,7R*,11S* showing NOE contact between H4b and H14b and between H4a and H6a (j). Detail of 2D NOESY spectrum of merediterpene **1b** showing the interaction between H4a and H6a (k).

(a)

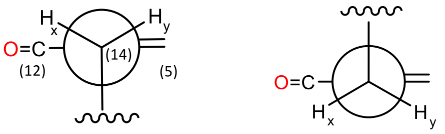

Experimental *J*-couplings

(b)

Carbon	Hx	Hy	Hx	Hy	H14b (2.570 ppm)	H14a (2.506 ppm)
C12	3.4	5.8	3.9	5.4	5.3	3.7
C5	6.3	4.3	6.6	4.0	3.9	5.2
C13	6.7	4.0	6.4	3.9	4.6	NR

(c)

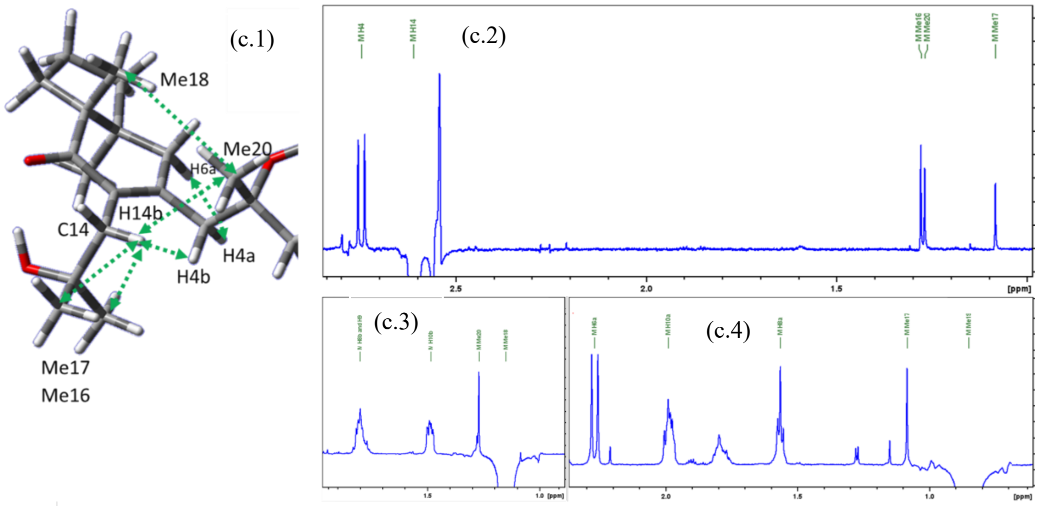

(d) (e) (f) (g) (h) (i)

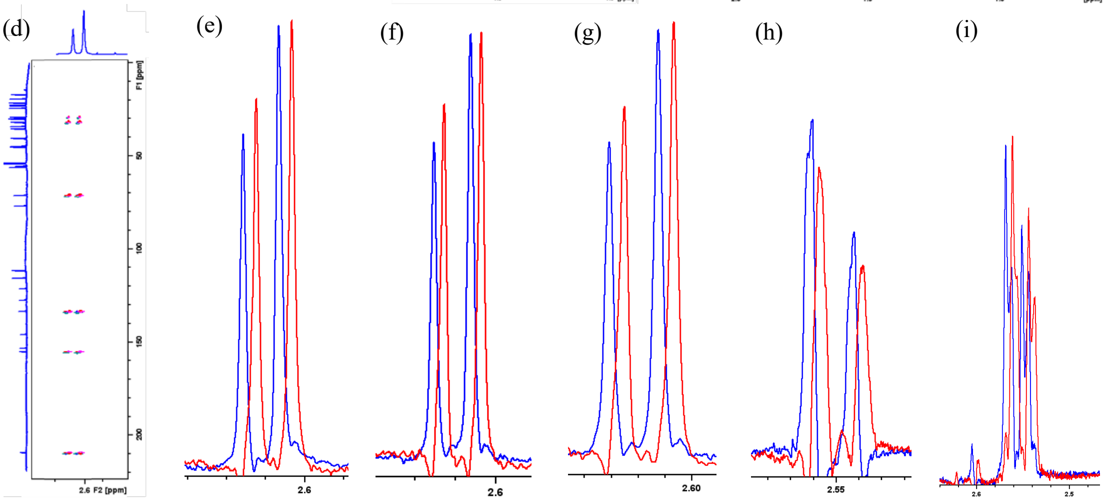

Figure S26. Rotamer fragment around ρ3 [C12-C13-C14-C15] of meroditerpene **1b** (a). Relevant experimental and computed (level of theory GIAO/OLYP/Def2TZV) $^{2,3}J_{CH}$ around ρ3 (b). Molecular model of compound **1b**, showing NOE (indicated by a green dashed arrow) contacts between H14b and H4b, H20, H16 and H17, between H4a and H6a, and finally between H20 and H18 (c.1). Selective 1D NOESY spectrum of H14b showing the NOE contact between H4b and H16, H17, and H20 (c.2). Selective 1D NOESY spectrum of H18 showing the NOE contact with H20 (c.3). Selective 1D NOESY spectrum of H19 showing the NOE contact with H6a, H10a, H8a, and H17 (c.4). 2D HSQMBC-IPAP spectrum (optimized to 6 Hz) after selective inversion of **H14b** (2.570 ppm) proton of compound **1b** (d). IPAP-HSQMBC α (blue) and β (red) multiplets of the cross-peaks involving from left to right H14b to C12 (5.3 Hz), H14b to C5 (3.9 Hz), H14b to C13 (4.6 Hz), **H14a** to C12 (3.7 Hz) and finally H14a to C5 (5.2 Hz) (e-i). Measurement of $^{2,3}J_{CH}$ values were performed by analysis of IPAP multiplet patterns. NR: Not readable

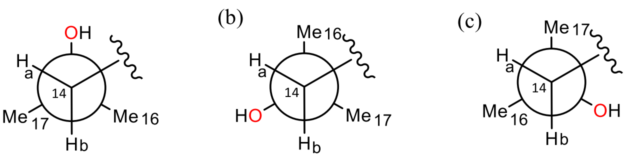

(d)

**Carbon**	Computed *J*-Coupling Rotamer a Ha	Rotamer a Hb	Rotamer b Ha	Rotamer b Hb	Rotamer c Ha	Rotamer c Hb	Experimental *J*-Coupling **H14b (2.570 ppm)**	**H14a (2.506ppm)**
Me17	3.2	1.1	5.6	2.9	0.9	5.7	2.4	4.5
Me16	5.5	1.0	2.7	5.5	1.3	3.4	1.3	1.8
C15	5.7	1.0	6.0	5.4	0.9	5.9	4.1	5.9

(e)

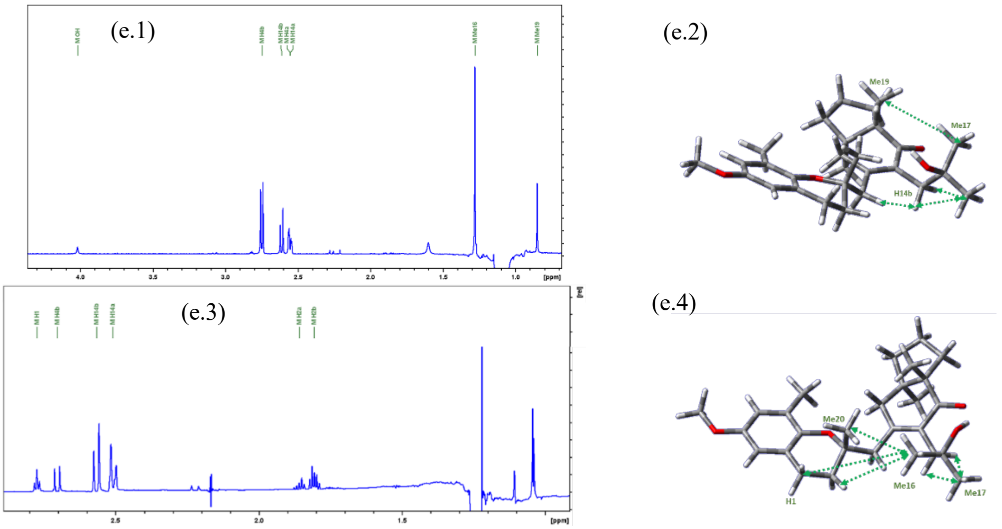

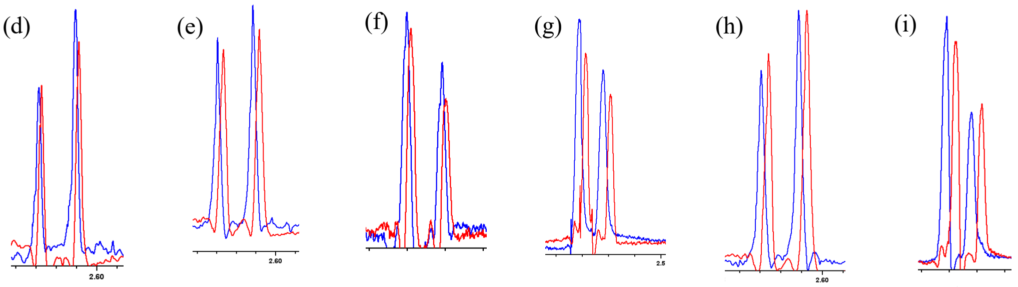

(j)

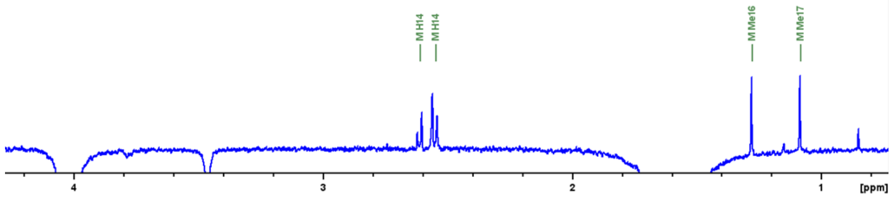

Figure S27. Rotamer fragment around ρ4 [C13-C14-C15-C16] of meroditerpene **1b** (a-c). Experimental and computed (GIAO/OLYP/DEF2TZV) $^{2,3}J_{CH}$ around ρ4 (d). Selective 1D NOESY spectrum of H17 showing the NOE contact between H19, H14b, H14a, H4b and H16 (e.1). Correlations are indicated in a molecular model. H17 does not show NOE contact with the chromane system (e.2). Slice from a 2D NOESY spectrum showing NOE contacts from H16 to H1, H2a, H2b, H4b, H14a and H14b (e.3). Correlations are shown in a molecular model. H17 does not show NOE contact with the bicyclo[4.3.0]nonane moiety (e.4). IPAP-HSQMBC α (blue) and β (red) multiplets of the cross-peaks involving H14b (2.570 ppm) to C16 (1.3 Hz), H14b to C17 (2.4 Hz), H14a (2.506 ppm) to C16 (1.8 Hz), H14a to C17 (4.5 Hz), H14b to C15 (4.1 Hz) and H14a to C15 (5.9 Hz) (d-i). Measurement of $^{2,3}J_{CH}$ values were performed by analysis of IPAP multiplet patterns. NOE 1D experiment on selective inversion over OH-C15 (j).

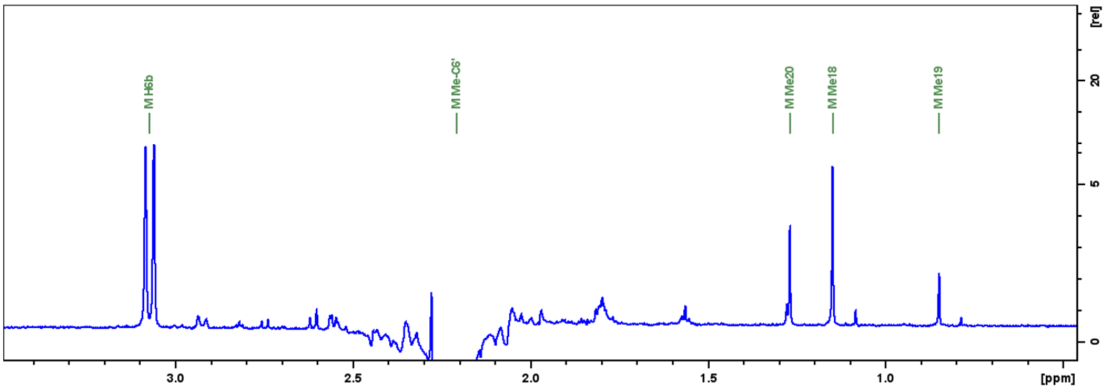

Figure S28. NOE 1D experiment of **1b** on selective inversion over Me-C6' showing NOE contacts to H6b, H20, H8 and H19. (CD_2Cl_2, NS: 400, 800 MHz). Parameters: Mixing time: 450 ms.

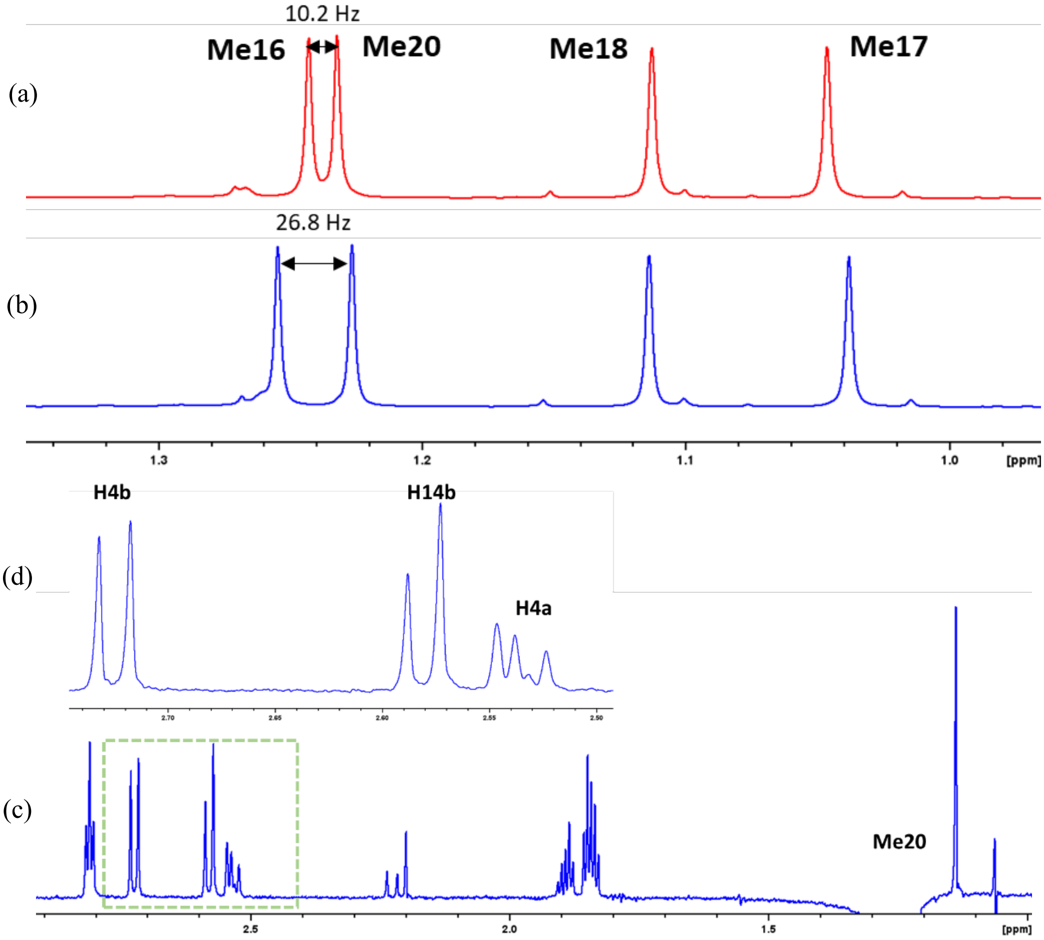

Figure S29. ^{1}H 1D spectrum section of **1b** from 1 ppm until 1.3 ppm at 298.1 k (red line) and at 280 K (blue line) (a,b). Selective NOE experiment over H20 at 280 K (c), showing in the inset (green dashed box) NOE contacts between H20 and H4b, H14b and H4a (d). Both experiments were measured in a 950 MHz Bruker spectrometer (Pulprog: *selnogp*, NS: 40, mixing time: 500 ms).

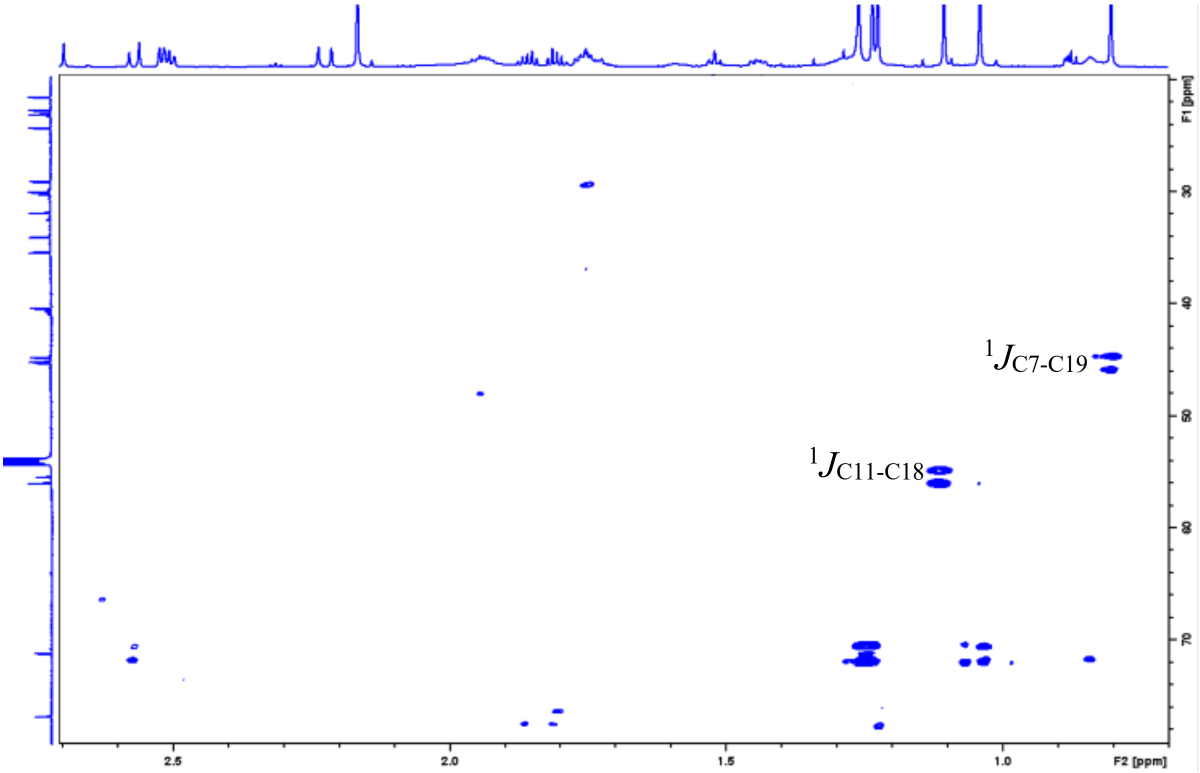

Figure S30. *J*-modulated ADEQUATE spectrum of **1b** (*adeq11etgpjcrdsp*) (a). Parameters: $^1J_{CH}$ = 138 Hz, $^1J_{CC}$ = 40 Hz, Scaling factor = 8, relaxation delay = 1.5 s, NUS: 35%/512/89. (800 MHz/CD_2Cl_2)

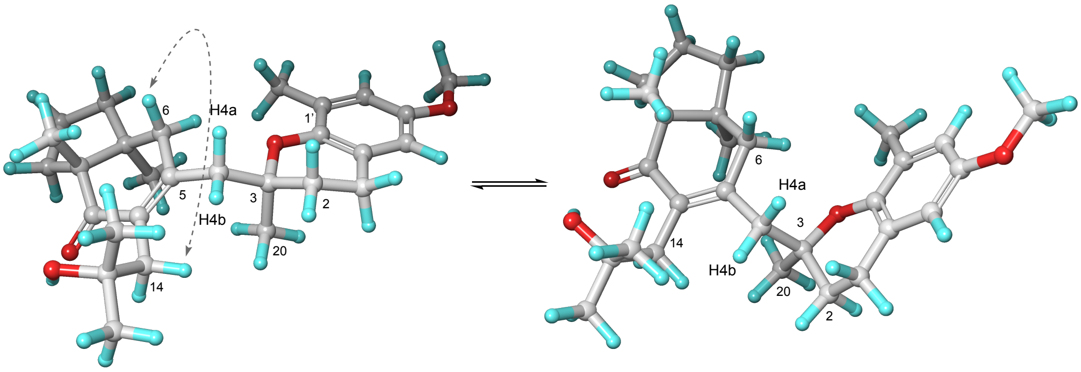

Figure S31. Carbon-proton coupling constants from an IPAP-HSQMBC experiment and NOE contacts to relate C3, C7, and C11 stereogenic centers of **(3S*,7S*,11R*)-1b**). $^2J_{CH}$ sign was measured as absolute value.

Table S5. DP4 (a) and i*J*-DP4 (b) Analysis of **1b**. **1**=(**3R7S11R)-1b**, **2**=(**3S7S11R)-1b**.

(a)

Settings	Type of data (shifts)
Default	Shielding tensors

Default settings	TMS	σ	v	Custom settings	TMS	σ	v
H	31.830573	0.185	14.18	H	31.830573	0.185	14.18
C	192.29325	2.306	11.38	C	192.29325	2.306	11.38
J	-	0.992	3.06	J	-	0.992	3.06
Slope scaling J		0.9509		Slope scaling J		0.9509	
Intercept scaling J		-0.1405		Intercept scaling J		-0.1405	

Most Likely Isomers		
Rank	Isomer	Probability
1º	1	100.00
2º	2	0.00
3º	-	-
4º	-	-

Isomer Nº		1	2	3	4	5	6	7	8	9	10	11	12	13	14	15
DP4 (%)	H	100.00	0.00	-	-	-	-	-	-	-	-	-	-	-	-	-
	C	94.25	5.75	-	-	-	-	-	-	-	-	-	-	-	-	-
	H+C	*100.00*	*0.00*	-	-	-	-	-	-	-	-	-	-	-	-	-
	J	-	-	-	-	-	-	-	-	-	-	-	-	-	-	-
	**all data**	**100.00**	**0.00**	-	-	-	-	-	-	-	-	-	-	-	-	-

(b)

Settings	Type of data (shifts)
Default	Shielding tensors

Default settings	TMS	σ	v	Custom settings	TMS	σ	v
H	31.8305727	0.185	14.18	H	31.8305727	0.185	14.18
C	192.29325	2.306	11.38	C	192.29325	2.306	11.38
J	-	0.992	3.06	J	-	0.992	3.06
Slope scaling J		0.9509		Slope scaling J		0.9509	
Intercept scaling J		-0.1405		Intercept scaling J		-0.1405	

Most Likely Isomers		
Rank	Isomer	Probability
1º	1	100.00
2º	2	0.00
3º	-	-
4º	-	-

Isomer Nº		1	2	3	4	5	6	7	8	9	10	11	12	13	14	15
DP4 (%)	H	100.00	0.00	-	-	-	-	-	-	-	-	-	-	-	-	-
	C	95.43	4.57	-	-	-	-	-	-	-	-	-	-	-	-	-
	H+C	*100.00*	*0.00*	-	-	-	-	-	-	-	-	-	-	-	-	-
	J	-	-	-	-	-	-	-	-	-	-	-	-	-	-	-
	**all data**	**100.00**	**0.00**	-	-	-	-	-	-	-	-	-	-	-	-	-

Note: Next pages are scaled, and unscaled values obtained from the DP4 and i*J*-DP4 analysis of **1b.**

UNSCALED ^{13}C-DP4

Exp 1b	(3R7S11R)-1b	(3S7S11R)-1b	Δδ (3R7S11R)-1b	Δδ (3S7S11R)-1b
115.64	108.27	108.12	7.37	7.52
153.13	148.55	148.79	4.58	4.34
111.58	110.45	110.15	1.13	1.43
121.16	123.37	124.75	2.21	3.59
145.77	142.11	141.98	3.66	3.79
127.43	124.99	125.33	2.44	2.10
23.06	26.91	27.41	3.85	4.35
34.04	37.64	37.88	3.60	3.84
76.80	79.00	80.32	2.20	3.52
40.37	41.67	41.03	1.30	0.66
133.45	133.93	134.67	0.48	1.22
155.10	164.21	164.45	9.11	9.35
45.15	44.23	45.58	0.92	0.43
17.08	19.70	19.73	2.62	2.65
55.97	52.74	52.76	3.23	3.21
71.17	69.76	70.21	1.41	0.96
31.88	29.54	30.29	2.34	1.59
29.08	30.49	29.68	1.41	0.60
209.32	206.20	206.11	3.12	3.21
55.42	63.12	63.00	7.70	7.58
45.15	54.24	54.28	9.09	9.13
44.77	48.33	47.73	3.56	2.96
30.03	33.48	33.53	3.45	3.50
19.32	21.02	21.09	1.70	1.77
35.38	36.15	36.17	0.77	0.79
22.71	23.59	23.73	0.88	1.02
21.51	22.22	22.40	0.71	0.89
24.28	27.63	29.48	3.35	5.20
			**3.15**	**3.26**
			RMSD	RMSD

UNSCALED ^{1}H-DP4

Exp 1b	(3R7S11R)-1b	(3S7S11R)-1b	Δδ (3R7S11R)-1b	Δδ (3S7S11R)-1b
6.56	6.71	6.70	0.15	0.14
6.45	6.56	6.56	0.11	0.11
2.77	2.88	2.85	0.11	0.08
2.77	2.71	2.62	0.07	0.16
1.86	2.06	1.92	0.20	0.06
1.81	1.94	2.11	0.13	0.31
2.71	2.64	2.83	0.07	0.12
2.57	2.62	2.53	0.05	0.04
2.51	2.72	2.85	0.21	0.34
2.77	2.82	2.77	0.05	0.01
2.17	2.32	2.33	0.16	0.16
3.70	3.82	3.82	0.12	0.12
1.23	1.10	1.15	0.13	0.07
1.04	1.20	1.17	0.16	0.13
3.04	3.24	2.89	0.20	0.15
2.22	2.44	2.73	0.22	0.51
1.94	2.29	1.67	0.35	0.27
1.44	1.67	2.30	0.23	0.86
1.74	1.91	1.92	0.17	0.17
1.74	1.90	1.88	0.16	0.14
1.75	2.05	1.65	0.29	0.11
1.52	1.63	1.96	0.11	0.44
0.80	1.02	1.13	0.21	0.33
1.11	1.39	1.37	0.28	0.27
1.24	1.39	1.37	0.15	0.13
			**0.16**	**0.21**
			RMSD	RMSD

SCALED ^{13}C-DP4

Exp 1b	(3R7S11R)-1b	(3S7S11R)-1b	Δδ (3R7S11R)-1b	Δδ (3S7S11R)-1b
115.64	108.23	107.82	7.41	7.82
153.13	149.59	149.58	3.54	3.55
111.58	110.48	109.90	1.10	1.68
121.16	123.73	124.90	2.57	3.74
145.77	142.97	142.58	2.80	3.19
127.43	125.40	125.49	2.03	1.94
23.06	24.71	24.96	1.65	1.90
34.04	35.72	35.71	1.68	1.67
76.80	78.18	79.28	1.38	2.48
40.37	39.86	38.94	0.51	1.43
133.45	134.58	135.07	1.13	1.62
155.10	165.67	165.65	10.57	10.55
45.15	42.50	43.62	2.65	1.53
17.08	17.31	17.08	0.23	0.00
55.97	51.22	50.99	4.75	4.98
71.17	68.70	68.90	2.47	2.27
31.88	27.41	27.93	4.47	3.95
29.08	28.39	27.29	0.69	1.79
209.32	208.77	208.42	0.55	0.90
55.42	61.89	61.50	6.47	6.08
45.15	52.76	52.55	7.61	7.40
44.77	46.70	45.82	1.93	1.05
30.03	31.46	31.25	1.43	1.22
19.32	18.66	18.47	0.66	0.85
35.38	34.20	33.95	1.18	1.43
22.71	21.30	21.18	1.41	1.53
21.51	19.89	19.82	1.62	1.69
24.28	25.45	27.09	1.17	2.81
			**2.70**	**2.89**
			RMSD	RMSD

SCALED [1]H-DP4

Exp 1b	(3R7S11R)-1b	(3S7S11R)-1b	Δδ (3R7S11R)-1b	Δδ (3S7S11R)-1b
6.56	6.62	6.69	0.06	0.13
6.45	6.47	6.54	0.02	0.09
2.77	2.74	2.71	0.03	0.06
2.77	2.57	2.48	0.21	0.30
1.86	1.91	1.76	0.05	0.10
1.81	1.79	1.95	0.02	0.15
2.71	2.50	2.69	0.21	0.01
2.57	2.48	2.39	0.09	0.18
2.51	2.58	2.71	0.07	0.20
2.77	2.68	2.63	0.09	0.14
2.17	2.18	2.18	0.01	0.01
3.70	3.69	3.72	0.01	0.02
1.23	0.94	0.96	0.29	0.26
1.04	1.04	0.98	0.00	0.06
3.04	3.11	2.75	0.07	0.28
2.22	2.30	2.60	0.07	0.37
1.94	2.15	1.50	0.21	0.44
1.44	1.51	2.15	0.07	0.71
1.74	1.76	1.75	0.02	0.01
1.74	1.75	1.72	0.01	0.02
1.75	1.90	1.48	0.15	0.28
1.52	1.48	1.80	0.04	0.28
0.80	0.86	0.95	0.05	0.14
1.11	1.23	1.19	0.13	0.09
1.24	1.23	1.19	0.00	0.05
			**0.08**	**0.18**
			RMSD	RMSD

UNSCALED ^{13}C-iJ/DP4

Exp 1b	(3R7S11R)-1b	(3S7S11R)-1b	Δδ (3R7S11R)-1b	Δδ (3S7S11R)-1b
115.64	108.25	108.03	7.39	7.61
153.13	148.44	148.65	4.69	4.48
111.58	110.54	110.22	1.04	1.36
121.16	122.92	124.16	1.76	3.00
145.77	142.04	141.73	3.73	4.04
127.43	124.66	124.82	2.77	2.61
23.06	26.57	26.64	3.51	3.58
34.04	37.55	38.48	3.51	4.44
76.80	78.47	79.58	1.67	2.78
40.37	41.62	40.36	1.25	0.01
133.45	133.66	135.01	0.21	1.56
155.10	164.47	164.38	9.37	9.28
45.15	43.08	43.70	2.07	1.45
17.08	19.19	19.05	2.11	1.97
55.97	52.60	52.47	3.37	3.50
71.17	69.54	70.04	1.63	1.13
31.88	29.24	30.36	2.64	1.52
29.08	30.53	28.76	1.45	0.32
209.32	206.47	205.83	2.85	3.49
55.42	63.00	62.69	7.58	7.27
45.15	54.12	53.84	8.97	8.69
44.77	48.37	46.78	3.60	2.01
30.03	33.33	33.11	3.30	3.08
19.32	20.86	20.78	1.54	1.46
35.38	35.98	35.87	0.60	0.49
22.71	23.42	23.45	0.71	0.74
21.51	22.07	22.12	0.56	0.61
24.28	26.79	28.58	2.51	4.30
			**3.08**	**3.10**
			**RMSD**	**RMSD**

UNSCALED [1]H-iJ/DP4

Exp 1b	(3R7S11R)-1b	(3S7S11R)-1b	Δδ (3R7S11R)-1b	Δδ (3S7S11R)-1b
6.56	6.71	6.70	0.15	0.14
6.45	6.56	6.56	0.11	0.11
2.77	2.88	2.85	0.11	0.08
2.77	2.71	2.62	0.07	0.16
1.86	2.06	1.92	0.20	0.06
1.81	1.94	2.11	0.13	0.31
2.71	2.64	2.83	0.07	0.12
2.57	2.62	2.53	0.05	0.04
2.51	2.72	2.85	0.21	0.34
2.77	2.82	2.77	0.05	0.01
2.17	2.32	2.33	0.16	0.16
3.70	3.82	3.82	0.12	0.12
1.23	1.10	1.15	0.13	0.07
1.04	1.20	1.17	0.16	0.13
3.04	3.24	2.89	0.20	0.15
2.22	2.44	2.73	0.22	0.51
1.94	2.29	1.67	0.35	0.27
1.44	1.67	2.30	0.23	0.86
1.74	1.91	1.92	0.17	0.17
1.74	1.90	1.88	0.16	0.14
1.75	2.05	1.65	0.29	0.11
1.52	1.63	1.96	0.11	0.44
0.80	1.02	1.13	0.21	0.33
1.11	1.39	1.37	0.28	0.27
1.24	1.39	1.37	0.15	0.13
			**0.16**	**0.21**
			**RMSD**	**RMSD**

SCALED ^{13}C-iJ/DP4

Exp 1b	(3R7S11R)-1b	(3S7S11R)-1b	Δδ (3R7S11R)-1b	Δδ (3S7S11R)-1b
115.64	108.35	108.04	7.29	7.60
153.13	149.53	149.63	3.60	3.50
111.58	110.69	110.28	0.89	1.30
121.16	123.38	124.56	2.22	3.40
145.77	142.97	142.55	2.80	3.22
127.43	125.16	125.23	2.27	2.20
23.06	24.67	24.71	1.61	1.65
34.04	35.92	36.82	1.88	2.78
76.8	77.84	78.91	1.04	2.11
40.37	40.09	38.76	0.28	1.61
133.45	134.38	135.66	0.93	2.21
155.1	165.94	165.73	10.84	10.63
45.15	41.58	42.17	3.57	2.98
17.08	17.11	16.93	0.03	0.15
55.97	51.34	51.15	4.63	4.82
71.17	68.69	69.14	2.48	2.03
31.88	27.41	28.51	4.47	3.37
29.08	28.72	26.88	0.36	2.20
209.32	208.97	208.18	0.35	1.14
55.42	61.99	61.62	6.57	6.20
45.15	52.89	52.55	7.74	7.40
44.77	47.00	45.33	2.23	0.56
30.03	31.59	31.33	1.56	1.30
19.32	18.82	18.70	0.50	0.62
35.38	34.31	34.16	1.07	1.22
22.71	21.44	21.44	1.27	1.27
21.51	20.06	20.08	1.45	1.43
24.28	24.89	26.70	0.61	2.42
			**2.66**	**2.90**
			**RMSD**	**RMSD**

SCALED ^{1}H-iJ/DP4

Exp 1b	(3R7S11R)-1b	(3S7S11R)-1b	Δδ (3R7S11R)-1b	Δδ (3S7S11R)-1b
6.563	6.62	6.69	0.06	0.13
6.45	6.47	6.54	0.02	0.09
2.774	2.74	2.71	0.03	0.06
2.774	2.57	2.48	0.21	0.30
1.859	1.91	1.76	0.05	0.10
1.805	1.79	1.95	0.02	0.15
2.705	2.50	2.69	0.21	0.01
2.57	2.48	2.39	0.09	0.18
2.506	2.58	2.71	0.07	0.20
2.774	2.68	2.63	0.09	0.14
2.165	2.18	2.18	0.01	0.01
3.701	3.69	3.72	0.01	0.02
1.225	0.94	0.96	0.29	0.26
1.041	1.04	0.98	0.00	0.06
3.038	3.11	2.75	0.07	0.28
2.223	2.30	2.60	0.07	0.37
1.944	2.15	1.50	0.21	0.44
1.441	1.51	2.15	0.07	0.71
1.744	1.76	1.75	0.02	0.01
1.744	1.75	1.72	0.01	0.02
1.754	1.90	1.48	0.15	0.28
1.52	1.48	1.80	0.04	0.28
0.804	0.86	0.95	0.05	0.14
1.105	1.23	1.19	0.13	0.09
1.235	1.23	1.19	0.00	0.05
			**0.08**	**0.18**
			**RMSD**	**RMSD**

6. Anisotropic measurements of 1b

Fine tuning of 3 mm compression device. We have tested our in-house-made 3 mm compression device (see the figure below) to do differentiation of epimers by studying an 800-µg sample of estrone in a 600 MHz Bruker spectrometer equipped with a 5 mm cryo-probe. Data was recorded and analyzed using the methodology and models published by our group (Nath, N.; Schmidt, M.; R. Gil, R.; Thomas Williamson, R.; E. Martin, G.; Navarro-Vázquez, A.; Griesinger, C.; Liu, Y. *J Am Chem Soc* **2016**, 138, 9548–9556) Polymerization chamber for Poly-HEMA was a 1.7 mm capillary tube. Mass of gel stick used in the 3 mm compression device was 40 mg while a gel stick of 112 mg is needed for the conventional 5 mm compression device; this implies a less severe alignment media background signal. Smaller amounts of analyte can be analyzed. (For Poly-HEMA preparation: L. F. Gil-Silva, R. Santamaría-Fernández, A. Navarro-Vázquez, R. R. Gil. *Chem A Eur. J.* **2016**, *22*, 472-476).

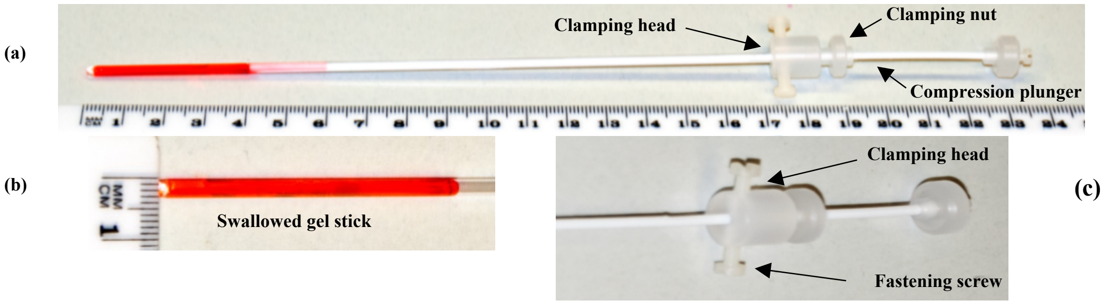

Figure S32. Experimental arrangement needed for our in-house-made 3 mm compression device. Complete alignment system shown here includes: a common 3 mm NMR tube, swallowed PMMA (75/0.25) gel in $CDCl_3$, and the semi-micro compression apparatus (a). Fully relaxed swallowed gel stick ideally has a length of 4.5 cm length, while when it is fully compressed has a length of 3.5 cm. (b). Compression device consists of a clamping head/nut and fastening screws, which keep the plunger attached to the 3 mm glass NMR tube when the gel stick is under compression (c). Gel was orange colored using a pigment from Faber Castell.

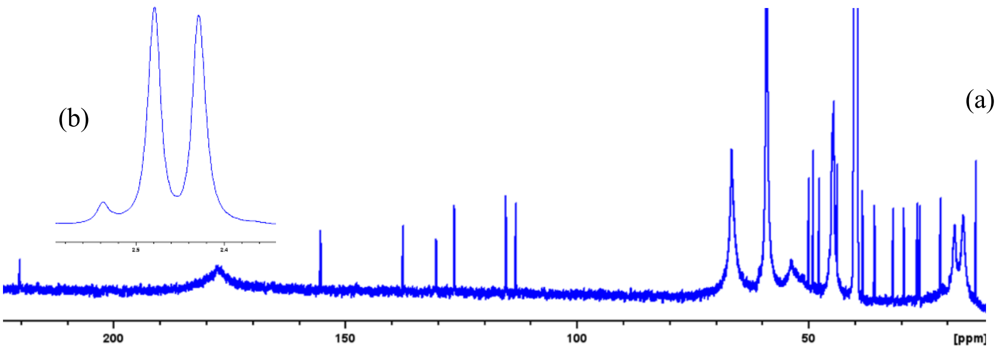

Figure S33. ^{13}C {^{1}H} spectrum of estrone (800 µg) swollen in relaxed Poly-HEMA (80/0.27) in a 3 mm compression device. Number of scans 16K. (a). Under anisotropic conditions Δ^2H_Q was 4.6 Hz (b). (NS = 16K, 600 MHz, DMSO-d_6).

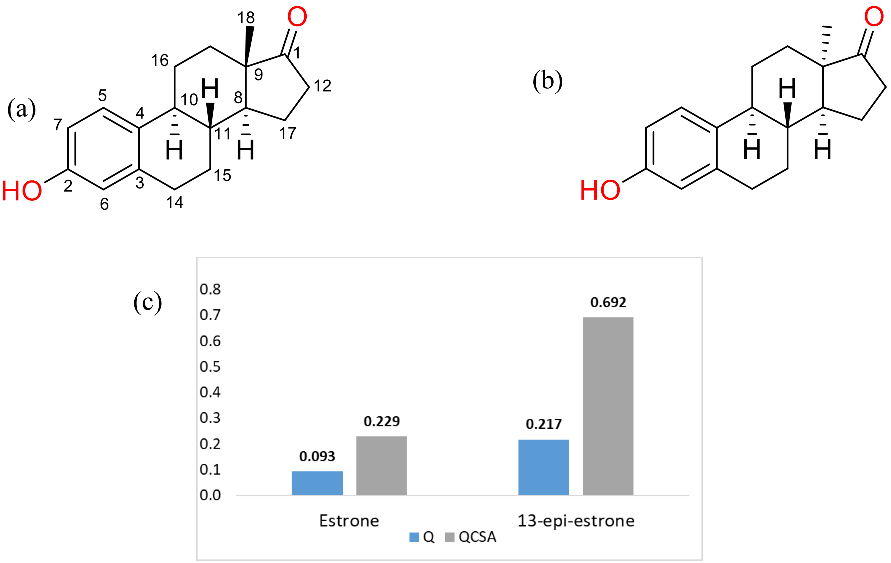

Figure S34. Chemical structure of estrone (a) and 13-*epi*-estrone (b). The quality of the data fitting is expressed as a quality factor (Q) where the correct structure is expected to have the lowest Q value. Results from the in-house 3 mm compression device: Q (blue) and Q_{CSA} (grey) factors calculated for lowest-energy structures of estrone and 13-*epi*-estrone from DFT calculation (GIAO/MPW1PW91/6-31+g(2d,p) iefpcm=DMSO) using only ^{13}C RCSAs.

Table S6. Estrone uncorrected residual chemical shift anisotropies data (Hz) analyzed in a 3 mm compression device.

Carbon	^{13}C RCSA, uncorrected (Hz)	Gel shift (Hz)
1	-1.54	-2.10
2	-0.91	-1.59
3	-4.88	-6.90
4	-1.23	-0.57
5	-1.26	-0.98
6	0.31	-1.06
7	-0.08	-0.35
8	Ref.	Ref.
**9**	-0.68	-0.33
10	-3.41	0.79
11	-0.05	-0.30
12	-0.77	-1.87
13	0.51	0.62
14	0.54	1.08
15	0.36	-0.04
16	-0.43	-2.51
17	NR	NR
18	-0.74	-2.86

NR: not readable. C8 was taken as reference

A first attempt to establish the relative configuration of meroditerpene **1b** by ^{13}C RCSA involved the use of a 4 mg sample in protonated PMMA swollen in CD_2Cl_2 using 5 mm outer diameter compression device. Also, 2.2 mg of **1b** was dissolved in CD_2Cl_2 and swollen into a deuterated chemically cross-linked poly (methyl methacrylate) gel (PMMA-*d*8) in an in-house made 3 mm outer diameter compression device.

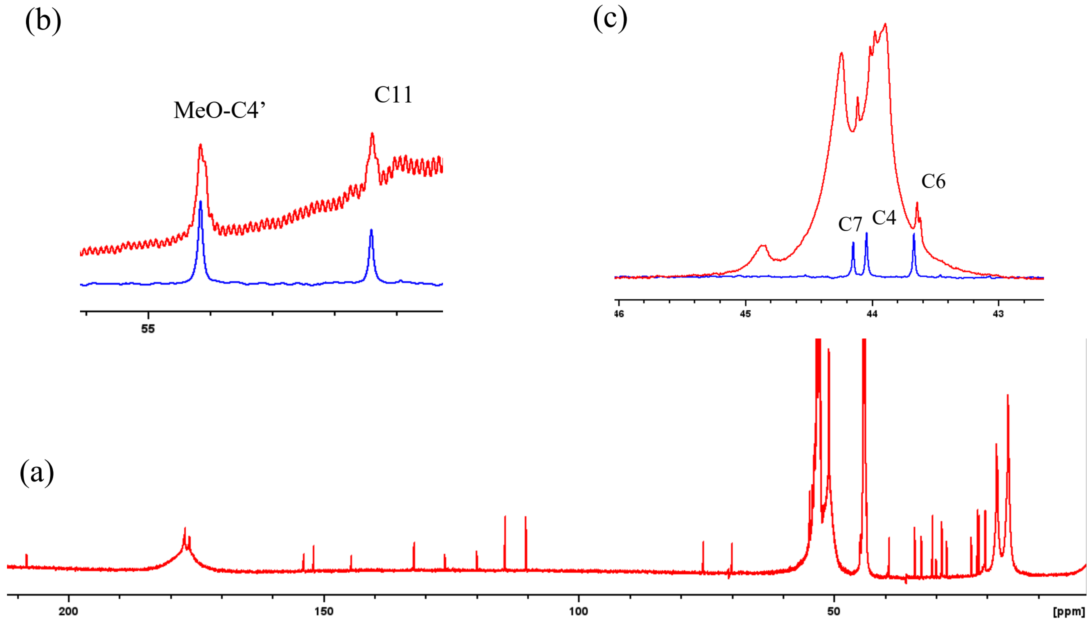

Figure S35. ^{13}C $\{^1H\}$ spectrum of **1b** swallow on PMMA-H_8 measured in a 950 MHz Bruker spectrometer (5 mm compression device) (a). Buried resonances of **1b** below polymer background signal: MeO-C4’, C11, C7, C4 and C6 are shown. In blue, isotropic ^{13}C $\{^1H\}$ spectrum of **1b** (2 mg sample in an 800 MHz Bruker spectrometer) and in red ^{13}C $\{^1H\}$ spectrum of **1b** swallow in PMMA-H8 (4 mg) (b). NS: 2048. Relaxation delay: 2 s.

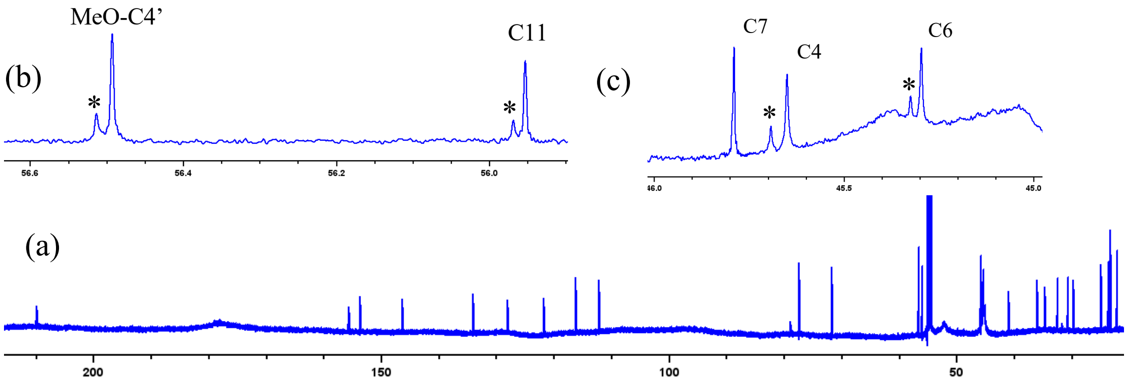

Figure 36. ^{13}C $\{^1H\}$ spectrum of **1b** swallow on PMMA-d_8 measured in an 800 MHz Bruker spectrometer (in-house-made 3 mm compression device) (a). Former buried resonances of **1b** below polymer background are visible now: MeO-C4’, C11, C7, C4 and C6. Isotropic contribution of some resonances is marked with an asterisk. Sample mass 2.2 mg. NS: 7200. Relaxation delay: 2 s.

Preparation of deuterated compression compatible PMMA gel (PMMA-d8 gel)

This procedure is a modified version of a reported before; [1,2] where the monomer and solvent are replaced by its deuterated version as Methyl-*d3* methacrylate-*d5* (MMA-*d8*) and acetone-*d6* respectably. [3,4] Monomer concentration was 70 % v/v, molar concentration of EGDMA (cross-linker) was established as 0.25 %. Another Monomer/Crosslinker ratio are also possible like 75/0.25. Cross linker and the radical initiator (V-70) were not deuterated. Polymerization was performed in either 1.5- or 1.7-mm outer diameter capillary tube in a water bath (50 °C/5 h). After gel synthesis is completed reaction chamber is opened and gel sticks are allowed to dry for 10 days. Afterward, gel sticks were washed 2 times in a methanol-*d4*/acetone-*d6* solution 1:1 (*Solution A*) for 48 h. Finally, gel sticks are subject to dialysis with the solvent chosen for the analysis several times during 48 h. Now, gels are cut into 4.5 cm pieces and are ready to be either use or storage. These gels can be stored, to our knowledge, for up to 1 year if they are immersed in deuterated solvent. The gel thus prepared, named PMMA-*d8* 70/0.25 is compatible with CD_2Cl_2 and $CDCl_3$. The same gel stick can be reused at least 2 times if it is longer soak in *solution A*. The use of protonated solvents during the gel washing must be avoided due its use contaminates the gel sticks. Chemicals used in the synthesis was purchased either at Sigma-Aldrich or Wako, while deuterated washing solvents were purchased at Deutero.

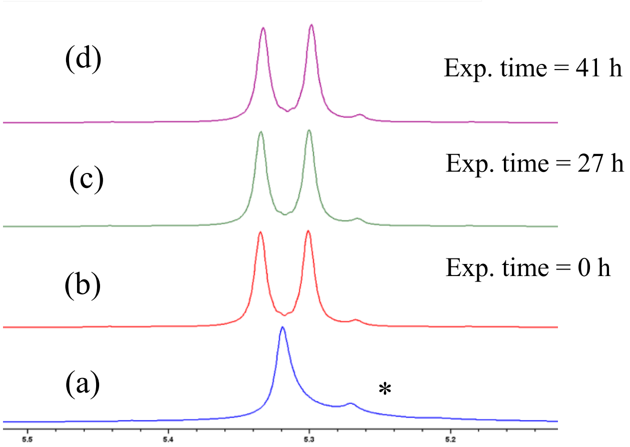

Figure S37. 1D ^{2}H spectrum of CD_2Cl_2 in the sample of compound 1b, when PMMA-*d8* gel stick is fully relaxed (a). ^{2}H spectrum of CD_2Cl_2 on fully compressed PMMA-*d8* gel (b), and evolution of it through the time. Experimental time is expressed in hours (Exp. Time) (c-d). PMMA-*d8* yields a stable Δ^2H_Q (4.2 Hz) during at least 41 h. (Solvent CD_2Cl_2).

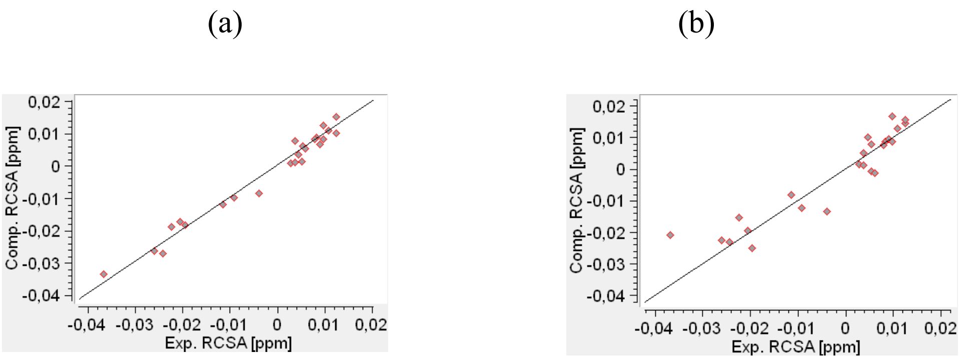

Figure S38 ^{13}C-RCSA analysis of meroditerpene **1b** swollen in 70/0.25 PMMA-*d8* gel (200 MHz, CD_2Cl_2) Fitting for carbon residual chemical shift anisotropies of diastereoisomers **(3R*,7S*,11R*)-1b** (a) and **(3S*,7S*,11R*)-1b** (b), when automatic isotropic shift correction is not applied. *Q* factors were 0.164 (a) and 0.358 (b).

In the Jackknife analysis each data set presented a student's t-distribution with N− k – 1 degrees of freedom. It allows us quickly visualized that the 24 jackknifed samples do not change the discrimination from the overall population with respect to the quality factor using the full data set and moreover, discrimination between possible configuration is present in all generated data sets. $Q_{Jack} \pm s_{Jack}$ **(3R*,7S*,11R*)-1b** = 0.101±0.004 and $Q_{Jack} \pm s_{Jack}$ **(3S*,7S*,11R*)-1b** = 0.417±0.030. Comparing these t-calculated values with the tabulated α significant level of 1% ($t_{calc.}$= 2.0687), confidence interval was computed, corroborating our initial assumption of the relative configuration of **1b** as 3R*,7S*,11R*.

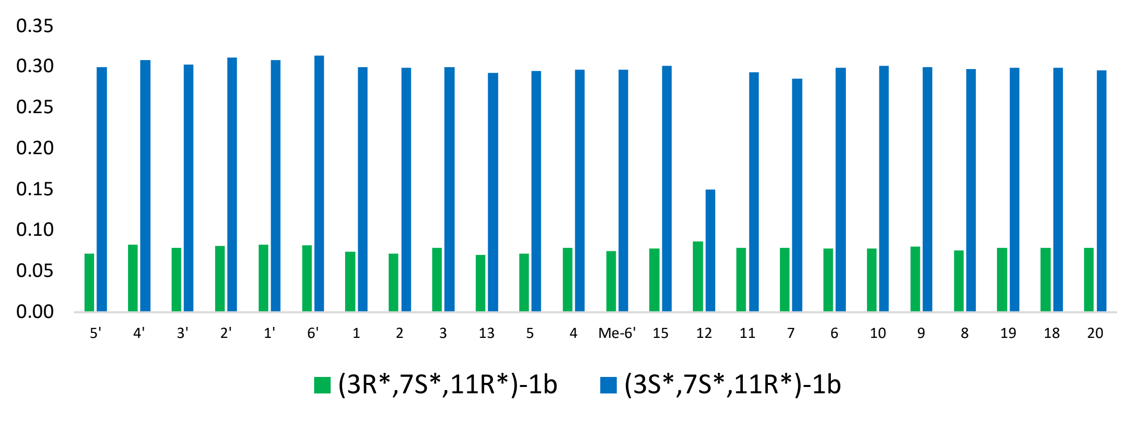

Figure S39. Resample influence bar-plot for the Jackknife analysis of ^{13}C RCSA of compound **1b**.

References

1. Gayathri, C.; Tsarevsky, N. v.; Gil, R.R. Residual Dipolar Couplings (RDCs) Analysis of Small Molecules Made Easy: Fast and Tuneable Alignment by Reversible Compression/Relaxation of Reusable PMMA Gels. c 2010, 16, 3622–3626, doi:10.1002/chem.200903378.

2. Nath, N.; Schmidt, M.; Gil, R.R.; Williamson, R.T.; Martin, G.E.; Navarro-Vázquez, A.; Griesinger, C.; Liu, Y. Determination of Relative Configuration from Residual Chemical Shift Anisotropy. Journal of the American Chemical Society 2016, 138, 9548–9556, doi:10.1021/jacs.6b04082

3. Hatada, K., Kitayama, T. & Yuki, H. Studies on the radical polymerization of methyl methacrylate using perdeuterated monomer. *Die Makromol. Chemie, Rapid Commun.* **1,** 51–56 (2018).

4. Hatada, K., Kitayama, T. & Masuda, E. Studies on the Radical Polymerization of Methyl Methacrylate in Bulk and in Benzene Using Totally Deuterated Monomer Technique. *Polym. J.* **18,** 395 (1986).

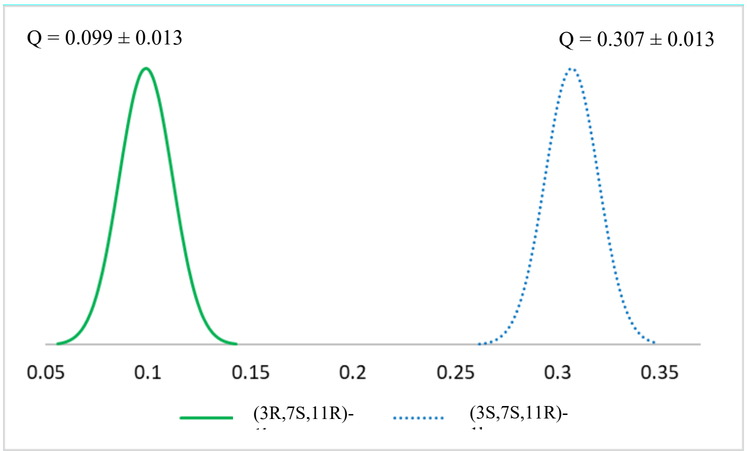

Figure S40. Bootstrapping bell curves for ^{13}C RCSA analysis of **(3R*,7S*,11R*)-1b** and **(3S*,7S*,11R*)-1b** are indicated in green line and dashed blue line, respectively. Inspection of bell curves derived from Bootstrapping, made discrimination between diastereoisomers evident. (Sample size = 5000 points, distribution: Gaussian).

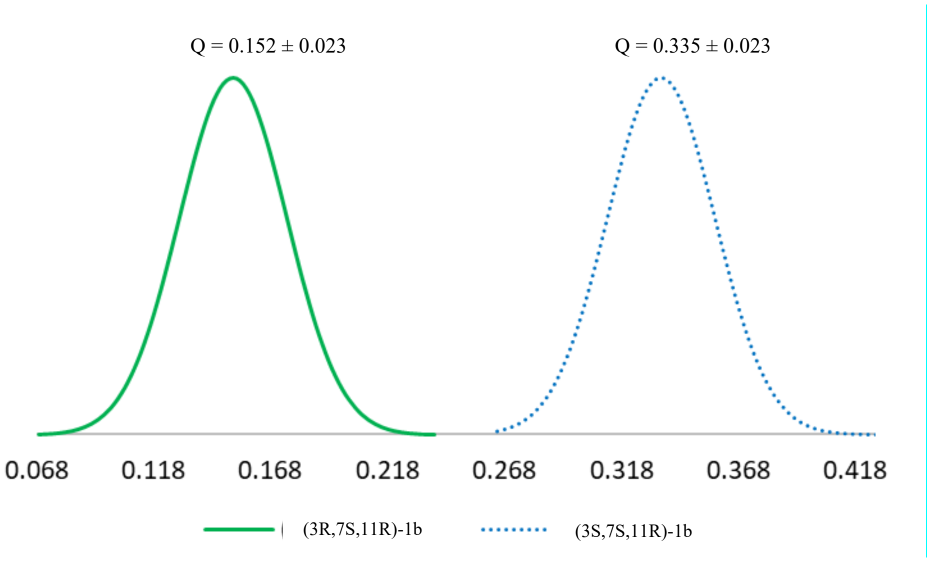

Figure S41. Bootstrapping bell curves for $^{1}D_{CH}$ analysis of **(3R*,7S*,11R*)-1b** and **(3S*,7S*,11R*)-1b** are indicated in green and dashed blue, respectively. (Sample size = 5000 points, distribution: Gaussian).

CASE 3-D analysis of both configurations of compound 1b.

(a) (b)

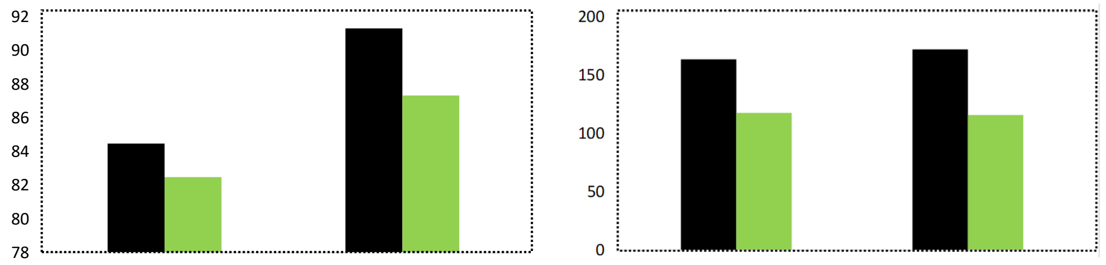

AIC: 84.455. χ^2: 82.455	AIC: 91.331. χ^2: 87.331	AIC: 163.363, χ^2: 117.363	AIC: 171.550, χ^2: 115.550
**(3*R**,7*S**,11*R**)-1b**	**(3*S**,7*S**,11*R**)-1b**	**(3*R**,7*S**,11*R**)-1b**	**(3*S**,7*S**,11*R**)-1b**

Figure S42. Bar plots for the CASE-3D analysis (AIC: black, χ^2: green) of both plausible configurations of compound **1b**. When conformer populations are fitted to its minimum by NMR isotropic data (a) and when populations populations were weighted by DFT energies at B3LYP/6-31G(d,p) level (b). Study was done on the same batch of conformers used in the *J*-DP4 analysis. The **(3*R**,7*S**,11*R**)-1b** configuration scores better.

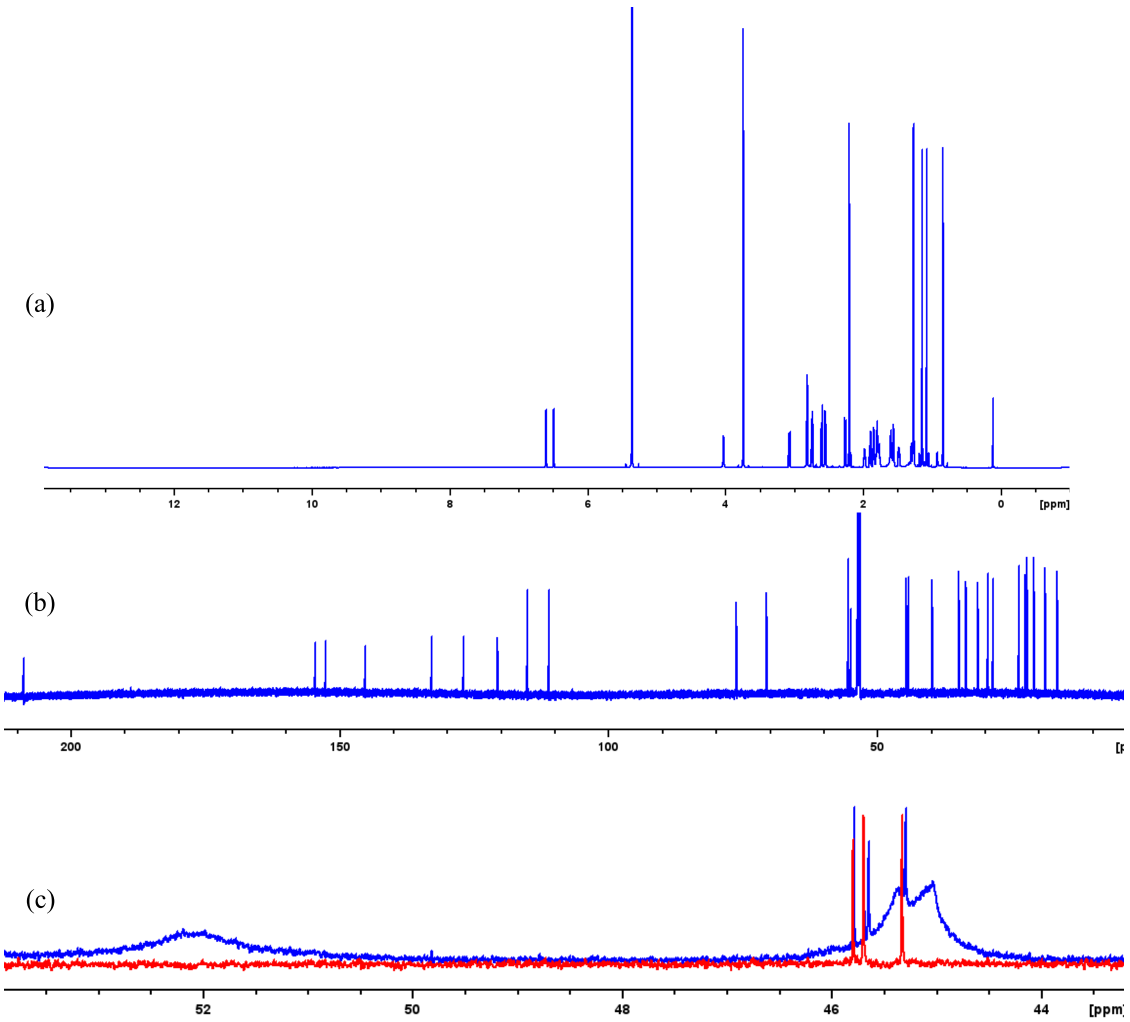

Figure S43. ^{1}H-1D experiment (a) and ^{13}C {^{1}H} spectrum (b) of **1b** recovered from the anisotropic sample (PMMA-d_8), NS was 16 and 800 respectively. No polymer resonances were observed in both ^{13}C {^{1}H} and ^{1}H 1D spectra of the recovered sample (b). Comparison between the ^{13}C {^{1}H} spectrum PMMA-H8 (blue) and PMMA-d_8 (red) (d); it is evident the reduction in the polymer background signal when PMMA-*d*8 is used. Experiments were measured either at 800 or 950 MHz/CD_2Cl_2.

Table S7. Uncorrected carbon residual chemical shift anisotropies data (Hz) for **1b**. C14 was taken as reference

Carbon	^{13}C RCSA uncorrected	Gel shift
1	1.7	11.8
2	0.7	5.2
3	1.8	14.0
4	0.9	3.2
5	-3.9	-0.3
6	0.6	6.0
7	2.0	10.7
8	2.2	13.2
9	2.5	11.8
10	2.0	11.8
11	1.1	8.5
12	-7.4	-17.1
13	-0.8	13.1
14	Ref.	Ref.
15	2.5	15.1
16	5.5	9.9
17	2.2	20.3
18	1.1	13.3
19	0.7	11.8
20	1.2	20.5
1'	-5.2	8.7
2'	-4.1	9.6
3'	-2.3	13.3
4'	-4.9	13.1
5'	-1.8	11.8
6'	-4.5	3.5
Me-6'	1.6	11.8
MeO-4'	1.1	7.6

7. Absolute configuration of 1b by TD-DFT ECD.

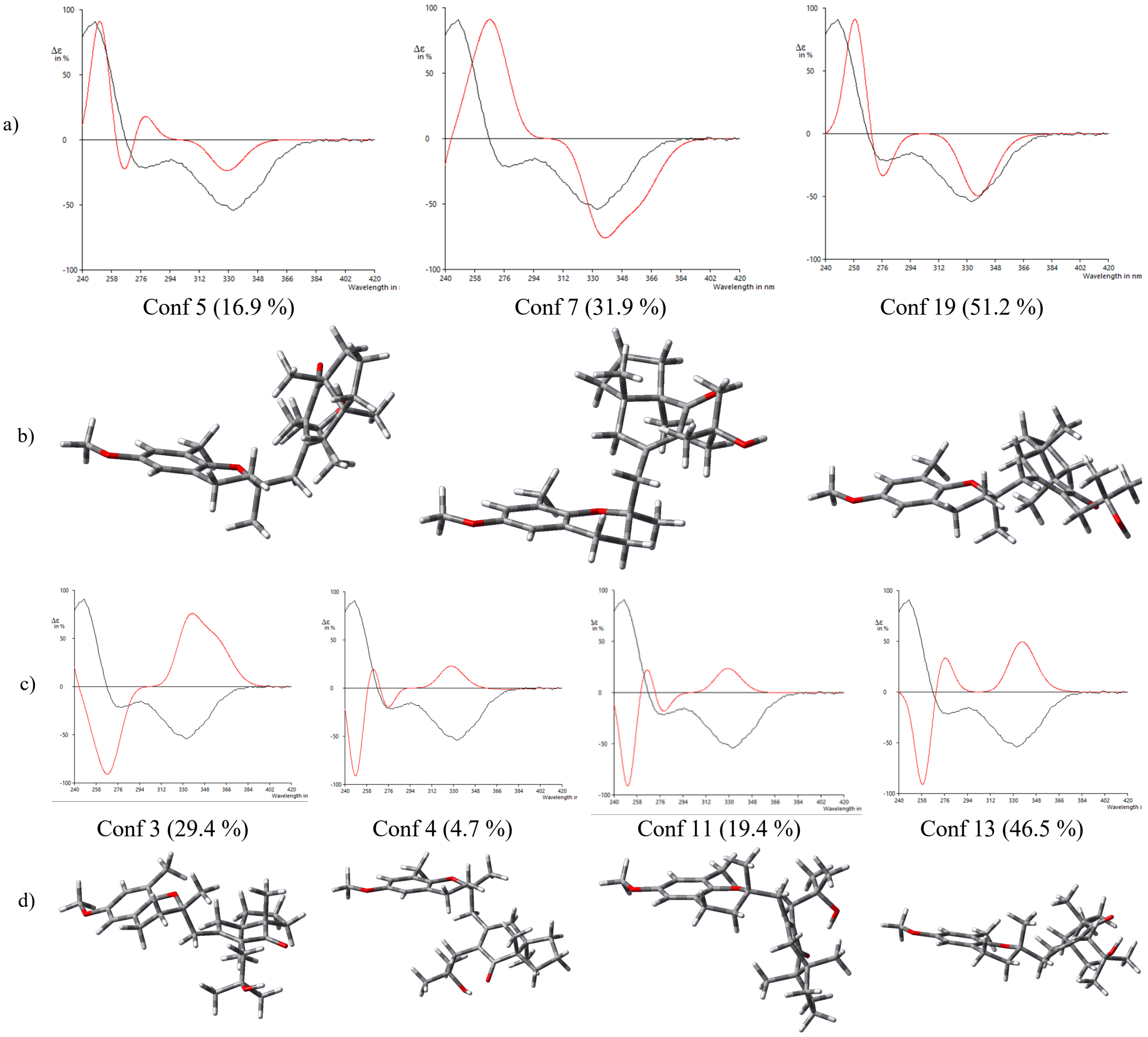

Figure 44. Calculated (red line) and experimental (black line) ECD of the conformers of **(3R,7S,11R)-1b** (a) and **(3S,7R,11S)-1b** (c). Conformers and its population are displayed in panels b and d respectively. Conformers were weighted by NMR anisotropy. ECD was computed at level: HSE0/6-311G+(2d,p)/DGA1 (solvent model/parameters/n-states: IEFPCM/CH_2Cl_2/38)

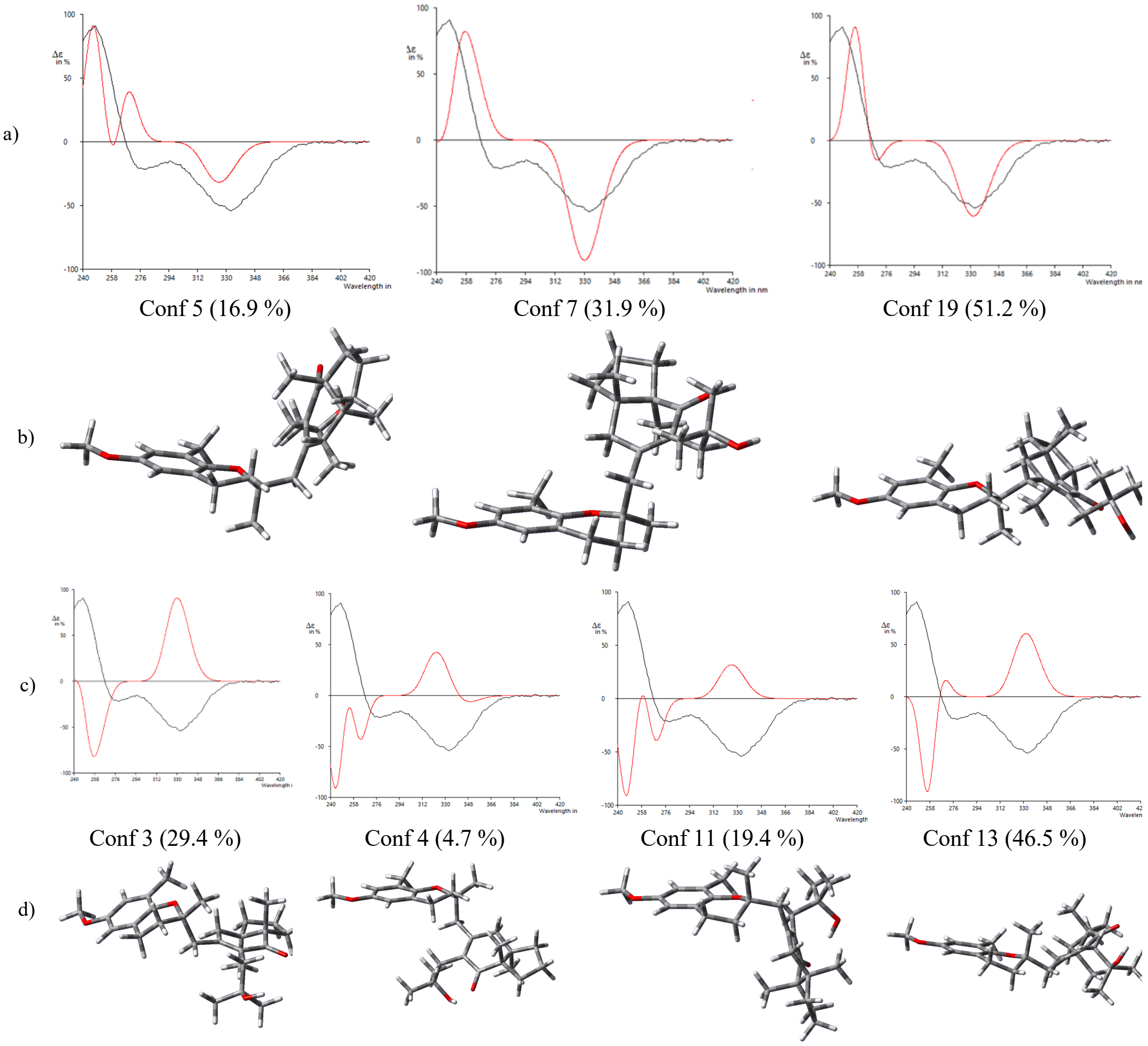

Figure 45. Calculated (red line) and experimental (black line) ECD of the conformers of **(3R,7S,11R)-1b** (a) and **(3S,7R,11S)-1b** (c). Conformers and its population are displayed in panels b and d respectively. Conformers were weighted by NMR anisotropy. ECD was computed at level: PBE0/Def2TZV/W06 (solvent model/parameters/n-states: COSMO/CH_2Cl_2/50).

Table S8. Comparison of experimental and calculated $[\alpha]_D^{25}$ values for compound **1b** and both stereoisomers pairs. Optical rotation ($[\alpha]_D^{25}$) was computed at CAM-B3LYP/6-311++(2d,2p)/DGA1 IEPCM (CH_2Cl_2) level of theory.

$[\alpha]_D^{25}$ (**1b**) Exp.	$[\alpha]_D^{25}$ Calc. **(3R,7S,11R)-1b**	$[\alpha]_D^{25}$ Calc. **(3S,7R,11S)-1b**
-11.7	-49.4	+37.3

$[\alpha]_D^{25}$ is expressed in deg*dm^{-1}(g/mL)$^{-1}$

8. DFT Calculations for (-)-α-santonin

Table S9. Functional-basis set combinational used on (-)-α-santonin test systems.

Note: The combination was chosen based on the linearity of the fits, low standard deviation and necessary calculation time.

Functional	Basis set	Solvent model	Density Fitting
OLYP	Def2SV	Gas phase	*
OLYP	Def2TZV	Gas phase	*
OLYP	Def2SV	COSMO	*
OLYP	Def2SVP	COSMO	*
OLYP	Def2SVPP	COSMO	*
OLYP	Def2TZV	COSMO	*
OLYP	Def2TZVP	COSMO	*
OLYP	Def2TZVPP	COSMO	*
OLYP	Def2QZV	COSMO	*
OLYP	Def2QZVP	COSMO	*
OLYP	Def2QZVPP	COSMO	*
mPW1PW91	6-311+g(2d,p)	Gas phase	*
mPW1PW91	6-311++g(2d,p)	Gas phase	*
mPW1PW91	6-311+g(2d,p)	COSMO	*
mPW1PW91	6-311++g(2d,p)	COSMO	*
mPW1PW91	6-311++g(2d,p)	COSMO	*
mPW1PW91	6-311++g(3d,3p)	COSMO	*
mPW1PW91	6-31+g(d,p)	COSMO	*
mPW1PW91	6-311++g(2d,p)	IEFPCM	*
mPW1PW91	cc-pVDZ	IEFPCM	*
mPW1PW91	aug-cc-pVDZ	IEFPCM	*
mPW1PW91	cc-pVTZ	IEFPCM	*

Functional	Basis set	Solvent model	Density Fitting
PBE0	Def2SV	COSMO	AUTO
PBE0	Def2SVP	COSMO	AUTO
PBE0	Def2SVPP	COSMO	AUTO
PBE0	Def2TZV	COSMO	AUTO
PBE0	cc-pVDZ	IEFPCM	DGA1
PBE0	Aug-cc-pVDZ	IEFPCM	DGA1
B972	cc-pVDZ	IEFPCM	*
B972	Aug-cc-pVDZ	IEFPCM	*
B972	cc-pVTZ	IEFPCM	*
B972	Aug-cc-pVTZ	IEFPCM	*
B972	6-311++G(2d,p)	IEFPCM	*
B972	6-311++G(2d,p)	COSMO	*
HSE06	6-31G(2d,p)	COSMO	AUTO
HSE06	6-31G++(3d,2p)	COSMO	AUTO
HSE06	6-311G++(2d,p)	COSMO	AUTO
HSE06	6-311G+(2d,p)	COSMO	AUTO
wB97x	6-311+G(2d,p)	COSMO	AUTO
wB97x	6-311+G(2d,p)	IEFPCM	AUTO
CAM-B3LYP	6-31+gG(d,p)	IEFPCM	AUTO
CAM-B3LYP	6-311++G(2d,p)	IEFPCM	AUTO
CAM-B3LYP	6-311++G(2d,p)	COSMO	AUTO
CAM-B3LYP	cc-pVDZ	IEFPCM	AUTO
CAM-B3LYP	Aug-cc-pVDZ	IEFPCM	AUTO

* No Density Fitting required

Table S10. Computation time needed for various combinations of functional/basis set combinational. Test systems: (-)-α-santonin.

Functional	Basis set	Solvent model	Density Fitting	Calculation time (min)
OLYP	Def2TZV	Gas phase	*	29
mPW1PW91	6-311+g(2d,p)	Gas phase	*	192
PBE0	Def2TZV	COSMO	DGA1	40
HSE06	6-311+G(2d,p)	COSMO	AUTO	81
B972	6-311++G(2d,p)	IEFPCM	*	221
CAM-B3LYP	6-311++G(2d,p)	IEFPCM	AUTO	262

* No Density Fitting required

9. Molecular coordinates

Molecular coordinates of the standards

(−)-α-santonin

C	3.475210	-0.306748	-0.527337
C	2.925708	1.053918	-0.423308
C	1.498690	1.217786	0.002389
C	0.755834	0.120521	0.296746
C	1.327515	-1.312675	0.296905
C	2.743129	-1.378501	-0.202107
C	-0.716943	0.069254	0.632895
C	-1.495848	-0.754180	-0.410666
C	-1.042650	-2.207761	-0.371289
C	0.474884	-2.255090	-0.625708
O	-1.450704	1.310985	0.713530
C	-2.759606	1.091798	0.366420
C	-2.940027	-0.364143	-0.080766
O	3.622363	2.032425	-0.695775
O	-3.588493	1.963254	0.426934
C	1.360869	-1.882932	1.750523
H	-1.252602	-0.337595	-1.400299
C	1.051130	2.659340	0.029204
C	-3.981427	-0.524898	-1.186913
H	4.502256	-0.384810	-0.871415
H	3.171159	-2.378260	-0.270738
H	-0.832437	-0.410574	1.615313
H	-1.295024	-2.655861	0.598975
H	-1.556127	-2.802227	-1.135639
H	0.663158	-1.966258	-1.667131
H	0.848598	-3.279499	-0.510668
H	-3.268873	-0.917919	0.813885
H	0.361648	-2.023150	2.170006
H	1.856652	-2.859396	1.753985
H	1.919849	-1.214016	2.410847
H	1.820865	3.260796	0.520854
H	0.977813	3.049540	-0.993106
H	0.097079	2.800779	0.525829
H	-4.940832	-0.106315	-0.871940
H	-4.127494	-1.581687	-1.431504
H	-3.669832	-0.001002	-2.096693

Strychnine

Conformer 1 (Population 97.5 %)

C	2.358819	0.439179	0.172033
C	2.012958	-0.912857	0.023033
C	2.989053	-1.844756	-0.315967
C	4.307009	-1.416621	-0.511967
C	4.635872	-0.068587	-0.352967
C	3.670774	0.882314	-0.004967
C	0.552980	-1.128006	0.365033
N	1.208742	1.198062	0.488033
C	1.081602	2.563049	0.314033
O	2.037525	3.317147	0.194033
C	0.000830	0.335938	0.415033
C	-0.359450	3.074901	0.317033
C	-1.420362	2.203792	-0.411967
C	-0.814222	0.835855	-0.792967
O	-2.523355	2.141680	0.488033
C	-3.714293	1.535557	-0.022967
C	-3.717139	0.034557	0.139033
C	-2.821057	-0.767352	-0.447967
C	-2.720905	-2.251341	-0.154967
C	-1.739111	-0.243241	-1.392967
C	-0.819992	-1.405146	-1.816967
C	-0.268923	-2.079090	-0.557967
C	0.366053	-1.839025	1.730033
C	-1.098902	-2.281174	1.703033
N	-1.367868	-2.619202	0.293033
H	2.735060	-2.895713	-0.426220
H	5.074971	-2.135595	-0.781009
H	5.662214	0.254978	-0.501121
H	3.916806	1.928318	0.115560
H	-0.597956	0.476118	1.320637
H	-0.328708	4.091553	-0.077958
H	-0.683401	3.148803	1.362390
H	-1.751302	2.708509	-1.333677
H	-0.088478	1.062229	-1.588140
H	-3.849794	1.826788	-1.077808
H	-4.533867	1.981199	0.547025
H	-4.457067	-0.392404	0.814562
H	-2.973963	-2.842036	-1.044777
H	-3.432791	-2.544808	0.621993
H	-2.224675	0.185628	-2.282287
H	0.009706	-1.034818	-2.429010
H	-1.356789	-2.137107	-2.429171
H	0.389220	-2.907698	-0.853249
H	0.605041	-1.191153	2.579037
H	1.032852	-2.707258	1.774778
H	-1.285770	-3.143941	2.353601
H	-1.750350	-1.461890	2.048713

Conformer 2 (Population 2.5 %)

C	-2.315948	0.484072	-0.171963
C	-1.953006	-0.867943	-0.064963
C	-2.933047	-1.826902	0.172037
C	-4.268030	-1.428845	0.307037
C	-4.610972	-0.078830	0.195037
C	-3.642930	0.899128	-0.049963
C	-0.464014	-1.060007	-0.303963
N	-1.165915	1.272022	-0.387963
C	-1.064856	2.634018	-0.206963
O	-2.037825	3.373060	-0.110963
C	0.048049	0.422971	-0.343963
C	0.362168	3.196957	-0.155963
C	1.493129	2.279909	0.390037
C	0.900071	0.928934	0.828037
O	2.451124	2.181868	-0.662963
C	3.665096	1.501816	-0.333963
C	3.566031	-0.001180	-0.465963
C	2.744998	-0.759144	0.272037
C	2.637934	-2.263141	0.095037
C	1.843024	-0.167106	1.355037
C	0.926977	-1.268067	1.927037
C	0.259945	-1.996038	0.761037
C	-0.167046	-1.798020	-1.657963
C	0.676901	-3.041057	-1.278963
N	1.260912	-2.792082	0.037037
H	-2.669966	-2.878799	0.248042
H	-5.037685	-2.171656	0.494212
H	-5.649510	0.222622	0.297481
H	-3.897337	1.946724	-0.135760
H	0.621861	0.580437	-1.259992
H	0.288199	4.124556	0.415687
H	0.642625	3.481460	-1.176827
H	1.977584	2.759487	1.255716
H	0.221858	1.167889	1.659953
H	3.980629	1.790917	0.682627
H	4.411012	1.891946	-1.031215
H	4.178205	-0.470312	-1.235202
H	3.148950	-2.744326	0.943357
H	3.175099	-2.581554	-0.802903
H	2.467467	0.255328	2.156121
H	0.174000	-0.828488	2.589065
H	1.489330	-1.995025	2.522775
H	-0.504130	-2.684883	1.144250
H	0.392653	-1.141063	-2.330936
H	-1.090492	-2.075010	-2.173753
H	0.028049	-3.924846	-1.207686
H	1.453391	-3.273551	-2.013764

Estrone

Epi-Estrone

O	5.936588	-0.237916	0.279353	O	5.818590	-0.254463	0.523426
C	2.589156	-1.513060	-0.339099	C	2.506981	-1.541761	-0.242045
C	3.963629	-1.469877	-0.157769	C	3.868115	-1.496075	0.017388
C	4.575235	-0.248612	0.105147	C	4.470383	-0.266535	0.266783
C	3.802059	0.901947	0.176343	C	3.702537	0.889065	0.238471
C	2.416765	0.857595	-0.005149	C	2.330281	0.841018	-0.025392
C	1.639957	2.154910	0.108979	C	1.553505	2.140935	-0.048008
C	0.215761	2.065413	-0.435825	C	0.204379	2.012778	-0.750980
C	-0.461920	0.788460	0.061550	C	-0.553855	0.791123	-0.224865
C	0.277827	-0.443493	-0.519127	C	0.215527	-0.490997	-0.593579
C	1.783771	-0.369341	-0.263939	C	1.705105	-0.392858	-0.260113
C	-0.372184	-1.770713	-0.069096	C	-0.494464	-1.697407	0.038169
C	-1.889422	-1.828465	-0.337284	C	-1.910170	-1.838067	-0.518199
C	-2.594934	-0.604664	0.247960	C	-2.761847	-0.569734	-0.394729
C	-1.937722	0.681412	-0.310408	C	-2.022526	0.761405	-0.709701
C	-2.936494	1.800149	0.036008	C	-2.881816	1.832135	0.017771
C	-4.300741	1.128938	-0.233128	C	-3.364334	1.150746	1.307860
C	-4.048559	-0.380678	-0.160093	C	-3.299301	-0.343061	1.025817
C	-2.626361	-0.674432	1.797774	C	-4.018311	-0.720929	-1.289481
O	-4.873467	-1.236836	-0.391690	O	-3.636893	-1.214792	1.796027
H	6.232903	0.661773	0.464153	H	6.110571	0.651402	0.683693
H	2.138909	-2.474468	-0.548972	H	2.063197	-2.509007	-0.441891
H	4.564246	-2.369002	-0.219775	H	4.466565	-2.398741	0.026380
H	4.281599	1.856985	0.373652	H	4.175865	1.850545	0.417843
H	2.192031	2.953411	-0.394669	H	2.159893	2.917505	-0.522511
H	1.598653	2.441206	1.167238	H	1.389682	2.474717	0.984221
H	-0.346658	2.950579	-0.127939	H	-0.369483	2.930675	-0.606283
H	0.228871	2.064538	-1.532139	H	0.352883	1.902039	-1.831660
H	-0.367212	0.769260	1.154904	H	-0.557243	0.854186	0.873367
H	0.137287	-0.385172	-1.609467	H	0.138635	-0.610424	-1.685565
H	-0.170261	-1.929028	0.994863	H	-0.515552	-1.575582	1.127027
H	0.091213	-2.609135	-0.592072	H	0.045500	-2.624651	-0.158475
H	-2.065036	-1.859193	-1.417862	H	-1.843182	-2.105912	-1.578608
H	-2.302911	-2.753268	0.073283	H	-2.435897	-2.657369	-0.020692
H	-1.972865	0.578862	-1.406249	H	-2.023114	0.936115	-1.790448
H	-2.793084	2.692045	-0.574115	H	-2.322017	2.744894	0.219146
H	-2.845460	2.104238	1.081398	H	-3.733501	2.117732	-0.601908
H	-4.671926	1.352162	-1.237598	H	-2.704352	1.353134	2.156707
H	-5.093062	1.412018	0.462920	H	-4.370224	1.434607	1.623000
H	-3.131969	0.182538	2.245911	H	-3.714223	-0.768741	-2.337648
H	-1.622600	-0.720719	2.218594	H	-4.719617	0.108658	-1.180611
H	-3.162576	-1.574564	2.105585	H	-4.549099	-1.642421	-1.041716

Molecular coordinates of 3R*,7S*,11S*-1b

Conf. A (17 %)				Conformer B (32 %)				Conformer C (51 %)			
C	4.739360	0.565502	-1.015350	C	4.760616	1.050844	-0.156084	C	5.101781	1.102943	-0.050496
C	5.286212	-0.693044	-0.736552	C	5.245445	-0.097411	-0.794487	C	5.866753	-0.058177	0.113870

C	4.587864	-1.577769	0.086922	C	4.562721	-1.305776	-0.644990	C	5.232009	-1.301312	0.128729
C	3.353764	-1.224220	0.638283	C	3.394400	-1.382567	0.117403	C	3.845802	-1.403039	-0.012036
C	2.818989	0.043336	0.353409	C	2.902704	-0.216229	0.727784	C	3.089296	-0.229722	-0.165458
C	3.504916	0.947939	-0.474883	C	3.589800	1.004993	0.611535	C	3.709739	1.030919	-0.190073
C	2.599151	-2.184368	1.532171	C	2.676732	-2.700976	0.311427	C	3.161305	-2.751969	0.000242
C	1.130631	-1.768071	1.663581	C	1.730206	-2.628312	1.512890	C	1.749421	-2.659371	-0.584479
C	0.998942	-0.261573	1.938580	C	0.878080	-1.345285	1.495060	C	0.979012	-1.446762	-0.027870
O	1.592480	0.466440	0.834776	O	1.761521	-0.195939	1.507439	O	1.716792	-0.236137	-0.340622
C	-1.106568	2.015589	-0.380380	C	-3.037612	-1.896615	0.388584	C	-2.948028	-1.879373	0.794763
C	-1.719056	0.647852	-0.139920	C	-2.428940	-0.512763	0.224659	C	-2.477689	-0.502353	0.353159
C	-1.452623	-0.152405	0.931878	C	-1.091151	-0.248513	0.143478	C	-1.341286	-0.252737	-0.362387
C	-0.472046	0.235147	2.027458	C	-0.030577	-1.327879	0.222863	C	-0.375696	-1.345479	-0.782338
C	2.916959	2.303199	-0.778885	C	3.084912	2.236726	1.322035	C	2.888401	2.283121	-0.370187
O	6.488645	-1.144274	-1.222839	O	6.379052	-0.137504	-1.568630	O	7.232157	-0.076666	0.260462
C	7.237119	-0.288642	-2.073658	C	7.122656	1.059732	-1.740202	C	7.929865	1.159660	0.244975
C	-1.972954	3.254786	0.014753	C	-3.464464	-2.686655	-0.891678	C	-3.953956	-2.656586	-0.116586
C	-1.314695	4.515617	-0.574151	C	-2.271224	-3.130837	-1.740468	C	-3.327518	-3.113050	-1.436057
O	-3.310357	3.145692	-0.487923	O	-4.055692	-3.924020	-0.436158	O	-4.267738	-3.887919	0.571556
C	-2.125517	3.402131	1.532840	C	-4.480569	-1.930100	-1.755855	C	-5.250880	-1.882867	-0.382516
C	-2.646475	0.176241	-1.222982	C	-3.424617	0.616667	0.246062	C	-3.325219	0.639404	0.850093
C	-3.063518	-1.279514	-1.179197	C	-2.860746	2.024580	0.334775	C	-2.769417	2.038806	0.650558
C	-3.438807	-1.625082	0.296415	C	-1.606371	2.134859	-0.582917	C	-2.124025	2.135778	-0.764383
C	-2.188237	-1.469886	1.166963	C	-0.544014	1.156258	-0.075980	C	-0.960615	1.143918	-0.837654
C	-4.327250	-1.695825	-1.958464	C	-3.742303	3.194227	-0.148535	C	-3.758633	3.222049	0.659158
C	-4.822369	-2.975405	-1.209929	C	-2.710744	4.319654	-0.485640	C	-3.011941	4.334682	-0.146216
C	-4.040014	-3.038645	0.141973	C	-1.299287	3.646506	-0.510512	C	-1.802676	3.643446	-0.857187
C	-4.553790	-0.693551	0.844070	C	-1.933573	1.797737	-2.062640	C	-3.138669	1.806263	-1.892130
C	-1.861722	-2.098906	-1.733659	C	-2.553788	2.254108	1.844813	C	-1.757191	2.256011	1.814812
O	-2.996380	0.923108	-2.138693	O	-4.633149	0.403292	0.291346	O	-4.362997	0.440640	1.475589
C	1.715599	0.151225	3.237280	C	0.063727	-1.227982	2.782140	C	0.789922	-1.532637	1.492775
H	5.261077	1.269676	-1.653341	H	5.284372	1.996389	-0.238619	H	5.570907	2.079988	-0.072188
H	5.018942	-2.554121	0.289869	H	4.963003	-2.195000	-1.123858	H	5.837245	-2.195924	0.245489
H	2.663228	-3.199751	1.124757	H	2.120693	-2.967755	-0.597492	H	3.754713	-3.475097	-0.570659
H	3.073016	-2.225099	2.522210	H	3.409719	-3.501708	0.460933	H	3.119618	-3.139037	1.027371
H	0.614605	-1.991354	0.724658	H	1.074429	-3.504779	1.548889	H	1.807652	-2.553103	-1.674746
H	0.634829	-2.336559	2.458146	H	2.315537	-2.622847	2.440429	H	1.186843	-3.575867	-0.376612
H	-0.142993	2.090883	0.124336	H	-2.369865	-2.554465	0.950919	H	-2.100572	-2.548368	0.961676
H	-0.899074	2.098074	-1.453544	H	-3.943926	-1.778501	0.992492	H	-3.444848	-1.754068	1.763038
H	-0.862621	-0.137359	2.982391	H	0.624352	-1.224837	-0.650762	H	-0.132323	-1.208935	-1.843497
H	-0.418649	1.320711	2.121128	H	-0.484770	-2.315805	0.154175	H	-0.846525	-2.323652	-0.692293
H	3.611295	2.903618	-1.372963	H	2.098154	2.538060	0.954286	H	3.528401	3.169672	-0.380475
H	1.978391	2.215327	-1.337075	H	2.977181	2.057431	2.397466	H	2.321589	2.257773	-1.307467
H	2.685938	2.851663	0.140870	H	3.771713	3.075583	1.179346	H	2.157428	2.399280	0.438140
H	8.138152	-0.844177	-2.337852	H	7.966153	0.798123	-2.380775	H	8.983847	0.909420	0.374502
H	6.681259	-0.043570	-2.988139	H	6.524076	1.839083	-2.229862	H	7.797048	1.685170	-0.709852
H	7.521411	0.640141	-1.561887	H	7.499954	1.440406	-0.781990	H	7.609348	1.813528	1.066673
H	-1.270654	4.448767	-1.666610	H	-1.611852	-3.787536	-1.164736	H	-2.481608	-3.781996	-1.250308
H	-1.906453	5.398227	-0.312544	H	-2.626623	-3.693212	-2.609110	H	-4.067114	-3.665894	-2.022961
H	-0.294836	4.654481	-0.196103	H	-1.696844	-2.270691	-2.096101	H	-2.984637	-2.259109	-2.027287
H	-3.289680	2.641391	-1.320005	H	-4.884598	-3.718262	0.018180	H	-4.769620	-3.672953	1.370173
H	-2.570271	2.503354	1.969901	H	-5.347069	-1.615628	-1.167125	H	-5.718797	-1.558110	0.551214
H	-1.161313	3.596667	2.015223	H	-4.043003	-1.026307	-2.190747	H	-5.066766	-0.984095	-0.978965
H	-2.792992	4.242542	1.746999	H	-4.813254	-2.579661	-2.571702	H	-5.946344	-2.525184	-0.932054
H	-1.503305	-2.310208	0.994125	H	-0.098617	1.509747	0.862529	H	-0.103633	1.493051	-0.249817
H	-2.452570	-1.528276	2.231891	H	0.297017	1.092792	-0.778737	H	-0.578553	1.063187	-1.863821
H	-5.075951	-0.899992	-1.933697	H	-4.323808	2.904813	-1.027994	H	-4.701554	2.943294	0.181083
H	-4.111493	-1.886058	-3.013303	H	-4.464406	3.502583	0.612084	H	-4.008686	3.537499	1.675585
H	-4.642490	-3.877393	-1.803139	H	-2.743216	5.122977	0.256975	H	-2.666261	5.137312	0.512727
H	-5.902202	-2.930091	-1.038712	H	-2.943464	4.782876	-1.449466	H	-3.682309	4.802365	-0.873966
H	-3.249418	-3.796247	0.098970	H	-0.736826	3.887042	0.398282	H	-0.863828	3.873725	-0.342039
H	-4.686845	-3.304203	0.985228	H	-0.691780	3.984765	-1.356970	H	-1.683829	3.976586	-1.893988
H	-4.271424	0.362288	0.843890	H	-2.303336	0.777882	-2.194832	H	-3.532363	0.788892	-1.825981
H	-5.482346	-0.785445	0.275486	H	-2.676008	2.477124	-2.488335	H	-3.990047	2.491309	-1.897803
H	-4.778086	-0.979608	1.878183	H	-1.020504	1.898252	-2.660472	H	-2.636651	1.903717	-2.861545
H	-1.686344	-1.803206	-2.773081	H	-3.489561	2.169444	2.406336	H	-2.295700	2.174115	2.764341
H	-0.933649	-1.929995	-1.183197	H	-1.851806	1.526413	2.257846	H	-0.948262	1.522038	1.825637
H	-2.076511	-3.171188	-1.728822	H	-2.150491	3.254807	2.022613	H	-1.311121	3.253517	1.773082
H	1.544366	1.212312	3.441302	H	-0.470071	-0.275763	2.825012	H	0.195062	-0.691194	1.855583
H	1.342864	-0.429798	4.087850	H	-0.672380	-2.035355	2.845153	H	0.274381	-2.459219	1.766514
H	2.794501	-0.004098	3.162644	H	0.725760	-1.289061	3.650655	H	1.752944	-1.512391	2.009153

Molecular coordinates of 3S*,7S*,11R*-1b

Conf A (7 %)				Conf B (44 %)				Conf C (49 %)			
C	-5.884446	-0.131072	0.819251	C	-4.873839	1.055504	-0.211755	C	-4.753279	0.630121	-0.970628
C	-6.122309	-0.230864	-0.557411	C	-5.332161	-0.110142	-0.838059	C	-5.285887	-0.655047	-0.811996

C	-5.042255	-0.319820	-1.437649	C	-4.619101	-1.299803	-0.680353	C	-4.579472	-1.603952	-0.071071
C	-3.727440	-0.316059	-0.964678	C	-3.447098	-1.340821	0.079021	C	-3.350837	-1.288801	0.514524
C	-3.505017	-0.225789	0.419296	C	-2.983407	-0.157581	0.678654	C	-2.829327	0.004870	0.349227
C	-4.577192	-0.128617	1.322129	C	-3.699904	1.045509	0.552398	C	-3.524447	0.974832	-0.393063
C	-2.553783	-0.404855	-1.916575	C	-2.694594	-2.638244	0.281127	C	-2.590113	-2.316881	1.322681
C	-1.258116	0.044442	-1.232246	C	-1.753175	-2.532451	1.483943	C	-1.127240	-1.900290	1.508594
C	-1.107797	-0.590418	0.159850	C	-0.928737	-1.232060	1.451192	C	-1.010961	-0.421418	1.911134
O	-2.241578	-0.190463	0.977356	O	-1.840886	-0.102538	1.455286	O	-1.607332	0.393009	0.870027
C	1.690259	2.026693	-0.725290	C	2.935145	-1.924478	0.470867	C	1.042059	1.977650	-0.289525
C	2.212568	0.683470	-0.254216	C	2.410227	-0.528341	0.183793	C	1.748568	0.657099	-0.058002
C	1.503269	-0.234856	0.462411	C	1.091494	-0.187268	0.104938	C	1.462028	-0.216627	0.947294
C	0.087526	0.008081	0.960156	C	-0.030393	-1.204670	0.169643	C	0.455172	0.079770	2.047681
C	-4.313042	-0.017372	2.803109	C	-3.222103	2.296864	1.247391	C	-2.953278	2.359656	-0.568760
O	-7.370038	-0.244035	-1.132254	O	-6.467001	-0.185320	-1.607836	O	-6.481772	-1.072920	-1.342407
C	-8.504763	-0.145795	-0.284985	C	-7.241388	0.991184	-1.786291	C	-7.235688	-0.151998	-2.116684
C	2.214168	3.279790	0.043627	C	3.426469	-2.798973	-0.725298	C	1.836097	3.265406	0.086502
C	1.692950	3.329809	1.482955	C	4.562026	-2.143205	-1.521636	C	2.053365	3.391690	1.597776
O	3.644221	3.256474	0.166830	O	3.897096	-4.045352	-0.166442	O	3.157296	3.250805	-0.477487
C	1.790049	4.543511	-0.723797	C	2.287135	-3.210043	-1.660291	C	1.073281	4.490744	-0.445683
C	3.618652	0.380638	-0.699074	C	3.469071	0.540486	0.096548	C	2.792753	0.315079	-1.085538
C	4.327270	-0.742031	0.029082	C	3.032107	1.859417	-0.517783	C	3.758391	-0.793770	-0.720045
C	3.352581	-1.959156	0.107274	C	1.706946	2.288487	0.190004	C	2.932574	-1.990560	-0.151393
C	2.120753	-1.553824	0.921028	C	0.622922	1.247002	-0.104974	C	2.215577	-1.532757	1.122033
C	5.586998	-1.353855	-0.613966	C	3.938593	3.089167	-0.317864	C	4.580522	-1.450529	-1.846048
C	5.653771	-2.792655	-0.005900	C	2.958056	4.300930	-0.437318	C	4.922317	-2.867340	-1.280240
C	4.273513	-3.058500	0.678817	C	1.509506	3.718515	-0.358092	C	4.015676	-3.083321	-0.024902
C	2.888697	-2.419091	-1.300858	C	1.887755	2.396334	1.728055	C	1.872636	-2.493069	-1.167997
C	4.724099	-0.166876	1.421155	C	2.870373	1.620936	-2.046155	C	4.752921	-0.174298	0.306326
O	4.165511	1.043789	-1.579904	O	4.616007	0.339652	0.483870	O	2.879906	0.940431	-2.142043
C	-1.094653	-2.124545	0.095552	C	-0.115686	-1.080747	2.735041	C	-1.738266	-0.125989	3.235400
H	-6.706300	-0.054996	1.522095	H	-5.420757	1.987169	-0.301572	H	-5.281929	1.384675	-1.541831
H	-5.240994	-0.385125	-2.503734	H	-4.998390	-2.202852	-1.150348	H	-4.999888	-2.599550	0.039936
H	-2.742612	0.218142	-2.798143	H	-2.128545	-2.893242	-0.625089	H	-2.637897	-3.291500	0.823675
H	-2.450286	-1.432702	-2.289957	H	-3.405623	-3.458128	0.432569	H	-3.072930	-2.453565	2.299843
H	-1.280907	1.132602	-1.108157	H	-1.078003	-3.393556	1.530782	H	-0.590438	-2.042911	0.565804
H	-0.386027	-0.196564	-1.848353	H	-2.340514	-2.529591	2.410222	H	-0.644701	-2.529398	2.264678
H	0.601076	2.057041	-0.698576	H	2.194415	-2.520311	1.009315	H	0.084672	1.992863	0.230768
H	1.979530	2.140504	-1.776684	H	3.792400	-1.803485	1.143585	H	0.812782	2.036485	-1.360382
H	0.007630	-0.406468	1.973467	H	-0.692650	-1.023392	-0.686772	H	0.842206	-0.362003	2.975454
H	-0.110734	1.076927	1.053176	H	0.368191	-2.210896	0.040212	H	0.394444	1.155750	2.223957
H	-5.250819	0.029172	3.363622	H	-3.935609	3.113811	1.108739	H	-2.023252	2.337771	-1.147345
H	-3.733210	-0.872145	3.168842	H	-3.092978	2.128936	2.322146	H	-3.662193	3.008864	-1.089862
H	-3.727206	0.878914	3.036795	H	-2.250594	2.626825	0.862670	H	-2.712867	2.815997	0.397656
H	-9.373393	-0.173465	-0.944604	H	-8.080469	0.702914	-2.421202	H	-8.128986	-0.691116	-2.435559
H	-8.557026	-0.987608	0.417985	H	-7.624481	1.370198	-0.829719	H	-7.533288	0.723482	-1.524743
H	-8.507079	0.797055	0.277639	H	-6.664687	1.781331	-2.284833	H	-6.678028	0.180997	-3.001814
H	1.967591	2.422675	2.028841	H	5.385016	-1.841544	-0.866263	H	2.572423	2.512840	1.991420
H	2.138241	4.184076	2.001807	H	4.937751	-2.853301	-2.264933	H	2.672906	4.269696	1.804868
H	0.603830	3.441144	1.506155	H	4.217946	-1.247224	-2.047212	H	1.100911	3.509358	2.125548
H	4.024709	2.942512	-0.668345	H	4.679776	-3.863961	0.372476	H	3.112121	2.863001	-1.365661
H	2.218504	4.543450	-1.732329	H	1.541266	-3.800723	-1.119615	H	0.976520	4.438967	-1.535877
H	0.699791	4.612347	-0.816144	H	1.799687	-2.333050	-2.096167	H	0.066844	4.556451	-0.016234
H	2.151300	5.434171	-0.200804	H	2.678903	-3.829685	-2.472549	H	1.619432	5.405694	-0.196203
H	2.383538	-1.442000	1.984002	H	0.268273	1.338531	-1.143255	H	2.939198	-1.396459	1.940555
H	1.365715	-2.344747	0.896695	H	-0.262566	1.413014	0.516889	H	1.526563	-2.306655	1.477498
H	5.494016	-1.379281	-1.702822	H	4.423474	3.055860	0.660991	H	3.996020	-1.513596	-2.767521
H	6.482015	-0.765970	-0.393237	H	4.741558	3.128503	-1.059440	H	5.475664	-0.870493	-2.086220
H	6.468079	-2.879335	0.720005	H	3.106538	4.841097	-1.377596	H	5.980215	-2.942501	-1.010287
H	5.856341	-3.534067	-0.784955	H	3.136603	5.024694	0.363926	H	4.741724	-3.639963	-2.033968
H	4.354826	-2.969881	1.767677	H	1.043819	3.694040	-1.349541	H	4.591422	-2.955167	0.898353
H	3.892073	-4.064217	0.470929	H	0.854377	4.317774	0.283638	H	3.583494	-4.089522	0.008635
H	2.342281	-1.642222	-1.842869	H	2.191177	1.451625	2.187344	H	1.135137	-1.728230	-1.426099
H	3.725124	-2.732882	-1.929652	H	2.630434	3.148288	2.005092	H	2.326439	-2.841270	-2.098726
H	2.222469	-3.282722	-1.193516	H	0.935282	2.695366	2.179336	H	1.334271	-3.342873	-0.732975
H	5.404692	0.676658	1.272835	H	3.841048	1.334016	-2.464331	H	5.282555	0.654452	-0.172842
H	3.872081	0.203719	1.994449	H	2.156660	0.830091	-2.287530	H	4.262184	0.227478	1.195081
H	5.247010	-0.916490	2.021407	H	2.553959	2.534062	-2.557884	H	5.498969	-0.908323	0.623344
H	-0.314798	-2.487950	-0.580238	H	0.641894	-1.867300	2.804188	H	-1.363057	-0.771632	4.036900
H	-0.929257	-2.547479	1.090550	H	0.390917	-0.113717	2.767106	H	-1.580337	0.916368	3.528024
H	-2.054613	-2.501197	-0.266372	H	-0.774674	-1.152771	3.605114	H	-2.814967	-0.286677	3.141833

10. Z-matrices and CSA tensors

Chemical Shielding tensors (GIAO MPW1PW91/6-311+G(2d,p) IEFPCM = DMSO)

Estrone

1 O Isotropic = 219.3777 Anisotropy = 69.4458
XX= 264.3507 YX= 6.1033 ZX= 6.2852
XY= 12.2448 YY= 170.4594 ZY= -7.2555
XZ= 3.9257 YZ= -9.9186 ZZ= 223.3230
Eigenvalues: 168.0872 224.3710 265.6749
2 C Isotropic = 55.5022 Anisotropy = 179.1153
XX= 29.1776 YX= -27.7221 ZX= -23.6955
XY= -30.9760 YY= -27.9407 ZY= -33.8073
XZ= -32.6648 YZ= -35.0210 ZZ= 165.2695
Eigenvalues: -48.9020 40.4961 174.9124
3 C Isotropic = 72.1356 Anisotropy = 143.6624
XX= 40.8995 YX= 39.4558 ZX= -10.4471
XY= 32.4091 YY= 12.5237 ZY= -19.7790
XZ= -13.1783 YZ= -20.6942 ZZ= 162.9836
Eigenvalues: -12.5199 61.0162 167.9105
4 C Isotropic = 27.0705 Anisotropy = 137.4049
XX= -56.8698 YX= -7.7226 ZX= -23.6249
XY= -0.9178 YY= 24.7752 ZY= -14.2821
XZ= -21.8941 YZ= -16.9502 ZZ= 113.3061
Eigenvalues: -60.3372 22.8750 118.6738
5 C Isotropic = 69.6497 Anisotropy = 118.6949
XX= 53.6038 YX= -35.9296 ZX= -16.7127
XY= -23.9319 YY= 12.0392 ZY= -23.6663
XZ= -9.9121 YZ= -27.5945 ZZ= 143.3061
Eigenvalues: -9.0903 69.2598 148.7797
6 C Isotropic = 42.3351 Anisotropy = 190.2369
XX= -6.0309 YX= 29.9459 ZX= -17.0315
XY= 29.6588 YY= -30.6512 ZY= -24.9716
XZ= -21.3428 YZ= -19.7424 ZZ= 163.6875
Eigenvalues: -50.8957 8.7413 169.1597
7 C Isotropic = 154.4653 Anisotropy = 21.4980
XX= 165.2293 YX= -1.9551 ZX= 4.6945
XY= -12.0906 YY= 154.6523 ZY= -2.4769
XZ= -1.5831 YZ= 2.9717 ZZ= 143.5144
Eigenvalues: 143.3423 151.2564 168.7973
8 C Isotropic = 158.4953 Anisotropy = 15.8645
XX= 156.3619 YX= 9.7357 ZX= -0.4150
XY= 8.9868 YY= 161.2069 ZY= -3.5179
XZ= -2.9486 YZ= -0.5169 ZZ= 157.9173
Eigenvalues: 149.1137 157.3006 169.0717
9 C Isotropic = 146.3761 Anisotropy = 5.6182
XX= 147.2654 YX= -5.7378 ZX= -0.2149
XY= 2.3441 YY= 144.2578 ZY= -0.8822
XZ= 5.1363 YZ= 1.0312 ZZ= 147.6051
Eigenvalues: 143.1569 145.8498 150.1216
10 C Isotropic = 139.8903 Anisotropy = 9.4644
XX= 136.5159 YX= -10.9680 ZX= -5.2087
XY= -5.2012 YY= 137.2817 ZY= -0.9415
XZ= 1.9661 YZ= -2.4578 ZZ= 145.8732
Eigenvalues: 128.4885 144.9824 146.1999
11 C Isotropic = 49.8868 Anisotropy = 178.9526
XX= -34.1737 YX= 0.4171 ZX= -26.8724
XY= 0.3017 YY= 22.3495 ZY= -21.5554
XZ= -22.7921 YZ= -30.7402 ZZ= 161.4845

Epi-estrone

1 O Isotropic = 219.1218 Anisotropy = 68.6206
XX= 262.8605 YX= 5.5215 ZX= 8.1459
XY= 12.5506 YY= 168.9205 ZY= -4.3697
XZ= 6.3472 YZ= -6.9293 ZZ= 225.5845
Eigenvalues: 167.3695 225.1272 264.8689
2 C Isotropic = 54.2114 Anisotropy = 180.2708
XX= 30.4358 YX= -27.5152 ZX= -30.4681
XY= -33.7173 YY= -31.3731 ZY= -28.1925
XZ= -40.3256 YZ= -27.9392 ZZ= 163.5714
Eigenvalues: -51.3790 39.6213 174.3919
3 C Isotropic = 71.6545 Anisotropy = 143.7996
XX= 42.7894 YX= 39.4362 ZX= -19.4700
XY= 32.2913 YY= 10.4966 ZY= -11.9778
XZ= -22.0020 YZ= -13.5968 ZZ= 161.6775
Eigenvalues: -12.6895 60.1320 167.5209
4 C Isotropic = 26.9623 Anisotropy = 136.6782
XX= -53.7130 YX= -8.1564 ZX= -33.5992
XY= -1.3582 YY= 23.4315 ZY= -10.1986
XZ= -28.9755 YZ= -12.9645 ZZ= 111.1682
Eigenvalues: -60.0035 22.8093 118.0811
5 C Isotropic = 69.8476 Anisotropy = 118.4808
XX= 55.1518 YX= -35.5076 ZX= -20.3520
XY= -23.7943 YY= 10.5273 ZY= -18.5356
XZ= -14.2173 YZ= -22.7220 ZZ= 143.8635
Eigenvalues: -8.7722 69.4801 148.8348
6 C Isotropic = 41.6112 Anisotropy = 189.0193
XX= -3.3718 YX= 29.3149 ZX= -29.3030
XY= 27.8971 YY= -32.4636 ZY= -11.6130
XZ= -33.4410 YZ= -8.5001 ZZ= 160.6690
Eigenvalues: -50.3238 7.5333 167.6240
7 C Isotropic = 154.0016 Anisotropy = 20.3842
XX= 164.2516 YX= -1.0015 ZX= 6.4842
XY= -10.9245 YY= 153.5705 ZY= -4.4887
XZ= 0.8051 YZ= 1.3135 ZZ= 144.1827
Eigenvalues: 143.5074 150.9063 167.5910
8 C Isotropic = 157.1621 Anisotropy = 18.1057
XX= 157.8645 YX= 10.0127 ZX= 1.0085
XY= 11.9426 YY= 156.7962 ZY= -5.6196
XZ= -2.3280 YZ= -2.6367 ZZ= 156.8258
Eigenvalues: 145.7340 156.5198 169.2326
9 C Isotropic = 143.4547 Anisotropy = 10.0813
XX= 137.7080 YX= -5.2846 ZX= -1.7386
XY= 6.2750 YY= 144.1310 ZY= 3.0805
XZ= 4.5136 YZ= 2.8138 ZZ= 148.5251
Eigenvalues: 137.5303 142.6582 150.1755
10 C Isotropic = 142.8259 Anisotropy = 11.4853
XX= 140.8036 YX= -7.8023 ZX= -1.3181
XY= 5.6449 YY= 138.4621 ZY= -2.2079
XZ= 7.0919 YZ= -1.5654 ZZ= 149.2119
Eigenvalues: 137.9762 140.0187 150.4827
11 C Isotropic = 50.3027 Anisotropy = 178.0889
XX= -30.2915 YX= 1.5943 ZX= -37.4921
XY= 0.0226 YY= 21.9711 ZY= -15.3631
XZ= -34.6645 YZ= -28.1997 ZZ= 159.2285

Eigenvalues: -37.4236 17.8954 169.1885
12 C Isotropic = 158.9744 Anisotropy = 18.5406
XX= 163.8741 YX= 7.6743 ZX= -0.3198
XY= 6.2254 YY= 161.6656 ZY= -7.5718
XZ= 1.1586 YZ= -9.1687 ZZ= 151.3835
Eigenvalues: 145.8380 159.7504 171.3348
13 C Isotropic = 153.3058 Anisotropy = 19.0065
XX= 150.3836 YX= -7.5000 ZX= -8.3517
XY= -1.8735 YY= 157.0259 ZY= 9.9132
XZ= -5.8230 YZ= 3.4093 ZZ= 152.5080
Eigenvalues: 144.2026 149.7381 165.9768
14 C Isotropic = 133.4149 Anisotropy = 22.5557
XX= 145.3260 YX= -15.0578 ZX= -6.1606
XY= -2.4098 YY= 123.8874 ZY= -1.0673
XZ= 5.2212 YZ= -4.9176 ZZ= 131.0312
Eigenvalues: 119.9773 131.8153 148.4520
15 C Isotropic = 134.4845 Anisotropy = 22.0671
XX= 149.1363 YX= 5.6068 ZX= 4.3386
XY= -5.9093 YY= 135.9540 ZY= 2.0670
XZ= -6.8931 YZ= 4.7241 ZZ= 118.3631
Eigenvalues: 117.6824 136.5751 149.1959
16 C Isotropic = 163.3058 Anisotropy = 24.2785
XX= 176.7016 YX= -9.1957 ZX= 1.3687
XY= -4.5847 YY= 162.1465 ZY= 3.5849
XZ= 3.7949 YZ= 3.3213 ZZ= 151.0694
Eigenvalues: 149.3102 161.1158 179.4915
17 C Isotropic = 147.4763 Anisotropy = 35.9089
XX= 143.1726 YX= -0.6513 ZX= 6.5327
XY= -2.6572 YY= 171.2367 ZY= 0.6870
XZ= -0.5817 YZ= 3.4200 ZZ= 128.0195
Eigenvalues: 127.3328 143.6805 171.4156
18 C Isotropic = -47.0653 Anisotropy = 175.0531
XX= -81.9312 YX= 9.3170 ZX= -41.7944
XY= 2.8263 YY= -118.7044 ZY= -2.9491
XZ= -36.6070 YZ= -0.5412 ZZ= 59.4397
Eigenvalues: -119.8255 -91.0071 69.6368
19 C Isotropic = 173.2513 Anisotropy = 23.9976
XX= 162.8696 YX= 7.5602 ZX= -4.0048
XY= 8.6510 YY= 169.7931 ZY= -5.4967
XZ= -3.6792 YZ= -2.5588 ZZ= 187.0913
Eigenvalues: 157.4826 173.0218 189.2497
20 O Isotropic = -232.6820 Anisotropy = 901.0914
XX= -475.3962 YX= -182.2073 ZX= -235.6484
XY= -181.2861 YY= -520.3551 ZY= -61.6810
XZ= -250.0246 YZ= -80.9610 ZZ= 297.7054
Eigenvalues: -727.7796 -338.3119 368.0456
21 H Isotropic = 27.4625 Anisotropy = 13.4963
XX= 29.6081 YX= 5.4469 ZX= 2.1659
XY= 2.8061 YY= 32.9926 ZY= 2.5738
XZ= 1.9239 YZ= 2.9078 ZZ= 19.7869
Eigenvalues: 19.0836 26.8439 36.4601
22 H Isotropic = 24.2284 Anisotropy = 11.3193
XX= 30.6896 YX= -3.5869 ZX= -0.3416
XY= -2.5042 YY= 23.1360 ZY= 0.5445
XZ= -0.1466 YZ= 0.2416 ZZ= 18.8597
Eigenvalues: 18.8238 22.0869 31.7746

Eigenvalues: -37.1014 18.9809 169.0287
12 C Isotropic = 156.0338 Anisotropy = 13.5188
XX= 158.4355 YX= 6.7478 ZX= -0.8131
XY= 4.7529 YY= 157.5489 ZY= -5.8385
XZ= -2.1190 YZ= -2.8278 ZZ= 152.1169
Eigenvalues: 149.3421 153.7129 165.0463
13 C Isotropic = 152.6552 Anisotropy = 27.6541
XX= 164.7842 YX= -5.7329 ZX= 7.8254
XY= -14.1986 YY= 148.5242 ZY= -0.2350
XZ= 7.2632 YZ= 2.2834 ZZ= 144.6572
Eigenvalues: 139.3826 147.4917 171.0912
14 C Isotropic = 129.9474 Anisotropy = 26.4540
XX= 135.6988 YX= -5.6551 ZX= -8.7193
XY= -2.0490 YY= 119.4827 ZY= 3.9376
XZ= -8.7045 YZ= 16.9117 ZZ= 134.6608
Eigenvalues: 114.1624 128.0965 147.5834
15 C Isotropic = 134.5288 Anisotropy = 18.9576
XX= 134.6859 YX= 11.2229 ZX= -10.3173
XY= 11.0602 YY= 136.8255 ZY= 2.5946
XZ= -0.3009 YZ= 2.3741 ZZ= 132.0751
Eigenvalues: 121.5496 134.8697 147.1672
16 C Isotropic = 164.1691 Anisotropy = 26.6708
XX= 159.8035 YX= -8.9897 ZX= -8.9244
XY= -4.7871 YY= 163.5529 ZY= 4.2631
XZ= -14.5226 YZ= 9.1473 ZZ= 169.1509
Eigenvalues: 151.4229 159.1347 181.9496
17 C Isotropic = 149.4047 Anisotropy = 42.1581
XX= 132.4064 YX= -3.9523 ZX= -7.7852
XY= -8.3139 YY= 176.5228 ZY= 5.3294
XZ= -0.8456 YZ= -1.6871 ZZ= 139.2849
Eigenvalues: 129.8301 140.8738 177.5101
18 C Isotropic = -48.3586 Anisotropy = 178.2581
XX= 45.3683 YX= 0.3121 ZX= 60.9205
XY= -4.1502 YY= -119.0875 ZY= -1.0566
XZ= 58.2626 YZ= -7.7492 ZZ= -71.3565
Eigenvalues: -119.6244 -95.9314 70.4802
19 C Isotropic = 160.4044 Anisotropy = 42.6993
XX= 173.4425 YX= 9.0098 ZX= 16.5146
XY= 10.6564 YY= 143.5015 ZY= -1.6907
XZ= 20.0928 YZ= -0.3945 ZZ= 164.2692
Eigenvalues: 138.4010 153.9416 188.8706
20 O Isotropic = -240.0027 Anisotropy = 921.5376
XX= 248.6591 YX= -63.9965 ZX= 303.6572
XY= -87.5573 YY= -546.7220 ZY= 173.2933
XZ= 329.0567 YZ= 190.9178 ZZ= -421.9452
Eigenvalues: -744.3979 -349.9659 374.3557
21 H Isotropic = 27.4674 Anisotropy = 13.5064
XX= 29.4233 YX= 5.4482 ZX= 2.4948
XY= 2.7663 YY= 33.1471 ZY= 2.0845
XZ= 2.4098 YZ= 2.7255 ZZ= 19.8319
Eigenvalues: 19.0855 26.8451 36.4717
22 H Isotropic = 24.2323 Anisotropy = 11.0873
XX= 30.3768 YX= -3.7514 ZX= 0.1905
XY= -2.6402 YY= 23.2861 ZY= -0.0205
XZ= 0.6720 YZ= -0.4794 ZZ= 19.0340
Eigenvalues: 19.0127 22.0604 31.6238

23 H Isotropic = 24.8253 Anisotropy = 5.3010
XX= 27.7307 YX= 1.5196 ZX= 0.9013
XY= 0.7369 YY= 25.4817 ZY= 0.7147
XZ= 0.7374 YZ= 0.8779 ZZ= 21.2635
Eigenvalues: 21.0606 25.0560 28.3593
24 H Isotropic = 24.9854 Anisotropy = 8.2953
XX= 29.6008 YX= -2.2454 ZX= 0.5686
XY= -2.4138 YY= 24.4702 ZY= 0.1747
XZ= 0.3256 YZ= 0.0364 ZZ= 20.8853
Eigenvalues: 20.8457 23.5949 30.5156
25 H Isotropic = 28.9172 Anisotropy = 9.6040
XX= 32.0413 YX= 3.0506 ZX= -0.6074
XY= 3.4750 YY= 31.1225 ZY= -1.9842
XZ= -0.3813 YZ= -3.6890 ZZ= 23.5878
Eigenvalues: 22.6009 28.8308 35.3199
26 H Isotropic = 28.8721 Anisotropy = 7.7042
XX= 29.4816 YX= -0.2970 ZX= 2.1530
XY= 0.7009 YY= 27.7489 ZY= 4.0443
XZ= 0.9085 YZ= 5.9495 ZZ= 29.3857
Eigenvalues: 23.3857 29.2223 34.0082
27 H Isotropic = 29.8462 Anisotropy = 7.8423
XX= 32.0831 YX= -1.1276 ZX= -0.2621
XY= -1.6327 YY= 34.3213 ZY= 0.9419
XZ= -0.3894 YZ= 1.1112 ZZ= 23.1342
Eigenvalues: 23.0363 31.4279 35.0744
28 H Isotropic = 30.4286 Anisotropy = 6.9034
XX= 29.5753 YX= 1.6310 ZX= 0.2641
XY= 1.1470 YY= 28.7423 ZY= -3.2149
XZ= 0.9160 YZ= -3.9740 ZZ= 32.9683
Eigenvalues: 26.0483 30.2067 35.0309
29 H Isotropic = 30.1763 Anisotropy = 4.4999
XX= 31.0185 YX= 2.6881 ZX= -0.2672
XY= 2.7416 YY= 28.4370 ZY= -0.2351
XZ= -2.0845 YZ= 0.4119 ZZ= 31.0735
Eigenvalues: 26.6073 30.7454 33.1763
30 H Isotropic = 29.5020 Anisotropy = 2.8006
XX= 29.3811 YX= 0.6778 ZX= -1.7452
XY= -0.2972 YY= 27.8592 ZY= 0.6154
XZ= 1.2112 YZ= 0.4061 ZZ= 31.2658
Eigenvalues: 27.7523 29.3847 31.3691
31 H Isotropic = 30.4056 Anisotropy = 5.6241
XX= 31.1737 YX= -1.1668 ZX= 1.4437
XY= -1.7313 YY= 29.1949 ZY= -3.5232
XZ= -1.0464 YZ= -3.7715 ZZ= 30.8483
Eigenvalues: 26.0764 30.9855 34.1550
32 H Isotropic = 29.4255 Anisotropy = 10.7026
XX= 32.0131 YX= -2.6194 ZX= -1.4321
XY= -3.1569 YY= 33.3985 ZY= 3.2894
XZ= -1.5422 YZ= 3.1976 ZZ= 22.8649
Eigenvalues: 21.9017 29.8142 36.5606
33 H Isotropic = 30.3962 Anisotropy = 8.2898
XX= 29.4561 YX= 0.4261 ZX= 1.7953
XY= 0.6344 YY= 29.0628 ZY= 4.2751
XZ= 1.6559 YZ= 4.1680 ZZ= 32.6696
Eigenvalues: 26.1879 29.0779 35.9227

23 H Isotropic = 24.8694 Anisotropy = 5.2426
XX= 27.6845 YX= 1.4525 ZX= 1.2144
XY= 0.6532 YY= 25.4785 ZY= 0.5035
XZ= 1.1855 YZ= 0.6928 ZZ= 21.4453
Eigenvalues: 21.1842 25.0596 28.3645
24 H Isotropic = 24.9978 Anisotropy = 8.2497
XX= 29.4617 YX= -2.2198 ZX= 1.2363
XY= -2.4129 YY= 24.4286 ZY= -0.1427
XZ= 1.0207 YZ= -0.2011 ZZ= 21.1032
Eigenvalues: 20.9472 23.5486 30.4976
25 H Isotropic = 28.9563 Anisotropy = 9.8057
XX= 32.2865 YX= 3.4326 ZX= -0.1275
XY= 3.6762 YY= 30.9208 ZY= -1.8985
XZ= 0.2118 YZ= -3.6690 ZZ= 23.6617
Eigenvalues: 22.5532 28.8223 35.4935
26 H Isotropic = 28.9688 Anisotropy = 7.4888
XX= 29.4053 YX= -0.4702 ZX= 1.8892
XY= 0.3545 YY= 28.2995 ZY= 4.0326
XZ= 0.7059 YZ= 5.9710 ZZ= 29.2016
Eigenvalues: 23.5816 29.3634 33.9613
27 H Isotropic = 29.6894 Anisotropy = 7.1840
XX= 31.9629 YX= -0.8203 ZX= -0.1664
XY= -1.6275 YY= 33.8829 ZY= -0.2383
XZ= -0.1282 YZ= 0.2200 ZZ= 23.2223
Eigenvalues: 23.2197 31.3697 34.4787
28 H Isotropic = 30.5373 Anisotropy = 6.9494
XX= 29.3951 YX= 2.2389 ZX= -0.6856
XY= 1.3122 YY= 28.3948 ZY= -2.6336
XZ= 0.2932 YZ= -3.0278 ZZ= 33.8219
Eigenvalues: 26.3640 30.0775 35.1702
29 H Isotropic = 30.8234 Anisotropy = 3.4058
XX= 32.8898 YX= 1.2100 ZX= -0.2384
XY= 0.2516 YY= 28.3567 ZY= 1.1537
XZ= -0.9251 YZ= 1.6667 ZZ= 31.2239
Eigenvalues: 27.6261 31.7503 33.0940
30 H Isotropic = 29.5236 Anisotropy = 2.8425
XX= 30.2860 YX= 0.5267 ZX= -1.5320
XY= -0.6531 YY= 27.5750 ZY= 1.4971
XZ= 1.5689 YZ= 1.8038 ZZ= 30.7098
Eigenvalues: 26.8650 30.2872 31.4186
31 H Isotropic = 31.0489 Anisotropy = 5.3881
XX= 31.1175 YX= -0.2965 ZX= 1.3140
XY= -0.2169 YY= 29.0017 ZY= -2.7312
XZ= -0.6698 YZ= -3.1894 ZZ= 33.0276
Eigenvalues: 27.4332 31.0726 34.6410
32 H Isotropic = 29.5819 Anisotropy = 10.8501
XX= 32.7697 YX= -3.2835 ZX= 0.2530
XY= -3.5723 YY= 33.9098 ZY= 0.3162
XZ= -0.2051 YZ= -0.0275 ZZ= 22.0663
Eigenvalues: 22.0641 29.8664 36.8153
33 H Isotropic = 30.3948 Anisotropy = 7.1724
XX= 30.1185 YX= -1.0531 ZX= 1.7848
XY= -0.4767 YY= 29.9533 ZY= 4.5628
XZ= 1.1809 YZ= 4.4962 ZZ= 31.1127
Eigenvalues: 25.4456 30.5624 35.1764

34 H Isotropic = 29.9512 Anisotropy = 9.5595
XX= 30.0072 YX= 2.6269 ZX= -0.7840
XY= 3.4236 YY= 34.7876 ZY= -0.5039
XZ= -1.0598 YZ= -0.5899 ZZ= 25.0587
Eigenvalues: 24.8926 28.6368 36.3242
35 H Isotropic = 30.2088 Anisotropy = 2.7356
XX= 29.8120 YX= -1.1691 ZX= 1.3515
XY= -1.8880 YY= 29.0100 ZY= -0.0106
XZ= -0.7731 YZ= -0.8165 ZZ= 31.8045
Eigenvalues: 27.8249 30.7690 32.0326
36 H Isotropic = 29.7898 Anisotropy = 8.9657
XX= 29.6973 YX= -0.8262 ZX= 0.1890
XY= -0.0747 YY= 33.9217 ZY= -3.8979
XZ= 0.1750 YZ= -4.5929 ZZ= 25.7506
Eigenvalues: 23.9442 29.6584 35.7670
37 H Isotropic = 30.1342 Anisotropy = 7.2349
XX= 28.7773 YX= -1.2486 ZX= -0.8472
XY= -0.9227 YY= 29.9544 ZY= 3.5806
XZ= -0.5036 YZ= 4.0463 ZZ= 31.6708
Eigenvalues: 26.8026 28.6425 34.9575
38 H Isotropic = 29.5999 Anisotropy = 9.1653
XX= 31.2012 YX= 0.3418 ZX= 4.4196
XY= -0.8718 YY= 27.4490 ZY= -2.1089
XZ= 4.9513 YZ= -2.1168 ZZ= 30.1494
Eigenvalues: 25.0504 28.0391 35.7101
39 H Isotropic = 29.2856 Anisotropy = 9.0499
XX= 33.8881 YX= -1.4680 ZX= -2.2289
XY= -2.1410 YY= 26.8636 ZY= 1.3687
XZ= -2.8641 YZ= 1.7476 ZZ= 27.1052
Eigenvalues: 25.3915 27.1465 35.3189
40 H Isotropic = 30.8841 Anisotropy = 8.8330
XX= 70.5817 YX= -0.9397 ZX= -4.1478
XY= -1.2145 YY= 68.0288 ZY= -1.6398
XZ= -3.0411 YZ= -0.3440 ZZ= 77.0447
Eigenvalues: 25.5115 30.3681 36.7728
41 H Isotropic = 30.5028 Anisotropy = 5.5433
XX= 70.5817 YX= -0.9397 ZX= -4.1478
XY= -1.2145 YY= 68.0288 ZY= -1.6398
XZ= -3.0411 YZ= -0.3440 ZZ= 77.0447
Eigenvalues: 25.9005 31.4096 34.1984
42 H Isotropic = 31.4942 Anisotropy = 11.2796
XX= 70.5817 YX= -0.9397 ZX= -4.1478
XY= -1.2145 YY= 68.0288 ZY= -1.6398
XZ= -3.0411 YZ= -0.3440 ZZ= 77.0447
Eigenvalues: 27.1369 28.3319 39.0140

34 H Isotropic = 29.5188 Anisotropy = 8.1542
XX= 30.2956 YX= 2.5756 ZX= -0.8575
XY= 2.7888 YY= 32.7387 ZY= -2.2040
XZ= -0.9463 YZ= -1.7466 ZZ= 25.5220
Eigenvalues: 25.0066 28.5948 34.9549
35 H Isotropic = 30.0041 Anisotropy = 4.0707
XX= 30.1041 YX= 1.1001 ZX= 0.7321
XY= 0.3954 YY= 27.6909 ZY= -0.8373
XZ= 1.3953 YZ= -0.9137 ZZ= 32.2172
Eigenvalues: 27.2109 30.0834 32.7178
36 H Isotropic = 29.7735 Anisotropy = 7.5848
XX= 29.9952 YX= 1.6211 ZX= -1.7647
XY= 1.8253 YY= 34.1561 ZY= 0.8341
XZ= -1.5678 YZ= 1.8269 ZZ= 25.1692
Eigenvalues: 24.3181 30.1724 34.8300
37 H Isotropic = 29.6877 Anisotropy = 10.1996
XX= 31.9557 YX= -4.8675 ZX= 3.4769
XY= -4.0795 YY= 29.0178 ZY= -1.4972
XZ= 2.7122 YZ= -1.1546 ZZ= 28.0896
Eigenvalues: 25.4260 27.1496 36.4874
38 H Isotropic = 29.5281 Anisotropy = 7.3582
XX= 27.8117 YX= 1.4811 ZX= 0.6925
XY= 0.9424 YY= 26.6069 ZY= 1.5294
XZ= -0.1312 YZ= 1.1585 ZZ= 34.1657
Eigenvalues: 25.7342 28.4166 34.4336
39 H Isotropic = 29.4052 Anisotropy = 9.4264
XX= 32.3050 YX= -2.1419 ZX= -4.3955
XY= -2.8171 YY= 27.2074 ZY= 1.0819
XZ= -3.9663 YZ= -0.2391 ZZ= 28.7033
Eigenvalues: 25.2371 27.2891 35.6895
40 H Isotropic = 30.9823 Anisotropy = 8.8193
XX= 29.2699 YX= 0.7381 ZX= 3.0169
XY= 0.7484 YY= 27.6732 ZY= 1.0543
XZ= 1.5541 YZ= 0.9879 ZZ= 36.0039
Eigenvalues: 27.3785 28.7065 36.8619
41 H Isotropic = 30.4620 Anisotropy = 9.3510
XX= 35.2213 YX= -3.2176 ZX= 3.5591
XY= -1.2642 YY= 29.8143 ZY= 1.6663
XZ= 2.6662 YZ= 0.5474 ZZ= 26.3504
Eigenvalues: 24.7419 29.9481 36.6960
42 H Isotropic = 31.0259 Anisotropy = 10.4256
XX= 33.7717 YX= 5.8527 ZX= 1.5105
XY= 3.9716 YY= 31.9724 ZY= -0.3131
XZ= 1.6316 YZ= -0.0923 ZZ= 27.3336
Eigenvalues: 26.3500 28.7514 37.9763

Chemical Shielding tensors (GIAO MPW1PW91/6-311+G(2d,p) IEFPCM = DMSO), 3R*,7S*,11R*-1b

Conf. A (17 %)

```
   1  C    Isotropic =    72.8410   Anisotropy =   131.1345
XX=    71.7102   YX=   -14.1267   ZX=    58.3838
XY=     2.1034   YY=    45.4428   ZY=    53.7820
XZ=    47.8844   YZ=    64.6182   ZZ=   101.3700
Eigenvalues:    -8.4851    66.7441   160.2640
   2  C    Isotropic =    27.4854   Anisotropy =   128.3085
XX=   -14.2758   YX=    30.9418   ZX=    59.9935
XY=    33.6347   YY=    28.0072   ZY=    20.4759
XZ=    59.3216   YZ=    20.1272   ZZ=    68.7250
Eigenvalues:   -50.6183    20.0501   113.0245
   3  C    Isotropic =    67.4605   Anisotropy =   126.5354
XX=    73.3093   YX=    47.5154   ZX=    24.9060
XY=    41.0577   YY=    16.3607   ZY=    40.0440
XZ=    33.4716   YZ=    38.7626   ZZ=   112.7116
Eigenvalues:   -11.8039    62.3679   151.8174
   4  C    Isotropic =    60.7801   Anisotropy =   169.3561
XX=    44.4838   YX=     8.3529   ZX=    72.8624
XY=    10.2724   YY=    28.4878   ZY=    61.1860
XZ=    53.3228   YZ=    68.8927   ZZ=   109.3688
Eigenvalues:   -19.5809    28.2371   173.6841
   5  C    Isotropic =    34.4257   Anisotropy =   118.3157
XX=    -9.7861   YX=    32.3777   ZX=    58.3827
XY=    33.4686   YY=    44.0136   ZY=    13.7861
XZ=    55.7150   YZ=    21.3418   ZZ=    69.0497
Eigenvalues:   -45.0567    35.0309   113.3029
   6  C    Isotropic =    53.7832   Anisotropy =   164.8183
XX=    48.3575   YX=    45.1070   ZX=    46.9923
XY=    40.6753   YY=     1.7571   ZY=    53.3644
XZ=    50.8701   YZ=    46.1904   ZZ=   111.2350
Eigenvalues:   -27.1392    24.8267   163.6620
   7  C    Isotropic =   160.5742   Anisotropy =    19.4919
XX=   165.3246   YX=     4.7748   ZX=    -4.2461
XY=     7.6666   YY=   158.3084   ZY=    -3.5995
XZ=   -12.0818   YZ=    -1.7590   ZZ=   158.0895
Eigenvalues:   152.3981   155.7557   173.5688
   8  C    Isotropic =   153.7030   Anisotropy =    10.8588
XX=   147.8749   YX=    -3.9355   ZX=     4.4082
XY=     2.3711   YY=   158.7913   ZY=     8.3779
XZ=    -2.2610   YZ=    -0.9037   ZZ=   154.4428
Eigenvalues:   147.5088   152.6579   160.9422
   9  C    Isotropic =   103.9349   Anisotropy =    47.6852
XX=   103.2462   YX=    11.2493   ZX=   -13.7579
XY=    14.2731   YY=    92.4558   ZY=   -18.1343
XZ=    -8.6864   YZ=   -22.1317   ZZ=   116.1026
Eigenvalues:    79.3041    96.7755   135.7250
  10  O    Isotropic =   189.5275   Anisotropy =   101.1327
XX=   192.1146   YX=    -2.3982   ZX=    39.7905
XY=    -1.2827   YY=   161.9670   ZY=    47.9327
XZ=    54.7688   YZ=     9.7656   ZZ=   214.5008
Eigenvalues:   136.9294   174.7037   256.9493
  11  C    Isotropic =   144.8231   Anisotropy =    42.0775
XX=   147.7598   YX=   -17.3028   ZX=    -7.4844
XY=    -7.6221   YY=   164.3518   ZY=     1.6979
XZ=   -12.6974   YZ=     9.1368   ZZ=   122.3578
Eigenvalues:   118.8116   142.7830   172.8748
  12  C    Isotropic =    42.2015   Anisotropy =   143.4627
XX=    56.0197   YX=   -70.5480   ZX=   -66.3756
XY=   -65.8559   YY=    14.8007   ZY=    -0.1180
XZ=   -56.1280   YZ=    -4.0791   ZZ=    55.7840
Eigenvalues:   -51.5574    40.3185   137.8433
  13  C    Isotropic =    25.2572   Anisotropy =   181.4030
XX=    41.9351   YX=   -90.4642   ZX=   -64.6414
XY=   -86.4608   YY=     9.5547   ZY=     7.9098
XZ=   -82.9881   YZ=    -2.1951   ZZ=    24.2819
Eigenvalues:   -85.9669    15.5460   146.1926
  14  C    Isotropic =   134.8075   Anisotropy =    34.7297
XX=   155.1712   YX=    -0.6979   ZX=    12.4229
XY=    -3.9164   YY=   116.8285   ZY=     5.6408
XZ=     4.4549   YZ=     7.2167   ZZ=   132.4229
Eigenvalues:   113.8703   132.5916   157.9607
  15  C    Isotropic =   166.5047   Anisotropy =    25.9204
XX=   163.8157   YX=    -6.9936   ZX=    -2.1059
XY=    -7.3209   YY=   179.2756   ZY=    -7.2280
XZ=     0.4912   YZ=    -7.9291   ZZ=   156.4228
Eigenvalues:   153.2449   162.4843   183.7850
```

```
  16  O    Isotropic =   235.6240   Anisotropy =    77.7286
XX=   265.5237   YX=   -24.3318   ZX=    17.0982
XY=   -58.0532   YY=   207.0518   ZY=    66.2266
XZ=    32.2705   YZ=    43.9311   ZZ=   234.2965
Eigenvalues:   145.0463   274.3826   287.4431
  17  C    Isotropic =   132.1010   Anisotropy =    72.5497
XX=   131.6444   YX=    27.2740   ZX=   -24.7303
XY=    23.7501   YY=   128.0296   ZY=   -21.9734
XZ=   -22.9317   YZ=   -24.0996   ZZ=   136.6291
Eigenvalues:   104.2270   111.6087   180.4675
  18  C    Isotropic =   110.7369   Anisotropy =    44.5581
XX=   132.3339   YX=    -0.5792   ZX=    18.0387
XY=     3.3276   YY=    99.8967   ZY=    -3.7160
XZ=    18.1805   YZ=    -4.0011   ZZ=    99.9799
Eigenvalues:    90.1612   101.6071   140.4423
  19  C    Isotropic =   153.1834   Anisotropy =    49.8408
XX=   143.7681   YX=    16.6380   ZX=    -7.1601
XY=    15.8424   YY=   171.2146   ZY=   -16.6257
XZ=    -8.6849   YZ=   -15.4859   ZZ=   144.5674
Eigenvalues:   136.0507   137.0889   186.4106
  20  O    Isotropic =   242.9619   Anisotropy =    52.6081
XX=   237.1525   YX=    -9.6688   ZX=    18.6419
XY=     1.5283   YY=   231.6416   ZY=    17.5406
XZ=    16.6016   YZ=    29.5945   ZZ=   260.0917
Eigenvalues:   211.7275   239.1244   278.0340
  21  C    Isotropic =   159.1455   Anisotropy =    35.2562
XX=   153.5243   YX=    -4.6623   ZX=    -1.6584
XY=     1.9698   YY=   141.6118   ZY=     5.2317
XZ=     2.4194   YZ=     2.3137   ZZ=   182.3006
Eigenvalues:   141.1125   153.6745   182.6497
  22  C    Isotropic =   -30.7256   Anisotropy =   192.1434
XX=    20.8630   YX=   -35.0668   ZX=   -96.3207
XY=   -47.6230   YY=   -71.4364   ZY=    15.1614
XZ=   -88.6833   YZ=    10.9387   ZZ=   -41.6033
Eigenvalues:  -113.4418   -76.1050    97.3700
  23  C    Isotropic =   126.7900   Anisotropy =    29.3439
XX=   128.4605   YX=    21.2022   ZX=     4.4061
XY=    14.0345   YY=   127.1530   ZY=    -1.5066
XZ=    -5.2208   YZ=   -10.3329   ZZ=   124.7566
Eigenvalues:   109.1269   124.8905   146.3526
  24  C    Isotropic =   136.1764   Anisotropy =    25.4856
XX=   124.7219   YX=    14.9166   ZX=     5.9214
XY=    16.4774   YY=   136.0583   ZY=    -2.8160
XZ=    15.5528   YZ=    -2.2435   ZZ=   147.7490
Eigenvalues:   110.8245   144.5379   153.1668
  25  C    Isotropic =   136.5891   Anisotropy =    37.7038
XX=   151.6437   YX=     9.8882   ZX=     3.0555
XY=    19.8195   YY=   130.3587   ZY=     9.5802
XZ=     7.6931   YZ=     8.9223   ZZ=   127.7651
Eigenvalues:   118.2847   129.7577   161.7250
  26  C    Isotropic =   154.1683   Anisotropy =    32.9240
XX=   161.8624   YX=    13.4032   ZX=    15.0682
XY=    22.2477   YY=   147.7940   ZY=    -5.1066
XZ=     8.6615   YZ=    -6.5865   ZZ=   152.8484
Eigenvalues:   129.6969   156.6902   176.1176
  27  C    Isotropic =   164.6931   Anisotropy =    28.3263
XX=   154.4269   YX=    15.9564   ZX=    12.6043
XY=    17.0870   YY=   164.5828   ZY=   -13.3507
XZ=    12.4144   YZ=   -11.8266   ZZ=   175.0695
Eigenvalues:   134.5869   175.9150   183.5772
  28  C    Isotropic =   148.7585   Anisotropy =    44.2751
XX=   137.8460   YX=    19.6935   ZX=    15.1327
XY=    17.4514   YY=   165.7841   ZY=     0.8953
XZ=    13.1134   YZ=     8.1779   ZZ=   142.6455
Eigenvalues:   122.3938   145.6065   178.2753
  29  C    Isotropic =   164.4079   Anisotropy =    38.9214
XX=   164.9550   YX=   -15.2767   ZX=   -15.5062
XY=   -17.0387   YY=   164.5200   ZY=     6.5738
XZ=   -14.8496   YZ=     7.1517   ZZ=   163.7488
Eigenvalues:   145.6072   157.2610   190.3555
  30  C    Isotropic =   163.7135   Anisotropy =    35.7236
XX=   178.7578   YX=   -12.7544   ZX=    -2.3703
XY=   -16.7664   YY=   152.3575   ZY=     9.4703
XZ=    -3.9553   YZ=    12.8951   ZZ=   160.0252
Eigenvalues:   141.2235   162.3878   187.5292
  31  O    Isotropic =  -253.8998   Anisotropy =   905.3571
XX=    52.7493   YX=   -53.1220   ZX=  -417.1311
XY=   -69.0788   YY=  -422.8521   ZY=   276.6076
XZ=  -399.6525   YZ=   294.1151   ZZ=  -391.5967
```

```
Eigenvalues:  -778.1578  -333.2133   349.6716
  32  C    Isotropic =   161.8703   Anisotropy =    39.8286
XX=   150.3192   YX=    10.3129   ZX=    16.4556
XY=     9.0238   YY=   155.3819   ZY=     4.1091
XZ=    14.1331   YZ=     5.1718   ZZ=   179.9098
Eigenvalues:   140.1899   156.9984   188.4227
  33  H    Isotropic =    25.1229   Anisotropy =     9.6465
XX=    28.3066   YX=    -4.0354   ZX=    -2.4137
XY=    -4.0782   YY=    25.5146   ZY=    -0.1711
XZ=    -2.4330   YZ=    -0.2399   ZZ=    21.5476
Eigenvalues:    20.2074    23.6074    31.5540
  34  H    Isotropic =    24.9508   Anisotropy =     8.3936
XX=    27.8386   YX=     0.0426   ZX=    -3.9993
XY=    -0.0007   YY=    23.4325   ZY=    -1.5186
XZ=    -4.5247   YZ=    -1.1208   ZZ=    23.5813
Eigenvalues:    20.5150    23.7909    30.5466
  35  H    Isotropic =    29.0290   Anisotropy =     9.2761
XX=    29.0757   YX=    -1.0844   ZX=    -2.2682
XY=    -2.4164   YY=    34.1722   ZY=     1.1603
XZ=    -1.9314   YZ=     2.4464   ZZ=    23.8390
Eigenvalues:    22.9808    28.8932    35.2130
  36  H    Isotropic =    28.8439   Anisotropy =     8.3178
XX=    29.3947   YX=    -3.0300   ZX=     2.6286
XY=    -3.1209   YY=    26.1684   ZY=    -1.9917
XZ=     1.3399   YZ=    -3.7263   ZZ=    30.9687
Eigenvalues:    23.9510    28.1916    34.3891
  37  H    Isotropic =    29.8880   Anisotropy =    10.5202
XX=    34.8871   YX=    -0.6727   ZX=     4.0563
XY=     0.6692   YY=    28.3747   ZY=     2.5002
XZ=     4.6502   YZ=     3.6092   ZZ=    26.4022
Eigenvalues:    23.0487    29.7138    36.9015
  38  H    Isotropic =    30.1006   Anisotropy =     6.4424
XX=    31.6639   YX=     0.8071   ZX=    -0.9510
XY=     1.4262   YY=    30.7974   ZY=    -3.5422
XZ=    -1.8975   YZ=    -3.8399   ZZ=    27.8405
Eigenvalues:    25.2917    30.6145    34.3955
  39  H    Isotropic =    28.6261   Anisotropy =    10.9171
XX=    33.9341   YX=    -2.7851   ZX=     2.6204
XY=    -2.7154   YY=    28.4066   ZY=    -2.9611
XZ=     0.9832   YZ=    -4.5313   ZZ=    23.5374
Eigenvalues:    21.4976    28.4764    35.9041
  40  H    Isotropic =    29.0792   Anisotropy =     2.5837
XX=    28.5765   YX=     1.5281   ZX=    -2.2687
XY=    -0.5536   YY=    29.0590   ZY=     0.3410
XZ=    -0.5499   YZ=    -1.1409   ZZ=    29.6022
Eigenvalues:    27.5686    28.8674    30.8017
  41  H    Isotropic =    29.7594   Anisotropy =     3.8642
XX=    31.7308   YX=     0.9042   ZX=    -0.3188
XY=     1.6820   YY=    26.1384   ZY=     0.9313
XZ=    -0.7967   YZ=    -0.9246   ZZ=    31.4090
Eigenvalues:    25.8511    31.0915    32.3355
  42  H    Isotropic =    28.1072   Anisotropy =     7.7140
XX=    27.5124   YX=    -3.5147   ZX=     2.2232
XY=    -2.7560   YY=    30.8812   ZY=    -0.9041
XZ=     3.3686   YZ=     0.0076   ZZ=    25.9281
Eigenvalues:    23.4354    27.6364    33.2499
  43  H    Isotropic =    29.9133   Anisotropy =     8.7892
XX=    31.1836   YX=     0.6022   ZX=    -3.3669
XY=     1.9708   YY=    31.4155   ZY=    -4.2235
XZ=    -4.0509   YZ=    -3.1878   ZZ=    27.1406
Eigenvalues:    23.9550    30.0121    35.7727
  44  H    Isotropic =    29.4024   Anisotropy =     9.2918
XX=    34.8085   YX=    -0.9254   ZX=     1.3515
XY=    -1.1867   YY=    26.0857   ZY=    -2.3949
XZ=     2.3376   YZ=    -4.0007   ZZ=    27.3130
Eigenvalues:    23.4311    29.1793    35.5969
  45  H    Isotropic =    29.3591   Anisotropy =     5.8379
XX=    27.9840   YX=     0.4948   ZX=    -2.6619
XY=    -1.4487   YY=    31.6852   ZY=     1.9168
XZ=    -3.1655   YZ=     1.9917   ZZ=    28.4082
Eigenvalues:    25.1156    29.7106    33.2511
  46  H    Isotropic =    27.9207   Anisotropy =     9.0586
XX=    31.5218   YX=    -1.5895   ZX=    -3.9161
XY=     0.0929   YY=    26.0191   ZY=    -1.3750
XZ=    -4.7560   YZ=    -0.3801   ZZ=    26.2212
Eigenvalues:    23.3154    26.4869    33.9598
  47  H    Isotropic =    28.1939   Anisotropy =     8.0207
XX=    26.3650   YX=     0.8003   ZX=    -2.5643
XY=    -1.2502   YY=    26.1207   ZY=    -2.4310
```

```
XZ=    -1.5038   YZ=    -2.7717   ZZ=    32.0959
Eigenvalues:    24.5482    26.4924    33.5410
  48  H    Isotropic =    28.1769   Anisotropy =     8.2952
XX=    30.1133   YX=     4.3548   ZX=    -1.2085
XY=     2.8529   YY=    29.8377   ZY=     0.2981
XZ=    -0.6880   YZ=    -1.4073   ZZ=    24.5796
Eigenvalues:    24.4209    26.4026    33.7070
  49  H    Isotropic =    30.5115   Anisotropy =     7.3727
XX=    27.6488   YX=     0.4927   ZX=    -1.4443
XY=     0.4760   YY=    30.6106   ZY=    -4.0478
XZ=    -0.6062   YZ=    -2.0374   ZZ=    33.2751
Eigenvalues:    27.4661    28.6418    35.4266
  50  H    Isotropic =    30.3703   Anisotropy =     9.1824
XX=    27.4318   YX=    -1.0703   ZX=    -0.6727
XY=    -2.5340   YY=    36.1106   ZY=    -0.1664
XZ=    -0.3288   YZ=    -0.9358   ZZ=    27.5686
Eigenvalues:    26.6661    27.9529    36.4919
  51  H    Isotropic =    30.5236   Anisotropy =     7.6111
XX=    33.5182   YX=     2.5301   ZX=     0.8675
XY=     3.3473   YY=    31.4426   ZY=    -0.4720
XZ=     0.3267   YZ=    -0.9464   ZZ=    26.6100
Eigenvalues:    26.3282    29.6450    35.5977
  52  H    Isotropic =    28.6311   Anisotropy =    18.9366
XX=    23.3356   YX=    -1.4662   ZX=     5.0793
XY=     0.4262   YY=    29.5665   ZY=     7.8688
XZ=     3.8546   YZ=    10.6636   ZZ=    32.9913
Eigenvalues:    19.0756    25.5623    41.2555
  53  H    Isotropic =    30.5465   Anisotropy =     4.7275
XX=    27.9676   YX=    -0.4697   ZX=    -2.4011
XY=     0.2390   YY=    33.0129   ZY=    -0.5978
XZ=    -2.3679   YZ=    -1.8628   ZZ=    30.6591
Eigenvalues:    26.4935    31.4479    33.6982
  54  H    Isotropic =    30.9467   Anisotropy =     6.6791
XX=    30.7709   YX=    -0.1585   ZX=     3.0497
XY=     0.5000   YY=    30.0609   ZY=     1.5156
XZ=     4.2332   YZ=     1.5665   ZZ=    32.0082
Eigenvalues:    27.3918    30.0487    35.3994
  55  H    Isotropic =    30.5452   Anisotropy =     9.0334
XX=    29.3830   YX=    -4.2954   ZX=    -2.0865
XY=    -2.8925   YY=    32.0665   ZY=     2.2173
XZ=    -2.4777   YZ=     3.3201   ZZ=    30.1861
Eigenvalues:    26.8368    28.2314    36.5675
  56  H    Isotropic =    28.8108   Anisotropy =     8.6735
XX=    33.2789   YX=    -1.3811   ZX=     3.5138
XY=    -0.0162   YY=    27.7786   ZY=    -0.2172
XZ=     3.3085   YZ=     0.7795   ZZ=    25.3750
Eigenvalues:    24.0371    27.8022    34.5931
  57  H    Isotropic =    29.6171   Anisotropy =     4.9476
XX=    30.4635   YX=     2.9474   ZX=     0.4558
XY=     1.8586   YY=    26.0040   ZY=    -0.7393
XZ=     1.7581   YZ=    -0.6678   ZZ=    32.3839
Eigenvalues:    24.7981    31.1379    32.9155
  58  H    Isotropic =    29.6830   Anisotropy =     8.2541
XX=    31.5032   YX=    -2.7928   ZX=     3.2741
XY=    -2.5208   YY=    29.9866   ZY=    -1.8372
XZ=     2.6726   YZ=    -2.1555   ZZ=    27.5591
Eigenvalues:    25.8672    27.9961    35.1857
  59  H    Isotropic =    30.2813   Anisotropy =     9.2429
XX=    27.4885   YX=     1.1371   ZX=     1.4896
XY=     1.5285   YY=    27.1445   ZY=     0.0076
XZ=     1.3054   YZ=     0.2989   ZZ=    36.2108
Eigenvalues:    25.9063    28.4944    36.4432
  60  H    Isotropic =    30.0183   Anisotropy =    11.5873
XX=    26.0782   YX=     2.4707   ZX=     1.7286
XY=     2.1261   YY=    34.2865   ZY=     4.5146
XZ=     1.5943   YZ=     4.5216   ZZ=    29.6903
Eigenvalues:    25.3647    26.9471    37.7432
  61  H    Isotropic =    29.9952   Anisotropy =    11.9716
XX=    36.8897   YX=     3.3298   ZX=     0.6767
XY=     3.5772   YY=    26.5729   ZY=    -0.5502
XZ=     1.0003   YZ=    -0.4875   ZZ=    26.5230
Eigenvalues:    25.1227    26.8866    37.9762
  62  H    Isotropic =    30.0130   Anisotropy =     7.6907
XX=    28.9688   YX=    -1.4249   ZX=     1.5975
XY=    -2.3558   YY=    34.2838   ZY=    -1.1130
XZ=     1.5003   YZ=    -0.9110   ZZ=    26.7866
Eigenvalues:    25.9829    28.9160    35.1402
  63  H    Isotropic =    30.2155   Anisotropy =     8.9709
XX=    29.1755   YX=     3.2600   ZX=    -2.2058
```

```
XY=     3.7398   YY=    29.9711   ZY=    -3.2586
XZ=    -2.2594   YZ=    -2.9673   ZZ=    31.5000
Eigenvalues:    26.0027    28.4478    36.1961
  64  H    Isotropic =    30.3131   Anisotropy =     8.3014
XX=    30.3339   YX=     0.5348   ZX=    -2.4356
XY=     0.9594   YY=    35.6474   ZY=     1.4475
XZ=    -1.6618   YZ=     1.1078   ZZ=    24.9579
Eigenvalues:    24.0843    31.0076    35.8474
  65  H    Isotropic =    30.8186   Anisotropy =     8.5709
XX=    36.0402   YX=    -1.3218   ZX=     0.8876
XY=    -2.8378   YY=    27.6740   ZY=     1.7376
XZ=     0.3067   YZ=     1.8834   ZZ=    28.7416
Eigenvalues:    25.9104    30.0129    36.5325
  66  H    Isotropic =    31.3874   Anisotropy =     9.9874
XX=    31.3199   YX=     0.7172   ZX=    -5.3017
XY=    -0.2210   YY=    27.8265   ZY=     0.8887
XZ=    -3.7255   YZ=    -0.3710   ZZ=    35.0158
Eigenvalues:    27.6380    28.4785    38.0456
  67  H    Isotropic =    31.0710   Anisotropy =     8.9043
XX=    30.1893   YX=    -0.4604   ZX=    -3.6269
XY=     0.0928   YY=    27.2866   ZY=     0.8912
XZ=    -2.1021   YZ=     0.5627   ZZ=    35.7371
Eigenvalues:    27.2226    28.9831    37.0072
  68  H    Isotropic =    30.8060   Anisotropy =    10.0846
XX=    36.5242   YX=    -0.4079   ZX=     2.9804
XY=     0.2703   YY=    26.3156   ZY=     1.8082
XZ=     2.4553   YZ=     3.7499   ZZ=    29.5781
Eigenvalues:    24.5463    30.3426    37.5290
  69  H    Isotropic =    30.6192   Anisotropy =     7.5985
XX=    30.0666   YX=    -1.6254   ZX=    -1.6637
XY=    -0.6550   YY=    34.1243   ZY=     2.1211
XZ=    -1.0919   YZ=     3.7100   ZZ=    27.6666
Eigenvalues:    26.3237    29.8490    35.6849
  70  H    Isotropic =    30.6028   Anisotropy =     7.4038
XX=    28.0561   YX=     1.0821   ZX=     2.3204
XY=    -1.0933   YY=    33.1290   ZY=     2.6724
XZ=     0.7344   YZ=     3.9911   ZZ=    30.6234
Eigenvalues:    26.8718    29.3981    35.5387
  71  H    Isotropic =    30.9617   Anisotropy =     9.6993
XX=    27.7669   YX=     0.0273   ZX=     0.5931
XY=     0.3983   YY=    28.3330   ZY=    -2.0582
XZ=    -0.6004   YZ=    -2.7754   ZZ=    36.7853
Eigenvalues:    27.5205    27.9368    37.4279
  72  H    Isotropic =    30.1092   Anisotropy =     6.6833
XX=    33.0900   YX=    -0.8346   ZX=     2.9137
XY=    -0.4723   YY=    26.3038   ZY=     2.7031
XZ=     1.6322   YZ=     1.3296   ZZ=    30.9339
Eigenvalues:    25.2751    30.4878    34.5648
```

Chemical Shielding tensors (GIAO MPW1PW91/6-311+G(2d,p) iefpcm = DMSO) 3R*,7S*,11R*-1b

Conf. B (32 %)

```
1  C     Isotropic =    72.7799   Anisotropy =   131.1922
XX=    80.0520   YX=   -50.1863   ZX=    40.5406
XY=   -32.2089   YY=    10.7206   ZY=   -19.8823
XZ=    44.8210   YZ=    -7.7637   ZZ=   127.5670
Eigenvalues:    -8.7103    66.8085   160.2413
   2  C     Isotropic =    27.4256   Anisotropy =   128.4252
XX=    -3.0185   YX=   -14.3734   ZX=    74.7475
XY=   -12.5068   YY=    23.9818   ZY=   -15.2880
XZ=    71.1754   YZ=   -13.1099   ZZ=    61.3134
Eigenvalues:   -50.7592    19.9936   113.0423
   3  C     Isotropic =    66.8152   Anisotropy =   127.3166
XX=    75.4845   YX=    14.3083   ZX=    54.0031
XY=     9.0494   YY=    16.5400   ZY=   -40.6356
XZ=    47.3061   YZ=   -46.4618   ZZ=   108.4209
Eigenvalues:   -12.5357    61.2882   151.6929
   4  C     Isotropic =    60.4616   Anisotropy =   170.6893
XX=    68.5296   YX=   -39.3217   ZX=    61.7194
XY=   -30.3721   YY=    -5.0061   ZY=   -20.1596
XZ=    78.0252   YZ=   -16.5789   ZZ=   117.8612
Eigenvalues:   -19.7839    26.9142   174.2544
   5  C     Isotropic =    35.4177   Anisotropy =   120.8592
XX=     2.8223   YX=   -20.7722   ZX=    68.4510
XY=   -12.1488   YY=    41.6693   ZY=   -12.6110
XZ=    75.0545   YZ=   -16.6477   ZZ=    61.7614
Eigenvalues:   -45.6389    35.9014   115.9905
   6  C     Isotropic =    54.3753   Anisotropy =   164.4667
XX=    60.2872   YX=     1.9144   ZX=    71.1233
XY=    -6.4147   YY=    -1.6262   ZY=   -44.6391
XZ=    67.6682   YZ=   -45.6086   ZZ=   104.4650
Eigenvalues:   -26.5354    25.6417   164.0198
   7  C     Isotropic =   161.1058   Anisotropy =    19.0134
XX=   163.6685   YX=     4.6738   ZX=    -7.3799
XY=     6.9186   YY=   164.9999   ZY=    -9.3117
XZ=    -0.3144   YZ=    -5.9212   ZZ=   154.6488
Eigenvalues:   150.5709   158.9651   173.7814
   8  C     Isotropic =   149.2933   Anisotropy =    28.2902
XX=   153.4179   YX=   -10.0415   ZX=     4.7945
XY=   -19.4241   YY=   152.6509   ZY=    -1.7020
XZ=     7.8273   YZ=     5.7742   ZZ=   141.8113
Eigenvalues:   133.8314   145.8951   168.1534
   9  C     Isotropic =   105.9092   Anisotropy =    50.6087
XX=   106.6322   YX=    27.8864   ZX=     9.6662
XY=    27.1199   YY=   116.6072   ZY=    -1.7273
XZ=     2.1879   YZ=    -3.3496   ZZ=    94.4883
Eigenvalues:    80.8572    97.2221   139.6484
  10  O     Isotropic =   188.7340   Anisotropy =   112.7029
XX=   179.5672   YX=     1.7845   ZX=    51.5271
XY=   -17.8131   YY=   134.6789   ZY=   -17.4173
XZ=     7.6318   YZ=    -5.0367   ZZ=   251.9559
Eigenvalues:   132.9253   169.4075   263.8693
  11  C     Isotropic =   143.2652   Anisotropy =    43.8553
XX=   123.5167   YX=    14.2430   ZX=     5.1052
XY=    16.1749   YY=   145.6570   ZY=     8.1024
XZ=    13.3383   YZ=    16.1327   ZZ=   160.6218
Eigenvalues:   115.5862   141.7073   172.5021
  12  C     Isotropic =    45.3190   Anisotropy =   136.0714
XX=    42.6248   YX=    17.0453   ZX=     3.4056
XY=    15.5216   YY=   -41.3349   ZY=    12.2086
XZ=    -3.4567   YZ=    18.6831   ZZ=   134.6671
Eigenvalues:   -45.6620    45.5857   136.0333
  13  C     Isotropic =    21.5590   Anisotropy =   189.8302
XX=    -0.8448   YX=    38.1806   ZX=     4.1720
XY=    20.5818   YY=   -78.7913   ZY=    30.0485
XZ=    15.9751   YZ=    18.4914   ZZ=   144.3131
Eigenvalues:   -90.3210     6.8856   148.1124
  14  C     Isotropic =   141.9189   Anisotropy =    25.9416
XX=   137.8648   YX=    -9.3865   ZX=     9.8213
XY=    -7.7510   YY=   147.3606   ZY=     2.9641
XZ=    20.3616   YZ=    -7.2716   ZZ=   140.5311
Eigenvalues:   123.0013   143.5420   159.2133
  15  C     Isotropic =   166.7172   Anisotropy =    26.6395
XX=   162.7635   YX=    -2.5537   ZX=    -4.3288
XY=    -4.3887   YY=   178.0500   ZY=    11.6394
XZ=    -3.0747   YZ=    11.1695   ZZ=   159.3381
Eigenvalues:   153.5344   162.1403   184.4769
```

```
  16  O    Isotropic =   235.5130   Anisotropy =    77.5712
XX=   264.2554   YX=   -29.3380   ZX=    -0.9752
XY=   -65.2083   YY=   163.6048   ZY=    13.7867
XZ=    -9.9750   YZ=    -8.6955   ZZ=   278.6787
Eigenvalues:   144.8829   274.4289   287.2271
  17  C    Isotropic =   132.0787   Anisotropy =    72.4712
XX=   131.5686   YX=    35.9159   ZX=    -3.2384
XY=    32.3039   YY=   153.7276   ZY=   -10.1222
XZ=    -4.3634   YZ=   -12.5724   ZZ=   110.9400
Eigenvalues:   104.1947   111.6487   180.3929
  18  C    Isotropic =   108.2250   Anisotropy =    52.7811
XX=   102.3091   YX=    23.4856   ZX=    -4.3566
XY=    22.1799   YY=   128.2541   ZY=   -10.8060
XZ=    -1.9329   YZ=    -7.7378   ZZ=    94.1118
Eigenvalues:    88.1315    93.1311   143.4124
  19  C    Isotropic =   158.8954   Anisotropy =    35.1394
XX=   171.4082   YX=   -10.8606   ZX=    -8.7186
XY=   -13.4727   YY=   148.4008   ZY=     3.4484
XZ=   -12.2136   YZ=     8.6357   ZZ=   156.8772
Eigenvalues:   142.9546   151.4099   182.3216
  20  O    Isotropic =   221.1694   Anisotropy =    71.3005
XX=   225.9680   YX=    26.9079   ZX=   -26.5019
XY=    20.6449   YY=   232.4401   ZY=   -12.6137
XZ=   -27.3520   YZ=   -22.9752   ZZ=   205.1001
Eigenvalues:   186.6057   208.1995   268.7031
  21  C    Isotropic =   155.6893   Anisotropy =    36.8460
XX=   161.5905   YX=   -15.5401   ZX=    11.3780
XY=   -11.5733   YY=   152.7439   ZY=    -7.4768
XZ=    15.4995   YZ=    -8.5035   ZZ=   152.7335
Eigenvalues:   142.0651   144.7496   180.2533
  22  C    Isotropic =   -25.0768   Anisotropy =   182.0712
XX=   -74.0720   YX=    21.2607   ZX=     7.2928
XY=    25.5745   YY=   -97.1138   ZY=    -5.1250
XZ=    -5.3509   YZ=   -11.3016   ZZ=    95.9552
Eigenvalues:  -111.9609   -59.5736    96.3040
  23  C    Isotropic =   126.9837   Anisotropy =    28.1554
XX=   127.0000   YX=     6.7423   ZX=    -6.9130
XY=     0.9215   YY=   138.4613   ZY=    -5.7418
XZ=    -4.4066   YZ=   -19.2138   ZZ=   115.4898
Eigenvalues:   109.2265   125.9707   145.7540
  24  C    Isotropic =   137.4343   Anisotropy =    25.2630
XX=   147.6773   YX=    -6.6408   ZX=    -5.8424
XY=    -3.7119   YY=   148.6807   ZY=    -3.5768
XZ=   -11.6447   YZ=     2.6753   ZZ=   115.9448
Eigenvalues:   113.6085   144.4180   154.2763
  25  C    Isotropic =   140.4118   Anisotropy =    30.1946
XX=   128.6613   YX=    -9.1108   ZX=     1.8981
XY=   -13.0972   YY=   152.2010   ZY=   -12.6538
XZ=     4.2349   YZ=    -4.0668   ZZ=   140.3733
Eigenvalues:   124.2459   136.4481   160.5416
  26  C    Isotropic =   153.9331   Anisotropy =    32.3897
XX=   158.5552   YX=    -2.7865   ZX=    -4.0263
XY=   -10.3082   YY=   166.5167   ZY=   -17.9579
XZ=     3.5727   YZ=   -13.9369   ZZ=   136.7273
Eigenvalues:   129.5198   156.7532   175.5262
  27  C    Isotropic =   164.7565   Anisotropy =    28.7515
XX=   182.5534   YX=    -0.6823   ZX=    -8.3237
XY=    -0.1539   YY=   174.0773   ZY=    -8.7805
XZ=    -6.9754   YZ=    -7.7115   ZZ=   137.6388
Eigenvalues:   134.6635   175.6819   183.9242
  28  C    Isotropic =   149.0123   Anisotropy =    42.9414
XX=   147.1803   YX=    -4.8023   ZX=    -3.0800
XY=     1.7066   YY=   177.4777   ZY=     2.5407
XZ=     2.7064   YZ=     1.7389   ZZ=   122.3789
Eigenvalues:   122.2953   147.1017   177.6399
  29  C    Isotropic =   164.6999   Anisotropy =    37.7094
XX=   156.2172   YX=     4.1580   ZX=     0.8116
XY=     4.9540   YY=   150.8142   ZY=    10.4682
XZ=     0.7952   YZ=     9.8756   ZZ=   187.0684
Eigenvalues:   146.3481   157.9122   189.8395
  30  C    Isotropic =   163.6601   Anisotropy =    34.3144
XX=   156.6639   YX=    -9.0048   ZX=     5.6684
XY=    -5.3895   YY=   149.4394   ZY=     5.6016
XZ=     7.8701   YZ=     1.8741   ZZ=   184.8769
Eigenvalues:   143.8695   160.5743   186.5363
  31  O    Isotropic =  -281.2428   Anisotropy =   957.9409
XX=  -840.0165   YX=   -57.5151   ZX=    43.4025
XY=   -32.3762   YY=  -359.5572   ZY=   -19.9162
XZ=    24.6398   YZ=   -18.0056   ZZ=   355.8452
```

```
Eigenvalues:  -845.0449  -356.0680   357.3844
  32  C    Isotropic =   159.6419   Anisotropy =    37.4758
XX=   158.6942   YX=    -0.1742   ZX=   -15.2943
XY=     1.5086   YY=   146.0168   ZY=     3.3116
XZ=   -17.2833   YZ=     2.7885   ZZ=   174.2147
Eigenvalues:   144.6239   149.6760   184.6258
  33  H    Isotropic =    25.0746   Anisotropy =     9.4279
XX=    27.6793   YX=    -1.3787   ZX=    -4.5905
XY=    -1.4870   YY=    23.9640   ZY=     2.1481
XZ=    -4.4053   YZ=     2.2516   ZZ=    23.5805
Eigenvalues:    20.3134    23.5506    31.3599
  34  H    Isotropic =    24.8669   Anisotropy =     8.2997
XX=    27.7462   YX=     2.2481   ZX=    -3.6431
XY=     2.4986   YY=    24.9113   ZY=    -0.6873
XZ=    -3.0844   YZ=    -0.8104   ZZ=    21.9432
Eigenvalues:    20.3779    23.8227    30.4001
  35  H    Isotropic =    28.7487   Anisotropy =     6.2279
XX=    29.6131   YX=     1.7706   ZX=     0.1651
XY=     0.1780   YY=    27.3054   ZY=     3.6449
XZ=     0.8288   YZ=     4.6771   ZZ=    29.3277
Eigenvalues:    23.9962    29.3493    32.9006
  36  H    Isotropic =    29.0461   Anisotropy =    10.4588
XX=    30.7815   YX=    -4.5995   ZX=    -2.4875
XY=    -4.4251   YY=    32.0729   ZY=    -0.1135
XZ=    -2.1984   YZ=    -2.3504   ZZ=    24.2838
Eigenvalues:    22.6733    28.4463    36.0186
  37  H    Isotropic =    29.9354   Anisotropy =     6.9861
XX=    29.6405   YX=     1.6460   ZX=    -1.4041
XY=     1.5686   YY=    33.7067   ZY=    -1.4569
XZ=    -1.1266   YZ=    -1.0956   ZZ=    26.4589
Eigenvalues:    25.9505    29.2629    34.5927
  38  H    Isotropic =    29.9486   Anisotropy =     8.2211
XX=    29.3322   YX=    -3.6801   ZX=     2.4995
XY=    -2.8058   YY=    27.6296   ZY=    -2.7041
XZ=     0.8378   YZ=    -2.6449   ZZ=    32.8842
Eigenvalues:    24.9471    29.4694    35.4294
  39  H    Isotropic =    29.3643   Anisotropy =     6.6967
XX=    31.4261   YX=     2.4826   ZX=     2.7786
XY=     1.3999   YY=    28.2548   ZY=    -0.1689
XZ=     4.0866   YZ=    -2.5112   ZZ=    28.4121
Eigenvalues:    24.7673    29.4969    33.8288
  40  H    Isotropic =    28.9633   Anisotropy =     3.6536
XX=    29.2548   YX=    -0.5151   ZX=    -1.3404
XY=     0.7336   YY=    29.2480   ZY=     1.8001
XZ=    -3.4458   YZ=     0.1546   ZZ=    28.3872
Eigenvalues:    26.1664    29.3245    31.3991
  41  H    Isotropic =    29.2626   Anisotropy =     5.4936
XX=    32.1752   YX=    -0.7314   ZX=     0.6006
XY=    -3.3518   YY=    26.6588   ZY=     0.1615
XZ=     0.9064   YZ=     0.9278   ZZ=    28.9537
Eigenvalues:    25.8029    29.0599    32.9250
  42  H    Isotropic =    28.9025   Anisotropy =    11.3175
XX=    33.3227   YX=     1.6787   ZX=     4.5862
XY=     3.2230   YY=    29.7131   ZY=     2.3022
XZ=     3.9176   YZ=     2.6278   ZZ=    23.6717
Eigenvalues:    21.7767    28.4833    36.4475
  43  H    Isotropic =    29.2944   Anisotropy =    10.2512
XX=    35.2142   YX=    -1.6738   ZX=     1.6292
XY=    -2.1368   YY=    29.7288   ZY=     0.4444
XZ=     2.5509   YZ=    -0.7743   ZZ=    22.9402
Eigenvalues:    22.5908    29.1638    36.1285
  44  H    Isotropic =    29.2579   Anisotropy =     6.7066
XX=    26.7103   YX=     2.3001   ZX=    -0.2044
XY=     1.1899   YY=    28.0586   ZY=     1.8736
XZ=    -1.5135   YZ=     2.1545   ZZ=    33.0048
Eigenvalues:    25.0653    28.9794    33.7290
  45  H    Isotropic =    29.8978   Anisotropy =     8.7237
XX=    30.8261   YX=     2.7851   ZX=    -1.9695
XY=     4.2274   YY=    33.1935   ZY=     0.7220
XZ=    -1.3898   YZ=     2.1143   ZZ=    25.6738
Eigenvalues:    24.4324    29.5474    35.7136
  46  H    Isotropic =    27.8829   Anisotropy =     9.0130
XX=    30.8178   YX=     1.2449   ZX=    -4.4825
XY=     2.9733   YY=    26.9107   ZY=    -1.0139
XZ=    -4.0517   YZ=     0.1375   ZZ=    25.9200
Eigenvalues:    23.3050    26.4521    33.8915
  47  H    Isotropic =    28.1497   Anisotropy =     7.8849
XX=    26.1954   YX=     1.8973   ZX=    -1.3384
XY=    -0.2989   YY=    31.0682   ZY=    -3.1771
```

```
XZ=    -1.8191   YZ=    -3.7438   ZZ=    27.1855
Eigenvalues:    24.5138    26.5290    33.4063
  48  H    Isotropic =    28.1464   Anisotropy =     8.3764
XX=    30.4595   YX=     4.1748   ZX=     1.6186
XY=     2.8657   YY=    28.1531   ZY=     3.0798
XZ=     1.1091   YZ=     1.2301   ZZ=    25.8264
Eigenvalues:    24.4190    26.2895    33.7306
  49  H    Isotropic =    30.6873   Anisotropy =     6.9358
XX=    33.3246   YX=    -2.6969   ZX=     2.7422
XY=    -2.6291   YY=    31.2480   ZY=     0.3073
XZ=     1.4966   YZ=     2.0617   ZZ=    27.4892
Eigenvalues:    25.9879    30.7628    35.3112
  50  H    Isotropic =    30.6396   Anisotropy =     8.9545
XX=    29.0027   YX=    -0.3444   ZX=     0.0037
XY=     0.0113   YY=    29.7547   ZY=     4.3489
XZ=     1.1714   YZ=     5.3330   ZZ=    33.1614
Eigenvalues:    26.2438    29.0657    36.6093
  51  H    Isotropic =    30.8870   Anisotropy =     5.4231
XX=    32.0143   YX=     3.6025   ZX=    -1.1722
XY=     2.7048   YY=    30.4582   ZY=     0.6129
XZ=    -1.2082   YZ=     1.5658   ZZ=    30.1886
Eigenvalues:    27.1514    31.0073    34.5024
  52  H    Isotropic =    31.3955   Anisotropy =    15.1849
XX=    37.3789   YX=     2.1402   ZX=    -6.8683
XY=     3.2912   YY=    29.4721   ZY=    -1.8571
XZ=    -6.6747   YZ=    -0.5544   ZZ=    27.3354
Eigenvalues:    23.9227    28.7450    41.5187
  53  H    Isotropic =    30.3115   Anisotropy =     7.4974
XX=    34.3961   YX=    -2.1328   ZX=    -1.4461
XY=    -2.3348   YY=    29.3310   ZY=     2.0463
XZ=     1.6069   YZ=     2.4388   ZZ=    27.2074
Eigenvalues:    25.5922    30.0325    35.3097
  54  H    Isotropic =    30.9729   Anisotropy =     6.0228
XX=    28.6553   YX=     1.3040   ZX=     1.9289
XY=     1.5961   YY=    34.1487   ZY=    -2.2942
XZ=     2.1950   YZ=    -1.5704   ZZ=    30.1148
Eigenvalues:    26.4969    31.4338    34.9881
  55  H    Isotropic =    30.7742   Anisotropy =     8.8859
XX=    30.7488   YX=     1.2986   ZX=     3.8915
XY=     0.4583   YY=    28.9386   ZY=     1.7497
XZ=     4.2244   YZ=     3.3388   ZZ=    32.6352
Eigenvalues:    26.9129    28.7116    36.6981
  56  H    Isotropic =    28.5294   Anisotropy =     9.3883
XX=    31.5695   YX=    -2.6375   ZX=     2.8076
XY=    -2.8660   YY=    28.3718   ZY=    -0.8228
XZ=     5.4572   YZ=    -0.0084   ZZ=    25.6469
Eigenvalues:    23.3434    27.4565    34.7883
  57  H    Isotropic =    29.9089   Anisotropy =     8.9744
XX=    34.2238   YX=    -3.6123   ZX=    -0.7840
XY=    -2.9032   YY=    29.2542   ZY=    -1.9444
XZ=    -0.0128   YZ=    -2.8535   ZZ=    26.2488
Eigenvalues:    24.4881    29.3468    35.8919
  58  H    Isotropic =    29.7959   Anisotropy =     8.5302
XX=    32.8734   YX=     0.1627   ZX=     3.8300
XY=    -0.3218   YY=    27.7077   ZY=    -0.0398
XZ=     4.5073   YZ=    -0.4136   ZZ=    28.8067
Eigenvalues:    26.1870    27.7180    35.4827
  59  H    Isotropic =    30.1720   Anisotropy =     9.6628
XX=    33.9672   YX=    -2.5647   ZX=    -4.1092
XY=    -2.6999   YY=    28.9567   ZY=     0.5502
XZ=    -3.4175   YZ=     0.5659   ZZ=    27.5922
Eigenvalues:    25.7071    28.1951    36.6139
  60  H    Isotropic =    29.9976   Anisotropy =    11.5576
XX=    27.4224   YX=    -1.4473   ZX=    -0.9831
XY=    -1.4226   YY=    35.9186   ZY=     4.3205
XZ=    -0.7335   YZ=     3.7798   ZZ=    26.6519
Eigenvalues:    25.0889    27.2013    37.7027
  61  H    Isotropic =    30.0402   Anisotropy =    11.8076
XX=    27.8520   YX=    -2.2071   ZX=     1.5830
XY=    -2.1748   YY=    31.9577   ZY=    -6.0779
XZ=     1.6286   YZ=    -5.9076   ZZ=    30.3108
Eigenvalues:    25.0611    27.1475    37.9119
  62  H    Isotropic =    29.9568   Anisotropy =     7.4637
XX=    31.6661   YX=     1.4365   ZX=     3.1727
XY=     1.8786   YY=    30.5105   ZY=     2.4793
XZ=     3.6080   YZ=     1.3794   ZZ=    27.6937
Eigenvalues:    25.5793    29.3585    34.9325
  63  H    Isotropic =    30.2914   Anisotropy =     8.2461
XX=    31.7390   YX=     1.9710   ZX=    -3.4774
```

```
XY=     1.9837   YY=    30.8542   ZY=    -3.0502
XZ=    -2.7178   YZ=    -2.9572   ZZ=    28.2812
Eigenvalues:    25.7855    29.3000    35.7888
  64  H    Isotropic =    30.7090   Anisotropy =     8.1367
XX=    26.4608   YX=     3.3803   ZX=     2.2143
XY=     2.8313   YY=    34.3456   ZY=     0.9134
XZ=     2.0341   YZ=     1.4300   ZZ=    31.3205
Eigenvalues:    24.9496    31.0439    36.1334
  65  H    Isotropic =    30.8873   Anisotropy =     8.6788
XX=    29.4573   YX=    -3.4022   ZX=     2.9436
XY=    -2.7772   YY=    28.9006   ZY=    -0.2938
XZ=     3.4374   YZ=    -1.9463   ZZ=    34.3042
Eigenvalues:    25.8249    30.1639    36.6732
  66  H    Isotropic =    31.5149   Anisotropy =     8.9824
XX=    31.2275   YX=    -0.3106   ZX=    -2.9758
XY=    -0.3345   YY=    28.1365   ZY=    -0.4424
XZ=    -4.5885   YZ=    -1.2153   ZZ=    35.1807
Eigenvalues:    27.7032    29.3384    37.5032
  67  H    Isotropic =    30.9751   Anisotropy =     9.7230
XX=    31.7655   YX=    -0.6489   ZX=    -3.9585
XY=    -0.7401   YY=    27.4096   ZY=     0.2287
XZ=    -5.0626   YZ=     0.9672   ZZ=    33.7501
Eigenvalues:    27.2961    28.1721    37.4571
  68  H    Isotropic =    30.6309   Anisotropy =     8.1622
XX=    29.8506   YX=    -5.2933   ZX=     0.6682
XY=    -4.6351   YY=    29.6750   ZY=    -1.5146
XZ=     2.8784   YZ=    -1.2402   ZZ=    32.3672
Eigenvalues:    24.7883    31.0321    36.0724
  69  H    Isotropic =    30.5474   Anisotropy =     7.5367
XX=    27.2805   YX=     0.9211   ZX=     0.4126
XY=     1.4976   YY=    32.0342   ZY=     2.5435
XZ=     1.1915   YZ=     3.7477   ZZ=    32.3276
Eigenvalues:    26.9899    29.0805    35.5719
  70  H    Isotropic =    30.2736   Anisotropy =     6.3861
XX=    29.5758   YX=    -4.5134   ZX=    -0.4671
XY=    -3.9568   YY=    30.7910   ZY=    -0.1399
XZ=    -0.1762   YZ=     0.9871   ZZ=    30.4540
Eigenvalues:    25.9047    30.3852    34.5310
  71  H    Isotropic =    30.6864   Anisotropy =     7.5693
XX=    32.2600   YX=     3.2904   ZX=    -1.4256
XY=     3.3126   YY=    29.6891   ZY=    -1.9571
XZ=    -1.3411   YZ=    -3.0887   ZZ=    30.1100
Eigenvalues:    26.7554    29.5711    35.7326
  72  H    Isotropic =    30.5841   Anisotropy =     9.0406
XX=    28.5115   YX=    -0.7959   ZX=     1.5685
XY=    -0.3375   YY=    27.1340   ZY=    -0.2683
XZ=     2.2815   YZ=    -0.7916   ZZ=    36.1068
Eigenvalues:    26.9275    28.2136    36.6111
```

Chemical Shielding tensors (GIAO mpw1pw91/6-311+g(2d,p) iefpcm = DMSO) 3R*,7S*,11R*-1b

Conf. C (51 %)

```
    1  C    Isotropic =    73.0079   Anisotropy =   130.2652
XX=    57.5284   YX=   -38.5595   ZX=    -8.4547
XY=   -16.7236   YY=     3.2836   ZY=     9.5386
XZ=   -10.4714   YZ=     6.7006   ZZ=   158.2118
Eigenvalues:    -8.4084    67.5808   159.8514
    2  C    Isotropic =    27.4782   Anisotropy =   127.9972
XX=   -48.6699   YX=    -6.4531   ZX=   -16.8483
XY=    -4.3926   YY=    20.2328   ZY=     5.9223
XZ=   -15.0426   YZ=     4.5975   ZZ=   110.8716
Eigenvalues:   -50.5850    20.2098   112.8097
    3  C    Isotropic =    67.0475   Anisotropy =   126.5704
XX=    39.2303   YX=    37.6330   ZX=   -13.5271
XY=    29.1019   YY=    12.6136   ZY=    12.8277
XZ=    -8.4968   YZ=    16.9703   ZZ=   149.2987
Eigenvalues:   -12.1245    61.8392   151.4278
    4  C    Isotropic =    60.3953   Anisotropy =   170.1491
XX=    22.7798   YX=   -24.1575   ZX=   -14.1134
XY=   -12.9694   YY=   -11.5147   ZY=     9.7054
XZ=   -29.8361   YZ=     7.7003   ZZ=   169.9207
Eigenvalues:   -19.6425    27.0004   173.8280
    5  C    Isotropic =    35.0441   Anisotropy =   118.1987
XX=   -42.4376   YX=   -11.9452   ZX=   -15.9378
XY=    -4.9980   YY=    36.8709   ZY=     5.3940
XZ=   -17.5302   YZ=    13.1352   ZZ=   110.6992
Eigenvalues:   -44.9248    36.2140   113.8432
    6  C    Isotropic =    54.6002   Anisotropy =   162.3293
XX=    12.2245   YX=    27.4552   ZX=   -17.9898
XY=    18.7502   YY=    -8.5918   ZY=    15.0233
XZ=   -15.7438   YZ=    12.7831   ZZ=   160.1678
Eigenvalues:   -25.8658    26.8466   162.8197
    7  C    Isotropic =   160.8352   Anisotropy =    19.6159
XX=   165.3486   YX=     6.0554   ZX=     3.3958
XY=    11.4631   YY=   163.9637   ZY=     5.1720
XZ=    -2.6123   YZ=     3.0794   ZZ=   153.1932
Eigenvalues:   151.1475   157.4456   173.9125
    8  C    Isotropic =   150.2228   Anisotropy =    27.1263
XX=   151.3529   YX=   -10.5856   ZX=    -5.1332
XY=   -14.5808   YY=   152.1966   ZY=    10.6390
XZ=   -10.5302   YZ=    -0.4492   ZZ=   147.1191
Eigenvalues:   138.3959   143.9656   168.3070
    9  C    Isotropic =   104.9797   Anisotropy =    50.2518
XX=   102.1096   YX=    25.8874   ZX=    -5.7662
XY=    24.1900   YY=   118.2471   ZY=    -9.4278
XZ=     1.5233   YZ=   -10.5577   ZZ=    94.5823
Eigenvalues:    82.2517    94.2065   138.4809
   10  O    Isotropic =   187.3909   Anisotropy =   122.2331
XX=   163.2115   YX=     3.2647   ZX=   -10.3696
XY=   -17.7980   YY=   131.2862   ZY=     0.8158
XZ=    21.2564   YZ=   -22.5791   ZZ=   267.6751
Eigenvalues:   129.0539   164.2392   268.8797
   11  C    Isotropic =   143.1199   Anisotropy =    44.5514
XX=   140.9389   YX=    20.5711   ZX=    16.7827
XY=    18.4145   YY=   145.0750   ZY=    -1.1417
XZ=    25.1117   YZ=     7.0672   ZZ=   143.3458
Eigenvalues:   115.2220   141.3169   172.8209
   12  C    Isotropic =    44.9424   Anisotropy =   136.7154
XX=    63.4490   YX=    24.4057   ZX=    43.4429
XY=    19.0253   YY=   -41.5718   ZY=     2.8981
XZ=    35.3326   YZ=     8.4388   ZZ=   112.9501
Eigenvalues:   -45.9157    44.6569   136.0860
   13  C    Isotropic =    22.3183   Anisotropy =   188.7687
XX=    41.2846   YX=    43.5690   ZX=    60.0533
XY=    35.0383   YY=   -77.0500   ZY=    17.3316
XZ=    71.6283   YZ=    -1.5887   ZZ=   102.7203
Eigenvalues:   -89.7269     8.5177   148.1641
   14  C    Isotropic =   134.6467   Anisotropy =    33.3728
XX=   145.4677   YX=   -18.0763   ZX=     9.3479
XY=   -10.1875   YY=   127.2055   ZY=     6.2536
XZ=    14.9575   YZ=    -1.1094   ZZ=   131.2669
Eigenvalues:   114.8789   132.1659   156.8952
   15  C    Isotropic =   166.0767   Anisotropy =    25.9504
XX=   167.6067   YX=    -7.9832   ZX=     1.3238
XY=    -9.6072   YY=   177.9576   ZY=    -3.2356
XZ=     1.5027   YZ=    -3.1194   ZZ=   152.6658
Eigenvalues:   152.2642   162.5889   183.3770
   16  O    Isotropic =   235.7343   Anisotropy =    77.7012
```

```
XX=   274.9535   YX=   -18.6078   ZX=     1.3261
XY=   -61.5843   YY=   157.7340   ZY=     1.9137
XZ=     3.9241   YZ=     4.7821   ZZ=   274.5153
Eigenvalues:   145.2090   274.4588   287.5351
  17  C    Isotropic =   132.1058   Anisotropy =    72.4368
XX=   127.9063   YX=    37.3265   ZX=    -1.0627
XY=    32.8998   YY=   156.9081   ZY=     0.1716
XZ=    -0.5745   YZ=     0.6351   ZZ=   111.5031
Eigenvalues:   104.3039   111.6166   180.3970
  18  C    Isotropic =   108.1374   Anisotropy =    52.7121
XX=    98.0731   YX=    16.9807   ZX=    -6.2954
XY=    14.5452   YY=   127.8734   ZY=   -20.0959
XZ=    -4.0002   YZ=   -18.1825   ZZ=    98.4656
Eigenvalues:    87.8717    93.2617   143.2788
  19  C    Isotropic =   158.8707   Anisotropy =    35.1862
XX=   158.7471   YX=    -5.8971   ZX=    -9.5257
XY=   -10.6278   YY=   148.5630   ZY=     9.7810
XZ=   -12.8491   YZ=    13.2513   ZZ=   169.3019
Eigenvalues:   142.5705   151.7134   182.3281
  20  O    Isotropic =   220.9344   Anisotropy =    70.8849
XX=   198.6176   YX=    12.7428   ZX=   -22.5803
XY=    12.3554   YY=   231.9384   ZY=   -20.8350
XZ=   -23.6088   YZ=   -32.8381   ZZ=   232.2472
Eigenvalues:   186.8323   207.7800   268.1910
  21  C    Isotropic =   155.6480   Anisotropy =    36.7163
XX=   170.2669   YX=   -17.9568   ZX=     1.6157
XY=   -13.8988   YY=   153.2833   ZY=    -0.9552
XZ=     5.5602   YZ=     0.0128   ZZ=   143.3938
Eigenvalues:   141.9387   144.8797   180.1255
  22  C    Isotropic =   -25.5426   Anisotropy =   182.7539
XX=   -33.5054   YX=    12.4670   ZX=    79.3696
XY=    18.8430   YY=   -98.0473   ZY=   -17.7881
XZ=    66.0933   YZ=   -21.4988   ZZ=    54.9249
Eigenvalues:  -112.6424   -60.2787    96.2934
  23  C    Isotropic =   126.9025   Anisotropy =    28.3341
XX=   119.0791   YX=    -3.1580   ZX=    -8.4753
XY=    -2.0598   YY=   138.5278   ZY=    -5.3493
XZ=    -6.6323   YZ=   -20.1328   ZZ=   123.1007
Eigenvalues:   109.4581   125.4575   145.7920
  24  C    Isotropic =   137.3610   Anisotropy =    24.3589
XX=   132.3253   YX=    -3.8679   ZX=   -14.1940
XY=    -4.9048   YY=   148.7572   ZY=    -1.4576
XZ=   -20.6300   YZ=     5.7469   ZZ=   131.0004
Eigenvalues:   114.1723   144.3104   153.6002
  25  C    Isotropic =   138.4951   Anisotropy =    31.6496
XX=   131.1780   YX=   -10.1673   ZX=     6.8280
XY=   -17.4671   YY=   151.4785   ZY=    -5.5820
XZ=     7.4904   YZ=     0.7294   ZZ=   132.8289
Eigenvalues:   121.5215   134.3690   159.5949
  26  C    Isotropic =   153.9629   Anisotropy =    32.4084
XX=   152.8524   YX=    -8.8986   ZX=   -13.0662
XY=   -17.5336   YY=   166.9471   ZY=   -10.6592
XZ=    -5.7275   YZ=   -10.7803   ZZ=   142.0893
Eigenvalues:   129.6258   156.6945   175.5685
  27  C    Isotropic =   164.6807   Anisotropy =    28.6275
XX=   165.0217   YX=    -4.2470   ZX=   -23.7165
XY=    -4.3901   YY=   174.0806   ZY=    -7.5487
XZ=   -22.5425   YZ=    -6.2563   ZZ=   154.9397
Eigenvalues:   134.6499   175.6264   183.7657
  28  C    Isotropic =   148.9495   Anisotropy =    43.0287
XX=   140.9260   YX=    -2.5005   ZX=   -13.0291
XY=     3.3670   YY=   177.5164   ZY=     1.0375
XZ=    -7.5811   YZ=     3.7974   ZZ=   128.4060
Eigenvalues:   122.5107   146.7025   177.6353
  29  C    Isotropic =   164.4742   Anisotropy =    38.2864
XX=   163.9766   YX=     8.5737   ZX=    14.0274
XY=     9.5442   YY=   150.4198   ZY=     6.6888
XZ=    13.6801   YZ=     6.5569   ZZ=   179.0264
Eigenvalues:   145.8805   157.5437   189.9985
  30  C    Isotropic =   163.5892   Anisotropy =    34.7085
XX=   169.4751   YX=    -7.1366   ZX=    15.0744
XY=    -2.4746   YY=   149.5445   ZY=     7.3282
XZ=    16.9184   YZ=     6.2043   ZZ=   171.7480
Eigenvalues:   143.5089   160.5304   186.7281
  31  O    Isotropic =  -283.0845   Anisotropy =   960.9268
XX=  -532.5320   YX=   -56.9332   ZX=   535.4594
XY=   -36.2534   YY=  -360.6674   ZY=    -7.1250
XZ=   519.2439   YZ=     5.2367   ZZ=    43.9458
Eigenvalues:  -848.4938  -358.2931   357.5333
```

```
  32  C    Isotropic =   166.9124   Anisotropy =    30.3950
XX=   150.2187   YX=     4.9944   ZX=    -6.4653
XY=     7.0776   YY=   166.7057   ZY=    -3.1517
XZ=   -11.0790   YZ=    -3.9739   ZZ=   183.8128
Eigenvalues:   146.7658   166.7956   187.1757
  33  H    Isotropic =    25.1094   Anisotropy =     9.8863
XX=    30.8577   YX=    -2.2837   ZX=     1.0969
XY=    -2.2819   YY=    24.1926   ZY=    -0.5640
XZ=     1.1351   YZ=    -0.6429   ZZ=    20.2779
Eigenvalues:    20.1244    23.5035    31.7003
  34  H    Isotropic =    24.9025   Anisotropy =     8.3277
XX=    29.1253   YX=     2.4899   ZX=     0.5006
XY=     2.7719   YY=    25.2009   ZY=    -0.0227
XZ=     0.1203   YZ=     0.1367   ZZ=    20.3813
Eigenvalues:    20.3700    23.8833    30.4543
  35  H    Isotropic =    29.0082   Anisotropy =    10.2132
XX=    31.8583   YX=    -2.9853   ZX=    -1.1252
XY=    -3.7038   YY=    30.9538   ZY=     2.7938
XZ=    -1.4311   YZ=     4.5973   ZZ=    24.2126
Eigenvalues:    22.5773    28.6304    35.8171
  36  H    Isotropic =    28.7777   Anisotropy =     7.5737
XX=    29.2918   YX=    -0.6482   ZX=     1.4248
XY=    -1.4071   YY=    28.3297   ZY=    -3.9082
XZ=     0.6662   YZ=    -5.7490   ZZ=    28.7114
Eigenvalues:    23.6881    28.8180    33.8268
  37  H    Isotropic =    29.8770   Anisotropy =     5.7748
XX=    30.0894   YX=    -2.3519   ZX=    -0.2612
XY=    -1.8071   YY=    27.0570   ZY=     2.8272
XZ=     1.4966   YZ=     2.8727   ZZ=    32.4845
Eigenvalues:    24.9338    30.9704    33.7268
  38  H    Isotropic =    30.0127   Anisotropy =     6.9917
XX=    31.4646   YX=     1.3591   ZX=     0.5119
XY=     1.5752   YY=    33.9828   ZY=     0.6541
XZ=     0.4130   YZ=    -0.1743   ZZ=    24.5908
Eigenvalues:    24.5577    30.8067    34.6738
  39  H    Isotropic =    29.3305   Anisotropy =     7.6889
XX=    34.2482   YX=     0.7618   ZX=     0.6025
XY=     1.1698   YY=    28.1467   ZY=    -0.7659
XZ=     1.4549   YZ=    -3.5182   ZZ=    25.5965
Eigenvalues:    24.1913    29.3437    34.4564
  40  H    Isotropic =    28.9764   Anisotropy =     3.1933
XX=    27.2962   YX=    -0.4437   ZX=    -0.2501
XY=     1.5944   YY=    29.2379   ZY=     1.1559
XZ=    -2.6586   YZ=     0.3333   ZZ=    30.3952
Eigenvalues:    26.4788    29.3452    31.1053
  41  H    Isotropic =    29.4343   Anisotropy =     2.3301
XX=    29.2778   YX=    -1.2640   ZX=     1.1545
XY=    -2.3920   YY=    28.4040   ZY=     1.1019
XZ=     0.3259   YZ=     0.1397   ZZ=    30.6210
Eigenvalues:    26.7308    30.5843    30.9877
  42  H    Isotropic =    28.4706   Anisotropy =    10.7037
XX=    34.5016   YX=     1.8773   ZX=     0.2904
XY=     3.5426   YY=    28.8580   ZY=     1.0515
XZ=    -0.5026   YZ=     1.8121   ZZ=    22.0522
Eigenvalues:    21.7287    28.0767    35.6064
  43  H    Isotropic =    29.8549   Anisotropy =     8.5272
XX=    31.9242   YX=     1.4875   ZX=     0.3640
XY=     3.3745   YY=    33.8870   ZY=    -0.5776
XZ=     0.2958   YZ=    -0.7909   ZZ=    23.7535
Eigenvalues:    23.6760    30.3490    35.5397
  44  H    Isotropic =    29.3082   Anisotropy =     6.9391
XX=    31.1774   YX=     0.6208   ZX=     2.8386
XY=    -0.4487   YY=    28.3859   ZY=    -2.3076
XZ=     4.1749   YZ=    -2.8790   ZZ=    28.3614
Eigenvalues:    24.6288    29.3616    33.9343
  45  H    Isotropic =    29.3722   Anisotropy =     8.4806
XX=    33.1462   YX=    -0.3522   ZX=    -3.0723
XY=    -1.4516   YY=    29.1553   ZY=     1.4993
XZ=    -3.9766   YZ=     2.6425   ZZ=    25.8150
Eigenvalues:    23.8954    29.1952    35.0259
  46  H    Isotropic =    27.8926   Anisotropy =     8.9922
XX=    33.1264   YX=     1.0999   ZX=     0.9007
XY=     3.2154   YY=    27.1875   ZY=     0.0595
XZ=     0.7986   YZ=    -0.1553   ZZ=    23.3639
Eigenvalues:    23.2747    26.5157    33.8874
  47  H    Isotropic =    28.1684   Anisotropy =     8.1302
XX=    28.2530   YX=     3.4375   ZX=    -2.1063
XY=     1.3631   YY=    29.3897   ZY=    -3.9348
XZ=    -1.8902   YZ=    -2.9029   ZZ=    26.8625
```

```
Eigenvalues:    24.4522    26.4644    33.5885
  48  H    Isotropic =    28.1713   Anisotropy =     8.0574
XX=    27.5153   YX=     3.0758   ZX=     1.7083
XY=     0.8365   YY=    30.3674   ZY=     3.7038
XZ=     1.9009   YZ=     3.1718   ZZ=    26.6313
Eigenvalues:    24.4496    26.5214    33.5429
  49  H    Isotropic =    30.6455   Anisotropy =     6.9216
XX=    33.4565   YX=    -1.8354   ZX=    -0.1127
XY=    -2.3532   YY=    31.4297   ZY=     1.6361
XZ=    -1.5020   YZ=     3.1132   ZZ=    27.0502
Eigenvalues:    26.0083    30.6683    35.2599
  50  H    Isotropic =    30.5882   Anisotropy =     9.0934
XX=    30.2629   YX=     2.0287   ZX=     1.7979
XY=     2.0423   YY=    29.7907   ZY=     3.7940
XZ=     2.8878   YZ=     4.7810   ZZ=    31.7109
Eigenvalues:    26.3516    28.7625    36.6504
  51  H    Isotropic =    30.7744   Anisotropy =     4.8303
XX=    29.9846   YX=     3.5350   ZX=    -0.9623
XY=     2.6443   YY=    30.5825   ZY=    -0.7383
XZ=    -1.1938   YZ=    -0.4361   ZZ=    31.7562
Eigenvalues:    27.1425    31.1861    33.9946
  52  H    Isotropic =    31.3800   Anisotropy =    14.9508
XX=    29.3903   YX=     1.5306   ZX=    -7.7886
XY=     1.9775   YY=    29.4177   ZY=    -3.0874
XZ=    -7.7126   YZ=    -1.4409   ZZ=    35.3322
Eigenvalues:    24.0553    28.7376    41.3472
  53  H    Isotropic =    30.2726   Anisotropy =     7.3238
XX=    32.6673   YX=    -0.7978   ZX=    -4.2760
XY=    -1.0803   YY=    29.3997   ZY=     2.9653
XZ=    -1.4002   YZ=     3.1633   ZZ=    28.7509
Eigenvalues:    25.6284    30.0344    35.1552
  54  H    Isotropic =    30.9075   Anisotropy =     6.2575
XX=    30.5058   YX=     0.3807   ZX=     1.8005
XY=     0.3832   YY=    34.1931   ZY=    -2.8565
XZ=     1.8733   YZ=    -2.0963   ZZ=    28.0236
Eigenvalues:    26.3242    31.3191    35.0791
  55  H    Isotropic =    30.7385   Anisotropy =     8.8674
XX=    34.5455   YX=     2.5987   ZX=     2.9733
XY=     1.1932   YY=    28.9500   ZY=     1.2628
XZ=     3.1977   YZ=     2.2139   ZZ=    28.7199
Eigenvalues:    26.8827    28.6826    36.6501
  56  H    Isotropic =    28.2364   Anisotropy =    10.5175
XX=    34.4100   YX=    -1.7762   ZX=     0.8359
XY=    -2.7328   YY=    28.0561   ZY=     0.0410
XZ=     2.1545   YZ=     1.1762   ZZ=    22.2432
Eigenvalues:    21.9291    27.5321    35.2481
  57  H    Isotropic =    29.4575   Anisotropy =     5.5351
XX=    29.8325   YX=    -3.6672   ZX=    -1.7531
XY=    -2.7969   YY=    29.2573   ZY=    -0.7466
XZ=    -2.5437   YZ=    -0.7389   ZZ=    29.2828
Eigenvalues:    25.2658    29.9592    33.1476
  58  H    Isotropic =    29.7450   Anisotropy =     8.5153
XX=    35.3837   YX=    -0.0599   ZX=     0.3202
XY=    -0.3512   YY=    27.7121   ZY=     0.0306
XZ=     0.7690   YZ=    -0.4788   ZZ=    26.1391
Eigenvalues:    26.0796    27.7335    35.4218
  59  H    Isotropic =    30.1553   Anisotropy =     9.4743
XX=    29.2040   YX=    -1.9347   ZX=    -4.7650
XY=    -2.1188   YY=    29.0251   ZY=     1.8074
XZ=    -4.3209   YZ=     1.8550   ZZ=    32.2368
Eigenvalues:    25.7988    28.1956    36.4715
  60  H    Isotropic =    29.9751   Anisotropy =    11.4731
XX=    26.6113   YX=     0.7855   ZX=    -0.6057
XY=     0.9755   YY=    35.9113   ZY=     4.3095
XZ=    -0.5508   YZ=     3.9647   ZZ=    27.4028
Eigenvalues:    25.1849    27.1166    37.6239
  61  H    Isotropic =    29.9889   Anisotropy =    11.8254
XX=    29.6344   YX=    -4.6180   ZX=     2.0556
XY=    -4.7852   YY=    31.9943   ZY=    -4.3678
XZ=     1.9444   YZ=    -4.1030   ZZ=    28.3378
Eigenvalues:    25.1159    26.9782    37.8724
  62  H    Isotropic =    29.9083   Anisotropy =     8.0466
XX=    33.9882   YX=     2.1015   ZX=     0.6713
XY=     2.7417   YY=    30.3265   ZY=     0.8174
XZ=     0.7295   YZ=     0.3431   ZZ=    25.4103
Eigenvalues:    25.3196    29.1327    35.2727
  63  H    Isotropic =    30.1990   Anisotropy =     8.4252
XX=    28.0173   YX=     0.8017   ZX=    -2.9670
XY=     0.4645   YY=    30.7074   ZY=    -3.8110
```

```
XZ=    -2.4900   YZ=    -3.5215   ZZ=    31.8723
Eigenvalues:    26.1518    28.6295    35.8159
  64  H    Isotropic =    30.5508   Anisotropy =     8.4455
XX=    28.9129   YX=     3.8499   ZX=     3.6697
XY=     3.1430   YY=    34.3276   ZY=    -0.7382
XZ=     3.1731   YZ=    -0.4981   ZZ=    28.4118
Eigenvalues:    24.3742    31.0970    36.1811
  65  H    Isotropic =    30.7782   Anisotropy =     8.5857
XX=    32.9706   YX=    -3.7384   ZX=     3.7714
XY=    -2.4351   YY=    28.9516   ZY=     0.9924
XZ=     4.0258   YZ=    -0.0643   ZZ=    30.4125
Eigenvalues:    25.7929    30.0398    36.5020
  66  H    Isotropic =    31.3150   Anisotropy =     9.5697
XX=    28.1106   YX=    -0.4218   ZX=     0.8971
XY=    -0.1924   YY=    28.1770   ZY=    -0.3131
XZ=    -1.0392   YZ=    -0.8761   ZZ=    37.6574
Eigenvalues:    27.8138    28.4363    37.6948
  67  H    Isotropic =    30.9936   Anisotropy =     9.3942
XX=    28.5935   YX=    -0.1148   ZX=    -0.7182
XY=    -0.6357   YY=    27.4593   ZY=     0.5100
XZ=    -2.1691   YZ=     1.2175   ZZ=    36.9281
Eigenvalues:    27.3244    28.4001    37.2564
  68  H    Isotropic =    30.6369   Anisotropy =     8.5355
XX=    32.3511   YX=    -5.0470   ZX=     1.2633
XY=    -4.8429   YY=    29.8471   ZY=     0.7827
XZ=     2.7445   YZ=     1.4809   ZZ=    29.7124
Eigenvalues:    25.0218    30.5617    36.3272
  69  H    Isotropic =    30.5598   Anisotropy =     7.4813
XX=    29.6697   YX=     2.6370   ZX=     2.4786
XY=     2.4186   YY=    31.9639   ZY=     1.3488
XZ=     2.8538   YZ=     2.9045   ZZ=    30.0459
Eigenvalues:    27.1289    29.0032    35.5474
  70  H    Isotropic =    30.6395   Anisotropy =     6.9755
XX=    31.5081   YX=    -3.7370   ZX=    -0.0760
XY=    -3.9682   YY=    30.2950   ZY=     1.1424
XZ=    -1.5197   YZ=     1.8608   ZZ=    30.1153
Eigenvalues:    26.8691    29.7595    35.2898
  71  H    Isotropic =    31.0427   Anisotropy =     8.9025
XX=    31.3619   YX=     1.8594   ZX=    -1.6810
XY=     2.5648   YY=    31.9928   ZY=    -3.7826
XZ=    -1.9069   YZ=    -5.2498   ZZ=    29.7735
Eigenvalues:    26.2321    29.9183    36.9777
  72  H    Isotropic =    30.3675   Anisotropy =     6.0273
XX=    32.7022   YX=    -0.7170   ZX=     1.8205
XY=    -0.5132   YY=    25.7017   ZY=     0.2137
XZ=     1.3290   YZ=    -1.7538   ZZ=    32.6987
Eigenvalues:    25.5891    31.1278    34.3857
```

Chemical Shielding tensors (GIAO MPW1PW91/6-311+G(2d,p) iefpcm = DMSO)
3S*,7S*,11R*-1b

Conf 1 (7 %)

```
   1  C    Isotropic =    72.9906   Anisotropy =   131.2719
XX=    28.1905   YX=     6.9320   ZX=    47.7567
XY=     4.0834   YY=   159.4458   ZY=   -11.9783
XZ=    26.2074   YZ=   -10.9652   ZZ=    31.3355
Eigenvalues:    -8.1010    66.5675   160.5052
   2  C    Isotropic =    27.5613   Anisotropy =   128.0513
XX=   -41.9334   YX=     2.1406   ZX=   -22.3357
XY=     3.0093   YY=   112.4653   ZY=    -5.1043
XZ=   -24.3616   YZ=    -6.9132   ZZ=    12.1522
Eigenvalues:   -50.6190    20.3742   112.9289
   3  C    Isotropic =    67.3117   Anisotropy =   126.7950
XX=    60.2138   YX=     2.0452   ZX=   -18.3224
XY=     8.1704   YY=   150.5403   ZY=   -12.3602
XZ=    -9.8400   YZ=   -11.4578   ZZ=    -8.8190
Eigenvalues:   -12.2874    62.3808   151.8417
   4  C    Isotropic =    60.4741   Anisotropy =   169.0697
XX=     2.8342   YX=     6.2143   ZX=    28.7032
XY=    -8.6523   YY=   171.9420   ZY=   -17.1251
XZ=    17.8055   YZ=   -10.9683   ZZ=     6.6462
Eigenvalues:   -18.9869    27.2221   173.1872
   5  C    Isotropic =    35.3477   Anisotropy =   118.5155
XX=   -36.0729   YX=     3.1224   ZX=   -23.7519
XY=     3.8634   YY=   112.8033   ZY=   -14.1794
XZ=   -29.5829   YZ=    -6.3412   ZZ=    29.3128
Eigenvalues:   -45.5700    37.2551   114.3580
   6  C    Isotropic =    53.9711   Anisotropy =   164.5802
XX=    23.7079   YX=     3.1901   ZX=   -14.1665
XY=     4.1421   YY=   162.6891   ZY=   -10.5334
XZ=    -6.5571   YZ=   -14.9870   ZZ=   -24.4836
Eigenvalues:   -27.3474    25.5695   163.6913
   7  C    Isotropic =   160.7803   Anisotropy =    19.6771
XX=   172.2021   YX=     3.7933   ZX=    -2.7574
XY=    -2.2834   YY=   154.3892   ZY=    -3.4616
XZ=    -7.8842   YZ=    -2.3792   ZZ=   155.7497
Eigenvalues:   151.6421   156.8005   173.8984
   8  C    Isotropic =   152.7907   Anisotropy =    17.9105
XX=   142.9096   YX=    -0.5064   ZX=     3.8434
XY=    -4.3113   YY=   159.8369   ZY=     0.2239
XZ=     7.1170   YZ=   -10.9760   ZZ=   155.6257
Eigenvalues:   140.8650   152.7761   164.7311
   9  C    Isotropic =   104.2271   Anisotropy =    50.4422
XX=   124.4600   YX=   -10.9446   ZX=   -24.8002
XY=    -2.5694   YY=    95.5303   ZY=     8.2885
XZ=   -19.7634   YZ=     4.7429   ZZ=    92.6910
Eigenvalues:    80.6683    94.1577   137.8552
  10  O    Isotropic =   180.8129   Anisotropy =   109.1538
XX=   157.2904   YX=     2.0502   ZX=     2.6631
XY=    24.0345   YY=   249.0785   ZY=    35.0782
XZ=    15.2065   YZ=    -1.7564   ZZ=   136.0699
Eigenvalues:   131.6331   157.2235   253.5821
  11  C    Isotropic =   145.9038   Anisotropy =    46.4817
XX=   131.3729   YX=     9.0020   ZX=    12.5997
XY=     8.5324   YY=   166.8095   ZY=     7.8033
XZ=    15.9530   YZ=    19.9250   ZZ=   139.5290
Eigenvalues:   120.5546   140.2651   176.8916
  12  C    Isotropic =    41.8622   Anisotropy =   143.1850
XX=    -4.7451   YX=    59.3965   ZX=    30.7615
XY=    58.6739   YY=    22.8409   ZY=    37.0249
XZ=    26.3899   YZ=    32.3041   ZZ=   107.4908
Eigenvalues:   -51.5822    39.8500   137.3189
  13  C    Isotropic =    26.5364   Anisotropy =   178.2762
XX=   -40.4106   YX=    63.7277   ZX=    40.9423
XY=    53.3749   YY=     4.7847   ZY=    47.5316
XZ=    42.8259   YZ=    26.6637   ZZ=   115.2351
Eigenvalues:   -81.6321    15.8541   145.3872
  14  C    Isotropic =   136.5662   Anisotropy =    28.7740
XX=   151.7497   YX=     2.9101   ZX=     2.3218
XY=     5.4895   YY=   120.6128   ZY=    12.4337
XZ=     9.6284   YZ=     9.1600   ZZ=   137.3360
Eigenvalues:   115.2807   138.6690   155.7488
  15  C    Isotropic =   167.1678   Anisotropy =    26.8855
XX=   161.6937   YX=    -0.5051   ZX=    -0.3203
XY=     0.2451   YY=   154.9051   ZY=     2.5423
XZ=     1.2640   YZ=     2.0899   ZZ=   184.9047
Eigenvalues:   154.7234   161.6886   185.0915
```

```
  16  O    Isotropic =   235.9280   Anisotropy =    77.7967
XX=   227.3034   YX=     5.0922   ZX=    48.3591
XY=     7.1671   YY=   273.5969   ZY=    -4.9685
XZ=    91.5208   YZ=    -7.7080   ZZ=   206.8836
Eigenvalues:   145.8057   274.1857   287.7924
  17  C    Isotropic =   132.1216   Anisotropy =    72.5819
XX=   158.3607   YX=    -4.2172   ZX=   -36.6787
XY=    -3.7148   YY=   112.0061   ZY=     2.2403
XZ=   -32.1621   YZ=     2.0682   ZZ=   125.9980
Eigenvalues:   104.1327   111.7225   180.5096
  18  C    Isotropic =   110.1857   Anisotropy =    46.5007
XX=   141.0179   YX=     4.4022   ZX=     1.1491
XY=     0.6379   YY=   100.4427   ZY=    -3.0440
XZ=     0.7334   YZ=    -1.4923   ZZ=    89.0965
Eigenvalues:    88.6221   100.7488   141.1861
  19  C    Isotropic =   160.6772   Anisotropy =    35.8992
XX=   159.7117   YX=     4.1313   ZX=    -9.4518
XY=    -1.6231   YY=   142.7229   ZY=     1.9599
XZ=   -12.7509   YZ=     2.4097   ZZ=   179.5971
Eigenvalues:   142.3368   155.0848   184.6100
  20  O    Isotropic =   235.9657   Anisotropy =    66.8435
XX=   235.2695   YX=    -2.6543   ZX=   -27.9421
XY=    -1.6804   YY=   222.0672   ZY=    11.1308
XZ=   -41.0947   YZ=    14.7154   ZZ=   250.5603
Eigenvalues:   204.8982   222.4708   280.5280
  21  C    Isotropic =   153.5373   Anisotropy =    50.4107
XX=   139.9039   YX=   -10.8377   ZX=     4.3275
XY=    -9.8936   YY=   172.0811   ZY=   -21.0807
XZ=     6.3058   YZ=   -20.6290   ZZ=   148.6269
Eigenvalues:   136.0371   137.4304   187.1444
  22  C    Isotropic =   -29.4743   Anisotropy =   189.9943
XX=   -88.6835   YX=    48.2534   ZX=    49.2280
XY=    51.4804   YY=    -2.2485   ZY=    64.2084
XZ=    51.8318   YZ=    75.6016   ZZ=     2.5092
Eigenvalues:  -115.8062   -69.8052    97.1886
  23  C    Isotropic =   126.4057   Anisotropy =    30.2007
XX=   126.0788   YX=    -0.8621   ZX=    -0.5111
XY=   -15.3789   YY=   143.2759   ZY=    -4.2967
XZ=    -3.0420   YZ=     0.4495   ZZ=   109.8624
Eigenvalues:   109.4129   123.2648   146.5395
  24  C    Isotropic =   135.7865   Anisotropy =    27.4978
XX=   152.8127   YX=    -5.1741   ZX=   -10.4730
XY=     1.0940   YY=   140.1431   ZY=   -13.2271
XZ=    -3.9293   YZ=    -9.1304   ZZ=   114.4038
Eigenvalues:   108.9575   144.2836   154.1184
  25  C    Isotropic =   141.0758   Anisotropy =    36.3967
XX=   147.4146   YX=   -19.1582   ZX=    -4.8060
XY=   -20.0413   YY=   137.8634   ZY=     9.8003
XZ=    -2.7282   YZ=     7.3262   ZZ=   137.9493
Eigenvalues:   121.1825   136.7046   165.3403
  26  C    Isotropic =   154.8035   Anisotropy =    31.9979
XX=   161.5023   YX=    -7.9243   ZX=   -15.7745
XY=   -13.6036   YY=   163.9994   ZY=     3.5176
XZ=   -13.6736   YZ=    -5.7329   ZZ=   138.9089
Eigenvalues:   130.5864   157.6888   176.1355
  27  C    Isotropic =   164.9916   Anisotropy =    28.2546
XX=   174.2061   YX=    -1.7476   ZX=   -13.3480
XY=    -0.9280   YY=   174.8185   ZY=   -13.9528
XZ=   -13.8237   YZ=   -15.5121   ZZ=   145.9502
Eigenvalues:   135.3416   175.8052   183.8280
  28  C    Isotropic =   149.0906   Anisotropy =    43.9282
XX=   164.1515   YX=   -19.4963   ZX=     3.9047
XY=   -15.9370   YY=   150.2206   ZY=   -15.1347
XZ=    -2.3764   YZ=   -16.2290   ZZ=   132.8997
Eigenvalues:   121.7089   147.1868   178.3761
  29  C    Isotropic =   164.4845   Anisotropy =    36.8112
XX=   156.4299   YX=     9.0810   ZX=    13.4117
XY=     9.1328   YY=   159.2596   ZY=     7.9292
XZ=    14.2442   YZ=     8.5811   ZZ=   177.7640
Eigenvalues:   147.2273   157.2009   189.0253
  30  C    Isotropic =   164.4863   Anisotropy =    31.6313
XX=   163.0902   YX=     1.3179   ZX=     5.7042
XY=     3.0609   YY=   153.0975   ZY=    14.2157
XZ=     0.1173   YZ=    17.1993   ZZ=   177.2712
Eigenvalues:   145.3383   162.5468   185.5738
  31  O    Isotropic =  -259.0213   Anisotropy =   926.2061
XX=  -373.0574   YX=    31.0336   ZX=   340.3430
XY=    61.4104   YY=  -156.0949   ZY=   439.6422
XZ=   329.2575   YZ=   481.0239   ZZ=  -247.9117
```

```
Eigenvalues:  -795.8846  -339.6287   358.4494
  32  C    Isotropic =   163.2036   Anisotropy =    26.7606
XX=   150.2539   YX=    -1.1292   ZX=    -7.8677
XY=    -9.1912   YY=   180.0633   ZY=    -5.6740
XZ=    -5.3462   YZ=     0.3886   ZZ=   159.2936
Eigenvalues:   145.7882   162.7786   181.0440
  33  H    Isotropic =    25.0921   Anisotropy =     9.0695
XX=    27.9354   YX=     0.0865   ZX=     3.6148
XY=     0.0520   YY=    20.3678   ZY=     0.4575
XZ=     3.6702   YZ=     0.3809   ZZ=    26.9731
Eigenvalues:    20.3367    23.8012    31.1384
  34  H    Isotropic =    24.9200   Anisotropy =     8.7695
XX=    30.7223   YX=    -0.3365   ZX=    -0.3083
XY=    -0.6342   YY=    20.4584   ZY=    -0.1556
XZ=    -0.4719   YZ=     0.1958   ZZ=    23.5794
Eigenvalues:    20.4355    23.5581    30.7664
  35  H    Isotropic =    29.0697   Anisotropy =     9.4265
XX=    29.9918   YX=    -0.1422   ZX=     1.8506
XY=     0.4084   YY=    24.5483   ZY=    -5.1110
XZ=     2.8994   YZ=    -3.4070   ZZ=    32.6692
Eigenvalues:    22.5657    29.2895    35.3541
  36  H    Isotropic =    28.9740   Anisotropy =     7.1126
XX=    28.9868   YX=    -0.0239   ZX=     1.0453
XY=    -1.3372   YY=    29.0185   ZY=     5.3489
XZ=     1.9607   YZ=     4.0000   ZZ=    28.9167
Eigenvalues:    23.8260    29.3802    33.7157
  37  H    Isotropic =    29.9215   Anisotropy =     8.5879
XX=    30.6008   YX=     2.5143   ZX=     2.7236
XY=     4.8417   YY=    32.9651   ZY=    -1.6918
XZ=     2.7832   YZ=    -2.1718   ZZ=    26.1986
Eigenvalues:    23.5876    30.5301    35.6467
  38  H    Isotropic =    30.0557   Anisotropy =     7.3685
XX=    34.7609   YX=     0.9562   ZX=    -1.0153
XY=     1.0982   YY=    25.1726   ZY=     0.5356
XZ=    -0.3996   YZ=    -0.1216   ZZ=    30.2335
Eigenvalues:    25.0485    30.1504    34.9680
  39  H    Isotropic =    29.3415   Anisotropy =    11.2485
XX=    33.2211   YX=     4.7344   ZX=     1.3036
XY=     4.7815   YY=    30.3802   ZY=    -0.7467
XZ=     2.8347   YZ=    -1.6528   ZZ=    24.4233
Eigenvalues:    23.0736    28.1104    36.8405
  40  H    Isotropic =    28.5672   Anisotropy =     3.5043
XX=    26.6054   YX=    -1.1759   ZX=     0.2288
XY=     0.2818   YY=    28.4177   ZY=     0.2747
XZ=    -1.6328   YZ=    -1.4582   ZZ=    30.6784
Eigenvalues:    26.3438    28.4543    30.9034
  41  H    Isotropic =    29.3484   Anisotropy =     3.3579
XX=    31.0941   YX=    -0.0638   ZX=     0.0234
XY=    -1.3529   YY=    25.5152   ZY=     0.9925
XZ=    -0.5616   YZ=    -1.4725   ZZ=    31.4358
Eigenvalues:    25.4144    31.0437    31.5870
  42  H    Isotropic =    28.6409   Anisotropy =     8.0503
XX=    30.0816   YX=     2.7316   ZX=    -0.4252
XY=     3.6397   YY=    31.3742   ZY=     0.9025
XZ=    -1.6032   YZ=     2.0891   ZZ=    24.4670
Eigenvalues:    23.6562    28.2588    34.0078
  43  H    Isotropic =    29.9381   Anisotropy =     8.9347
XX=    33.0958   YX=    -0.2588   ZX=    -2.1218
XY=    -0.4016   YY=    24.3602   ZY=     0.5480
XZ=    -4.0999   YZ=     0.6184   ZZ=    32.3583
Eigenvalues:    24.3165    29.6033    35.8946
  44  H    Isotropic =    29.3475   Anisotropy =     6.9015
XX=    29.6706   YX=    -2.7267   ZX=    -0.6044
XY=    -3.5009   YY=    27.5047   ZY=    -4.1490
XZ=     0.6612   YZ=    -2.9848   ZZ=    30.8673
Eigenvalues:    23.9733    30.1207    33.9486
  45  H    Isotropic =    29.3620   Anisotropy =     7.1965
XX=    29.7388   YX=     2.4607   ZX=    -0.6336
XY=     3.3687   YY=    28.8230   ZY=     4.7157
XZ=     0.3028   YZ=     3.4546   ZZ=    29.5241
Eigenvalues:    24.1036    29.8228    34.1596
  46  H    Isotropic =    27.9007   Anisotropy =     8.9827
XX=    33.8241   YX=    -0.1434   ZX=     1.7201
XY=    -0.2903   YY=    23.3515   ZY=     0.1904
XZ=    -0.3753   YZ=     0.2447   ZZ=    26.5266
Eigenvalues:    23.3300    26.4830    33.8892
  47  H    Isotropic =    28.1673   Anisotropy =     8.3097
XX=    29.9460   YX=     2.9200   ZX=    -3.8058
XY=     2.7755   YY=    26.3036   ZY=    -1.8681
```

```
XZ=    -1.5980   YZ=    -2.6284   ZZ=    28.2525
Eigenvalues:    24.5202    26.2747    33.7072
  48  H    Isotropic =    28.1685   Anisotropy =     8.2453
XX=    29.5929   YX=    -3.6034   ZX=    -3.1847
XY=    -3.0958   YY=    27.4145   ZY=     2.1713
XZ=    -0.9922   YZ=     2.8348   ZZ=    27.4980
Eigenvalues:    24.5782    26.2619    33.6653
  49  H    Isotropic =    30.6215   Anisotropy =     4.4641
XX=    26.8908   YX=     0.3399   ZX=     0.2243
XY=    -0.5459   YY=    33.0950   ZY=    -0.2987
XZ=     0.3756   YZ=    -1.5407   ZZ=    31.8788
Eigenvalues:    26.8724    31.3946    33.5976
  50  H    Isotropic =    30.6872   Anisotropy =     9.1000
XX=    27.7238   YX=     1.9195   ZX=     0.6250
XY=     1.4159   YY=    32.1657   ZY=     3.4761
XZ=     0.9406   YZ=     5.0258   ZZ=    32.1721
Eigenvalues:    26.9851    28.3227    36.7539
  51  H    Isotropic =    31.0836   Anisotropy =     7.3600
XX=    34.5528   YX=    -0.1188   ZX=    -2.4885
XY=    -0.3221   YY=    29.9211   ZY=     1.1112
XZ=    -3.7404   YZ=     1.2849   ZZ=    28.7768
Eigenvalues:    27.0550    30.2055    35.9903
  52  H    Isotropic =    30.6022   Anisotropy =    15.6032
XX=    31.6264   YX=    -1.8107   ZX=    -7.5571
XY=    -2.6400   YY=    28.6277   ZY=     4.1575
XZ=    -6.2015   YZ=     7.0272   ZZ=    31.5525
Eigenvalues:    23.0938    27.7085    41.0043
  53  H    Isotropic =    30.5933   Anisotropy =     7.8110
XX=    27.6015   YX=    -0.0339   ZX=    -1.1525
XY=     0.9004   YY=    31.0557   ZY=    -4.3359
XZ=    -1.9885   YZ=    -2.1987   ZZ=    33.1228
Eigenvalues:    27.1100    28.8694    35.8007
  54  H    Isotropic =    30.6446   Anisotropy =     7.8248
XX=    33.2555   YX=    -2.2097   ZX=     1.5098
XY=    -3.0407   YY=    31.3012   ZY=    -2.1328
XZ=     1.9623   YZ=    -1.8613   ZZ=    27.3772
Eigenvalues:    26.4723    29.6004    35.8612
  55  H    Isotropic =    30.4306   Anisotropy =     9.2463
XX=    26.8858   YX=     0.6230   ZX=     0.2254
XY=     1.6977   YY=    36.4259   ZY=     1.1162
XZ=    -0.0361   YZ=    -0.1179   ZZ=    27.9802
Eigenvalues:    26.7457    27.9513    36.5948
  56  H    Isotropic =    29.1227   Anisotropy =     3.9456
XX=    30.2291   YX=    -1.2863   ZX=    -0.5721
XY=    -0.5453   YY=    25.5221   ZY=     0.4716
XZ=    -0.3184   YZ=    -0.6373   ZZ=    31.6171
Eigenvalues:    25.3459    30.2692    31.7532
  57  H    Isotropic =    29.0975   Anisotropy =    10.2621
XX=    35.8567   YX=     0.1498   ZX=    -0.7490
XY=     0.7989   YY=    27.7285   ZY=    -1.2108
XZ=    -0.8122   YZ=    -0.0389   ZZ=    23.7071
Eigenvalues:    23.5721    27.7815    35.9389
  58  H    Isotropic =    29.6743   Anisotropy =     8.3546
XX=    27.9560   YX=     0.0366   ZX=    -3.1257
XY=    -0.7666   YY=    27.2455   ZY=    -0.6109
XZ=    -3.1824   YZ=    -1.0609   ZZ=    33.8215
Eigenvalues:    26.1553    27.6236    35.2440
  59  H    Isotropic =    30.2567   Anisotropy =     9.3359
XX=    34.9088   YX=     2.9484   ZX=    -1.2595
XY=     2.9719   YY=    29.9335   ZY=    -0.2826
XZ=    -1.4021   YZ=    -0.7603   ZZ=    25.9279
Eigenvalues:    25.7323    28.5572    36.4806
  60  H    Isotropic =    29.9927   Anisotropy =    11.7345
XX=    34.8859   YX=    -2.8396   ZX=     3.9987
XY=    -3.0794   YY=    27.5003   ZY=    -2.3906
XZ=     3.6895   YZ=    -2.1639   ZZ=    27.5919
Eigenvalues:    25.1971    26.9652    37.8157
  61  H    Isotropic =    30.0160   Anisotropy =    11.7450
XX=    29.0340   YX=    -3.8688   ZX=    -3.5025
XY=    -3.7600   YY=    32.7305   ZY=     3.9382
XZ=    -3.2601   YZ=     3.8364   ZZ=    28.2834
Eigenvalues:    25.2178    26.9842    37.8459
  62  H    Isotropic =    29.9286   Anisotropy =     7.9284
XX=    28.1816   YX=    -1.1109   ZX=     1.0099
XY=    -1.3694   YY=    28.0207   ZY=    -3.5740
XZ=     0.3089   YZ=    -2.8094   ZZ=    33.5835
Eigenvalues:    26.1760    28.3956    35.2142
  63  H    Isotropic =    30.2649   Anisotropy =     8.6030
XX=    29.1349   YX=     0.8915   ZX=    -0.2503
```

```
XY=     0.8762   YY=    35.7102   ZY=    -1.0363
XZ=    -0.2644   YZ=    -1.5576   ZZ=    25.9495
Eigenvalues:    25.7741    29.0204    36.0002
  64  H    Isotropic =    30.7717   Anisotropy =     6.1804
XX=    30.1338   YX=    -2.5932   ZX=     2.5540
XY=    -2.8973   YY=    30.1641   ZY=     0.1125
XZ=     3.5132   YZ=    -0.0515   ZZ=    32.0173
Eigenvalues:    26.4376    30.9857    34.8920
  65  H    Isotropic =    30.7624   Anisotropy =     8.3652
XX=    31.1937   YX=    -0.4202   ZX=    -0.9290
XY=    -1.0960   YY=    27.6034   ZY=     3.9276
XZ=    -2.0613   YZ=     4.7586   ZZ=    33.4900
Eigenvalues:    25.3000    30.6479    36.3392
  66  H    Isotropic =    31.3404   Anisotropy =     8.9159
XX=    32.5547   YX=     3.3060   ZX=     0.7522
XY=     3.1504   YY=    33.3125   ZY=     2.1122
XZ=     1.8800   YZ=     3.9648   ZZ=    28.1540
Eigenvalues:    26.7416    29.9953    37.2843
  67  H    Isotropic =    30.8553   Anisotropy =     9.0288
XX=    31.7562   YX=     3.2403   ZX=     1.5146
XY=     4.0813   YY=    33.1155   ZY=     1.6194
XZ=     2.2391   YZ=     1.9204   ZZ=    27.6941
Eigenvalues:    26.9291    28.7622    36.8745
  68  H    Isotropic =    30.5547   Anisotropy =     6.7514
XX=    31.8044   YX=    -2.1174   ZX=    -0.3171
XY=    -1.3480   YY=    29.0672   ZY=     4.5061
XZ=    -1.8769   YZ=     3.2055   ZZ=    30.7925
Eigenvalues:    25.8970    30.7115    35.0556
  69  H    Isotropic =    30.6383   Anisotropy =     8.1431
XX=    29.6631   YX=    -2.0980   ZX=     4.7141
XY=    -0.8742   YY=    29.5462   ZY=     0.3118
XZ=     4.2544   YZ=    -0.5696   ZZ=    32.7056
Eigenvalues:    26.0696    29.7783    36.0670
  70  H    Isotropic =    30.8037   Anisotropy =     8.9077
XX=    34.1177   YX=    -2.4594   ZX=    -1.8723
XY=    -3.3430   YY=    30.7483   ZY=     2.7813
XZ=    -1.5673   YZ=     2.9014   ZZ=    27.5453
Eigenvalues:    25.8851    29.7839    36.7422
  71  H    Isotropic =    30.6558   Anisotropy =     6.9730
XX=    30.2170   YX=     0.2524   ZX=    -0.0258
XY=    -1.6069   YY=    31.4642   ZY=    -5.4559
XZ=     1.0756   YZ=    -3.0335   ZZ=    30.2863
Eigenvalues:    26.5892    30.0738    35.3045
  72  H    Isotropic =    30.0970   Anisotropy =     5.5533
XX=    32.0594   YX=     1.9564   ZX=     2.2723
XY=     0.0052   YY=    32.4676   ZY=     0.7429
XZ=     2.8169   YZ=     0.0535   ZZ=    25.7639
Eigenvalues:    24.8637    31.6280    33.7992
```

Chemical Shielding tensors (GIAO MPW1PW91/6-311+G(2d,p) iefpcm = DMSO)
3S*,7S*,11R*-1b

Conf 2 (44 %)

```
   1  C    Isotropic =    72.7942   Anisotropy =   131.1507
XX=    77.6215   YX=    51.2263   ZX=   -40.3634
XY=    33.3169   YY=    12.4616   ZY=   -19.8926
XZ=   -44.7398   YZ=    -7.6233   ZZ=   128.2995
Eigenvalues:    -8.6771    66.8317   160.2280
   2  C    Isotropic =    27.4292   Anisotropy =   128.4293
XX=    -4.0519   YX=    12.9298   ZX=   -74.1504
XY=    11.1405   YY=    24.3071   ZY=   -16.8195
XZ=   -70.8418   YZ=   -14.6167   ZZ=    62.0324
Eigenvalues:   -50.7168    19.9557   113.0487
   3  C    Isotropic =    66.8401   Anisotropy =   127.1642
XX=    75.7463   YX=   -13.4212   ZX=   -52.6655
XY=    -8.1558   YY=    15.3237   ZY=   -41.1517
XZ=   -45.8709   YZ=   -46.9084   ZZ=   109.4504
Eigenvalues:   -12.5154    61.4196   151.6163
   4  C    Isotropic =    60.2345   Anisotropy =   170.9127
XX=    66.0015   YX=    40.4901   ZX=   -61.5335
XY=    31.1655   YY=    -3.8475   ZY=   -21.1999
XZ=   -77.6534   YZ=   -17.6370   ZZ=   118.5495
Eigenvalues:   -19.9120    26.4392   174.1763
   5  C    Isotropic =    35.6034   Anisotropy =   120.2680
XX=     1.5728   YX=    18.9536   ZX=   -68.2089
XY=    10.2570   YY=    42.6410   ZY=   -14.0272
XZ=   -74.2408   YZ=   -17.7511   ZZ=    62.5966
Eigenvalues:   -45.5397    36.5679   115.7821
   6  C    Isotropic =    54.3634   Anisotropy =   164.2358
XX=    59.6327   YX=    -1.5742   ZX=   -69.7538
XY=     6.7026   YY=    -2.4198   ZY=   -45.4516
XZ=   -66.2480   YZ=   -46.3198   ZZ=   105.8773
Eigenvalues:   -26.8008    26.0371   163.8539
   7  C    Isotropic =   161.1648   Anisotropy =    18.9875
XX=   163.9655   YX=    -4.6179   ZX=     7.5928
XY=    -7.0025   YY=   164.6872   ZY=    -9.2116
XZ=     0.5765   YZ=    -5.8471   ZZ=   154.8416
Eigenvalues:   150.7033   158.9679   173.8231
   8  C    Isotropic =   148.6942   Anisotropy =    28.6410
XX=   152.2971   YX=    10.0266   ZX=    -6.3441
XY=    19.2709   YY=   152.8724   ZY=    -1.3439
XZ=    -8.8559   YZ=     5.7934   ZZ=   140.9132
Eigenvalues:   132.1222   146.1722   167.7882
   9  C    Isotropic =   105.3565   Anisotropy =    51.0655
XX=   107.5466   YX=   -28.5356   ZX=    -9.3972
XY=   -27.3104   YY=   114.7730   ZY=    -1.5687
XZ=    -1.6078   YZ=    -2.9055   ZZ=    93.7499
Eigenvalues:    80.5990    96.0703   139.4001
  10  O    Isotropic =   185.0924   Anisotropy =   117.7883
XX=   173.0957   YX=    -3.0827   ZX=   -53.2036
XY=    18.6420   YY=   131.0070   ZY=   -17.5811
XZ=    -9.6759   YZ=    -5.3741   ZZ=   251.1746
Eigenvalues:   129.2759   162.3834   263.6180
  11  C    Isotropic =   144.0494   Anisotropy =    47.4788
XX=   124.1367   YX=   -13.9194   ZX=    -5.6232
XY=   -18.9046   YY=   148.0494   ZY=    10.2581
XZ=   -15.4306   YZ=    17.9595   ZZ=   159.9620
Eigenvalues:   115.5471   140.8992   175.7019
  12  C    Isotropic =    44.6440   Anisotropy =   134.9740
XX=    41.4976   YX=   -21.6911   ZX=    -5.8656
XY=   -20.8730   YY=   -37.9608   ZY=    28.3127
XZ=     1.2285   YZ=    23.5442   ZZ=   130.3952
Eigenvalues:   -46.7292    46.0346   134.6267
  13  C    Isotropic =    22.6254   Anisotropy =   184.4614
XX=    -1.9670   YX=   -38.1037   ZX=     0.3852
XY=   -21.8612   YY=   -73.5927   ZY=    26.5015
XZ=     9.3145   YZ=    16.8042   ZZ=   143.4358
Eigenvalues:   -86.5927     8.8692   145.5996
  14  C    Isotropic =   144.4821   Anisotropy =    25.0849
XX=   142.9237   YX=     7.0974   ZX=    -9.2097
XY=     7.9927   YY=   146.6409   ZY=     3.9892
XZ=   -21.4298   YZ=    -5.6780   ZZ=   143.8816
Eigenvalues:   126.8301   145.4108   161.2053
  15  C    Isotropic =   166.7238   Anisotropy =    26.6212
XX=   162.7020   YX=     2.2627   ZX=     4.2859
XY=     3.9720   YY=   178.3798   ZY=    11.6054
XZ=     2.7302   YZ=    11.0619   ZZ=   159.0896
Eigenvalues:   153.4450   162.2551   184.4713
```

```
  16  O    Isotropic =   235.5560   Anisotropy =    77.5734
XX=   261.9081   YX=    31.6169   ZX=     0.4551
XY=    67.8029   YY=   166.1133   ZY=    14.5559
XZ=     9.6404   YZ=    -7.4463   ZZ=   278.6467
Eigenvalues:   144.9674   274.4291   287.2716
  17  C    Isotropic =   132.0812   Anisotropy =    72.4993
XX=   133.3046   YX=   -36.4859   ZX=     3.7023
XY=   -32.8627   YY=   151.7952   ZY=   -10.2552
XZ=     4.8797   YZ=   -12.6407   ZZ=   111.1437
Eigenvalues:   104.1757   111.6538   180.4141
  18  C    Isotropic =   108.9294   Anisotropy =    52.8958
XX=    99.5641   YX=   -20.3030   ZX=     4.9463
XY=   -18.9633   YY=   130.6144   ZY=   -15.1750
XZ=     2.9632   YZ=   -11.9017   ZZ=    96.6095
Eigenvalues:    88.7295    93.8653   144.1933
  19  C    Isotropic =   156.4194   Anisotropy =    37.7901
XX=   167.0124   YX=    15.0782   ZX=   -12.0283
XY=    11.3534   YY=   150.7279   ZY=    -5.6270
XZ=   -15.3073   YZ=    -5.8160   ZZ=   151.5178
Eigenvalues:   142.2463   145.3990   181.6128
  20  O    Isotropic =   222.1789   Anisotropy =    70.6573
XX=   218.5231   YX=   -23.4289   ZX=    27.4043
XY=   -16.9697   YY=   234.1060   ZY=   -17.0779
XZ=    27.2778   YZ=   -27.5919   ZZ=   213.9077
Eigenvalues:   188.5142   208.7388   269.2838
  21  C    Isotropic =   159.5263   Anisotropy =    35.7365
XX=   170.9492   YX=     9.4492   ZX=    10.6715
XY=    12.4224   YY=   148.0460   ZY=     4.2258
XZ=    13.7707   YZ=     8.9100   ZZ=   159.5837
Eigenvalues:   143.4275   151.8008   183.3506
  22  C    Isotropic =   -25.0965   Anisotropy =   182.8467
XX=   -65.1357   YX=   -36.3623   ZX=   -31.9013
XY=   -40.6826   YY=   -75.3649   ZY=    50.3197
XZ=   -40.5160   YZ=    56.2208   ZZ=    65.2109
Eigenvalues:  -111.1691   -60.9217    96.8013
  23  C    Isotropic =   127.1178   Anisotropy =    30.0976
XX=   124.8584   YX=    -9.3907   ZX=    -5.6744
XY=     0.3057   YY=   145.9815   ZY=    -4.1284
XZ=    -6.5901   YZ=     7.8593   ZZ=   110.5135
Eigenvalues:   108.2485   125.9220   147.1828
  24  C    Isotropic =   136.0694   Anisotropy =    27.7675
XX=   150.0522   YX=     5.2534   ZX=    -4.0896
XY=     0.7358   YY=   146.1510   ZY=    -5.0553
XZ=   -13.0891   YZ=    -9.6275   ZZ=   112.0050
Eigenvalues:   108.9879   144.6393   154.5810
  25  C    Isotropic =   142.8683   Anisotropy =    35.5613
XX=   128.6990   YX=    12.1889   ZX=     3.9841
XY=    16.3204   YY=   159.9067   ZY=     5.8610
XZ=     3.3833   YZ=     3.0628   ZZ=   139.9991
Eigenvalues:   122.9652   139.0638   166.5758
  26  C    Isotropic =   154.7389   Anisotropy =    31.7405
XX=   160.2544   YX=     1.2860   ZX=    -1.7606
XY=    10.9027   YY=   172.9302   ZY=     7.5284
XZ=     1.4337   YZ=     2.9319   ZZ=   131.0320
Eigenvalues:   130.3607   157.9567   175.8992
  27  C    Isotropic =   164.6824   Anisotropy =    29.0601
XX=   183.4835   YX=    -1.9087   ZX=    -3.7076
XY=    -2.0281   YY=   175.6541   ZY=    -3.0825
XZ=    -2.6602   YZ=    -4.5053   ZZ=   134.9095
Eigenvalues:   134.3304   175.6610   184.0558
  28  C    Isotropic =   149.0429   Anisotropy =    43.8007
XX=   147.8529   YX=     6.6902   ZX=    -2.5242
XY=     2.3269   YY=   171.3144   ZY=   -17.9837
XZ=     2.9212   YZ=   -17.5583   ZZ=   127.9615
Eigenvalues:   121.4965   147.3890   178.2434
  29  C    Isotropic =   164.0501   Anisotropy =    35.4760
XX=   155.8476   YX=    -3.2322   ZX=    -1.0970
XY=    -4.4241   YY=   148.7699   ZY=     1.3921
XZ=    -2.7319   YZ=     1.0046   ZZ=   187.5326
Eigenvalues:   147.0928   157.3567   187.7007
  30  C    Isotropic =   165.1162   Anisotropy =    31.6223
XX=   156.9263   YX=    12.6107   ZX=    -0.3596
XY=     7.5580   YY=   154.7911   ZY=     5.9520
XZ=     2.8436   YZ=     9.9512   ZZ=   183.6313
Eigenvalues:   145.0158   164.1351   186.1977
  31  O    Isotropic =  -281.7290   Anisotropy =   965.2505
XX=  -747.5499   YX=   -10.7819   ZX=  -307.4803
XY=   -52.1795   YY=  -281.0885   ZY=   219.6120
XZ=  -327.3955   YZ=   236.3395   ZZ=   183.4514
```

```
Eigenvalues:  -848.1268  -358.8316   361.7714
  32  C    Isotropic =   159.7828   Anisotropy =    36.6667
XX=   159.0104   YX=     1.3648   ZX=    14.4547
XY=    -0.7359   YY=   146.2560   ZY=     3.8625
XZ=    16.8808   YZ=     3.6434   ZZ=   174.0820
Eigenvalues:   145.2087   149.9125   184.2273
  33  H    Isotropic =    25.0770   Anisotropy =     9.4262
XX=    27.5645   YX=     1.4904   ZX=     4.5610
XY=     1.6060   YY=    24.1079   ZY=     2.2407
XZ=     4.3606   YZ=     2.3579   ZZ=    23.5585
Eigenvalues:    20.2975    23.5723    31.3611
  34  H    Isotropic =    24.8728   Anisotropy =     8.3597
XX=    27.8837   YX=    -2.1951   ZX=     3.6853
XY=    -2.4415   YY=    24.7711   ZY=    -0.6183
XZ=     3.1200   YZ=    -0.7555   ZZ=    21.9636
Eigenvalues:    20.3836    23.7889    30.4459
  35  H    Isotropic =    28.7623   Anisotropy =     6.1171
XX=    29.8444   YX=    -1.7404   ZX=    -0.1630
XY=    -0.2263   YY=    27.2404   ZY=     3.6303
XZ=    -0.8809   YZ=     4.6804   ZZ=    29.2022
Eigenvalues:    23.9174    29.5292    32.8404
  36  H    Isotropic =    29.0572   Anisotropy =    10.4189
XX=    30.6312   YX=     4.5270   ZX=     2.4886
XY=     4.3659   YY=    32.2661   ZY=    -0.0620
XZ=     2.2528   YZ=    -2.3085   ZZ=    24.2742
Eigenvalues:    22.6794    28.4890    36.0031
  37  H    Isotropic =    29.9273   Anisotropy =     6.7843
XX=    29.8895   YX=    -1.6331   ZX=     1.4386
XY=    -1.7173   YY=    33.4418   ZY=    -1.4562
XZ=     1.1105   YZ=    -1.0740   ZZ=    26.4506
Eigenvalues:    25.9602    29.3715    34.4502
  38  H    Isotropic =    29.9670   Anisotropy =     8.0930
XX=    29.3410   YX=     3.6385   ZX=    -2.5563
XY=     2.7301   YY=    27.7163   ZY=    -2.6760
XZ=    -0.8000   YZ=    -2.5428   ZZ=    32.8436
Eigenvalues:    25.0787    29.4599    35.3623
  39  H    Isotropic =    29.4433   Anisotropy =     7.5022
XX=    31.9210   YX=    -2.5885   ZX=    -2.8080
XY=    -2.0048   YY=    28.0383   ZY=     0.1153
XZ=    -4.2819   YZ=    -2.2195   ZZ=    28.3706
Eigenvalues:    24.7474    29.1378    34.4448
  40  H    Isotropic =    28.5886   Anisotropy =     4.2578
XX=    28.7463   YX=     0.8291   ZX=     1.5833
XY=    -0.4499   YY=    28.8798   ZY=     1.2908
XZ=     3.9207   YZ=     0.4848   ZZ=    28.1397
Eigenvalues:    25.5849    28.7537    31.4271
  41  H    Isotropic =    29.3395   Anisotropy =     5.8429
XX=    32.4718   YX=     0.9328   ZX=    -0.1245
XY=     3.5077   YY=    26.6278   ZY=    -0.1711
XZ=    -0.5671   YZ=     0.6229   ZZ=    28.9191
Eigenvalues:    25.8456    28.9382    33.2348
  42  H    Isotropic =    29.0406   Anisotropy =    11.2642
XX=    33.4826   YX=    -1.7657   ZX=    -4.3288
XY=    -3.0397   YY=    29.9519   ZY=     2.4486
XZ=    -3.7368   YZ=     3.1767   ZZ=    23.6873
Eigenvalues:    21.7840    28.7878    36.5501
  43  H    Isotropic =    29.8872   Anisotropy =     8.7889
XX=    30.9896   YX=    -2.8572   ZX=     2.0683
XY=    -4.3057   YY=    33.0483   ZY=     0.7471
XZ=     1.4296   YZ=     2.1374   ZZ=    25.6238
Eigenvalues:    24.3289    29.5863    35.7465
  44  H    Isotropic =    29.2400   Anisotropy =     6.5060
XX=    26.8918   YX=    -2.3807   ZX=     0.4253
XY=    -1.2686   YY=    28.0588   ZY=     1.9528
XZ=     1.6996   YZ=     2.2097   ZZ=    32.7694
Eigenvalues:    24.9825    29.1601    33.5773
  45  H    Isotropic =    29.3429   Anisotropy =     9.8827
XX=    34.9924   YX=     1.8195   ZX=    -1.2170
XY=     2.1598   YY=    30.0422   ZY=     0.2198
XZ=    -2.2150   YZ=    -1.0467   ZZ=    22.9942
Eigenvalues:    22.7510    29.3464    35.9314
  46  H    Isotropic =    27.8854   Anisotropy =     9.0012
XX=    30.9316   YX=    -1.1198   ZX=     4.5005
XY=    -2.8514   YY=    26.8106   ZY=    -0.9022
XZ=     4.0465   YZ=     0.2329   ZZ=    25.9139
Eigenvalues:    23.3130    26.4569    33.8861
  47  H    Isotropic =    28.1490   Anisotropy =     8.3672
XX=    30.5975   YX=    -4.1375   ZX=    -1.6656
XY=    -2.8144   YY=    28.0247   ZY=     3.0240
```

```
XZ=    -1.1275   YZ=     1.1968   ZZ=    25.8246
Eigenvalues:    24.4346    26.2852    33.7271
  48  H    Isotropic =    28.1492   Anisotropy =     7.9314
XX=    26.1978   YX=    -2.0381   ZX=     1.4428
XY=     0.1701   YY=    30.9888   ZY=    -3.1836
XZ=     1.9060   YZ=    -3.7253   ZZ=    27.2611
Eigenvalues:    24.5203    26.4905    33.4368
  49  H    Isotropic =    30.6626   Anisotropy =     6.9621
XX=    34.7350   YX=     1.3026   ZX=     1.9794
XY=     1.5954   YY=    29.7935   ZY=     1.9534
XZ=    -0.8492   YZ=     2.4505   ZZ=    27.4591
Eigenvalues:    26.1257    30.5580    35.3040
  50  H    Isotropic =    30.8064   Anisotropy =     9.0172
XX=    31.2935   YX=    -2.0110   ZX=    -3.9604
XY=    -0.9760   YY=    29.4762   ZY=     2.0838
XZ=    -4.1277   YZ=     3.5573   ZZ=    31.6494
Eigenvalues:    26.9084    28.6929    36.8178
  51  H    Isotropic =    31.0062   Anisotropy =     6.9085
XX=    28.4715   YX=    -1.0333   ZX=    -2.5326
XY=    -1.2504   YY=    34.7766   ZY=    -2.3047
XZ=    -2.3120   YZ=    -2.0901   ZZ=    29.7704
Eigenvalues:    26.0248    31.3819    35.6119
  52  H    Isotropic =    31.4140   Anisotropy =    15.1494
XX=    35.4494   YX=    -2.2345   ZX=     7.7156
XY=    -3.1601   YY=    29.6563   ZY=    -2.6882
XZ=     7.6327   YZ=    -1.1454   ZZ=    29.1362
Eigenvalues:    23.9931    28.7352    41.5136
  53  H    Isotropic =    30.7043   Anisotropy =     7.0685
XX=    33.8789   YX=     2.4317   ZX=    -2.4253
XY=     2.6187   YY=    30.9202   ZY=     0.5744
XZ=    -1.0744   YZ=     2.2484   ZZ=    27.3137
Eigenvalues:    25.9846    30.7117    35.4167
  54  H    Isotropic =    30.9097   Anisotropy =     4.7810
XX=    31.1385   YX=    -3.4618   ZX=     1.4984
XY=    -2.3322   YY=    30.9622   ZY=     0.2751
XZ=     1.5277   YZ=     0.8957   ZZ=    30.6284
Eigenvalues:    27.4526    31.1795    34.0970
  55  H    Isotropic =    30.6594   Anisotropy =     9.0815
XX=    28.9840   YX=     0.0598   ZX=    -0.0858
XY=    -0.2666   YY=    30.1886   ZY=     4.4957
XZ=    -1.2155   YZ=     5.5145   ZZ=    32.8056
Eigenvalues:    26.2864    28.9781    36.7137
  56  H    Isotropic =    29.5795   Anisotropy =     4.7436
XX=    31.1393   YX=     2.6662   ZX=     1.5988
XY=     2.6352   YY=    28.0090   ZY=    -0.2764
XZ=     0.8769   YZ=    -2.0686   ZZ=    29.5902
Eigenvalues:    25.7887    30.2079    32.7419
  57  H    Isotropic =    28.4696   Anisotropy =    13.1744
XX=    34.5448   YX=     3.4485   ZX=    -2.9643
XY=     2.7783   YY=    29.1062   ZY=    -0.8479
XZ=    -5.8661   YZ=    -1.3084   ZZ=    21.7579
Eigenvalues:    20.3802    27.7761    37.2525
  58  H    Isotropic =    29.6259   Anisotropy =     8.4967
XX=    31.9999   YX=     0.4115   ZX=     4.2635
XY=     1.3922   YY=    27.7193   ZY=     0.7142
XZ=     4.3533   YZ=     0.7160   ZZ=    29.1585
Eigenvalues:    26.0409    27.5464    35.2904
  59  H    Isotropic =    30.3733   Anisotropy =     9.6424
XX=    34.9264   YX=     1.3281   ZX=    -3.9412
XY=     1.4415   YY=    28.7732   ZY=    -0.2264
XZ=    -3.7438   YZ=    -0.5974   ZZ=    27.4201
Eigenvalues:    25.7931    28.5252    36.8015
  60  H    Isotropic =    30.0388   Anisotropy =    11.6892
XX=    27.7859   YX=     1.8226   ZX=    -1.7979
XY=     1.8096   YY=    32.7206   ZY=    -5.9700
XZ=    -1.8373   YZ=    -5.6608   ZZ=    29.6098
Eigenvalues:    25.0931    27.1916    37.8315
  61  H    Isotropic =    29.9993   Anisotropy =    11.7055
XX=    27.8257   YX=     2.3271   ZX=     1.0875
XY=     2.3484   YY=    35.0172   ZY=     4.8341
XZ=     0.8133   YZ=     4.4422   ZZ=    27.1550
Eigenvalues:    24.9999    27.1951    37.8029
  62  H    Isotropic =    30.0353   Anisotropy =     7.3207
XX=    30.6320   YX=    -0.5627   ZX=     3.3480
XY=    -0.9697   YY=    29.1329   ZY=    -3.2625
XZ=     3.0839   YZ=    -2.5720   ZZ=    30.3412
Eigenvalues:    26.1094    29.0809    34.9158
  63  H    Isotropic =    30.2288   Anisotropy =     8.2887
XX=    32.0393   YX=    -2.9816   ZX=    -2.3747
```

```
XY=    -2.8267   YY=    32.4872   ZY=     1.3036
XZ=    -2.4003   YZ=     0.6346   ZZ=    26.1599
Eigenvalues:    25.3120    29.6198    35.7546
  64  H    Isotropic =    30.6828   Anisotropy =     5.6381
XX=    27.1381   YX=    -0.7196   ZX=     1.3995
XY=    -0.1876   YY=    32.2837   ZY=    -1.5698
XZ=     0.6093   YZ=    -2.0849   ZZ=    32.6265
Eigenvalues:    26.9566    30.6502    34.4415
  65  H    Isotropic =    30.7680   Anisotropy =     8.4891
XX=    29.6389   YX=     3.9466   ZX=     1.2669
XY=     3.7889   YY=    29.4054   ZY=     2.2576
XZ=     1.5158   YZ=     3.6106   ZZ=    33.2596
Eigenvalues:    25.4578    30.4187    36.4274
  66  H    Isotropic =    31.1709   Anisotropy =     8.6482
XX=    32.1432   YX=    -0.2857   ZX=    -3.1158
XY=    -0.6508   YY=    29.2355   ZY=     2.1832
XZ=    -5.3462   YZ=     2.7077   ZZ=    32.1340
Eigenvalues:    26.8811    29.6952    36.9364
  67  H    Isotropic =    31.0963   Anisotropy =     9.3721
XX=    31.8240   YX=    -1.0700   ZX=    -4.3855
XY=    -1.5358   YY=    29.2485   ZY=     1.6616
XZ=    -5.3446   YZ=     1.1592   ZZ=    32.2164
Eigenvalues:    27.1504    28.7941    37.3444
  68  H    Isotropic =    30.7505   Anisotropy =     6.2979
XX=    29.4042   YX=     3.8417   ZX=    -1.1353
XY=     3.4804   YY=    31.3686   ZY=     2.4433
XZ=     0.3306   YZ=     2.0895   ZZ=    31.4786
Eigenvalues:    26.0353    31.2671    34.9491
  69  H    Isotropic =    30.6783   Anisotropy =     7.7451
XX=    26.6800   YX=    -0.3766   ZX=    -0.2845
XY=    -1.2459   YY=    30.8679   ZY=    -2.0286
XZ=     0.3546   YZ=    -3.1187   ZZ=    34.4872
Eigenvalues:    26.4990    29.6943    35.8418
  70  H    Isotropic =    30.6592   Anisotropy =     7.3038
XX=    32.6010   YX=    -3.1592   ZX=     1.1743
XY=    -3.4446   YY=    29.2748   ZY=    -1.6682
XZ=     1.0968   YZ=    -2.8727   ZZ=    30.1018
Eigenvalues:    26.6233    29.8258    35.5284
  71  H    Isotropic =    30.1875   Anisotropy =     6.9285
XX=    29.4450   YX=     4.7598   ZX=     0.0806
XY=     3.8928   YY=    31.2889   ZY=    -0.1255
XZ=    -0.1491   YZ=     0.9084   ZZ=    29.8286
Eigenvalues:    25.9244    29.8317    34.8065
  72  H    Isotropic =    30.6099   Anisotropy =     8.9358
XX=    28.6281   YX=     0.8215   ZX=    -1.6735
XY=     0.3058   YY=    27.1748   ZY=    -0.2010
XZ=    -2.3137   YZ=    -0.7324   ZZ=    36.0269
Eigenvalues:    26.9757    28.2871    36.5671
```

Chemical Shielding tensors (GIAO MPW1PW91/6-311+G(2d,p) iefpcm = DMSO)
3S*,7S*,11R*-1b

Conf 3 (49 %)

```
    1  C    Isotropic =    72.8958   Anisotropy =   130.9830
XX=    71.6122   YX=    19.6490   ZX=   -57.4528
XY=     2.7807   YY=    35.4385   ZY=    46.9482
XZ=   -48.2582   YZ=    58.1589   ZZ=   111.6366
Eigenvalues:    -8.3854    66.8549   160.2178
    2  C    Isotropic =    27.4929   Anisotropy =   128.2581
XX=   -13.3201   YX=   -25.7359   ZX=   -63.1683
XY=   -28.3892   YY=    24.0134   ZY=    15.5406
XZ=   -62.4674   YZ=    15.1514   ZZ=    71.7855
Eigenvalues:   -50.6208    20.1013   112.9983
    3  C    Isotropic =    67.4958   Anisotropy =   126.4549
XX=    74.4691   YX=   -44.3964   ZX=   -29.6431
XY=   -37.0452   YY=     9.1145   ZY=    30.1418
XZ=   -37.5457   YZ=    28.6862   ZZ=   118.9039
Eigenvalues:   -11.6905    62.3789   151.7991
    4  C    Isotropic =    60.6888   Anisotropy =   169.7838
XX=    44.7747   YX=    -1.6168   ZX=   -74.4284
XY=    -5.2951   YY=    17.1466   ZY=    52.2773
XZ=   -55.2501   YZ=    59.4187   ZZ=   120.1450
Eigenvalues:   -19.7455    27.9338   173.8780
    5  C    Isotropic =    34.4245   Anisotropy =   118.3110
XX=    -8.9272   YX=   -27.3680   ZX=   -61.3657
XY=   -28.6517   YY=    40.3026   ZY=    10.3876
XZ=   -59.1404   YZ=    17.8105   ZZ=    71.8982
Eigenvalues:   -45.1003    35.0753   113.2985
    6  C    Isotropic =    53.9148   Anisotropy =   164.6205
XX=    49.7050   YX=   -40.0039   ZX=   -51.5270
XY=   -35.0046   YY=    -7.2828   ZY=    41.5867
XZ=   -55.0814   YZ=    34.2842   ZZ=   119.3222
Eigenvalues:   -26.9240    25.0066   163.6618
    7  C    Isotropic =   160.7321   Anisotropy =    19.8168
XX=   165.6421   YX=    -5.2057   ZX=     3.9502
XY=    -8.6018   YY=   158.4895   ZY=    -3.8286
XZ=    11.4260   YZ=    -1.8296   ZZ=   158.0646
Eigenvalues:   152.7134   155.5396   173.9433
    8  C    Isotropic =   153.9884   Anisotropy =     9.3476
XX=   148.0355   YX=     3.7609   ZX=    -3.5652
XY=    -4.8312   YY=   157.0343   ZY=     7.3450
XZ=     3.1886   YZ=    -0.8794   ZZ=   156.8953
Eigenvalues:   148.0038   153.7413   160.2201
    9  C    Isotropic =   103.4813   Anisotropy =    46.9310
XX=   103.1209   YX=   -12.0807   ZX=    13.6544
XY=   -14.5336   YY=    95.1224   ZY=   -19.3819
XZ=     7.1273   YZ=   -22.9598   ZZ=   112.2007
Eigenvalues:    79.3466    96.3287   134.7687
   10  O    Isotropic =   189.5549   Anisotropy =   101.8438
XX=   191.6327   YX=     7.0639   ZX=   -41.6200
XY=     8.6448   YY=   156.1819   ZY=    43.0863
XZ=   -52.2553   YZ=     3.5439   ZZ=   220.8502
Eigenvalues:   135.6126   175.6013   257.4508
   11  C    Isotropic =   146.1883   Anisotropy =    45.7299
XX=   145.4043   YX=    17.1289   ZX=    10.3670
XY=    10.8039   YY=   167.4313   ZY=     1.4004
XZ=    13.3454   YZ=    11.6411   ZZ=   125.7294
Eigenvalues:   120.1608   141.7293   176.6749
   12  C    Isotropic =    40.8215   Anisotropy =   145.5230
XX=    41.3187   YX=    70.3575   ZX=    63.8585
XY=    72.5977   YY=    23.2662   ZY=     8.0113
XZ=    58.6664   YZ=     1.8814   ZZ=    57.8796
Eigenvalues:   -53.8091    38.4367   137.8368
   13  C    Isotropic =    26.7400   Anisotropy =   176.9246
XX=    30.3231   YX=    74.8940   ZX=    83.1306
XY=    72.9022   YY=     5.8441   ZY=    17.4138
XZ=    83.7953   YZ=    -4.9571   ZZ=    44.0529
Eigenvalues:   -81.2888    16.8190   144.6898
   14  C    Isotropic =   137.6662   Anisotropy =    31.8308
XX=   156.1232   YX=     3.5590   ZX=   -11.3224
XY=     6.8255   YY=   120.6691   ZY=     4.5776
XZ=    -3.5323   YZ=     5.7361   ZZ=   136.2065
Eigenvalues:   117.7377   136.3742   158.8868
   15  C    Isotropic =   166.3961   Anisotropy =    26.0132
XX=   163.8033   YX=     6.7467   ZX=     3.0174
XY=     7.1240   YY=   180.5481   ZY=    -4.8813
XZ=    -0.1029   YZ=    -5.5948   ZZ=   154.8369
Eigenvalues:   152.9915   162.4585   183.7383
```

```
  16  O    Isotropic =   235.6971   Anisotropy =    77.8094
 XX=   264.8299   YX=    26.5314   ZX=   -15.4388
 XY=    61.2667   YY=   198.1722   ZY=    62.9638
 XZ=   -27.0589   YZ=    40.0445   ZZ=   244.0891
 Eigenvalues:   145.1321   274.3891   287.5700
  17  C    Isotropic =   132.1095   Anisotropy =    72.5252
 XX=   131.9972   YX=   -29.2047   ZX=    22.4218
 XY=   -25.5618   YY=   131.6262   ZY=   -22.3032
 XZ=    20.8804   YZ=   -24.5156   ZZ=   132.7050
 Eigenvalues:   104.2451   111.6238   180.4596
  18  C    Isotropic =   110.1647   Anisotropy =    47.3314
 XX=   131.0298   YX=     2.8722   ZX=   -20.8674
 XY=    -0.9484   YY=    99.7254   ZY=    -3.4939
 XZ=   -21.1121   YZ=    -4.0439   ZZ=    99.7390
 Eigenvalues:    88.4329   100.3422   141.7190
  19  C    Isotropic =   160.3296   Anisotropy =    35.6914
 XX=   154.4781   YX=     5.4846   ZX=     3.8134
 XY=    -0.6471   YY=   142.8244   ZY=     4.4692
 XZ=     0.6284   YZ=     1.8448   ZZ=   183.6864
 Eigenvalues:   142.1691   154.6959   184.1239
  20  O    Isotropic =   236.2591   Anisotropy =    64.7785
 XX=   212.9671   YX=     5.9567   ZX=    -8.5972
 XY=     3.2209   YY=   223.4930   ZY=    13.6236
 XZ=   -21.3517   YZ=    17.3494   ZZ=   272.3171
 Eigenvalues:   205.2213   224.1112   279.4447
  21  C    Isotropic =   153.4209   Anisotropy =    49.7787
 XX=   147.5416   YX=   -18.9776   ZX=     7.1092
 XY=   -18.5395   YY=   169.5952   ZY=   -14.8252
 XZ=     8.9299   YZ=   -14.0705   ZZ=   143.1259
 Eigenvalues:   136.6269   137.0291   186.6067
  22  C    Isotropic =   -29.3753   Anisotropy =   190.0990
 XX=   -28.0348   YX=    79.9274   ZX=    61.9460
 XY=    77.7048   YY=     0.1519   ZY=    32.0617
 XZ=    63.9771   YZ=    43.7743   ZZ=   -60.2429
 Eigenvalues:  -114.6793   -70.8039    97.3574
  23  C    Isotropic =   126.4562   Anisotropy =    30.4625
 XX=   121.1844   YX=    -0.8320   ZX=    -6.2827
 XY=   -16.1967   YY=   143.5813   ZY=     2.0622
 XZ=    -8.4949   YZ=    -0.5037   ZZ=   114.6030
 Eigenvalues:   109.2911   123.3130   146.7646
  24  C    Isotropic =   135.6663   Anisotropy =    27.4731
 XX=   139.1468   YX=    -9.9447   ZX=   -22.5395
 XY=    -6.0642   YY=   139.1190   ZY=   -12.6605
 XZ=   -16.0799   YZ=    -5.9812   ZZ=   128.7332
 Eigenvalues:   108.9509   144.0664   153.9817
  25  C    Isotropic =   139.5646   Anisotropy =    38.0633
 XX=   141.4731   YX=   -14.4302   ZX=    -8.5477
 XY=   -13.4195   YY=   139.3238   ZY=    18.4788
 XZ=    -5.3476   YZ=    14.9223   ZZ=   137.8969
 Eigenvalues:   120.4440   133.3097   164.9401
  26  C    Isotropic =   154.8167   Anisotropy =    32.0960
 XX=   144.8115   YX=   -10.5527   ZX=   -18.2601
 XY=   -11.2823   YY=   164.3164   ZY=     9.0204
 XZ=   -16.2574   YZ=    -1.7942   ZZ=   155.3222
 Eigenvalues:   130.6263   157.6098   176.2141
  27  C    Isotropic =   164.9700   Anisotropy =    28.3161
 XX=   157.1185   YX=    -9.5667   ZX=   -18.7035
 XY=    -8.1736   YY=   173.8054   ZY=   -12.5546
 XZ=   -19.3760   YZ=   -13.4556   ZZ=   163.9859
 Eigenvalues:   135.1991   175.8635   183.8473
  28  C    Isotropic =   148.9671   Anisotropy =    44.2300
 XX=   158.2634   YX=   -25.2727   ZX=    -7.7995
 XY=   -21.8561   YY=   149.5452   ZY=    -6.5039
 XZ=   -14.4279   YZ=    -5.7637   ZZ=   139.0926
 Eigenvalues:   121.7157   146.7319   178.4537
  29  C    Isotropic =   164.2012   Anisotropy =    36.2010
 XX=   172.5277   YX=    12.5499   ZX=    15.2199
 XY=    12.6796   YY=   159.6341   ZY=     2.1859
 XZ=    16.0272   YZ=     3.3428   ZZ=   160.4418
 Eigenvalues:   147.0162   157.2522   188.3352
  30  C    Isotropic =   164.4095   Anisotropy =    32.0602
 XX=   168.6821   YX=     9.4927   ZX=    10.1810
 XY=     9.5044   YY=   153.9115   ZY=    12.1277
 XZ=     4.4693   YZ=    15.4798   ZZ=   170.6349
 Eigenvalues:   145.0386   162.4068   185.7830
  31  O    Isotropic =  -258.6251   Anisotropy =   926.9569
 XX=   -74.9499   YX=   257.2012   ZX=   240.7453
 XY=   262.8885   YY=  -127.6204   ZY=   348.6854
 XZ=   232.4658   YZ=   399.9540   ZZ=  -573.3049
```

```
Eigenvalues:  -796.2271  -338.9943   359.3462
  32  C    Isotropic =   161.7412   Anisotropy =    40.0831
XX=   150.4412   YX=    -9.1411   ZX=   -17.4030
XY=    -8.0182   YY=   154.4635   ZY=     1.6165
XZ=   -15.3251   YZ=     2.8847   ZZ=   180.3189
Eigenvalues:   139.7896   156.9708   188.4633
  33  H    Isotropic =    25.1164   Anisotropy =     9.6733
XX=    28.1958   YX=     3.8380   ZX=     2.8215
XY=     3.8985   YY=    25.6007   ZY=     0.2397
XZ=     2.7850   YZ=     0.1658   ZZ=    21.5526
Eigenvalues:    20.1948    23.5891    31.5653
  34  H    Isotropic =    24.9586   Anisotropy =     8.4113
XX=    27.8312   YX=    -0.3827   ZX=     4.0348
XY=    -0.3653   YY=    23.6835   ZY=    -1.4813
XZ=     4.5236   YZ=    -1.0808   ZZ=    23.3612
Eigenvalues:    20.5045    23.8051    30.5662
  35  H    Isotropic =    29.0318   Anisotropy =     9.1429
XX=    29.1472   YX=     0.7415   ZX=     2.3397
XY=     2.1283   YY=    33.7281   ZY=     2.0748
XZ=     2.1301   YZ=     3.3710   ZZ=    24.2202
Eigenvalues:    22.9487    29.0197    35.1271
  36  H    Isotropic =    28.8575   Anisotropy =     8.3668
XX=    29.4420   YX=     3.2651   ZX=    -2.3484
XY=     3.2431   YY=    26.7528   ZY=    -2.4539
XZ=    -1.0243   YZ=    -4.1574   ZZ=    30.3777
Eigenvalues:    23.9315    28.2057    34.4353
  37  H    Isotropic =    29.7547   Anisotropy =    11.3039
XX=    35.0317   YX=     0.5809   ZX=    -4.4225
XY=    -0.3260   YY=    27.7799   ZY=     2.7382
XZ=    -5.0318   YZ=     3.7909   ZZ=    26.4523
Eigenvalues:    22.5584    29.4149    37.2906
  38  H    Isotropic =    30.0818   Anisotropy =     6.5434
XX=    31.7529   YX=    -0.9642   ZX=     0.7766
XY=    -1.6295   YY=    31.2945   ZY=    -3.3325
XZ=     1.8683   YZ=    -3.5548   ZZ=    27.1980
Eigenvalues:    25.1976    30.6037    34.4441
  39  H    Isotropic =    28.5976   Anisotropy =    11.6356
XX=    33.9049   YX=     3.2050   ZX=    -2.3393
XY=     3.3426   YY=    28.7715   ZY=    -3.4294
XZ=    -0.9767   YZ=    -4.6082   ZZ=    23.1163
Eigenvalues:    21.0299    28.4082    36.3547
  40  H    Isotropic =    28.6613   Anisotropy =     2.8313
XX=    28.2004   YX=    -1.5525   ZX=     2.4199
XY=     0.0388   YY=    28.0711   ZY=     0.4397
XZ=     0.0294   YZ=    -0.9798   ZZ=    29.7124
Eigenvalues:    27.1642    28.2709    30.5488
  41  H    Isotropic =    29.7877   Anisotropy =     3.8314
XX=    31.6001   YX=    -0.8789   ZX=     0.4088
XY=    -1.9396   YY=    25.9897   ZY=     0.7393
XZ=     0.4581   YZ=    -1.2458   ZZ=    31.7733
Eigenvalues:    25.6521    31.3690    32.3419
  42  H    Isotropic =    28.4001   Anisotropy =     7.1845
XX=    27.7096   YX=     3.4668   ZX=    -1.8533
XY=     2.5439   YY=    31.1919   ZY=    -0.5856
XZ=    -3.0607   YZ=     0.4347   ZZ=    26.2986
Eigenvalues:    23.9528    28.0576    33.1897
  43  H    Isotropic =    29.3304   Anisotropy =     9.5111
XX=    34.7231   YX=     1.3271   ZX=    -1.2680
XY=     1.6516   YY=    26.5330   ZY=    -2.5008
XZ=    -2.3310   YZ=    -4.0964   ZZ=    26.7350
Eigenvalues:    23.3307    28.9894    35.6711
  44  H    Isotropic =    29.8873   Anisotropy =     8.7241
XX=    31.2905   YX=    -0.9476   ZX=     3.3341
XY=    -2.3600   YY=    32.0137   ZY=    -3.6793
XZ=     3.7863   YZ=    -2.5679   ZZ=    26.3577
Eigenvalues:    23.9344    30.0242    35.7034
  45  H    Isotropic =    29.3293   Anisotropy =     5.6738
XX=    28.0170   YX=    -0.7022   ZX=     2.6208
XY=     1.1986   YY=    31.1833   ZY=     2.0788
XZ=     3.2137   YZ=     2.1474   ZZ=    28.7877
Eigenvalues:    25.1947    29.6815    33.1119
  46  H    Isotropic =    27.9177   Anisotropy =     9.0560
XX=    31.4549   YX=     1.2826   ZX=     4.0960
XY=    -0.4445   YY=    26.1896   ZY=    -1.3346
XZ=     4.7616   YZ=    -0.3342   ZZ=    26.1086
Eigenvalues:    23.3070    26.4911    33.9551
  47  H    Isotropic =    28.1710   Anisotropy =     8.2777
XX=    30.1753   YX=    -4.4366   ZX=     0.8399
XY=    -2.8850   YY=    29.8113   ZY=     0.7776
```

```
XZ=     0.4633   YZ=    -0.9500   ZZ=    24.5265
Eigenvalues:    24.4176    26.4059    33.6895
  48  H    Isotropic =    28.1896   Anisotropy =     8.0237
XX=    26.3206   YX=    -1.0190   ZX=     2.4855
XY=     1.1440   YY=    26.6413   ZY=    -2.8846
XZ=     1.6066   YZ=    -3.2578   ZZ=    31.6068
Eigenvalues:    24.5420    26.4880    33.5387
  49  H    Isotropic =    30.5854   Anisotropy =     4.6326
XX=    28.3781   YX=    -0.3230   ZX=     2.5449
XY=    -0.8791   YY=    32.9691   ZY=    -0.0221
XZ=     2.7126   YZ=    -1.6150   ZZ=    30.4090
Eigenvalues:    26.5753    31.5071    33.6738
  50  H    Isotropic =    30.6324   Anisotropy =     9.2374
XX=    29.3043   YX=     4.1377   ZX=     2.1343
XY=     2.7672   YY=    32.4736   ZY=     2.4386
XZ=     2.4828   YZ=     3.4919   ZZ=    30.1194
Eigenvalues:    26.9998    28.1068    36.7907
  51  H    Isotropic =    30.9881   Anisotropy =     6.5805
XX=    30.7219   YX=     0.3679   ZX=    -2.9621
XY=    -0.2933   YY=    29.8817   ZY=     1.4377
XZ=    -4.1110   YZ=     1.2969   ZZ=    32.3608
Eigenvalues:    27.5562    30.0331    35.3751
  52  H    Isotropic =    30.4224   Anisotropy =    15.7210
XX=    25.8916   YX=     1.5589   ZX=    -4.7641
XY=    -0.7169   YY=    29.4541   ZY=     5.6612
XZ=    -3.4801   YZ=     7.8408   ZZ=    35.9216
Eigenvalues:    22.6891    27.6751    40.9031
  53  H    Isotropic =    30.5462   Anisotropy =     7.2837
XX=    28.0407   YX=    -0.5821   ZX=     1.9849
XY=    -0.9486   YY=    30.5992   ZY=    -4.0070
XZ=     1.0053   YZ=    -1.9806   ZZ=    32.9985
Eigenvalues:    27.6216    28.6149    35.4020
  54  H    Isotropic =    30.5202   Anisotropy =     7.5428
XX=    34.0438   YX=    -2.1046   ZX=    -1.0752
XY=    -3.0650   YY=    30.9897   ZY=    -0.2251
XZ=    -0.6439   YZ=    -0.7088   ZZ=    26.5270
Eigenvalues:    26.2842    29.7276    35.5487
  55  H    Isotropic =    30.3995   Anisotropy =     9.2304
XX=    27.3962   YX=     0.5507   ZX=     0.7982
XY=     1.9393   YY=    36.3714   ZY=    -0.0511
XZ=     0.4566   YZ=    -0.7885   ZZ=    27.4307
Eigenvalues:    26.6381    28.0073    36.5530
  56  H    Isotropic =    29.1428   Anisotropy =     3.6516
XX=    30.1688   YX=    -1.5203   ZX=     0.4573
XY=    -0.2601   YY=    25.9044   ZY=     0.8116
XZ=     0.6498   YZ=     0.1892   ZZ=    31.3552
Eigenvalues:    25.6624    30.1888    31.5772
  57  H    Isotropic =    29.3023   Anisotropy =     8.7377
XX=    32.7993   YX=    -0.2262   ZX=    -4.4465
XY=    -0.0325   YY=    28.4206   ZY=    -1.7050
XZ=    -4.3662   YZ=    -0.4287   ZZ=    26.6871
Eigenvalues:    24.1438    28.6358    35.1275
  58  H    Isotropic =    29.7142   Anisotropy =     8.0521
XX=    26.8450   YX=    -0.4682   ZX=     0.6318
XY=    -1.0434   YY=    27.2623   ZY=     0.1035
XZ=     0.4307   YZ=    -0.6324   ZZ=    35.0354
Eigenvalues:    26.2616    27.7988    35.0823
  59  H    Isotropic =    30.2864   Anisotropy =     9.3601
XX=    32.0402   YX=     2.0749   ZX=    -4.4136
XY=     2.2486   YY=    29.8472   ZY=    -1.6879
XZ=    -4.6719   YZ=    -2.0969   ZZ=    28.9717
Eigenvalues:    25.6880    28.6447    36.5265
  60  H    Isotropic =    30.0171   Anisotropy =    11.8577
XX=    36.5838   YX=    -3.5506   ZX=    -0.4456
XY=    -3.8861   YY=    27.5018   ZY=    -0.8102
XZ=    -0.8292   YZ=    -0.6772   ZZ=    25.9656
Eigenvalues:    25.1437    26.9853    37.9222
  61  H    Isotropic =    30.0423   Anisotropy =    11.6858
XX=    26.2547   YX=    -1.8187   ZX=    -2.2717
XY=    -1.6374   YY=    33.1935   ZY=     5.0828
XZ=    -2.1508   YZ=     5.1190   ZZ=    30.6787
Eigenvalues:    25.3008    26.9932    37.8328
  62  H    Isotropic =    29.9606   Anisotropy =     8.0162
XX=    30.0082   YX=    -2.1723   ZX=     3.1767
XY=    -2.7277   YY=    27.9413   ZY=    -2.4620
XZ=     2.4076   YZ=    -1.8369   ZZ=    31.9323
Eigenvalues:    26.2973    28.2798    35.3047
  63  H    Isotropic =    30.2876   Anisotropy =     8.7067
XX=    28.4087   YX=    -0.0835   ZX=    -1.3197
```

```
XY=     0.2517   YY=    35.6393   ZY=    -1.8317
XZ=    -1.4651   YZ=    -2.1766   ZZ=    26.8148
Eigenvalues:    25.7093    29.0614    36.0920
  64  H    Isotropic =    30.7183   Anisotropy =     6.7698
XX=    33.5652   YX=    -2.3368   ZX=     2.1101
XY=    -2.6535   YY=    30.2532   ZY=     1.2215
XZ=     2.4005   YZ=     1.1639   ZZ=    28.3365
Eigenvalues:    26.3510    30.5724    35.2315
  65  H    Isotropic =    30.8287   Anisotropy =     7.8717
XX=    30.8183   YX=     1.7194   ZX=     0.5215
XY=     0.7203   YY=    28.0563   ZY=     4.0871
XZ=    -0.8226   YZ=     4.7097   ZZ=    33.6113
Eigenvalues:    25.3911    31.0184    36.0765
  66  H    Isotropic =    31.3950   Anisotropy =     9.0529
XX=    33.2362   YX=     4.4178   ZX=    -1.6814
XY=     3.5968   YY=    33.5898   ZY=    -0.0692
XZ=    -0.9256   YZ=     1.8888   ZZ=    27.3590
Eigenvalues:    26.5026    30.2522    37.4303
  67  H    Isotropic =    30.8618   Anisotropy =     9.0690
XX=    32.5252   YX=     3.7507   ZX=    -0.9874
XY=     4.2830   YY=    33.1208   ZY=    -0.7221
XZ=    -0.3680   YZ=    -0.0755   ZZ=    26.9395
Eigenvalues:    26.8565    28.8211    36.9078
  68  H    Isotropic =    30.5882   Anisotropy =     6.4970
XX=    30.8752   YX=    -0.3487   ZX=    -0.0587
XY=     0.7432   YY=    29.4481   ZY=     4.6767
XZ=    -1.7718   YZ=     3.8663   ZZ=    31.4412
Eigenvalues:    25.9513    30.8937    34.9195
  69  H    Isotropic =    30.6630   Anisotropy =     8.3170
XX=    34.1391   YX=    -1.9518   ZX=     4.2115
XY=    -0.4895   YY=    29.6308   ZY=     0.6107
XZ=     3.6323   YZ=     0.4202   ZZ=    28.2191
Eigenvalues:    25.9826    29.7987    36.2076
  70  H    Isotropic =    30.9575   Anisotropy =     9.7359
XX=    27.7442   YX=    -0.0262   ZX=    -0.5868
XY=    -0.5303   YY=    28.7822   ZY=    -2.7359
XZ=     0.5451   YZ=    -3.4427   ZZ=    36.3462
Eigenvalues:    27.4413    27.9831    37.4482
  71  H    Isotropic =    30.6154   Anisotropy =     7.3830
XX=    28.0310   YX=    -1.0357   ZX=    -2.4031
XY=     0.9517   YY=    32.5379   ZY=     2.7455
XZ=    -0.6579   YZ=     4.1196   ZZ=    31.2774
Eigenvalues:    26.9867    29.3221    35.5374
  72  H    Isotropic =    30.1126   Anisotropy =     6.6646
XX=    33.0690   YX=     1.1089   ZX=    -2.8263
XY=     0.6247   YY=    26.0041   ZY=     2.1699
XZ=    -1.5931   YZ=     0.8650   ZZ=    31.2648
Eigenvalues:    25.3210    30.4611    34.5557
```

11. Alignment tensors

Alignment tensor of ^{13}C RCSA analysis of

Estrone

Alignment tensor

A'x= 7.951e-05

A'y= 1.509e-04

A'z=-2.304e-04

Saupe tensor

S'x= 1.193e-04

S'y= 2.264e-04

S'z=-3.457e-04

Alignment tensor eigenvectors

e[x]=(-0.184, 0.171, 0.968)

e[y]=(0.126,-0.972, 0.196)

e[z]=(0.975, 0.158, 0.158)

Alignment tensor in laboratory coordinates:

[-2.138e-04,-5.652e-05,-4.591e-05]

[-5.652e-05,1.393e-04,-2.134e-05]

[-4.591e-05,-2.134e-05,7.453e-05]

SVD condition number is 1.429e+01

Axial component Aa = -3.457e-04

Rhombic component Ar = -7.142e-05

rhombicity R = 0.207

Asimmetry parameter etha =3.099e-01

GDO = 4.086e-04

ZY'Z'' Euler Angles (degrees)

Set 1

(9.2,80.9,168.6)

Set 2

(-170.8,-80.9,-11.4)

13-epi-estrone

Alignment tensor

A'x=-2.338e-05

A'y=-2.466e-04

A'z= 2.700e-04

Saupe tensor

S'x=-3.507e-05

S'y=-3.699e-04

S'z= 4.050e-04

Alignment tensor eigenvectors

e[x]=(0.214, 0.749, 0.627)

e[y]=(0.954,-0.024,-0.298)

e[z]=(-0.208, 0.662,-0.720)

Alignment tensor in laboratory coordinates:

[-2.140e-04,-3.537e-05,1.074e-04]

[-3.537e-05,1.049e-04,-1.414e-04]

[1.074e-04,-1.414e-04,1.090e-04]

SVD condition number is 8.665e+00

Axial component Aa = 4.050e-04

Rhombic component Ar = 2.232e-04

rhombicity R = 0.551

Asimmetry parameter etha =8.268e-01

GDO = 5.417e-04

ZY'Z'' Euler Angles (degrees)

Set 1

(107.5,136.1,-154.6)

Set 2

(-72.5,-136.1,25.4)

Bootstrapping analysis

Estrone

Distribution size=512

Distribution type=Gaussian

CSA general Std. Dev=0.0007 ppm

Alignment tensor

<A'x> = 7.345e-05 Std.Dev = 1.507e-05

<A'y> = 1.562e-04 Std.Dev = 1.525e-05

<A'z> = -2.297e-04 Std.Dev = 6.480e-06

Quality factors statistic

<Q> = 0.107

StdDev(Q) = 0.015

Highest Q = 0.170

Lowest Q = 0.063

13-epi-estrone

Distribution size=512

Distribution type=Gaussian

CSA general Std. Dev=0.0007 ppm

Alignment tensor

<A'x> = -2.803e-05 Std.Dev = 1.474e-05

<A'y> = -2.360e-04 Std.Dev = 8.037e-05

<A'z> = 2.640e-04 Std.Dev = 8.606e-05

Quality factors statistic

<Q> = 0.224

StdDev(Q) = 0.016

Highest Q = 0.273

Lowest Q = 0.180

Alignment tensor of ${}^{1}D_{CH}$ analysis of

(3*R**,7*S**,11*R**)-1b

Conformationally averaged solution
Alignment tensor
A'x= 2.068e-05
A'y= 2.660e-04
A'z=-2.867e-04
Saupe tensor
S'x= 3.102e-05
S'y= 3.991e-04
S'z=-4.301e-04
Alignment tensor eigenvectors
e[x]=(0.326, 0.779, 0.536)
e[y]=(-0.232,-0.483, 0.844)
e[z]=(0.916,-0.400, 0.024)

Alignment tensor in laboratory coordinates:
[-2.242e-04,1.401e-04,-5.476e-05]
[1.401e-04,2.895e-05,-9.721e-05]
[-5.476e-05,-9.721e-05,1.953e-04]

SVD condition number is 4.002e+00
Axial component Aa = -4.301e-04
Rhombic component Ar = -2.454e-04
rhombicity R = 0.571
Asimmetry parameter etha =8.558e-01
GDO = 5.805e-04

ZY'Z" Euler Angles (degrees)
Set 1
(-23.6,88.7,122.4)
Set 2
(156.4,-88.7,-57.6)
Grid points: 16

(3*S**,7*S**,11*R**)-1b

Conformationally averaged solution
Alignment tensor
A'x=-2.584e-06
A'y=-2.716e-04
A'z= 2.741e-04
Saupe tensor
S'x=-3.876e-06
S'y=-4.073e-04
S'z= 4.112e-04
Alignment tensor eigenvectors
e[x]=(0.076, 0.983,-0.169)
e[y]=(-0.995, 0.065,-0.069)
e[z]=(-0.057, 0.174, 0.983)

Alignment tensor in laboratory coordinates:
[-2.682e-04,1.471e-05,-3.416e-05]
[1.471e-05,4.620e-06,4.847e-05]
[-3.416e-05,4.847e-05,2.636e-04]

SVD condition number is 3.092e+00
Axial component Aa = 4.112e-04
Rhombic component Ar = 2.690e-04
rhombicity R = 0.654
Asimmetry parameter etha =9.811e-01
GDO = 5.779e-04

ZY'Z" Euler Angles (degrees)
Set 1
(108.2,10.5,-22.3)
Set 2
(-71.8,-10.5,157.7)
Grid points: 16

Alignment tensor of ^{13}C RCSA analysis of

(3*R**,7*S**,11*R**)-1b

Conformationally averaged solution
Alignment tensor
A'x=-1.209e-05
A'y=-2.882e-04
A'z= 3.003e-04
Saupe tensor
S'x=-1.814e-05
S'y=-4.323e-04
S'z= 4.504e-04
Alignment tensor eigenvectors
e[x]=(0.302, 0.728, 0.615)
e[y]=(0.910,-0.412, 0.040)
e[z]=(0.283, 0.548,-0.787)

Alignment tensor in laboratory coordinates:
[-2.158e-04,1.520e-04,-7.971e-05]
[1.520e-04,3.474e-05,-1.301e-04]
[-7.971e-05,-1.301e-04,1.811e-04]

SVD condition number is 5.570e+00
Axial component Aa = 4.504e-04
Rhombic component Ar = 2.761e-04
rhombicity R = 0.613
Asimmetry parameter etha =9.195e-01
GDO = 6.204e-04

ZY'Z'' Euler Angles (degrees)
Set 1
(62.7,141.9,176.3)
Set 2
(-117.3,-141.9,-3.7)

(3*S**,7*S**,11*R**)-1b

Conformationally averaged solution
Alignment tensor
A'x=-2.998e-05
A'y=-3.805e-04
A'z= 4.105e-04
Saupe tensor
S'x=-4.497e-05
S'y=-5.707e-04
S'z= 6.157e-04
Alignment tensor eigenvectors
e[x]=(0.064,-0.994, 0.086)
e[y]=(0.995, 0.071, 0.070)
e[z]=(-0.075, 0.081, 0.994)

Alignment tensor in laboratory coordinates:
[-3.745e-04,-2.731e-05,-5.719e-05]
[-2.731e-05,-2.882e-05,3.382e-05]
[-5.719e-05,3.382e-05,4.034e-04]

SVD condition number is 6.742e+00
Axial component Aa = 6.157e-04
Rhombic component Ar = 3.505e-04
rhombicity R = 0.569
Asimmetry parameter etha =8.539e-01
GDO = 8.305e-04

ZY'Z'' Euler Angles (degrees)
Set 1
(132.8,6.4,141.1)
Set 2
(-47.2,-6.4,-38.9)
